# Synthetic utility of functionalized alkylsilyl peroxides for Fe-catalyzed and visible-light-promoted radical transformation†

**DOI:** 10.1039/d3sc06857a

**Published:** 2024-02-22

**Authors:** Jiahao Liu, Shiyong Liu, Zhe Wang, Terumasa Kato, Yan Liu, Keiji Maruoka

**Affiliations:** a School of Chemical Engineering and Light Industry, Guangdong University of Technology Guangzhou 510006 China terumasa.kato.j97@kyoto-u.jp yanliu@gdut.edu.cn maruoka.keiji.4w@kyoto-u.ac.jp; b Guangdong Provincial Key Laboratory of Plant Resources Biorefinery, Guangdong University of Technology Guangzhou 510006 China; c Graduate School of Pharmaceutical Sciences, Kyoto University Sakyo Kyoto 606-8501 Japan

## Abstract

α-Keto-, β-acetoxy- and β-amidoalkylsilyl peroxides are prepared from various precursors and utilized for Fe-catalyzed and visible-light-promoted radical functionalization with coupling partners under mild conditions with a broad substrate scope.

## Graphical abstract

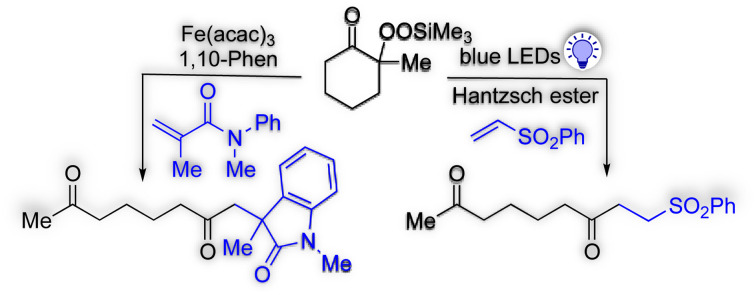

## Introduction

**Fig. 1:**
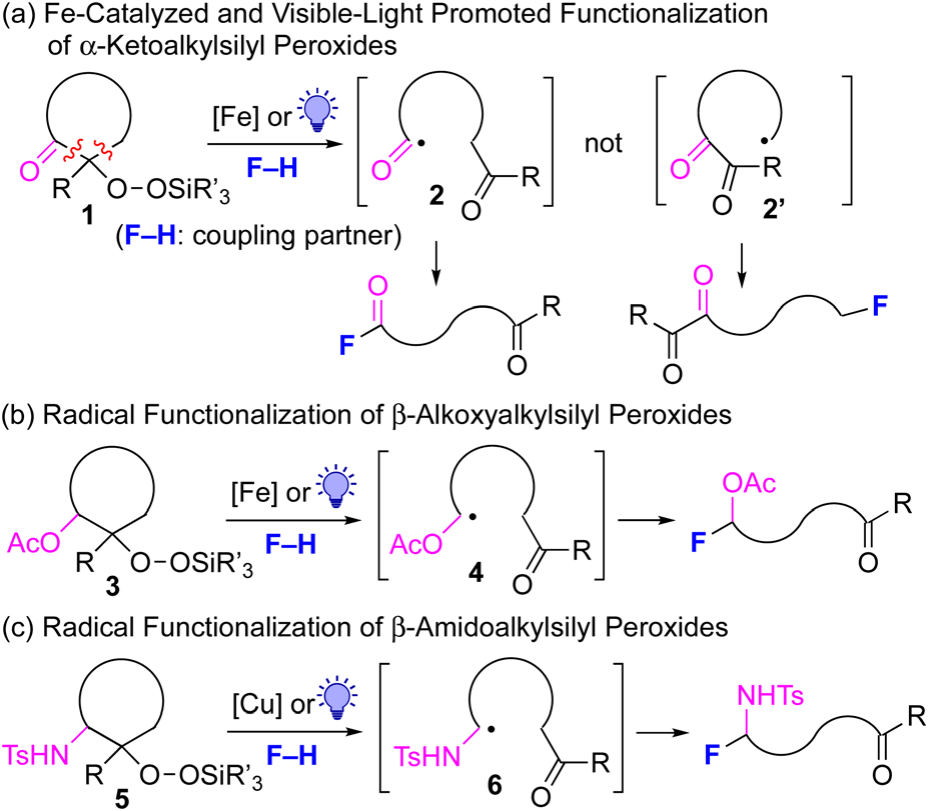
Transformation of α-ketoalkylsilyl peroxides 1 and β-acetoxyalkyl and β-amidoalkyl peroxides 3 and 5 for metal-catalyzed and visible-light-promoted functionalization.

The generation of unstabilized, reactive alkyl radicals from appropriate organic precursors under mild conditions is very important and challenging in modern radical chemistry.^1^ One of the most reliable approaches to generate such alkyl radicals is the β-scission of alkoxy radicals, which are generally prepared from the corresponding alkanols under strongly oxidative conditions.^2^ However, due to the use of strong oxidants in this method, the choice of substrates is limited by the functional group tolerance and reaction conditions. Recently, the photocatalyzed generation of alkoxy radicals has also been reported with limited success.^3^ In this context, we have recently reported the successful generation of alkyl radicals from alkylsilyl peroxides^4^ under mildly reductive conditions (reductive β-scission strategy) using Cu, Fe or Ni catalysts; the *in situ*-generated alkyl radicals were then reacted with various coupling partners to furnish new C(sp^3^)–N,^5^ C(sp^3^)–C(sp),^6^ C(sp^3^)–B,^7^ C(sp^3^)–Si,^7^ C(sp^3^)–O,^8^ C(sp^3^)–C(sp^2^),^9^ and C(sp^3^)–C(sp^3^)^10^ bonds.^11^ Thus far, we have developed this radical chemistry using alkylsilyl peroxides without any functional groups. However, if various functional groups could be introduced into the carbon skeletons of the alkylsilyl peroxides, this radical chemistry would be further enhanced to a synthetically more useful level. In this work, α-keto-substituted alkylsilyl peroxides of type 1 are prepared, and transformed into the more-stable acyl radicals 2,^12,13^ rather than the alkyl radicals 2′ using the reductive β-scission strategy (Fig. 1a). In a similar manner, β-acetoxy- and β-amido-substituted alkylsilyl peroxides of types 3 and 5 are utilized for the generation of α-acetoxyalkyl and α-amidoalkyl radicals 4 and 6, respectively (Fig. 1b and c).^14,15^ These functionalized carbon radicals, thus generated, are then utilized for subsequent transformation with various types of coupling partners (F–H), thereby providing more synthetically valuable products.

## Results and discussion

α-Ketoalkylsilyl peroxide 1a was conveniently prepared from 2-methylcyclohexanone *via* an initial hydroperoxylation and subsequent trimethylsilylation using a literature procedure (see ESI†). First, based on our previously reported conjugate addition–cyclization sequence,^16,17^ reaction of α-ketoalkylsilyl peroxide 1a as an alkyl radical source was carried out with methacrylamide 7 as a coupling partner. Thus, the reaction of α-ketoalkylsilyl peroxide 1a (1.2 equiv.) with methacrylamide 7 in dioxane in the presence of 20 mol% each of CuI and 1,10-phenanthroline (1,10-phen) at 80 °C for 4 h gave rise to desired conjugate addition–cyclization product 8a exclusively in 11% yield (entry 1 in Table 1). The use of more-Lewis-acidic Cu(MeCN)_4_BF_4_ under similar conditions afforded 8a in slightly higher yield (entry 2). Replacing the Cu catalysts with Ni(OAc)_2_·4H_2_O catalyst exhibited a similar low reactivity (entry 3). Interestingly, the addition of Fe catalysts such as FeCl_2_ and Fe(acac)_2_ enhanced the yield of 8a to 42–53% yield (entries 4 and 5), although the use of FeCl_3_ gave less satisfactory results (entry 6). Finally, the use of Fe(acac)_3_ catalyst under similar conditions exhibited good yield (entry 7). Having identified Fe(acac)_3_ as a suitable catalyst, the solvent effect was then examined. The use of DMSO solvent at low temperature afforded product 8a in higher yield (69%) than MeCN, DCE, or benzene (entry 12 *vs.* 8–11). Furthermore, 95% of 8a was obtained by using excess 1a (2 equiv.) (entry 13). For more details of the reaction optimization, see Tables S1 and S2 in the ESI.†

**Table 1:** Optimization of the synthesis of 8a from α-ketoalkylsilyl peroxide 1a and 7^a^

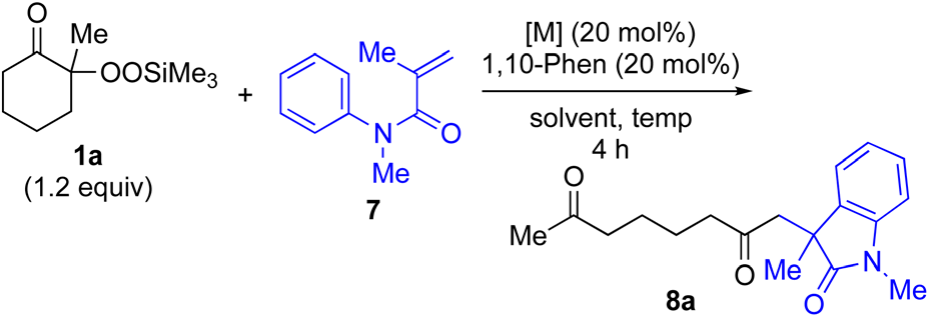				
Entry	Metal catalyst	Solvent	Temp. (°C)	Yield^b^ (%)
1	CuI	Dioxane	80	11
2	Cu(MeCN)_4_BF_4_	Dioxane	80	19
3	Ni(OAc)_2_·4H_2_O	Dioxane	80	18
4	FeCl_2_	Dioxane	80	42
5	Fe(acac)_2_	Dioxane	80	53
6	FeCl_3_	Dioxane	80	28
7	Fe(acac)_3_	Dioxane	80	59
8	Fe(acac)_3_	MeCN	80	50
9	Fe(acac)_3_	DCE	80	42
10	Fe(acac)_3_	Benzene	80	50
11	Fe(acac)_3_	DMSO	80	65
12	Fe(acac)_3_	DMSO	40	69
13^c^	Fe(acac)_3_	DMSO	40	95^d^

a The reactions of 7 (0.2 mmol) and 1a (0.24 mmol) were carried out in the presence of metal catalyst (0.04 mmol) and 1,10-phen ligand (0.04 mmol) in solvent (1 mL) at the indicated temperature for 4 h.

b The yield of 8a was determined by ^1^H NMR spectroscopy using nitromethane as an internal standard.

c 1a (2.0 equiv.).

d Isolated yield of 8a.

With the optimized conditions for the Fe(acac)_3_-catalyzed conjugate addition–cyclization sequence of 1a in hand, we subsequently examined the substrate scope of the Fe(acac)_3_-catalyzed radical functionalization of various α-ketoalkylsilyl peroxides 1a–m as shown in Table 2. Thus, the Fe(acac)_3_-catalyzed reaction of 5–8-membered α-ketoalkylsilyl peroxides 1a–d with methacrylamide 7 furnished conjugate addition–cyclization products 8a–d in high-to-excellent yield (entries 1–4). In a similar manner, aromatic-substituted α-ketoalkylsilyl peroxide 1e reacted with methacrylamide 7 to give product 8e in good yield (entry 5). Acyclic α-ketoalkylsilyl peroxide 1f also worked well (entry 6), but decarbonylation of the intermediary acyl radical took place in the case of the more-substituted substrate 1g to furnish a mixture of 8g and decarbonylated 9g in 20% and 15% yields, respectively (entry 7). Longer reaction time (24 h) enhanced the product yield of 8g (entry 8). Similarly, facile decarbonylation was observed with more-substituted substrate 1h (entries 9 and 10). Furthermore, the separate treatment of the diastereomers 1i and 1j of l-menthone-derived α-ketoalkylsilyl peroxides with methacrylamide 7 afforded the same coupling product 8i in high yield (entries 11 and 12). Ethyl-substituted 1k afforded the corresponding ethyl ketone 8k in good yield (entry 13), though phenyl-substituted 1m provided phenyl ketone 8m in low yield (entry 14).

**Table 2:** Substrate scope of the selective functionalization of various α-ketoalkylsilyl peroxides 1 with methacrylamide 7^a^

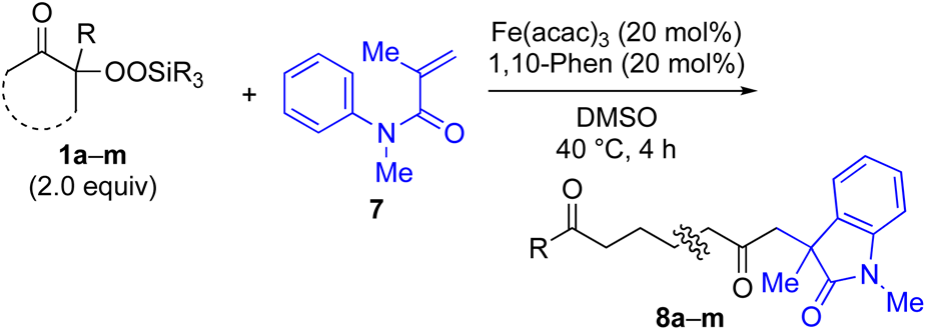			
Entry	α-Ketoalkylsilyl peroxide 1	Product 8	Yield^b^ (%)
	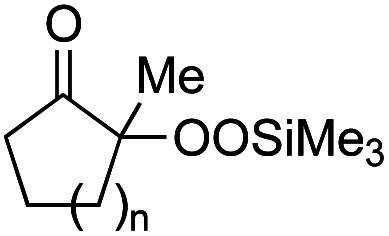	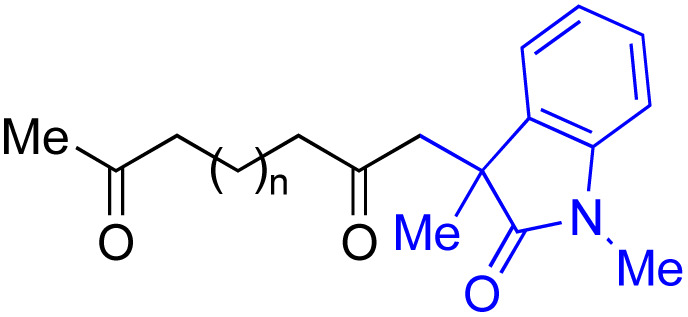	
1	1b (*n* = 1)	8b	77
2	1a (*n* = 2)	8a	95
3	1c (*n* = 3)	8c	90
4	1d (*n* = 4)	8d	84
5	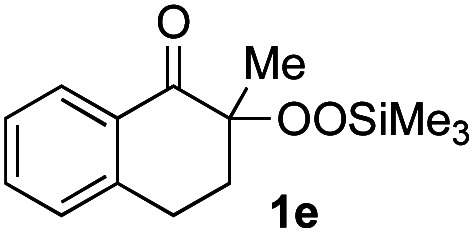	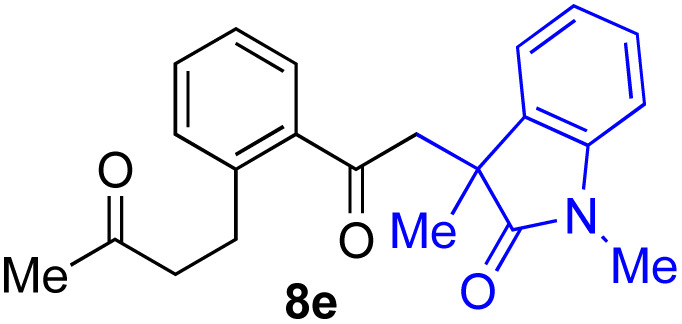	65
	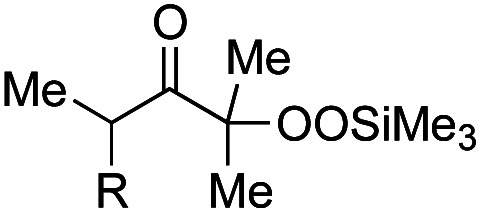	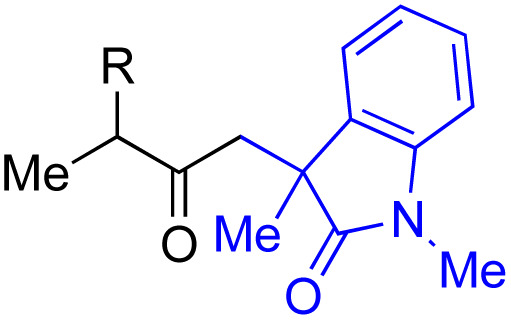	
6	1f (R = H)	8f	89
7	1g (R = Me)	8g	20 (15)^c^
8	1g (R = Me)	8g	57^d^ (40)^c^^,^^d^
9	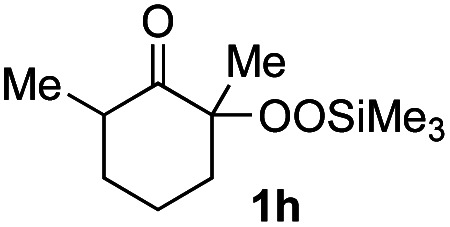	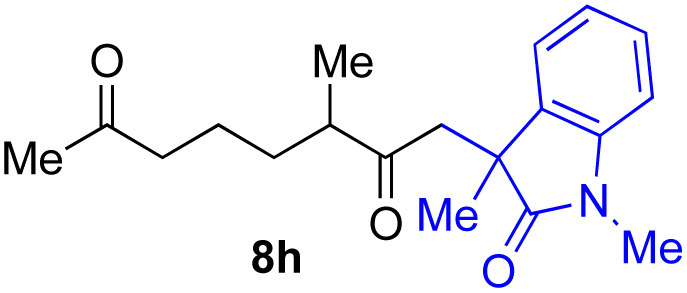	18 (0)^c^
10			37^e^ (43)^c^^,^^e^
11	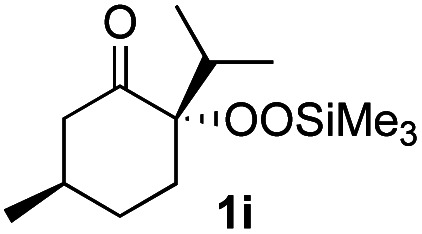	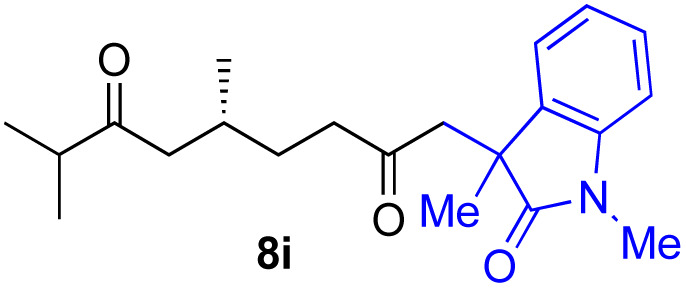	46 (99)^e^
12	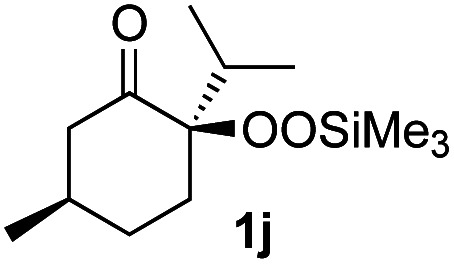	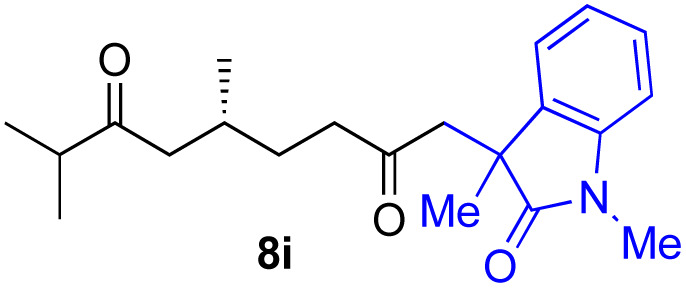	52 (86)^e^
13	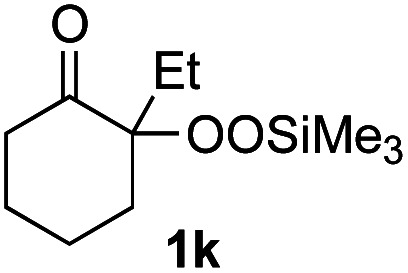	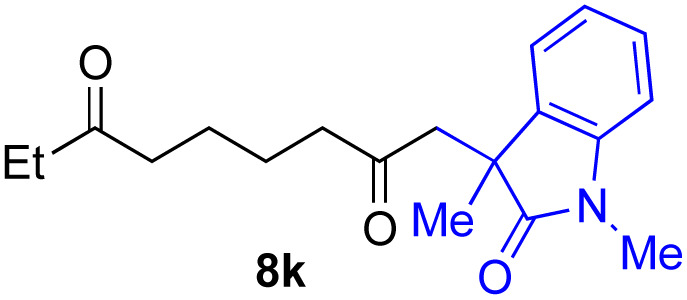	73
14	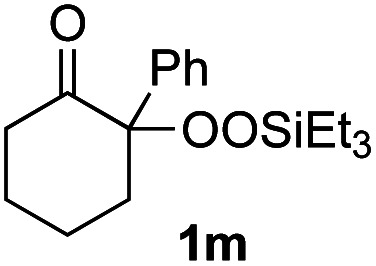	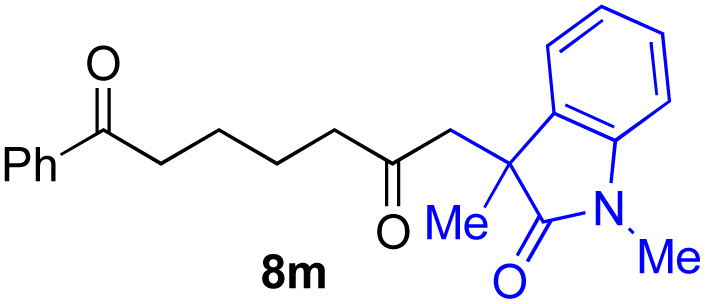	0 (27)^f^
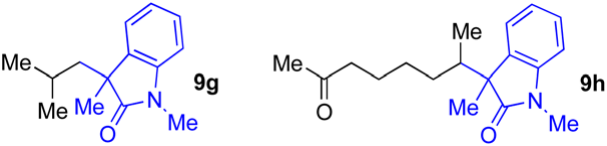			

a Unless otherwise specified, the reactions were carried out in the presence of 1 (0.4 mmol), 7 (0.2 mmol), Fe(acac)_3_ catalyst (0.04 mmol), 1,10-phen ligand (0.04 mmol) in DMSO (1 mL) at 40 °C for 4 h.

b Isolated yield.

c The yield of decarbonylation product 9g or 9h.

d For 24 h.

e For 12 h.

f At 80 °C for 16 h.

Next, we examined the substrate scope of various coupling partners (F–H) with α-ketoalkylsilyl peroxide 1a, as shown in Table 3. The Fe(acac)_3_-catalyzed reaction of α-ketoalkylsilyl peroxide 1a with 1-methylquinoxalin-2(1*H*)-one 10 furnished addition–rearomatization product 11 in high yield (entry 1). Although treatment of 1a with 2-isocyano-5-methyl-1,1′-biphenyl 12 gave addition–cyclization product 13 in very low yield under the standard conditions, the use of DMF in place of DMSO afforded 13 in high yield (entry 2). In a similar manner, while the initial reaction of 1a with cinnamic acid 14 provided the decarboxylated coupling product 15 in low yield, the use of excess 14 without the ligand 1,10-phen under otherwise similar conditions afforded 15 in good yield (entry 3). The reaction of 1a with 1,1-diphenylethylene 16 under the standard condition also gave poor results, but the FeSO_4_·7H_2_O-catalyzed reaction in DMF at 80 °C with excess 16 (3.0 equiv.) afforded 17 in good yield (entry 4). Furthermore, treatment of 1a with diethyl 2-benzylidenemalonate 18 produced the conjugate addition product 19 in moderate yield (entry 5).

**Table 3:** Substrate scope of various coupling partners with α-ketoalkylsilyl peroxide 1a^a^

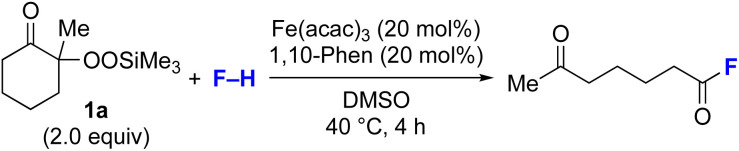			
Entry	Coupling partner (F–H)	Product	Yield^b^ (%)
1	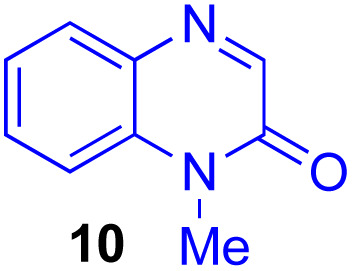	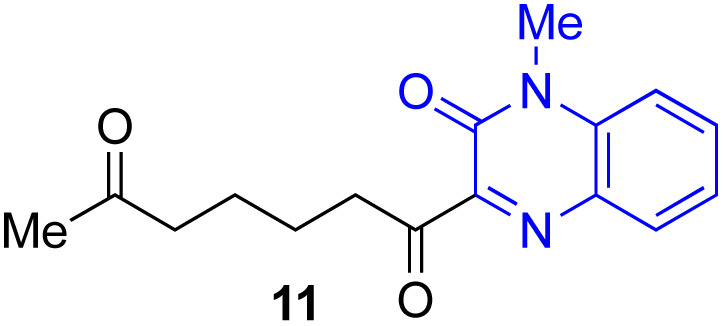	91
2	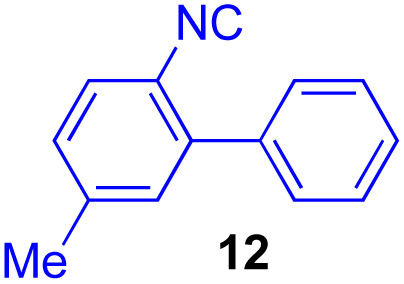	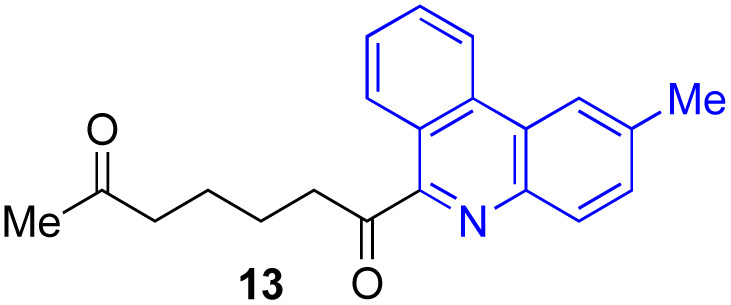	9 (78)^c^
3	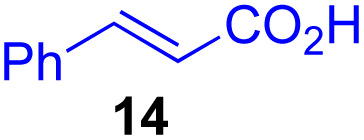	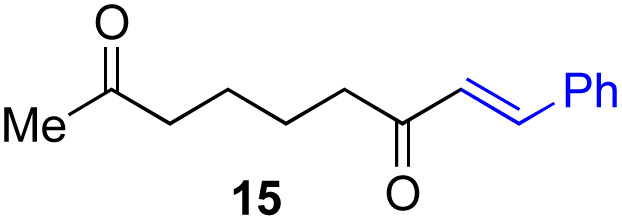	19 (56)^c^^,^^d^^,^^e^
4	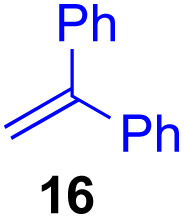	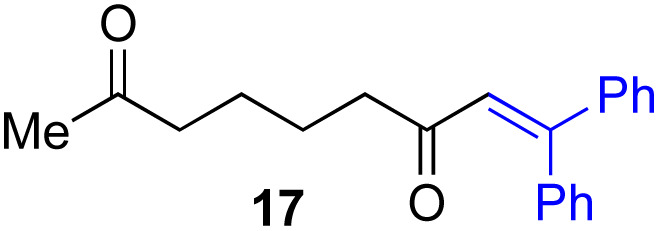	<5^f^ (66)^d^^,^^g^^,^^h^^,^^i^
5	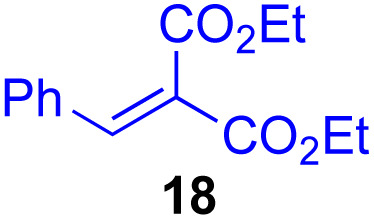	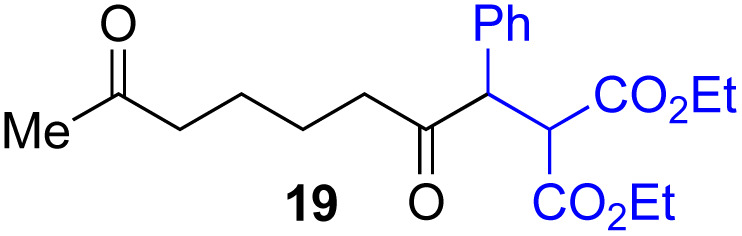	0 (43)^d^^,^^g^^,^^h^^,^^j^
6	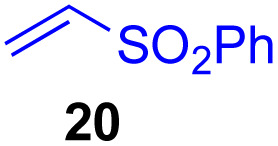	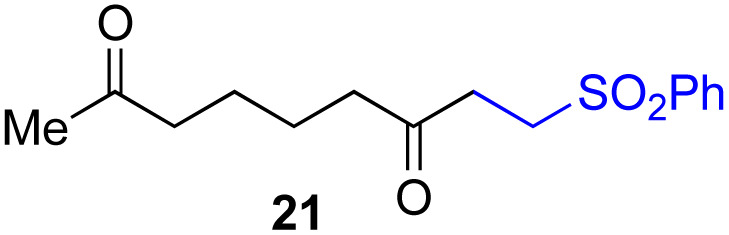	<5^h^ (34)^d^^,^^g^^,^^h^

a Unless otherwise specified, the reactions were carried out in the presence of 1a (0.4 mmol), coupling partner (0.2 mmol), Fe(acac)_3_ (0.04 mmol), 1,10-phen (0.04 mmol) in DMSO (1 mL) at 40 °C for 4 h under argon atmosphere.

b Isolated yield.

c In DMF.

d 1,10-Phen ligand was not used.

e Use of 14 (5 equiv.) at 80 °C for 24 h.

f NMR yield using nitromethane as an internal standard.

g FeSO_4_·7H_2_O was used instead of Fe(acac)_3_.

h In DMF at 80 °C.

i Use of 1a (0.2 mmol) and 16 (3.0 equiv.).

j Use of 1a (0.2 mmol) and 18 (2.0 equiv.).

**Fig. 2:**
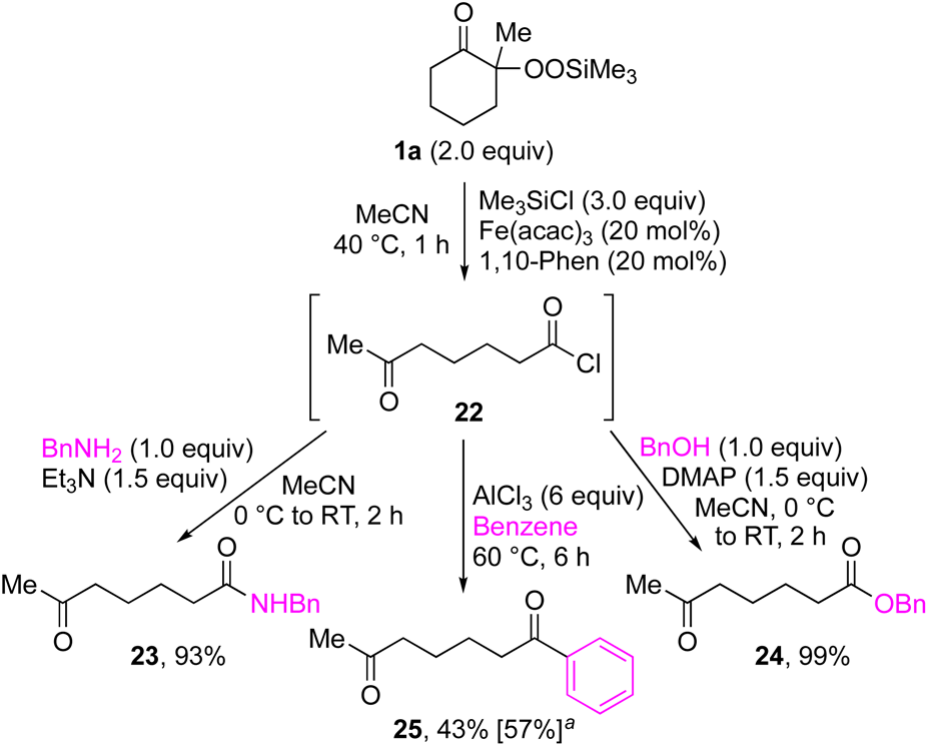
Synthetic transformations of acyl chloride 22 derived from 1a. ^*a*^Generation of 22 with Me_3_SiCl (3.0 equiv.), Fe(acac)_3_ (1 mol%), 1,10-phen (1 mol%) in CH_2_Cl_2_ at 40 °C for 1 h.

**Fig. 3:**
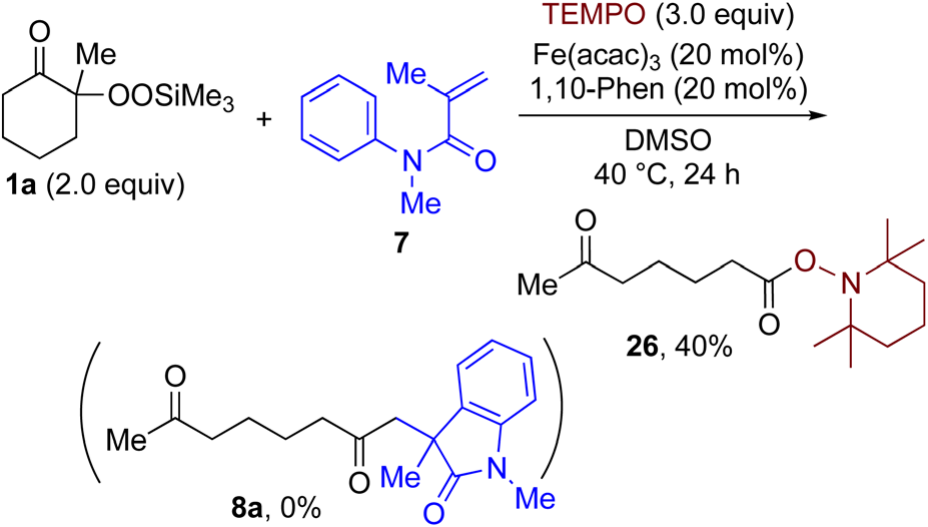
Radical trapping experiment of 1a with TEMPO.

By taking advantage of the generation of reactive acyl chloride intermediate 22 in a practical manner, several synthetically useful transformations were accomplished in a highly efficient manner, as shown in Fig. 2. Thus, treatment of the intermediary 6-oxoheptanoyl chloride (22) with BnNH_2_/NEt_3_ or BnOH/DMAP afforded the corresponding amide 23 and ester 24, respectively, in excellent yields. Even a one-mmol-scale experiment using 1a afforded 23 in 84% yield. Friedel–Crafts acylation of 22 afforded the desired phenyl ketone 25 in moderate yield. These results demonstrate that our strategy is highly versatile due to the high synthetic utility of the acyl chloride intermediates 22. A control experiment for the conjugate addition–cyclization reaction with 7 was carried out to obtain insight into the reaction mechanism: the results supported the hypothesized generation of acyl radical intermediates. Specifically, when the reaction of α-ketoalkylsilyl peroxide 1a and methyacrylamide 7 with 20 mol% each of Fe(acac)_3_ and 1,10-phen in DMSO was conducted at 40 °C for 24 h in the presence of a radical scavenger (2,2,6,6-tetramethylpiperidin-1-yl)oxy, TEMPO, the conjugate addition–cyclization reaction was significantly inhibited, and the acyl radical/TEMPO adduct 26 was obtained in 40% NMR yield (Fig. 3). This observation provides evidence that the *in situ*-generated acyl radical is most likely involved in this sequential transformation.

**Fig. 4:**
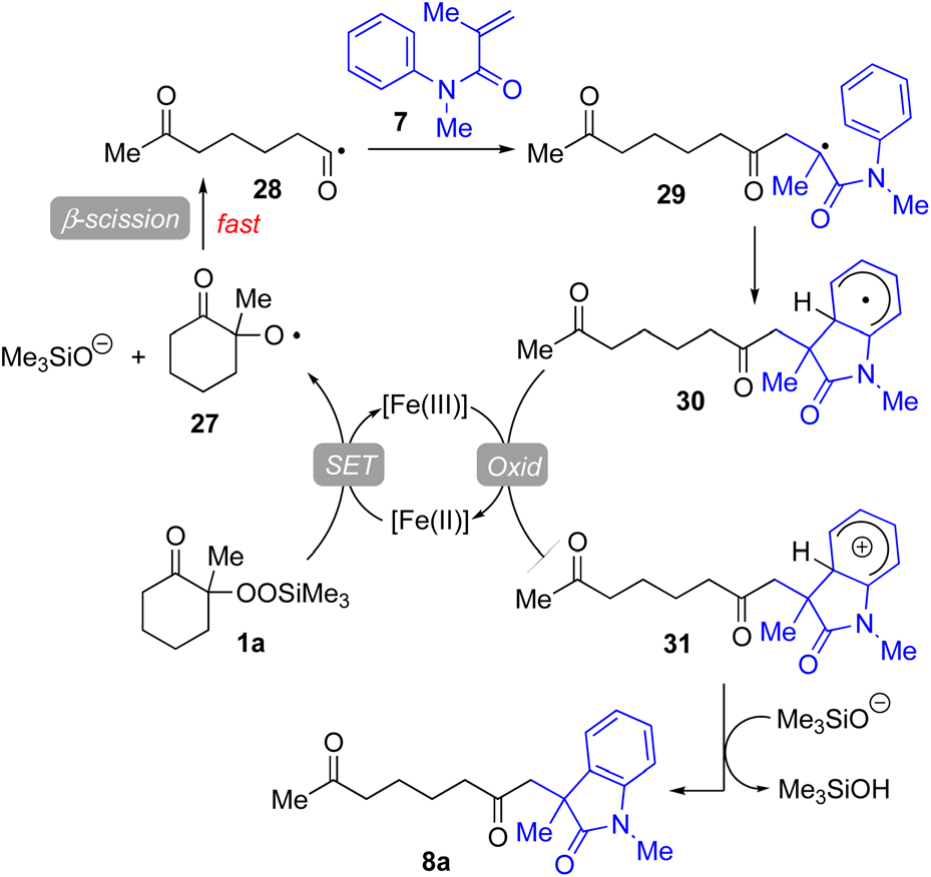
Proposed reaction mechanism for Fe-catalyzed reaction of 1a with 7.

Based on our experimental results, a plausible reaction mechanism has been proposed for the Fe(acac)_3_-catalyzed conjugate addition–cyclization sequence of methacrylamide 9 with 1a (Fig. 4). The use of Fe(ii) salts such as FeCl_2_, Fe(acac)_2_, and FeSO_4_·7H_2_O provided good to better results in the radical cleavage reaction of 1a (entries 4 and 5 in Table 1; entries 4–6 in Table 3).^16^ Thus, 1,10-phen-coordinated Fe(ii) species would cleave the O–O bond of 1a*via* single-electron transfer (SET) process, leading to alkoxy radical 27 and trimethylsilanoxide. Oxy radical 27 then easily undergoes β-scission to generate the functionalized acyl radical 28. This acyl radical 28 subsequently reacts with 7 to afford the intermediary carbon radical 29, which further adds to the benzene ring to furnish the radical intermediate 30. This radical is then oxidized by the Fe(iii) catalyst to give the corresponding carbocation species 31, which is deprotonated by trimethylsilanoxide to afford the final product 8a.

**Fig. 5:**
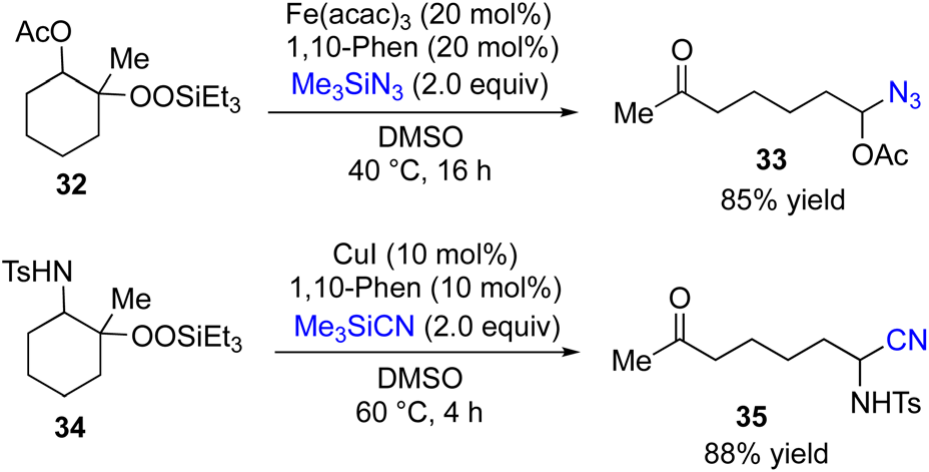
Metal-catalyzed reactions of β-acetoxy, and β-amidoalkylsilyl peroxides.

Attempted reactions of β-acetoxyalkylsilyl peroxides 32 with various coupling partners such as 7, 10, 12, 16 and 20 resulted in producing none or very low yields of desired coupling products. In contrast, the choice of Me_3_SiN_3_ as coupling partner gave the corresponding coupling product 33 in 85% yield (Fig. 5). In addition, Fe-catalyzed reactions of β-amidoalkylsilyl peroxides 34 with various coupling partners afforded none of desired coupling products. However, the Cu-catalyzed reaction of 34 with Me_3_SiCN as coupling partner gave the corresponding coupling product 35 in 88% yield.

**Fig. 6:**
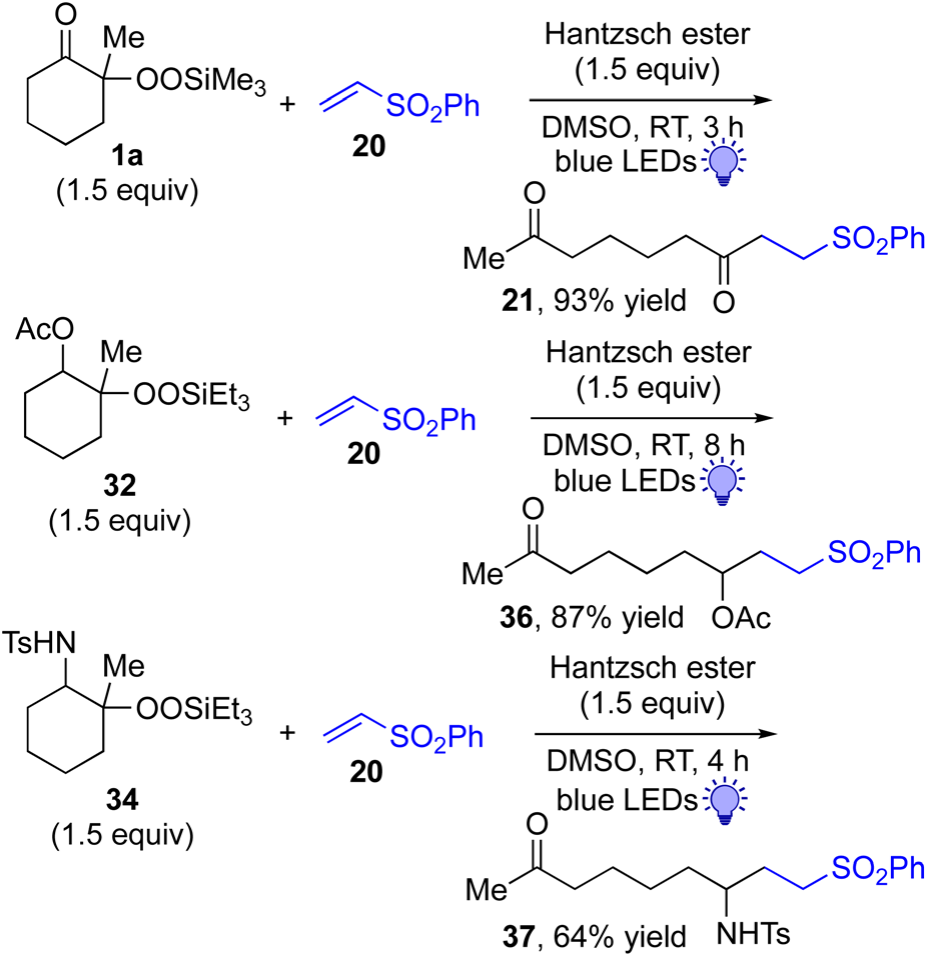
Visible light-promoted reaction of α-keto, β-acetoxy, and β-amidoalkylsilyl peroxides.

This approach is also applicable to our recently developed visible-light-promoted alkylation of electron-deficient alkenes with alkylsilyl peroxides.^18^ Treatment of α-ketoalkylsilyl peroxide 1a with phenyl vinyl sulfone (20) and an equimolar amount of Hantzsch ester in DMSO under blue light irradiation at room temperature for 3 h afforded the desired conjugate addition product 21 in 93% yield (Fig. 6). This approach can be further expanded to the visible-light-promoted alkylation of other functionalized alkylsilyl peroxides. For example, the reaction of β-acetoxy- and β-amidoalkylsilyl peroxides 32 and 34 (1.5 equiv.) with phenyl vinyl sulfone (20) and Hantzsch ester (1.5 equiv.) in DMSO under blue light irradiation at room temperature for 4–8 h gave rise to conjugate adducts 36 and 37, respectively, in 87% and 64% yields (Fig. 6).^19^

## Conclusions

In summary, we have developed an Fe-catalyzed and visible-light-promoted radical transformations for functionalized alkylsilyl peroxides, such as α-keto-, β-acetoxy-, and β-amidoalkylsilyl peroxides with several coupling partners under mild conditions and with a broad substrate scope. The synthetic utility of our approach is demonstrated by the facile generation of reactive acyl chloride intermediates, which can be easily transformed to the corresponding amides, esters, and phenyl ketones. A mechanistic study suggested the participation of intermediary acyl, α-acetoxyalkyl, and α-amidoalkyl radical species in the radical-promoted coupling reactions.

## Data availability

The datasets supporting this article have been uploaded as part of the ESI.†

## Author contributions

K. M. conceptualized the research. J. L. and S. L. performed the experiments. T. K. and K. M. prepared the manuscript and the ESI.† Z. W. and Y. L. edited the ESI.† K. M. supervised the project and edited the manuscript.

## Conflicts of interest

There are no conflicts to declare.

## Supplementary Material

SC-015-D3SC06857A-s001
