# Supplementary material for: Synthetic utility of functionalized alkylsilyl peroxides for Fe-catalyzed and visible-light-promoted radical transformation
Source: Chem Sci. 2024 Feb 22;15(13):4757–62. doi: 10.1039/d3sc06857a (PMC10966965; doi:10.1039/d3sc06857a)
Supplement: SC-015-D3SC06857A-s001 [file SC-015-D3SC06857A-s001.pdf]

Electronic Supplementary Material (ESI) for Chemical Science.
This journal is © The Royal Society of Chemistry 2024

Supporting Information

Synthetic Utility of Functionalized Alkylsilyl Peroxides for Fe-Catalyzed and Visible-Light-Promoted Radical Transformation

Jiahao Liu,[ab] Shiyong Liu,[ab] Zhe Wang,[ab] Terumasa Kato,*[abc] Yan Liu,*[ab]

and Keiji Maruoka*[abc]

maruoka.keiji.4w@kyoto-u.ac.jp

[a] *School of Chemical Engineering and Light Industry, Guangdong University of Technology, Guangzhou, 510006, China*

[b] *Guangdong Provincial Key Laboratory of Plant Resources Biorefinery, Guangdong University of Technology, Guangzhou, 510006, China*

[c] *Laboratory of Organocatalytic Chemistry, Graduate School of Pharmaceutical Sciences, Kyoto University, Sakyo, Kyoto, 606-8501, Japan*

Table of Contents

1. General information ··· S2

2. Reaction optimization ··· S3–S6

3. Synthesis and characterization of alkylsilyl peroxides ··· S7–S25

4. Procedures for the transformation of alkylsilyl peroxides ··· S26–S43

5. References ··· S44–S45

6. ^{1}H NMR and ^{13}C NMR Spectra ··· S46–S102

1. General information

^{1}H NMR and ^{13}C NMR spectra were recorded on Bruker AVANCE III 400 MHz spectrometer (400 MHz for ^{1}H NMR, 101 MHz for ^{13}C NMR) and JEOL JNM-ECZ500R/S1 500 MHz spectrometer (500 MHz for ^{1}H NMR, 126 MHz for ^{13}C NMR). Data were reported as follows: chemical shift, integration, multiplicity (s = singlet, d = doublet, t = triplet, q = quartet, m = multiplet, br = broad), coupling constants (Hz), and assignment. Tetramethylsilane (TMS) was used as the internal standard (0 ppm) for the ^{1}H NMR spectra. $CDCl_3$ (77.16 ppm) and CD_3OD (49.00 ppm) was used as the internal standard for the ^{13}C NMR spectra. High-resolution mass spectra (HRMS) were recorded on a ThermoFisher Q-Exactive Orbitrap spectrometer with electrospray ionization (ESI) sources. Melting points were obtained on a JIAHANG JH30 melting-point apparatus. Infrared (IR) spectra were obtained on a Thermo-Filsher Nicolet 6700 spectrometer. Optical rotations were recorded on Anton Paar MCP 500 Polarimeter. Unless otherwise noted, all reactions were performed under argon atmosphere. Reactions were monitored by thin-layer chromatography (TLC, silica gel HSGF 254, 0.25±0.02 mm). Reaction products were purified by column chromatography on silica gel (Qingdao Haiyang Chemical, zcx-II, 300–400 mesh). Dry solvents, such as 1,4-dioxane (Dioxane), tetrahydrofuran (THF), benzene, acetonitrile (MeCN), and dichloromethane (DCM) were purchased from Innochem and Energy Chemical as "Dehydrated". Compounds **7**,[1] **10**[2] and **12**[3] were synthesized according to literatures. Hydrogen peroxide (H_2O_2) in *tert*-butyl methyl ether (MTBE) solution was prepared according to the literature.[4] All other reagents and solvents were purchased from reagent companies and used as received.

2. Reaction optimization

Table S1. The effect of catalysts and ligands[a]

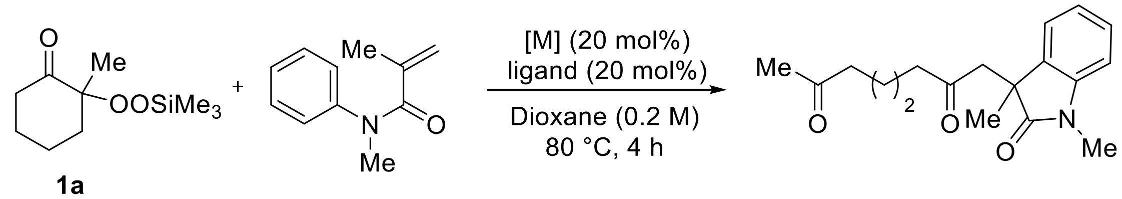

1a (1.2 equiv) **7** **8a**

Entry	[M]	Ligand	Yield (%)[b]
1	CuI	**L1**	11
2	$Cu(MeCN)_4BF_4$	**L1**	19
3	$Ni(OAc)_2 \cdot 4H_2O$	**L1**	18
4	$FeCl_2$	**L1**	42
5	$Fe(acac)_2$	**L1**	53
6	$FeCl_3$	**L1**	28
7	$Fe(acac)_3$	**L1**	59
8	$Fe(acac)_3$	-	40
9	$Fe(acac)_3$	**L2**	44
10	$Fe(acac)_3$	**L3**	49
11	$Fe(acac)_3$	**L4**	48
12	$Fe(acac)_3$	**L5**	44
13	$Fe(acac)_3$	**L6**	45
14	$Fe(acac)_3$	**L7**	38
15	$Fe(acac)_3$	**L8**	39

[a] The reactions were carried out in the presence of **1a** (0.24 mmol), **7** (0.20 mmol), metal catalyst (0.04 mmol), ligand (0.04 mmol) in Dioxane (1.0 mL) at 80 °C for 4 h. [b]The yield was determined by ^{1}H NMR spectroscopy using nitromethane as an internal standard.

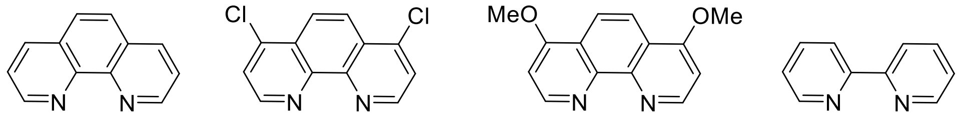

L1 **L2** **L3** **L4**

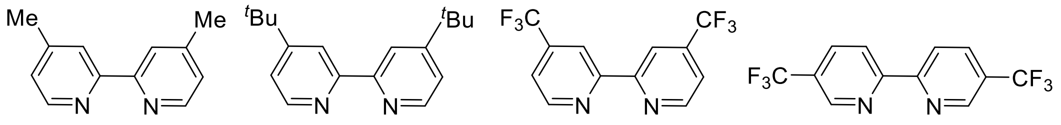

L5 **L6** **L7** **L8**

Table S2. The effect of solvents, temperature, molar ratio and ligand[a]

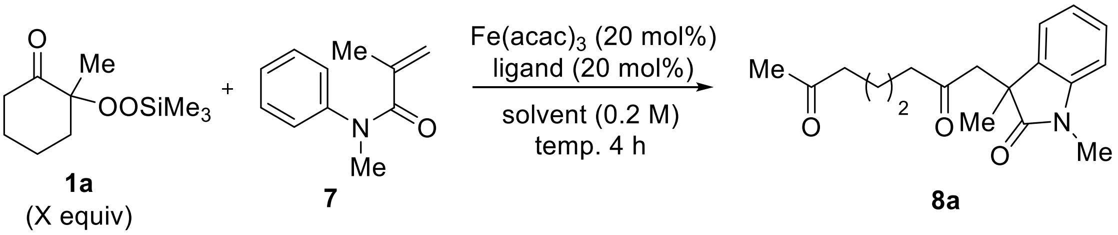

Entry	**1a** (equiv)	Ligand	Solvent	Temperature (°C)	Yield (%)[b]
1	1.2	1,10-Phen	Dioxane	80	59
2	1.2	1,10-Phen	MeCN	80	50
3	1.2	1,10-Phen	DCE	80	42
4	1.2	1,10-Phen	Benzene	80	50
5	1.2	1,10-Phen	DMSO	80	65
6	1.2	1,10-Phen	DMSO	40	69
7	1.2	1,10-Phen	DMSO	25	38
8	1.5	1,10-Phen	DMSO	40	81
9	2.0	1,10-Phen	DMSO	40	91 (95)[e]
10	2.0	-	DMSO	40	16
11[c]	2.0	-	DMSO	40	38
12[d]	2.0	-	DMSO	40	75

[a]The reactions were carried out in the presence of **1a** (0.24~0.40 mmol), **7** (0.20 mmol), $Fe(acac)_3$ (0.04 mmol) with or without 1,10-Phen (0~0.04 mmol) in solvent (1.0 mL) at indicated temperature for 4 h. [b]The yield was determined by ^{1}H NMR spectroscopy using nitromethane as an internal standard. [c]For 8 h. [d]For 12 h. [e]Isolated yield.

Table S3. Reaction optimization for the decarboxylative coupling reaction with cinnamic acid[a]

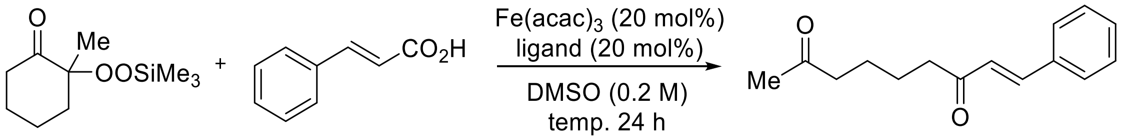

1a **14** **15**

Entry	**1a** (equiv)	**14** (equiv)	Ligand	Temperature (°C)	Yield (%)[b]
1	2.0	1.0	1,10-Phen	80	25
2	2.0	1.0	-	80	24
3[c]	2.0	1.0	1,10-Phen	40	19
4	2.0	1.0	1,10-Phen	40	21
5	1.0	3.0	1,10-Phen	40	41
6	1.0	5.0	1,10-Phen	40	53
7	1.0	5.0	-	80	59 (56)[d]

[a]The reactions were carried out in the presence of **1a** and **14** (1.0 equiv = 0.20 mmol), $Fe(acac)_3$ (0.04 mmol) with or without 1,10-Phen (0~0.04 mmol) in DMSO (1.0 mL) at 40~80 °C for 24 h. [b]The yield was determined by ^1H NMR spectroscopy using nitromethane as an internal standard. [c]For 4 h. [d]Isolated yield.

Table S4. Reaction optimization for the coupling reaction with 1,1-diphenylethylene[a]

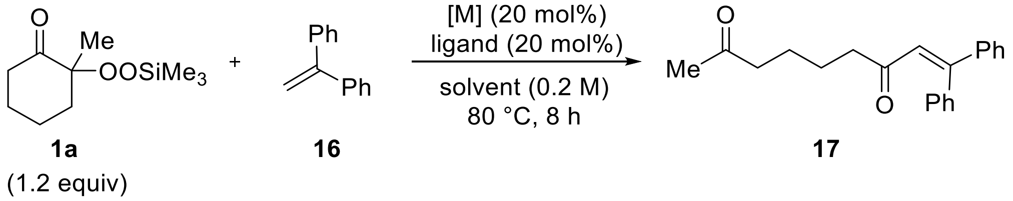

Entry	1a (equiv)	[M]	Ligand	Solvent	Yield (%)[b]
1	1.2	CuBr	1,10-Phen	Dioxane	23
2	1.2	CuBr	-	Dioxane	22
3	1.2	$FeCl_3$	-	Dioxane	<5
4	1.2	$Fe(acac)_3$	-	Dioxane	<5
5	1.2	$FeCl_2$	-	Dioxane	21
6	1.2	$Fe(acac)_2$	-	Dioxane	6
7	1.2	$Fe(OTf)_2$	-	Dioxane	15
8	1.2	$FeSO_4 \cdot 7H_2O$	-	Dioxane	30
9	1.2	$FeSO_4 \cdot 7H_2O$	-	DCE	<5
10	1.2	$FeSO_4 \cdot 7H_2O$	-	MeCN	7
11	1.2	$FeSO_4 \cdot 7H_2O$	-	DMSO	29
12	1.2	$FeSO_4 \cdot 7H_2O$	-	DMF	59
13	1.2	$FeSO_4 \cdot 7H_2O$	1,10-Phen	DMF	46
14	2.0	$FeSO_4 \cdot 7H_2O$	1,10-Phen	DMF	43
15[c]	2.0	$FeSO_4 \cdot 7H_2O$	-	DMF	52
16[c,e]	2.0	$FeSO_4 \cdot 7H_2O$	1,10-Phen	DMF	37
17[c,d]	2.0	$Fe(acac)_3$	1,10-Phen	DMSO	<5
18[f]	1.0	$FeSO_4 \cdot 7H_2O$	-	DMF	73 (66)[g]

[a]The reactions were carried out in the presence of **1a** (0.24~0.40 mmol) and **16** (0.20 mmol), catalyst (0.04 mmol), ligand (0~0.04 mmol) in solvent (1.0 mL) at 80 °C for 8h. [b]The yield was determined by ^1H NMR spectroscopy using nitromethane as an internal standard. [c]At 40 °C. [d]For 4 h. [e]For 24 h. [f]**16** (3.0 equiv). [g]Isolated yield.

3. Synthesis and characterization of alkylsilyl peroxides

[A] Synthesis of α-ketoalkylsilyl peroxides

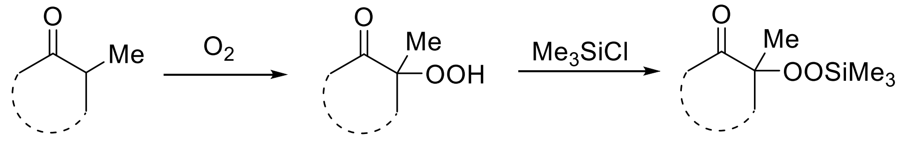

38 **1**

I. Synthesis of α-keto hydroperoxides

(1) General procedure of the synthesis of α-keto hydroperoxide[5, 6]

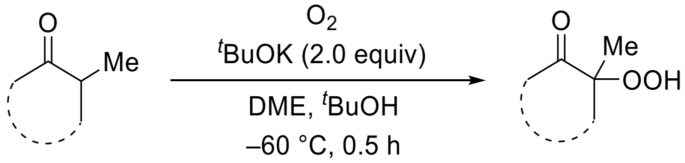

38

To a solution of potassium *tert*-butoxide (^{t}BuOK, 4.04 g, 36 mmol, 2.0 equiv) in *tert*-butyl alcohol (^{t}BuOH, 40 mL) and 1,2-dimethoxyethane (DME, 60 mL) was added ketone substrate (18 mmol) at –60 °C. The oxygen gas (15 L × 3) was bubbled into the stirred solution at same temperature for a period of 0.5 h. After completion of bubbling, the reaction mixture was neutralized with solution of H_3PO_4 (85%, 3.0 mL) in water (18 mL) at –60 °C. The mixture was poured into ice cold water (200 mL) and exacted with DCM (100 mL × 3). The combined organic phase was washed with water (100 mL × 3) and then brine (100 mL). This was dried over Na_2SO_4, filtrated and concentrated under reduce pressure. The obtained crude product was purified by column chromatography on silica gel (eluted with petroleum ether/ethyl acetate = 15/1 to 10/1, gradient) to give the corresponding α-keto hydroperoxide **38**.

2-Hydroperoxy-2-methylcyclohexan-1-one (38a) [5, 6]

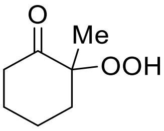

Colorless oil, 43% isolated yield (1.12 g, 7.7 mmol). **^{1}H NMR (400 MHz, Chloroform-*d*)**: δ 9.44 (s, 1H), 2.66–2.57 (m, 1H), 2.39–2.27 (m, 1H), 2.11–2.00 (m, 1H), 1.92–1.75 (m, 3H), 1.74–1.66 (m, 1H), 1.70–1.52 (m, 1H), 1.34 (s, 3H); **^{13}C NMR (101 MHz, Chloroform-*d*)**: δ 213.1, 87.9, 39.7, 37.3, 27.5, 22.3, 20.0; **IR (neat)**: 2941, 1702, 1409, 1361, 1175, 1083, 881, 726, 594, 437 cm^{-1}; **HRMS (ESI)** m/z: $[M+Na]^+$ Calcd for $C_7H_{12}NaO_3^+$ 167.0679; Found 167.0679.

2-Hydroperoxy-2-methylcyclopentan-1-one (38b)

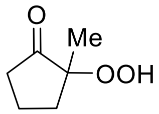

Colorless oil, 25% isolated yield (0.59 g, 4.5 mmol). **^{1}H NMR (400 MHz, Chloroform-*d*)**: δ 8.91 (s, 1H), 2.47–2.37 (m, 1H), 2.37–2.27 (m, 2H), 2.13–1.99 (m, 1H), 1.98–1.86 (m, 1H), 1.86–1.71 (m, 1H), 1.27 (s, 3H); **^{13}C NMR (101 MHz, Chloroform-*d*)**: δ 218.1, 87.9, 36.1, 32.7, 18.7, 17.4; **IR (neat)**: 3325, 2978, 1738, 1471, 1440, 1389, 1370, 1303, 1193, 1171, 1076, 1045, 933, 860, 813, 691, 621, 594, 538 cm^{-1}; **HRMS (ESI)** m/z: $[M+Na]^+$ Calcd for $C_6H_{10}NaO_3^+$ 153.0522; Found 153.0518.

2-Hydroperoxy-2-methylcycloheptan-1-one (38c)

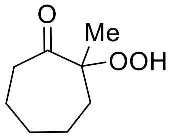

Colorless oil, 60% isolated yield (1.71 g, 10.8 mmol). **^{1}H NMR (400 MHz, Chloroform-*d*)**: δ 9.36 (s, 1H), 2.65–2.49 (m, 2H), 1.98 (ddd, *J* = 15.0, 8.2, 1.7 Hz, 1H), 1.93–1.80 (m, 1H), 1.83–1.71 (m, 1H), 1.74–1.60 (m, 3H), 1.60–1.48 (m, 1H), 1.47 (s, 3H), 1.46–1.34 (m, 1H); **^{13}C NMR (101 MHz, Chloroform-*d*)**: δ 215.2, 89.9, 39.8, 36.2, 29.2, 25.6, 24.4, 20.8; **IR (neat)**: 3385, 2936, 2861, 1702, 1451, 1372, 1324, 1196, 1169, 1110, 1070, 1051, 943, 519, 470 cm^{-1}; **HRMS (ESI)** m/z: $[M+Na]^+$ Calcd for $C_8H_{14}NaO_3^+$ 181.0835; Found 181.0833.

2-Hydroperoxy-2-methylcyclooctan-1-one (38d)

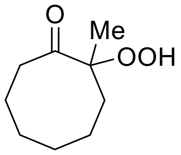

Prepared from 2-methylcyclooctan-1-one (0.98g, 7.0 mmol). Light yellow oil, 40% isolated yield (0.48 g, 3.4 mmol). **^{1}H NMR (400 MHz, Chloroform-*d*)**: δ 9.45 (s, 1H), 2.65–2.42 (m, 2H), 2.19–2.04 (m, 1H), 2.01–1.92 (m, 1H), 1.91–1.80 (m, 2H), 1.79–1.58 (m, 2H), 1.52–1.22 (m, 7H); **^{13}C NMR (101 MHz, Chloroform-*d*)**: δ 218.8, 88.5, 37.3, 34.5, 29.0, 26.0, 24.9, 22.7, 20.1; **IR (neat)**: 3385, 2934, 2860, 1701, 1448, 1373, 1333, 1242, 1160, 1123, 1082, 1028 cm^{-1}; **HRMS (ESI)** m/z: $[M+Na]^+$ Calcd for $C_9H_{16}NaO_3^+$ 195.0992; Found 195.0990.

2-Hydroperoxy-2-methyl-3,4-dihydronaphthalen-1(2*H*)-one (38e)[7, 8]

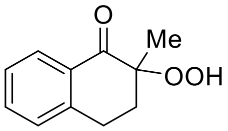

Prepared from 2-methyl-3,4-dihydronaphthalen-1(2*H*)-one (2.40 g, 15 mmol). Yellow oil, 61% isolated yield (1.71 g, 8.9 mmol). **^{1}H NMR (400 MHz, Chloroform-*d*)**: δ 9.59 (s, 1H), 8.03 (d, *J* = 7.9 Hz, 1H), 7.52 (t, *J* = 7.5 Hz, 1H), 7.33 (t, *J* = 7.5 Hz, 1H), 7.26 (d, *J* = 7.7 Hz, 1H), 3.15–3.08 (m, 2H), 2.75–2.58 (m, 1H), 2.16–2.05 (m, 1H), 1.47 (s, 3H). **^{13}C NMR (101 MHz, Chloroform-*d*)**: δ 199.8, 143.0, 134.2, 131.2, 128.9, 128.0, 127.0, 85.1, 32.3, 26.7, 18.5; **IR (neat)**: 3371, 2936, 1682, 1600, 1455, 1370, 1309, 1224, 1182, 1151, 1093, 977, 915, 843, 795, 785, 737, 678, 573, 531, 504, 484, 449 cm^{-1}; **HRMS (ESI)** m/z: $[M+Na]^+$ Calcd for $C_{11}H_{12}NaO_3^+$ 215.0679; Found 215.0678.

2-Hydroperoxy-2-methylpentan-3-one (38f)

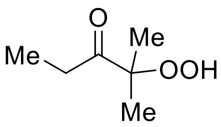

Light yellow oil, 29% isolated yield (0.69 g, 5.2 mmol). **^{1}H NMR (400 MHz, Chloroform-*d*)**: δ 8.71 (s, 1H), 2.66 (q, J = 7.3 Hz, 2H), 1.37 (s, 6H), 1.07 (t, J = 7.3 Hz, 3H); **^{13}C NMR (101 MHz, Chloroform-*d*)**: δ 214.0, 88.1, 29.7, 21.8, 7.8; **IR (neat)**: 3394, 2985, 2941, 2883, 1709, 1461, 1377, 1363, 1212, 1168, 1102, 1043 cm^{-1}; **HRMS (ESI)** m/z: $[M+Na]^+$ Calcd for $C_6H_{12}NaO_3^+$ 155.0679; Found 155.0675.

2-Hydroperoxy-2,4-dimethylpentan-3-one (38g)[5,6]

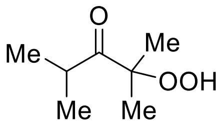

Prepared from 2,4-dimethylpentan-3-one (2.28 g, 20 mmol). Colorless oil, 44% isolated yield (1.28 g, 8.8 mmol). **^{1}H NMR (400 MHz, Chloroform-*d*)**: δ 8.86 (s, 1H), 3.27–3.14 (m, 1H), 1.40 (s, 6H), 1.10 (d, J = 6.8 Hz, 6H); **^{13}C NMR (101 MHz, Chloroform-*d*)**: δ 218.0, 88.2, 34.2, 21.5, 19.9.

2-Hydroperoxy-2,6-dimethylcyclohexan-1-one (38h)

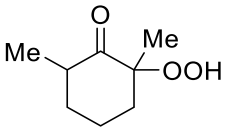

Colorless oil, 29% isolated yield (0.82 g, 5.2 mmol) as a 58/42 diastereomeric mixture. **^{1}H NMR (400 MHz, Chloroform-*d*)**: δ 9.63 (s, 0.39H, *minor*), 8.41 (s, 0.53H, *major*), 3.18–3.09 (m, 0.58H, *major*), 2.62–2.52 (m, 0.41H, *minor*), 2.13–2.03 (m, 2H), 2.00–1.91 (m, 0.60H, *major*), 1.90–1.74 (m, 1.28H, *minor*), 1.59–1.53 (m, 0.54H, *major*), 1.53–1.49 (m, 0.57H, *major*), 1.47 (s, 1.28H, *minor*), 1.40–1.35 (m, 0.42H, *minor*), 1.33 (s, 1.79H, *major*), 1.31–1.25 (m, 0.62H, *major*), 1.04–1.01 (m, 3H); **^{13}C NMR (101 MHz, Chloroform-*d*)**: δ 215.4, 212.8, 88.2, 87.4, 43.0, 41.3, 39.3, 37.3, 37.0, 36.1, 22.2, 20.9, 20.5, 18.8, 14.5, 14.2; **IR (neat)**: 3400, 2936, 2871, 1713, 1456, 1374, 1316, 1238, 1157, 1129, 1096, 996, 980, 954, 869, 858, 574, 505, 475 cm^{-1}; **HRMS (ESI)** m/z: $[M+Na]^+$ Calcd for $C_8H_{14}NaO_3^+$ 181.0835; Found 181.0833.

2-Ethyl-2-hydroperoxycyclohexan-1-one (38k)

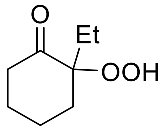

Colorless oil, 56% isolated yield (1.60 g, 10.1 mmol). **^{1}H NMR (400 MHz, Chloroform-d)**: δ 9.17 (s, 1H), 2.56–2.48 (m, 1H), 2.43–2.34 (m, 1H), 2.11–2.00 (m, 2H), 2.00–1.93 (m, 1H), 1.92–1.83 (m, 2H), 1.74–1.63 (m, 3H), 0.89 (t, *J* = 7.5 Hz, 3H); **^{13}C NMR (101 MHz, Chloroform-d)**: δ 213.4, 90.2, 40.2, 33.7, 27.2, 25.4, 22.2, 6.9; **IR (neat)**: 3366, 2942, 2867, 1711, 1460, 1433, 1380, 1310, 1257, 1123, 1087, 959, 564 cm^{-1}. **HRMS:** *m/z*: [M-H]$^-$ Calcd for $C_8H_{13}O_3^-$ 157.0870; Found 157.0862.

(2) Procedure for the synthesis of α-ketohydroperoxide from menthone

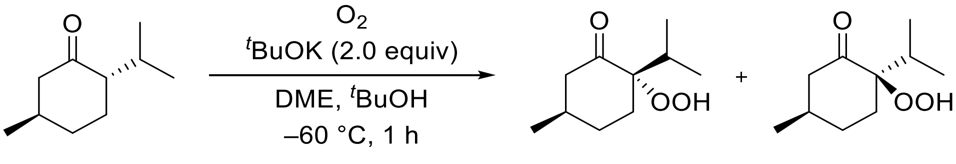

38i **38j**

To a solution of ^{t}BuOK (8.08 g, 72 mmol, 2.0 equiv) in ^{t}BuOH (80 mL) and DME (120 mL) was added L-menthone (5.55 g, 36 mmol) at –60 °C. The oxygen gas (15 L × 6) was bubbled into the stirred solution at same temperature for a period of 1 h. After completion of bubbling, the reaction mixture was neutralized with the solution of H_3PO_4 (85%, 6.0 mL) in water (36 mL) at –60 °C. The mixture was poured into ice cold water (200 mL) and exacted with DCM (100 mL × 3). The combined organic phase was washed with water (100 mL × 3) and then brine (100 mL). This was dried over Na_2SO_4, filtrated and concentrated under reduce pressure. The residue was purified by column chromatography on silica gel (eluted with petroleum ether/ethyl acetate = 50/1 to 10/1, gradient) to give the products **38i** and **38j**.

The stereochemistry of **38i** and **38j** were determined after the transformation to the corresponding alcohols **39i** and **39j**, respectively.

(2*S*, 5*R*)-2-Hydroperoxy-2-isopropyl-5-methylcyclohexan-1-one (38i)

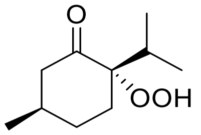

Colorless oil, 24% isolated yield (1.64 g, 8.6 mmol). $[\alpha]_D^{25}$ = +20.7 (c 1.0, $CHCl_3$). **^{1}H NMR (400 MHz, Chloroform-*d*)**: δ 9.21 (s, 1H), 2.39 (ddd, *J* = 13.4, 4.1, 2.4 Hz, 1H), 2.34 – 2.22 (m, 1H), 2.24–2.00 (m, 3H), 1.97–1.74 (m, 1H), 1.50–1.33 (m, 1H), 1.01 (d, *J* = 6.4 Hz, 3H), 0.98 (d, *J* = 6.9 Hz, 3H), 0.84 (d, *J* = 7.0 Hz, 3H); **^{13}C NMR (101 MHz, Chloroform-*d*)**: δ 213.1, 91.7, 48.7, 35.0, 30.9, 30.7, 30.4, 21.9, 16.8, 15.3; **IR (neat)**: 3424, 2959, 2875, 1708, 1459, 1426, 1371, 1273, 1140, 1119, 1097, 1040, 1015, 979, 705, 598, 557, 518, 474, 441 cm^{-1}; **HRMS (ESI)** m/z: $[M+Na]^+$ Calcd for $C_{10}H_{18}NaO_3^+$ 209.1148; Found 209.1146.

(2*R*, 5*R*)-2-Hydroperoxy-2-isopropyl-5-methylcyclohexan-1-one (38j)

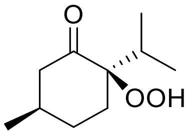

Colorless oil, 7% isolated yield (0.49 g, 2.5 mmol). $[\alpha]_D^{25}$ = –11.0 (c 1.0, $CHCl_3$). **^{1}H NMR (400 MHz, Chloroform-*d*)**: δ 8.63 (s, 1H), 2.53–2.44 (m, 1H), 2.43 (d, *J* = 6.6 Hz, 2H), 2.15 (ddd, *J* = 15.1, 7.9, 4.4 Hz, 1H), 2.10–2.01 (m, 1H), 1.83–1.68 (m, 2H), 1.65–1.52 (m, 1H), 0.99 (d, *J* = 6.8 Hz, 3H), 0.94 (d, *J* = 6.9 Hz, 3H), 0.89 (d, *J* = 7.0 Hz, 3H); **^{13}C NMR (101 MHz, Chloroform-*d*)**: δ 211.5, 90.4, 47.4, 33.5, 28.6, 28.5, 27.7, 20.6, 17.1, 16.5; **IR (neat)**: 3388, 2958, 2876, 1713, 1456, 1433, 1386, 1335, 1314, 1276, 1252, 1219, 1121, 1094, 1001, 978, 934, 854, 688, 601, 568, 524, 467 cm^{-1}; **HRMS (ESI)** m/z: $[M+Na]^+$ Calcd for $C_{10}H_{18}NaO_3^+$ 209.1148; Found 209.1145.

***Transformation of alkyl hydroperoxide to alcohol**[9]

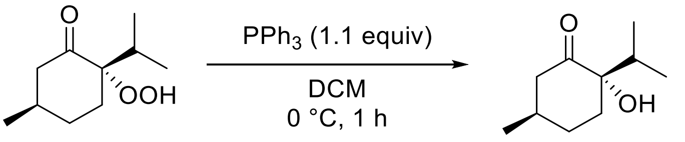

38i **39i**

To a solution of **38i** (350 mg, 1.9 mmol) in DCM (10 mL) was added triphenylphosphine (543 mg, 2.1 mmol, 1.1 equiv) at 0 °C. After stirring the mixture for 1 h, the resulting mixture was concentrated under reduced pressure. The residue was purified by column chromatography on silica gel (eluted with petroleum ether/ethyl acetate = 50/1 to 25/1, gradient) to give the alcohol **39i**. The stereochemistry of **39i** was determined to be (2*S*,5*R*) by optical rotation according to the literature.[10, 11]

The stereochemistry of alcohol **39j** was determined to be (2*R*,5*R*) by the same manner as described above using **38j**.[10, 11]

(2*S*, 5*R*)-2-Hydroxy-2-isopropyl-5-methylcyclohexan-1-one (39i)[11, 12]

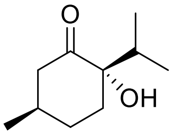

Colorless oil. 47% isolated yield (151 mg, 0.89 mmol). $[\alpha]_D^{25}$ = +127.3 (c 1.0, $CHCl_3$).
^{1}H NMR (400 MHz, Chloroform-*d*): δ 3.72 (s, 1H), 2.40 (ddd, *J* = 13.1, 4.3, 2.2 Hz, 1H), 2.30–2.25 (m, 1H), 2.21–2.10 (m, 2H), 1.91–1.75 (m, 1H), 1.72–1.61 (m, 1H), 1.45–1.30 (m, 2H), 1.01 (d, *J* = 6.5 Hz, 3H), 0.94 (d, *J* = 6.7 Hz, 3H), 0.64 (d, *J* = 6.8 Hz, 3H); **^{13}C NMR (101 MHz, Chloroform-*d*)**: δ 214.5, 80.3, 46.1, 37.1, 36.2, 30.9, 30.7, 22.3, 16.0, 15.3.

(2*R*, 5*R*)-2-Hydroxy-2-isopropyl-5-methylcyclohexan-1-one (39j)[11, 12]

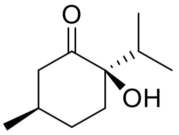

Colorless oil. 43% isolated yield (138 mg, 0.81 mmol). $[\alpha]_D^{25}$ = –124.6 (c 1.0, $CHCl_3$). **^{1}H NMR (400 MHz, Chloroform-*d*)**: δ 3.72 (s, 1H), 2.65 (dd, *J* = 13.2, 6.4 Hz, 1H), 2.47–2.35 (m, 1H), 2.21–2.08 (m, 3H), 1.89 (tt, *J* = 14.0, 4.3 Hz, 1H), 1.59 (td, *J* = 14.0, 4.1 Hz, 1H), 1.48–1.40 (m, 1H), 0.93 (d, *J* = 6.7 Hz, 3H), 0.88 (d, *J* = 7.2 Hz, 3H), 0.63 (d, *J* = 6.8 Hz, 3H); **^{13}C NMR (101 MHz, Chloroform-*d*)**: δ 214.9, 80.7, 44.4, 33.0, 32.3, 30.9, 27.9, 18.8, 16.1, 15.5.

II. Synthesis of α-ketoalkylsilyl peroxide

General procedure of the synthesis of α-ketoalkylsilyl peroxide

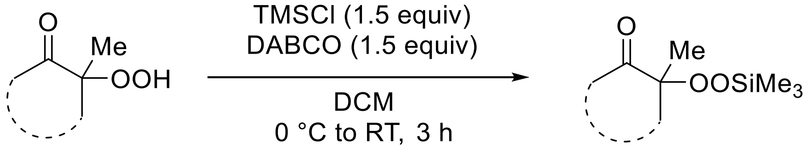

38 **1**

To a solution of α-keto hydroperoxide **38** (0.50 mmol) and 1,4-diazabicyclo[2.2.2]octane (DABCO, 84 mg, 0.75 mmol, 1.5 equiv) in dry DCM (2.5 mL) was added trimethylsilyl chloride (TMSCl, 81 mg, 0.75 mmol, 1.5 equiv) at 0 °C. After the stirring at room temperature for 3 h, the reaction mixture was diluted with DCM (15 mL) and washed with water (10 mL × 3) and then brine (10 mL). The organic layer was dried over Na_2SO_4, filtrated and concentrated under reduced pressure. The residue was purified by short column chromatography on silica gel (eluted with ice-cold DCM) to give the corresponding α-ketoalkylsilyl peroxide **1**.

2-Methyl-2-((trimethylsilyl)peroxy)cyclohexan-1-one (1a)[6]

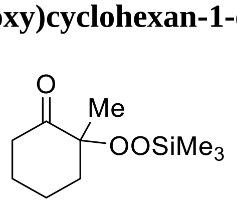

Prepared from **36a** (2.08 g, 14.4 mmol). Colorless oil, 42% isolated yield (1.31 g, 6.1 mmol). **^{1}H NMR (400 MHz, Chloroform-*d*)**: δ 2.94 (td, *J* = 13.5, 13.0, 5.9 Hz, 1H), 2.30 – 2.20 (m, 1H), 2.13–2.02 (m, 2H), 1.97–1.82 (m, 1H), 1.68–1.45 (m, 3H), 1.27

(s, 3H), 0.18 (s, 9H). **^{13}C NMR (101 MHz, Chloroform-*d*)**: δ 211.3, 86.9, 39.2, 38.9, 28.4, 20.9, 19.2, –1.1; **IR (neat)**: 2937, 2865, 1729, 1449, 1433, 1372, 1310, 1251, 1165, 1123, 1085, 1025, 978, 904, 881, 848, 806, 775, 752, 739, 692, 520 cm^{-1}; **HRMS (ESI)** m/z: $[M+Na]^+$ Calcd for $C_{10}H_{20}NaO_3Si^+$ 239.1074; Found 239.1072.

2-Methyl-2-((trimethylsilyl)peroxy)cyclopentan-1-one (1b)

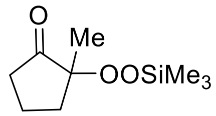

Colorless oil, 50% isolated yield (50.8 mg, 0.25 mmol). **^{1}H NMR (400 MHz, Chloroform-*d*)**: δ 2.44 (dt, *J* = 13.2, 7.6 Hz, 1H), 2.39–2.15 (m, 2H), 2.11–1.96 (m, 1H), 1.87–1.74 (m, 1H), 1.75–1.68 (m, 1H), 1.22 (s, 3H), 0.16 (s, 9H); **^{13}C NMR (101 MHz, Chloroform-*d*)**: δ 215.3, 86.8, 36.7, 33.5, 18.7, 17.8, –1.2; **IR (neat)**: 2962, 2926, 2856, 1716, 1662, 1494, 1448, 1413, 1364, 1261, 1186, 1092, 1021, 970, 863, 801, 703, 583, 530 cm$^{–1}$; **HRMS (ESI)** m/z: $[M+Na]^+$ Calcd for $C_9H_{18}NaO_3Si^+$ 225.0917; Found 225.0909.

2-Methyl-2-((trimethylsilyl)peroxy)cycloheptan-1-one (1c)

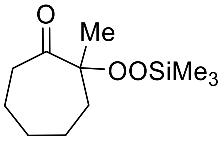

Light yellow oil, 71% isolated yield (81.5 mg, 0.35 mmol). **^{1}H NMR (400 MHz, Chloroform-*d*)**: δ 3.11–2.95 (m, 1H), 2.36–2.19 (m, 1H), 1.96–1.81 (m, 1H), 1.85–1.74 (m, 1H), 1.75–1.65 (m, 2H), 1.63–1.49 (m, 2H), 1.46–1.34 (m, 2H), 1.35 (s, 3H), 0.18 (s, 9H); **^{13}C NMR (101 MHz, Chloroform-*d*)**: δ 213.2, 90.2, 39.8, 36.8, 28.4, 24.5, 24.5, 21.3, –1.1; **IR (neat)**: 2936, 2861, 1711, 1448, 1369, 1251, 1168, 1112, 1070, 942, 876, 848, 788, 772, 752, 737 cm$^{–1}$; **HRMS (ESI)** m/z: $[M+Na]^+$ Calcd for $C_{11}H_{22}NaO_3Si^+$ 253.1230; Found 253.1226.

2-Methyl-2-((trimethylsilyl)peroxy)cyclooctan-1-one (1d)

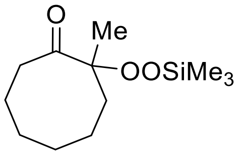

Light yellow oil, 56% isolated yield (68.2 mg, 0.28 mmol). **^{1}H NMR (400 MHz, Chloroform-*d*)**: δ 3.18 (ddd, *J* = 12.8, 11.5, 3.6 Hz, 1H), 2.15–2.00 (m, 2H), 2.03–1.89 (m, 1H), 1.85–1.67 (m, 1H), 1.71–1.59 (m, 4H), 1.56–1.49 (m, 1H), 1.42–1.36 (m, 1H), 1.35 (s, 3H), 1.10–0.95 (m, 1H), 0.17 (s, 9H); **^{13}C NMR (101 MHz, Chloroform-*d*)**: δ 217.3, 90.0, 38.1, 36.8, 29.0, 28.0, 26.6, 23.5, 20.5, 0.0; **IR (neat)**: 2934, 2860, 1719, 1470, 1448, 1371, 1251, 1081, 848, 752, 730 cm^{-1}; **HRMS (ESI)** m/z: [M+Na]$^+$ Calcd for $C_{12}H_{24}NaO_3Si^+$ 267.1387; Found 267.1383.

2-Methyl-2-((trimethylsilyl)peroxy)-3,4-dihydronaphthalen-1(2H)-one (1e)

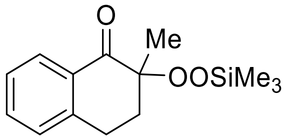

Yellow oil, 68% isolated yield (89.7 mg, 0.34 mmol). **^{1}H NMR (400 MHz, Chloroform-*d*)**: δ 8.04 (d, *J* = 7.2 Hz, 1H), 7.46 (t, *J* = 7.5 Hz, 1H), 7.30 (t, *J* = 7.5 Hz, 1H), 7.22 (d, *J* = 7.7 Hz, 1H), 3.27–3.07 (m, 1H), 2.86 (dt, *J* = 17.0, 5.8 Hz, 1H), 2.67–2.52 (m, 1H), 2.16–1.99 (m, 1H), 1.46 (s, 3H), 0.07 (s, 9H); **^{13}C NMR (101 MHz, Chloroform-*d*)**: δ 195.3, 143.5, 133.4, 132.1, 128.6, 128.4, 126.8, 83.7, 33.8, 25.9, 19.6, –1.2; **IR (neat)**: 2961, 2936, 1701, 1603, 1456, 1371, 1310, 1251, 1234, 1199, 1153, 1093, 967, 904, 875, 848, 776, 737, 694 cm^{-1}; **HRMS (ESI)** m/z: [M+Na]$^+$ Calcd for $C_{14}H_{20}NaO_3Si^+$ 287.1074; Found 287.1070.

2-Methyl-2-((trimethylsilyl)peroxy)pentan-3-one (1f)

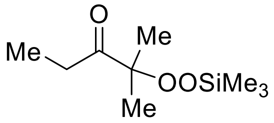

Light yellow oil, 34% isolated yield (34.4 mg, 0.17 mmol). **^{1}H NMR (400 MHz, Chloroform-*d*)**: δ 2.72 (q, *J* = 7.3 Hz, 2H), 1.29 (s, 6H), 1.04 (t, *J* = 7.3 Hz, 3H), 0.18 (s, 9H); **^{13}C NMR (101 MHz, Chloroform-*d*)**: δ 214.2, 88.1, 29.0, 22.0, 7.8, –1.2; **IR (neat)**: 2972, 2940, 1723, 1463, 1412, 1376, 1361, 1252, 1216, 1168, 1101, 1042, 893, 851, 739 cm^{-1}; **HRMS (ESI)** m/z: $[M+Na]^+$ Calcd for $C_9H_{20}NaO_3Si^+$ 227.1074; Found 227.1067.

2,4-Dimethyl-2-((trimethylsilyl)peroxy)pentan-3-one (1g)

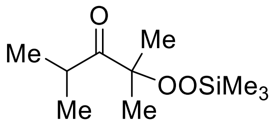

Colorless oil, 60% isolated yield (65.1 mg, 0.30 mmol). **^{1}H NMR (400 MHz, Chloroform-*d*)** δ 3.47–3.33 (m, 1H), 1.30 (s, 6H), 1.07 (d, *J* = 6.8 Hz, 6H), 0.18 (s, 9H); **^{13}C NMR (101 MHz, Chloroform-*d*)**: δ 218.0, 88.3, 33.1, 22.0, 20.1, –1.2; **IR (neat)**: 2972, 2940, 2875, 1721, 1470, 1378, 1363, 1253, 1166, 1096, 1043, 904, 875, 849, 753, 736 cm^{-1}; **HRMS (ESI)** m/z: $[M+Na]^+$ Calcd for $C_{10}H_{22}NaO_3Si^+$ 241.1230; Found 241.1222.

2,6-Dimethyl-2-((trimethylsilyl)peroxy)cyclohexan-1-one (1h)

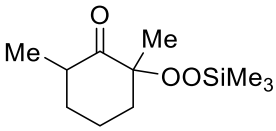

Colorless oil, 60% isolated yield (68.7 mg, 0.30 mmol) as a 74/26 diastereomeric mixture. **^{1}H NMR (400 MHz, Chloroform-*d*)**: δ 3.18–3.02 (m, 0.75H, *major*), 2.56–2.42 (m, 0.25H, *minor*), 2.23–2.14 (m, 0.26H, *minor*), 2.13–1.84 (m, 3H), 1.74–1.68 (m, 0.26H, *minor*), 1.56–1.43 (m, 1.90H), 1.36 (s, 0.74H, *minor*), 1.27 (s, 2.41H, *major*), 1.24–1.19 (m, 0.74H, *major*), 1.09 (d, *J* = 6.7 Hz, 0.77H, *minor*), 1.01 (d, *J* = 6.5 Hz, 2.33H, *major*), 0.19 (s, 2.20H, *minor*), 0.16 (s, 6.79H, *major*); **^{13}C NMR (101 MHz, Chloroform-*d*)** δ 212.8, 210.1, 87.5, 87.0, 43.4, 41.0, 39.6, 37.4, 37.2, 35.0, 21.5, 21.4, 20.8, 19.3, 15.1, 14.5, –1.0, –1.1; **IR (neat)**: 2967, 2936, 2870, 1728, 1457, 1371, 1252,

1157, 1128, 1007, 898, 875, 848, 775, 741, 729 cm^{-1}; **HRMS (ESI)** m/z: $[M+Na]^+$ Calcd for $C_{11}H_{22}NaO_3Si^+$ 253.1230; Found 253.1226.

(2*S*,5*R*)-2-Isopropyl-5-methyl-2-((trimethylsilyl)peroxy)cyclohexan-1-one (1i)

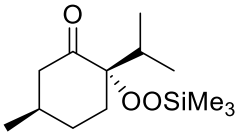

Colorless oil, 74% isolated yield (95.2 mg, 0.37 mmol). $[\alpha]_D^{25}$ = –13.6 (c 1.00 in $CHCl_3$). **^{1}H NMR (400 MHz, Chloroform-*d*)**: δ 2.74 (dd, *J* = 13.7, 5.1 Hz, 1H), 2.56–2.38 (m, 1H), 2.23–2.09 (m, 1H), 2.09–1.85 (m, 4H), 1.45–1.29 (m, 1H), 0.98–0.90 (m, 6H), 0.83 (d, *J* = 7.0 Hz, 3H), 0.18 (s, 9H); **^{13}C NMR (101 MHz, Chloroform-*d*)**: δ 208.4, 90.1, 47.3, 32.4, 29.0, 28.6, 26.9, 20.1, 17.2, 16.1, –1.0; **IR (neat)**: 2960, 2876, 1727, 1459. 1435, 1424, 1384, 1366, 1336, 1288, 1251, 1218, 1196, 1119, 1063, 1034, 1016, 982, 872, 847, 776, 741, 699, 557 cm^{-1}; **HRMS (ESI)** m/z: $[M+Na]^+$ Calcd for $C_{13}H_{26}NaO_3Si^+$ 281.1543; Found 281.1540.

(2*R*,5*R*)-2-Isopropyl-5-methyl-2-((trimethylsilyl)peroxy)cyclohexan-1-one (1j)

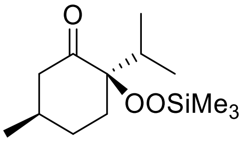

Colorless oil, 75% isolated yield (96.4 mg, 0.37 mmol). $[\alpha]_D^{25}$ = +26.5 (c 1.00 in $CHCl_3$). **^{1}H NMR (400 MHz, Chloroform-*d*)** δ 2.68–2.56 (m, 1H), 2.53 (t, *J* = 12.9 Hz, 1H), 2.27–2.18 (m, 1H), 1.97–1.88 (m, 1H), 1.87–1.70 (m, 1H), 1.63–1.48 (m, 3H), 1.00 (d, *J* = 6.5 Hz, 3H), 0.87 (d, *J* = 6.9 Hz, 3H), 0.86 (d, *J* = 6.9 Hz, 3H), 0.18 (s, 9H); **^{13}C NMR (101 MHz, Chloroform-*d*)**: δ 209.0, 88.8, 47.7, 34.9, 29.0, 28.6, 26.8, 22.5, 18.5, 16.2, –1.1; **IR (neat)**: 2959, 2929, 2876, 1727, 1456, 1434, 1423, 1384, 1365, 1252, 1121, 1091, 1000, 978, 902, 879, 847, 777, 742, 684 cm^{-1}; **HRMS (ESI)** m/z: $[M+Na]^+$ Calcd for $C_{13}H_{26}NaO_3Si^+$ 281.1543; Found 281.1539.

2-Ethyl-2-((trimethylsilyl)peroxy)cyclohexan-1-one (1k)

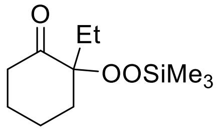

Colorless oil, 72% isolated yield (82.9 mg, 0.36 mmol). **^{1}H NMR (400 MHz, Chloroform-*d*)**: δ 2.95–2.76 (m, 1H), 2.30–2.18 (m, 1H), 2.20–2.09 (m, 1H), 2.08–1.91 (m, 2H), 1.93–1.76 (m, 1H), 1.68–1.48 (m, 3H), 1.49–1.36 (m, 1H), 0.82 (t, *J* = 7.5 Hz, 3H), 0.17 (s, 9H); **^{13}C NMR (101 MHz, Chloroform-*d*)**: δ 212.2, 89.6, 40.5, 36.3, 29.3, 24.6, 22.1, 8.0, 0.0; **IR (neat) :** 2967, 2943, 2865, 1728, 1463, 1449, 1434, 1310, 1251, 1124, 1086, 906, 882, 846, 808, 781, 741, 692 cm^{-1}. **HRMS (ESI)** m/z: [M+Na]$^+$ Calcd for $C_{11}H_{22}NaO_3Si^+$ 253.1230; Found 253.1230.

[B] Synthesis of α -ketoalkylsilyl peroxide 1m[13]

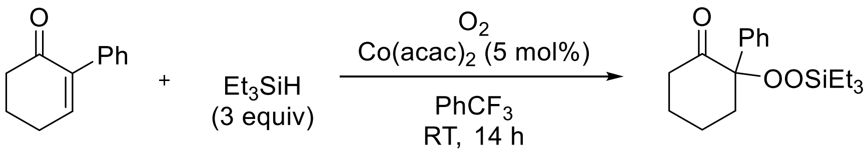

1m

To a solution of 2-phenyl-2-cyclohexen-1-one (172 mg, 1 mmol) and triethylsilane (349 mg, 3 mmol, 3 equiv) in trifluoromethylbenzene (2.0 mL) was added cobalt(II) acetylacetonate (13 mg, 0.05 mmol, 5 mol%). The mixture was stirred vigorously at room temperature under O_2 atmosphere. After stirring for 14 h, the volatiles were removed under reduced pressure. The residue was purified by column chromatography on silica gel (eluted with DCM : hexane = 1:1) to give target alkylsilyl peroxide **1m**.

2-Phenyl-2-((triethylsilyl)peroxy)cyclohexan-1-one (1m)

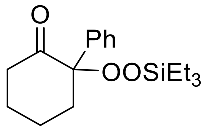

Colorless oil, 17% isolated yield (55 mg, 0.17 mmol). **^{1}H NMR (500 MHz, Chloroform-*d*)**: δ 7.45–7.41 (m, 2H), 7.39–7.33 (m, 2H), 7.32–7.28 (m, 1H), 2.97–2.84 (m, 1H), 2.45–2.39 (m, 1H),, 2.38–2.32 (m, 1H), 2.23–2.15 (m, 1H), 2.12–1.98 (m, 2H), 1.88–1.78 (m, 1H), 1.73–1.65 (m, 1H), 0.98 (t, J = 7.9 Hz, 9H), 0.76–0.69 (m, 6H); **^{13}C NMR (126 MHz, Chloroform-*d*)**: δ 207.1, 138.1, 127.8, 127.7, 127.5, 90.7, 40.2, 38.9, 27.8, 21.8, 6.8, 3.8; **IR(neat)**: 2953, 2913, 2876, 1731, 1686, 1449, 1413, 1288, 1275, 1220, 1179, 1124, 1078, 1018, 1004, 989, 970, 845, 806, 786, 729, 696, 566 cm^{-1}; **HRMS (ESI)** m/z: $[M+Na]^+$ Calcd for $C_{18}H_{28}NaO_3Si^+$ 343.1700; Found 343.1702.

[C] Synthesis of β-acetoxyalkylsilyl and β-amidoalkylsilyl peroxides

I. Synthesis of β-acetoxyalkylsilyl peroxide 32

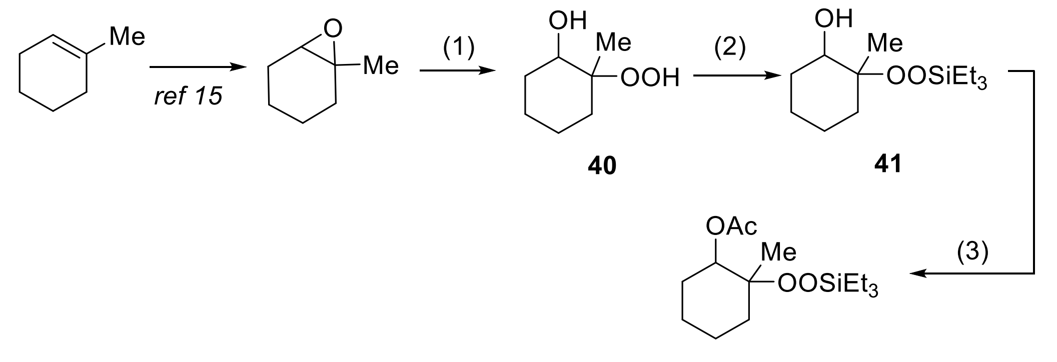

(1) Synthesis of 38[14]

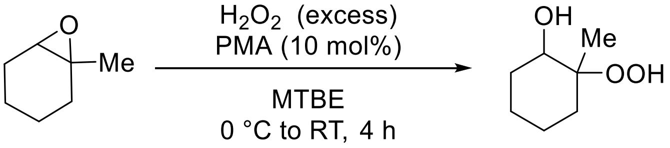

To the solution of 1-methyl-7-oxabicyclo[4.1.0]heptane[15] (4.5 g, 40 mmol) and H_2O_2 in *tert*-butyl methyl ether[4] (200 mL) was added phosphomolybdic acid n-hydrate (PMA, 600 mg, ca. 4.0 mmol, 10 mol%) at 0 °C. After stirring at room temperature for 4 h, the resulting mixture was quenched with water (150 mL). The organic layer was separated and aqueous layer was extracted with ethyl acetate (50 mL × 3). The organic layers

were combined, washed with water (150 mL) and then brine (150 mL). This was dried over Na_2SO_4, filtrated and concentrated under reduced pressure. The residue was purified by column chromatography on silica gel (eluted with petroleum ether/ethyl acetate = 5/1 to 3/1, gradient) to give product **40**.

2-Hydroperoxy-2-methylcyclohexan-1-ol (40)

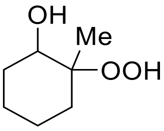

Colorless oil, 60% isolated yield (3.50 g, 23.9 mmol). **^{1}H NMR (400 MHz, Chloroform-*d*)**: δ 3.94 (dd, *J* = 10.9, 4.7 Hz, 1H), 1.97–1.85 (m, 1H), 1.78–1.57 (m, 4H), 1.40–1.22 (m, 3H), 1.21 (s, 3H); **^{13}C NMR (101 MHz, Chloroform-*d*)**: δ 86.1, 73.1, 33.4, 31.0, 24.03, 23.0, 15.1; **IR (neat)**: 3325, 2935, 2865, 1448, 1377, 1353, 1282, 1152, 1136, 1110, 1069, 1045, 998, 983, 946, 848, 832, 718, 598, 574, 494, 467, 449 cm^{-1}; **HRMS (ESI)** m/z: $[M+Na]^+$ Calcd for $C_7H_{14}NaO_3^+$ 169.0835; Found 169.0835.

(2) Synthesis of 41

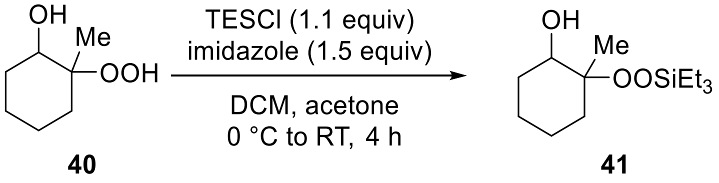

To a solution of **40** (3.5 g, 23.9 mmol) and 1*H*-imidazole (2.45 g, 35.9 mmol, 1.5 equiv) in dry DCM (90 mL) and acetone (30 mL) was added triethylsilyl chloride (TESCl, 4.0 g, 26.3 mmol, 1.5 equiv) slowly at 0 °C. After stirring at room temperature for 4 h, the resulting mixture was quenched with water (30 mL) and extracted with DCM (30 mL × 3). The combined organic layer was washed with water (150 mL) and then brine (50 mL). This was dried over Na_2SO_4, filtrated and concentrated under reduced pressure.

The residue was purified by column chromatography on silica gel (eluted with petroleum ether to petroleum ether/ethyl acetate =30/1, gradient) to give product **41**.

2-Methyl-2-((triethylsilyl)peroxy)cyclohexan-1-ol (41)

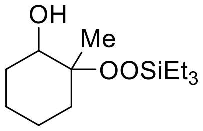

Colorless oil. 56% isolated yield (3.48 g, 13.4 mmol). **^{1}H NMR (400 MHz, Chloroform-*d*)**: δ 3.86 (dd, *J* = 10.9, 4.9 Hz, 1H), 3.30 (s, 1H), 1.91–1.81 (m, 1H), 1.72–1.60 (m, 2H), 1.56–1.48 (m, 1H), 1.41–1.21 (m, 7H), 0.99 (t, *J* = 7.9 Hz, 9H), 0.69 (q, *J* = 8.0 Hz, 6H); **^{13}C NMR (101 MHz, Chloroform-*d*)**: δ 85.8, 75.9, 33.8, 30.9, 23.9, 23.1, 15.2, 6.9, 4.0; **IR (neat)**: 2938, 2876, 1459, 1412, 1379, 1352, 1280, 1238, 1174, 1151, 1135, 1109, 1080, 1041, 1004, 974, 948, 833, 804, 788, 727, 676, 595, 554, 515, 470, 436 cm^{-1}; **HRMS (ESI)** m/z: $[M+Na]^+$ Calcd for $C_{13}H_{28}NaO_3Si^+$ 283.1700; Found 283.1697.

(3) Synthesis of 32

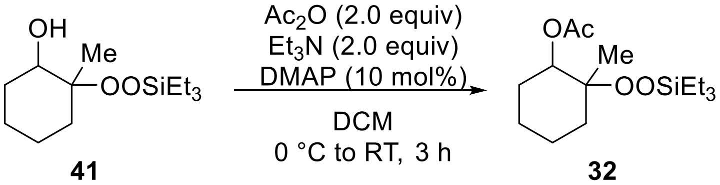

41 **32**

To a solution of **41** (52.1 mg, 0.20 mmol), triethylamine (40.4 mg, 0.40 mmol, 2.0 equiv) and 4-dimethylaminopyridine (DMAP, 2.4 mg, 0.020 mmol, 10 mol%) in DCM (1.0 mL) was added acetic anhydride (40.8 mg, 0.40 mmol, 2.0 equiv) at 0 °C. After stirring at room temperature for 3 h, the reaction mixture was quenched with saturated $NaHCO_3$ aq. (5.0 mL). The mixture was extracted with DCM (10 mL × 3) and the organic layer was washed with water (10 mL) and then brine (10 mL). This was dried over Na_2SO_4, filtrated and concentrated under reduced pressure. The residue was purified by column

chromatography on silica gel (petroleum ether to petroleum ether/ethyl acetate = 50/1, gradient) to give alkylsilyl peroxide **32**.

2-Methyl-2-((triethylsilyl)peroxy)cyclohexyl acetate (32)

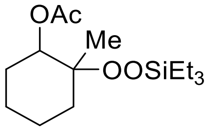

Colorless oil, 56% isolated yield (34.0 mg, 0.11 mmol). **^{1}H NMR (400 MHz, Chloroform-*d*)**: δ 5.12 (dd, *J* = 7.5, 3.9 Hz, 1H), 2.04 (s, 3H), 1.93–1.83 (m, 1H), 1.79–1.71 (m, 1H), 1.69–1.62 (m, 1H), 1.61–1.45 (m, 3H), 1.43–1.30 (m, 2H), 1.18 (s, 3H), 0.96 (t, *J* = 7.9 Hz, 9H), 0.64 (q, *J* = 7.6 Hz, 6H); **^{13}C NMR (101 MHz, Chloroform-*d*)**: δ 170.4, 82.6, 72.8, 33.4, 27.9, 22.0, 22.0, 21.5, 18.9, 6.9, 4.0; **IR (neat)**: 2951, 2938, 2877, 1743, 1460, 1370, 1234, 1185, 1039, 1016, 1007, 979, 856, 834, 802, 739, 728, 676, 492 cm^{-1}; **HRMS (ESI)** m/z: [M+Na]$^+$ Calcd for $C_{15}H_{30}NaO_4Si^+$ 325.1806; Found 325.1802.

II. Synthesis of β-amidoalkylsilyl peroxide 34

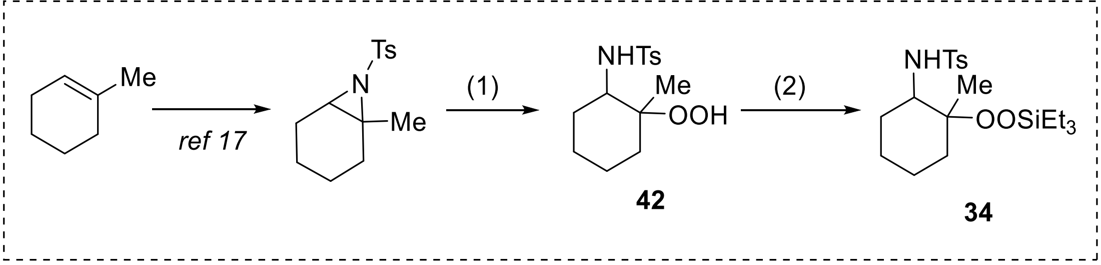

(1) Synthesis of 42[16]

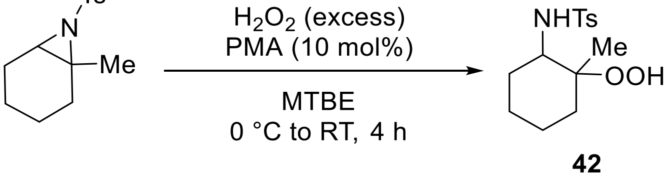

To a mixture of 1-methyl-7-tosyl-7-azabicyclo[4.1.0]heptane[17] (1.72 g, 6.5 mmol) and H_2O_2 in *tert*-butyl methyl ether[4] (30 mL) was added PMA (82.5 mg, ca. 0.55 mmol, 10 mol%) at 0 °C. After stirring at room temperature for 4 h, the mixture was quenched

with water and diluted with ethyl acetate (30 mL). The organic layer was separated and the aqueous layer was extracted with ethyl acetate (10 mL × 3). The organic layers were combined, washed with water (20 mL) and then brine (20 mL). This was filtrated, dried over Na_2SO_4 and concentrated under reduced pressure. The residue was purified by column chromatography on silica gel (eluted with petroleum ether/ethyl acetate = 4/1) to give product **42**.

***N*-(2-Hydroperoxy-2-methylcyclohexyl)-4-methylbenzenesulfonamide (42)**

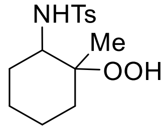

White solid, 43% isolated yield. (0.83 g, 2.8 mmol). **m.p.** 119.5–120.7 °C **^{1}H NMR (400 MHz, Chloroform-*d*)**: δ 9.01 (s, 1H), 7.80 (d, *J* = 8.3 Hz, 2H), 7.32 (d, *J* = 8.1 Hz, 2H), 4.99 (d, *J* = 8.6 Hz, 1H), 3.63–3.48 (m, 1H), 2.43 (s, 3H), 2.04–1.91 (m, 1H), 1.68–1.52 (m, 3H), 1.47–1.39 (m, 1H), 1.32–1.18 (m, 2H), 1.17–1.06 (m, 1H), 1.04 (s, 3H); **^{13}C NMR (101 MHz, Chloroform-*d*)**: δ 143.8, 137.7, 130.0, 127.0, 84.3, 54.5, 33.8, 30.7, 25.0, 23.0, 21.7, 16.2; **IR (neat)**: 3365, 3340, 3276, 2937, 2867, 1598, 1496, 1434, 1399, 1381, 1333, 1317, 1304, 1288, 1261, 1199, 1148, 1077, 1052, 1018, 965, 909, 865, 848, 824, 815, 727, 706, 663, 618, 592, 572, 543, 505, 474, 443 cm^{-1}; **HRMS (ESI)** m/z: $[M+Na]^+$ Calcd for $C_{14}H_{21}NNaO_4S^+$ 322.1084; Found 322.1080.

(2) Synthesis of 34

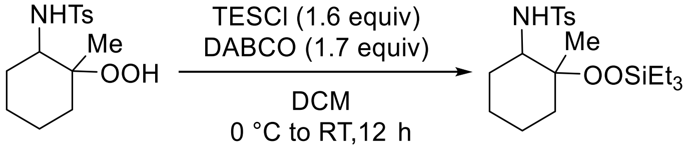

42 **34**

To a solution of **42** (1.31 g, 4.4 mmol) and DABCO (0.84 g, 7.5 mmol, 1.7 equiv) in dry DCM (25 mL) was added TESCl (1.06 g, 7.0 mmol, 1.6 equiv) slowly at 0 °C. After stirring at room temperature for 12 h, the reaction mixture was diluted with petroleum

ether (50 mL). The organic layer was washed with water (30 mL × 3) and then brine (30 mL). This was dried over Na_2SO_4, filtrated and concentrated under reduced pressure. The residue was purified by column chromatography on silica gel (eluted with petroleum ether/ethyl acetate = 50/1 to 30/1, gradient) to give alkylsilyl peroxide **34**.

4-Methyl-*N*-(2-methyl-2-((triethylsilyl)peroxy)cyclohexyl)benzenesulfonamide (34)

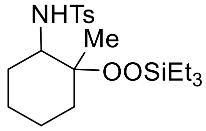

White solid, 49% isolated yield (0.89 g, 2.2 mmol). **m.p.** 69.9–70.8 °C. **^{1}H NMR (400 MHz, Chloroform-*d*)**: δ 7.69 (d, *J* = 8.2 Hz, 2H), 7.21 (d, *J* = 8.3 Hz, 2H), 5.04 (d, *J* = 3.6 Hz, 1H), 3.33–3.22 (m, 1H), 2.35 (s, 3H), 2.15–2.04 (m, 1H), 1.58–1.36 (m, 4H), 1.29–1.11 (m, 3H), 1.07 (s, 3H), 0.85 (t, *J* = 8.0 Hz, 9H), 0.51 (q, *J* = 7.9 Hz, 6H); **^{13}C NMR (101 MHz, Chloroform-*d*)**: δ 143.2, 137.2, 129.6, 127.4, 83.7, 56.3, 33.9, 28.6, 22.9, 22.4, 21.6, 17.7, 6.8, 3.9; **IR (neat)**: 3279, 2955, 2933, 2875, 1448, 1423, 1372, 1327, 1240, 1187, 1163, 1143, 1094, 1050, 1007, 975, 936, 904, 880, 852, 834, 816, 801, 739, 707, 669, 611, 566, 554, 519, 500, 485, 459, 444 cm^{-1}; **HRMS (ESI)** m/z: $[M+Na]^+$ Calcd for $C_{20}H_{35}NNaO_4SSi^+$ 436.1948; Found 436.1945.

4. Procedures for the transformation of alkylsilyl peroxides

(1) General procedure for coupling reaction of α-ketoalkylsilyl peroxide

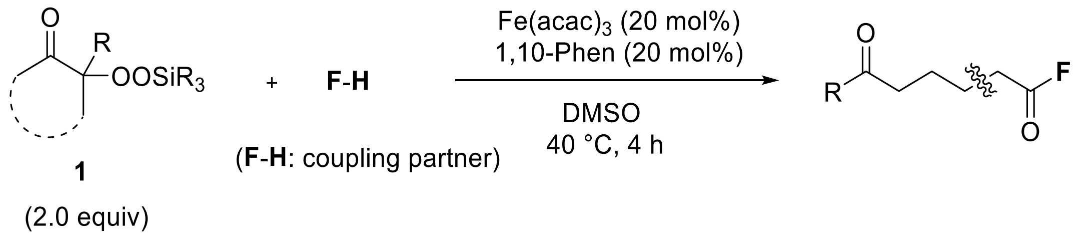

To a solution of coupling partner (0.20 mmol), tris(2,4-pentanedionato)iron(III) ($Fe(acac)_3$,14.1 mg, 0.040 mmol, 20 mol%) and 1,10-phenanthoroline (1,10-Phen, 7.2 mg, 0.040 mmol, 20 mol%) in dry DMSO (1.0 mL) was added α-ketoalkylsilyl peroxide **1** (0.40 mmol, 2.0 equiv) dropwise at room temperature under argon atmosphere. The reaction mixture was stirred at 40 °C for 4 h. After being cooled to room temperature, the reaction mixture was quenched with water (2.0 mL) and extracted with ethyl acetate (15 mL × 3). The combined organic layer was washed with water (10 mL × 3), saturated K_2CO_3 (10 mL) aq. and then brine (10 mL). This was dried over Na_2SO_4, filtrated and concentrated under reduce pressure. The residue was purified by column chromatography on silica gel (eluted with petroleum ether/ethyl acetate) to afford the corresponding coupling products.

1-(1,3-Dimethyl-2-oxoindolin-3-yl)octane-2,7-dione (8a)

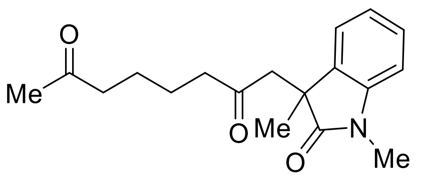

Colorless oil, 95% isolated yield (57.5 mg, 0.19 mmol). **^{1}H NMR (400 MHz, Chloroform-*d*)**: δ 7.22 (t, *J* = 7.7 Hz, 1H), 7.10 (d, *J* = 7.3 Hz, 1H), 6.97 (t, *J* = 7.5 Hz, 1H), 6.84 (d, *J* = 7.8 Hz, 1H), 3.24 (s, 3H), 3.05 (s, 2H), 2.35–2.17 (m, 4H), 2.05 (s, 3H), 1.41–1.32 (m, 4H), 1.30 (s, 3H); **^{13}C NMR (101 MHz, Chloroform-*d*)**: δ 208.8, 206.7, 180.5, 143.8, 133.6, 128.0, 122.3, 121.8, 108.3, 49.8, 45.3, 43.4, 42.6, 30.0, 26.5, 24.6, 23. 1, 22.9; **IR (neat)**: 3500, 3410, 3055, 2931, 1713, 1613, 1494, 1471, 1350,

1310, 1249, 1161, 1125, 1103, 1074, 1048, 1031, 1019, 949, 755, 701, 589, 544, 489, 475 cm^{-1}; **HRMS (ESI)** m/z: $[M+H]^+$ Calcd for $C_{18}H_{24}NO_3^+$ 302.1751; Found 302.1747.

1-(1,3-Dimethyl-2-oxoindolin-3-yl)heptane-2,6-dione (8b)

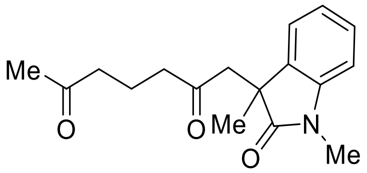

Light yellow oil, 77% isolated yield (44.3 mg 0.15 mmol). **^{1}H NMR (400 MHz, Chloroform-*d*)**: δ 7.29–7.20 (m, 1H), 7.11 (d, *J* = 7.4 Hz, 1H), 6.99 (t, *J* = 7.5 Hz, 1H), 6.85 (d, *J* = 7.8 Hz, 1H), 3.26 (s, 3H), 3.05 (s, 2H), 2.33–2.21 (m, 4H), 2.03 (s, 3H), 1.70 – 1.58 (m, 2H), 1.31 (s, 3H); **^{13}C NMR (101 MHz, Chloroform-*d*)**: δ 208.4, 206.6, 180.5, 143.8, 133.5, 128.1, 122.3, 121.8, 108.3, 49.7, 45.3, 42.2, 41.6, 29.9, 26.5, 24.6, 17.4; **IR (neat)**: 3496, 3410, 3055, 2964, 2929, 1701, 1612, 1493, 1470, 1451, 1378, 1350, 1310, 1250, 1163, 1125, 1101, 1068, 1044, 1019, 948, 753, 701, 543, 489, 474 cm^{-1}; **HRMS (ESI)** m/z: $[M+H]^+$ Calcd for $C_{17}H_{22}NO_3^+$ 288.1594; Found 288.1586.

1-(1,3-Dimethyl-2-oxoindolin-3-yl)nonane-2,8-dione (8c)

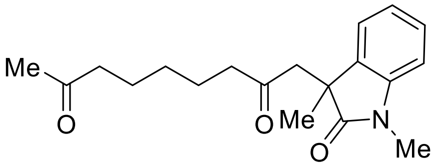

Light yellow oil, 90% isolated yield (56.9 mg, 0.18 mmol). **^{1}H NMR (400 MHz, Chloroform-*d*)**: δ 7.28–7.19 (m, 1H), 7.11 (d, *J* = 7.2 Hz, 1H), 6.99 (t, *J* = 7.5 Hz, 1H), 6.85 (d, *J* = 7.7 Hz, 1H), 3.25 (s, 3H), 3.05 (s, 2H), 2.33 (t, *J* = 7.4 Hz, 2H), 2.32–2.14 (m, 2H), 2.08 (s, 3H), 1.52–1.33 (m, 4H), 1.31 (s, 3H), 1.17–1.04 (m, 2H); **^{13}C NMR (101 MHz, Chloroform-*d*)**: δ 209.1, 206.9, 180.5, 143.8, 133.6, 128.0, 122.3, 121.8, 108.3, 49.8, 45.3, 43.4, 42.6, 30.0, 28.5, 26.5, 24.6, 23.5, 23.2; **IR (neat)**: 3406, 3055, 2926, 1701, 1613, 1494, 1468, 1377, 1349, 1310, 1249, 1159, 1124, 1103, 1077, 1058,

1031, 1018, 949, 753, 701, 588, 543, 488, 476 cm^{-1}; **HRMS (ESI)** m/z: $[M+H]^+$ Calcd for $C_{19}H_{26}NO_3^+$ 316.1907; Found 316.1901.

1-(1,3-Dimethyl-2-oxoindolin-3-yl)decane-2,9-dione (8d)

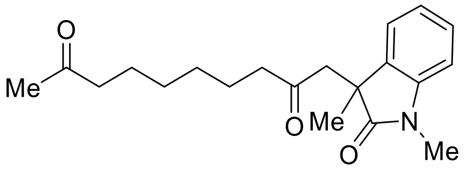

Light yellow oil, 84% isolated yield (55.2 mg, 0.17 mmol). **^{1}H NMR (400 MHz, Chloroform-*d*)**: δ 7.28–7.19 (m, 1H), 7.11 (d, *J* = 7.2 Hz, 1H), 6.99 (t, *J* = 7.5 Hz, 1H), 6.85 (d, *J* = 7.8 Hz, 1H), 3.25 (s, 3H), 3.05 (s, 2H), 2.35 (t, *J* = 7.4 Hz, 2H), 2.33–2.13 (m, 2H), 2.09 (s, 3H), 1.54–1.42 (m, 2H), 1.43–1.34 (m, 2H), 1.31 (s, 3H), 1.24–1.05 (m, 4H); **^{13}C NMR (101 MHz, Chloroform-*d*)**: δ 209.2, 207.1, 180.5, 143.8, 133.6, 128.0, 122.3, 121.8, 108.3, 49.8, 45.3, 43.7, 42.8, 30.0, 28.9, 28.8, 26.5, 24.6, 23.6, 23.3; **IR (neat)**: 3408, 3054, 2930, 2860, 1701, 1613, 1494, 1471, 1452, 1420, 1378, 1351, 1311, 1249, 1160, 1125, 1105, 1082, 1061, 1032, 1018, 755, 701, 589, 544, 489, 475 cm^{-1}; **HRMS (ESI)** m/z: $[M+H]^+$ Calcd for $C_{20}H_{28}NO_3^+$ 330.2064; Found 330.2057.

1,3-Dimethyl-3-(2-oxo-2-(2-(3-oxobutyl)phenyl)ethyl)indolin-2-one (8e)

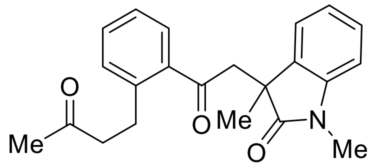

Yellow oil, 65% isolated yield (45.3 mg, 0.13 mmol). **^{1}H NMR (400 MHz, Chloroform-*d*)**: δ 7.55 (dd, *J* = 7.7, 1.5 Hz, 1H), 7.32 (td, *J* = 7.5, 1.5 Hz, 1H), 7.29–7.19 (m, 2H), 7.13 (d, *J* = 7.4 Hz, 2H), 6.98 (td, *J* = 7.5, 1.0 Hz, 1H), 6.84 (d, *J* = 7.8 Hz, 1H), 3.63 (d, *J* = 17.2 Hz, 1H), 3.53 (d, *J* = 17.2 Hz, 1H), 3.21 (s, 3H), 2.72–2.60 (m, 1H), 2.62–2.50 (m, 1H), 2.50–2.33 (m, 2H), 1.99 (s, 3H), 1.40 (s, 3H); **^{13}C NMR (101 MHz, Chloroform-*d*)**: δ 208.4, 200.7, 180.5, 144.0, 141.2, 137.3, 133.4, 131.6, 131.3, 128.7, 128.1, 126.2, 122.3, 122.0, 108.3, 49.3, 45.8, 45.4, 29.8, 28.1, 26.5, 24.9; **IR (neat)**: 3056, 3023, 2965, 2928, 1701, 1612, 1571, 1494, 1470, 1450, 1420, 1378,

1348, 1310, 1279, 1250, 1211, 1160, 1125, 1105, 1091, 1060, 1032, 1018, 1007, 943, 755, 701, 647, 596, 544, 520, 488, 475 cm^{-1}; **HRMS (ESI)** m/z: $[M+H]^+$ Calcd for $C_{22}H_{24}NO_3^+$ 350.1751; Found 350.1744.

1,3-Dimethyl-3-(2-oxobutyl)indolin-2-one (8f)[18, 19, 20]

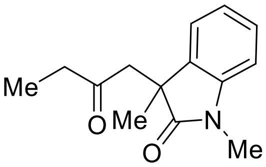

Light yellow oil, 89% isolated yield (41.2 mg, 0.18 mmol). **^{1}H NMR (400 MHz, Chloroform-*d*)**: δ 7.29–7.20 (m, 1H), 7.12 (d, *J* = 6.8 Hz, 1H), 7.00 (t, *J* = 7.5 Hz, 1H), 6.86 (d, *J* = 7.7 Hz, 1H), 3.27 (s, 3H), 3.07 (s, 2H), 2.38–2.17 (m, 2H), 1.33 (s, 3H), 0.88 (t, *J* = 7.3 Hz, 3H); **^{13}C NMR (101 MHz, Chloroform-*d*)**: δ 207.5, 180.6, 143.8, 133.7, 128.0, 122.3, 121.8, 108.3, 49.5, 45.3, 36.0, 26.5, 24.6, 7.5.

1,3-Dimethyl-3-(3-methyl-2-oxobutyl)indolin-2-one (8g)[20]

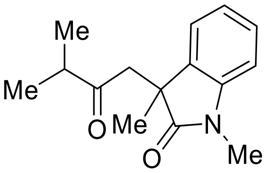

For 24 h. Light yellow oil, 57% isolated yield (27.9 mg, 0.11 mmol). **^{1}H NMR (400 MHz, Chloroform-*d*)**: δ 7.29–7.20 (m, 1H), 7.11 (d, *J* = 7.3 Hz, 1H), 6.99 (td, *J* = 7.5, 1.0 Hz, 1H), 6.86 (d, *J* = 7.8 Hz, 1H), 3.27 (s, 3H), 3.16 (d, *J* = 17.8 Hz, 1H), 3.10 (d, *J* = 17.8 Hz, 1H), 2.51–2.39 (m, 1H), 1.33 (s, 3H), 0.98 (d, *J* = 6.9 Hz, 3H), 0.94 (d, *J* = 7.0 Hz, 3H); **^{13}C NMR (101 MHz, Chloroform-*d*)**: δ 210.8, 180.7, 143.9, 133.8, 128.0, 122.2, 121.7, 108.3, 47.7, 45.3, 40.7, 26.5, 24.7, 18.1, 17.9.

1-(1,3-Dimethyl-2-oxoindolin-3-yl)-3-methyloctane-2,7-dione (8h)

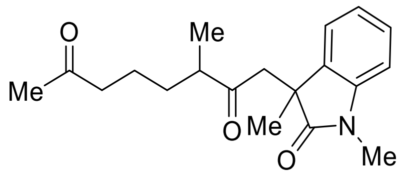

For 12 h. Light yellow oil, 37% isolated yield (23.1 mg, 0.074 mmol) as a 1:1 diastereomeric mixture. **^{1}H NMR (400 MHz, Chloroform-*d*)**: δ 7.28–7.18 (m, 1H), 7.11 (d, *J* = 7.4 Hz, 1H), 7.02–6.93 (m, 1H), 6.85 (d, *J* = 7.8 Hz, 1H), 3.26 (s, 3H), 3.20–3.05 (m, 2H), 2.42–2.25 (m, 3H), 2.09 (s, 1.5H), 2.07 (s, 1.5H), 1.56–1.37 (m, 2H), 1.35–1.30 (m, 3H), 1.31–1.13 (m, 2H), 0.96 (d, *J* = 7.0 Hz, 1.5H), 0.92 (d, *J* = 7.0 Hz, 1.5H); **^{13}C NMR (101 MHz, Chloroform-*d*)**: δ 210.6, 210.5, 208.7, 208.6, 180.6, 143.9, 143.9, 133.7, 133.7, 128.0, 122.2 122.2, 121.7, 121.7, 108.3, 48.5, 48.3, 46.0, 45.9, 45.2, 45.2, 43.5, 43.5, 43.5, 42.8, 32.1, 32.0, 30.0, 26.5, 24.7, 24.7, 21.4, 21.2, 16.2, 16.1; **IR (neat)**: 2960, 2926, 1712, 1614, 1494, 1471, 1453, 1420, 1378, 1351, 1309, 1249, 1164, 1145, 1125, 1092, 1047, 1031, 1019, 754, 742, 544 cm^{-1}; **HRMS (ESI)** m/z: $[M+H]^+$ Calcd for $C_{19}H_{26}NO_3^+$ 316.1907; Found 316.1902.

(4*R*)-1-(1,3-Dimethyl-2-oxoindolin-3-yl)-4,8-dimethylnonane-2,7-dione (8i)

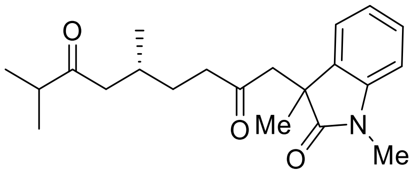

Using 2.0 equiv. of **1i** for 12 h. Light yellow oil, 99% isolated yield (68.0 mg, 0.20 mmol) as a 1:1 diastereomeric mixture. **^{1}H NMR (400 MHz, Chloroform-*d*)**: δ 7.23 (td, *J* = 7.7, 1.3 Hz, 1H), 7.15–7.07 (m, 1H), 6.98 (td, *J* = 7.5, 1.0 Hz, 1H), 6.84 (d, *J* = 7.8 Hz, 1H), 3.25 (s, 3H), 3.05 (s, 2H), 2.64–2.42 (m, 1H), 2.40–2.23 (m, 2H), 2.27 – 2.13 (m, 1H), 2.15–2.00 (m, 1H), 1.88–1.73 (m, 1H), 1.47–1.32 (m, 1H), 1.31 (s, 1.5H), 1.31 (s, 1.5H), 1.31–1.20 (m, 1H), 1.08–0.99 (m, 6H), 0.72 (d, *J* = 6.6 Hz, 1.5H), 0.70 (d, *J* = 6.6 Hz, 1.5H); **^{13}C NMR (101 MHz, Chloroform-*d*)**: δ 214.7, 214.6, 206.7, 206.6, 180.4, 180.4, 143.8, 143.8, 133.6, 133.5, 128.0, 122.3, 122.3, 121.8, 121.8, 108.3,

108.3, 50.4, 50.3, 50.2, 50.1, 45.4, 45.3, 40.9, 40.9, 37.9, 37.9, 30.6, 30.5, 28.8, 28.7, 26.5, 24.6, 24.6, 19.6, 19.6, 18.4, 18.4, 18.4; **IR (neat)**: 3405, 3055, 2965, 1701, 1616, 1494, 1468, 1380, 1351, 1310, 1250, 1157, 1125, 1067, 1018, 958, 935, 753, 701, 587, 567, 543, 489, 474 cm^{-1}; **HRMS (ESI)** m/z: $[M+H]^+$ Calcd for $C_{21}H_{30}NO_3^+$ 344.2220; Found 344.2213.

1-(1,3-Dimethyl-2-oxoindolin-3-yl)nonane-2,7-dione (8k)

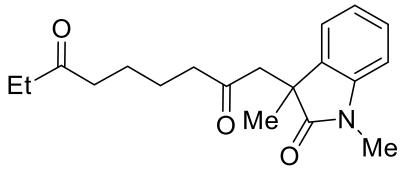

Light yellow oil, 73% isolated yield (46.0 mg, 0.15 mmo). **^{1}H NMR (400 MHz, Chloroform-*d*)**: δ 7.24 (td, *J* = 7.8, 1.2 Hz, 1H), 7.16–7.05 (m, 1H), 7.03–6.94 (m, 1H), 6.85 (d, *J* = 7.8 Hz, 1H), 3.25 (s, 3H), 3.06 (s, 2H), 2.39–2.15 (m, 6H), 1.45–1.36 (m, 4H), 1.32 (s, 3H), 1.00 (t, *J* = 7.3 Hz, 3H). **^{13}C NMR (101 MHz, Chloroform-*d*)**: δ 211.3, 206.7, 180.4, 143.8, 133.6, 128.0, 122.3, 121.8, 108.3, 49.8, 45.3, 42.6, 42.0, 36.0, 26.5, 24.6, 23.2, 23.0, 7.9; **IR (neat)**: 2928, 1705, 1613, 1494, 1471, 1452, 1410, 1378, 1350, 1310, 1250, 1125, 1055, 1033, 1018, 755, 544, 474 cm^{-1}; **HRMS:** m/z: $[M+H]^+$ Calcd for $C_{19}H_{26}NO_3^+$ 316.1907; Found 364.1904.

7-(1,3-Dimethyl-2-oxoindolin-3-yl)-1-phenylheptane-1,6-dione (8m)

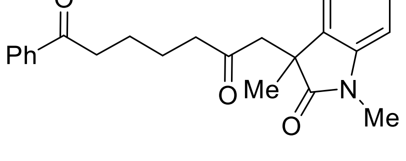

At 80 °C for 16 h. Orange oil, 27% isolated yield (19.5 mg, 0.054 mmol). **^{1}H NMR (400 MHz, Chloroform-d)**: δ 7.93–7.86 (m, 2H), 7.57–7.51 (m, 1H), 7.47–7.42 (m, 2H), 7.26–7.20 (m, 1H), 7.15–7.10 (m, 1H), 7.01–6.96 (m, 1H), 6.85 (d, *J* = 7.8 Hz, 1H), 3.26 (s, 3H), 3.08 (s, 2H), 2.87 (t, *J* = 7.1 Hz, 2H), 2.41–2.22 (m, 2H), 1.63–1.54 (m, 2H), 1.54–1.45 (m, 2H), 1.33 (s, 3H); **^{13}C NMR (101 MHz, Chloroform-d)**: δ 206.8, 200.0, 180.5, 143.8, 137.1, 133.6, 133.1, 128.7, 128.1, 128.0, 122.4, 121.9, 108.3,

49.85, 45.4, 42.7, 38.3, 26.5, 24.6, 23.6, 23.2; **IR (neat)**: 2926, 1708, 1683, 1613, 1597, 1494, 1470, 1449, 1378, 1350, 1310, 1251, 1221, 1180, 1158, 1124, 1099, 1018, 1002, 800, 753, 691, 568, 543, 489, 474 cm^{-1}. **HRMS:** m/z: $[M+H]^+$ Calcd for $C_{23}H_{26}NO_3^+$ 364.1907; Found 364.1903.

3-Isobutyl-1,3-dimethylindolin-2-one (9g)[21]

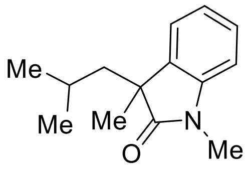

For 24 h. Light yellow oil, 40% isolated yield (17.3 mg, 0.087 mmol). **^{1}H NMR (400 MHz, Chloroform-*d*)**: δ 7.31–7.20 (m, 1H), 7.16 (d, *J* = 7.4 Hz, 1H), 7.06 (t, *J* = 7.5 Hz, 1H), 6.84 (d, *J* = 7.8 Hz, 1H), 3.21 (s, 3H), 1.94 (dd, *J* = 13.9, 7.6 Hz, 1H), 1.76 (dd, *J* = 13.9, 5.4 Hz, 1H), 1.32 (s, 3H), 1.30–1.19 (m, 1H), 0.65 (d, *J* = 6.6 Hz, 3H), 0.60 (d, *J* = 6.7 Hz, 3H); **^{13}C NMR (101 MHz, Chloroform-*d*)**: δ 181.2, 143.3, 134.4, 127.7, 123.0, 122.5, 108.1, 48.2, 46.9, 26.3, 26.3, 25.7, 24.3, 23.0.

1,3-Dimethyl-3-(2-methyl-6-oxoheptyl)indolin-2-one (9h)

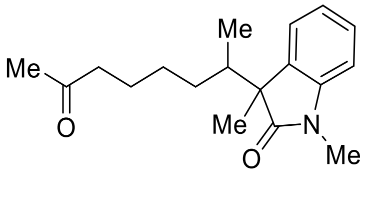

For 12 h. Colorless oil, 43% isolated yield (25.0 mg, 0.086 mmol) as a 1:1 diastereomeric mixture. **^{1}H NMR (400 MHz, Chloroform-*d*)**: δ 7.25 (t, *J* = 7.7 Hz, 1H), 7.14 (t, *J* = 7.7 Hz, 1H), 7.04 (t, *J* = 7.5 Hz, 1H), 6.84 (d, *J* = 7.8 Hz, 1H), 3.21 (s, 3H), 2.27–2.09 (m, 2H), 2.05 (s, 3H), 2.03–1.96 (m, 0.5H), 1.88–1.80 (m, 1H), 1.70–1.62 (m, 0.5H), 1.55–1.32 (m, 2H), 1.31 (s, 3H), 1.17–0.81 (m, 3H), 0.62 (d, *J* = 6.4 Hz, 1.5H), 0.51 (d, *J* = 6.5 Hz, 1.5H); **^{13}C NMR (101 MHz, Chloroform-*d*)**: δ 209.3, 209.2, 181.2, 181.0, 143.3, 143.2, 134.4, 134.1, 127.8, 127.8, 123.0, 122.9, 122.5, 122.5, 108.1, 108.1, 48.2, 48.0, 45.2, 44.7, 43.8, 43.8, 37.6, 36.7, 30.1, 30.0, 29.9, 26.3, 26.3,

26.2, 26.0, 20.9, 20.9, 20.8, 20.0; **IR (neat)**: 3055, 2956, 2925, 2870, 1709, 1613, 1493, 1469, 1421, 1377, 1347, 1308, 1249, 1166, 1124, 1086, 1051, 1021, 754, 742, 700, 543, 488 cm^{-1}; **HRMS (ESI)** m/z: $[M+H]^+$ Calcd for $C_{18}H_{26}NO_2^+$ 288.1958; Found 288.1953.

1-(4-Methyl-3-oxo-3,4-dihydroquinoxalin-2-yl)heptane-1,6-dione (11)

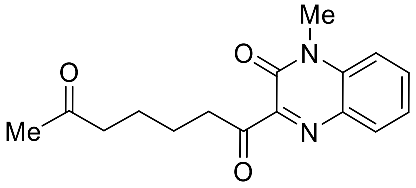

Yellow solid, 91% isolated yield (51.9 mg, 0.18 mmol). **m.p.** 60.8–62.3 °C. **^{1}H NMR (400 MHz, Chloroform-*d*)**: δ 7.93 (dd, *J* = 8.0, 1.5 Hz, 1H), 7.71–7.61 (m, 1H), 7.44–7.35 (m, 1H), 7.35 (d, *J* = 8.5 Hz, 1H), 3.72 (s, 3H), 3.09 (t, *J* = 7.0 Hz, 2H), 2.49 (t, *J* = 7.1 Hz, 2H), 2.14 (s, 3H), 1.83–1.60 (m, 4H); **^{13}C NMR (101 MHz, Chloroform-*d*)**: δ 208.9, 200.6, 153.0, 152.8, 134.4, 132.7, 132.1, 131.5, 124.3, 114.0, 43.6, 40.6, 30.0, 29.2, 23.4, 23.0; **IR (neat)**: 3427, 2937, 2869, 1720, 1705, 1651, 1603, 1584, 1545, 1466, 1414, 1373, 1326, 1211, 1168, 1045, 1017, 933, 765, 459 cm^{-1}; **HRMS (ESI)** m/z: $[M+H]^+$ Calcd for $C_{16}H_{19}N_2O_3^+$ 287.1390; Found 287.1386.

1-(2-Methylphenanthridin-6-yl)heptane-1,6-dione (13)

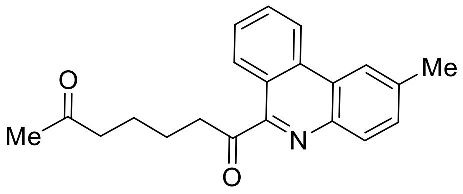

In DMF. Brown solid, 78% isolated yield (49.8 mg, 0.16 mmol). **m.p.** 112.7–114.2 °C. **^{1}H NMR (400 MHz, Chloroform-*d*)**: δ 8.79 (d, *J* = 8.3 Hz, 1H), 8.64 (d, *J* = 8.3 Hz, 1H), 8.36 (s, 1H), 8.10 (d, *J* = 8.3 Hz, 1H), 7.90–7.79 (m, 1H), 7.75–7.65 (m, 1H), 7.60 (dd, *J* = 8.3, 1.9 Hz, 1H), 3.43 (t, *J* = 7.1 Hz, 2H), 2.65 (s, 3H), 2.54 (t, *J* = 7.2 Hz, 2H), 2.16 (s, 3H), 1.90–1.71 (m, 4H); **^{13}C NMR (101 MHz, Chloroform-*d*)**: δ 209.0, 204.7, 153.5, 140.9, 139.2, 133.3, 130.9, 130.8, 130.7, 128.1, 127.8, 125.3, 123.3, 122.1, 121.8, 43.8, 40.1, 30.1, 23.8, 23.6, 22.3; **IR (neat)**: 2931, 2863, 1719, 1700, 1615, 1567, 1496,

1465, 1444, 1409, 1378, 1360, 1244, 1215, 1161, 1138, 1128, 1098, 1073, 1043, 1034, 961, 925, 824, 775, 759, 731, 726, 715, 655, 585 cm^{-1}; **HRMS (ESI)** m/z: $[M+H]^+$ Calcd for $C_{21}H_{22}NO_2^+$ 320.1645; Found 320.1639.

9-Phenylnon-8-ene-2,7-dione (15)

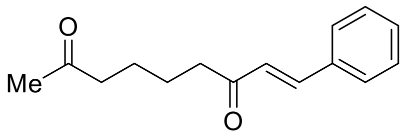

Using 5.0 equiv of **14** and 1,10-Phen was not used, in DMF at 80 °C for 24 h. White solid, 56% isolated yield (25.7 mg, 0.11 mmol). **m.p.** 66.7–67.8 °C. **^{1}H NMR (400 MHz, Chloroform-*d*)**: δ 7.61–7.49 (m, 3H), 7.45–7.37 (m, 3H), 6.73 (d, *J* = 16.2 Hz, 1H), 2.69 (t, *J* = 6.9 Hz, 2H), 2.48 (t, *J* = 6.8 Hz, 2H), 2.14 (s, 3H), 1.73–1.58 (m, 4H); **^{13}C NMR (101 MHz, Chloroform-*d*)**: δ 208.9, 200.1, 142.7, 134.6, 130.6, 129.1, 128.4, 126.3, 43.6, 40.7, 30.1, 23.8, 23.5; **IR (neat)**: 2987, 2939, 2922, 2897, 2863, 1721, 1703, 1469, 1447, 1426, 1414, 1376, 1348, 1307, 1287, 1266, 1250, 1238, 1224, 1171, 1147, 1103, 1085, 1072, 1044, 990, 792, 774, 744, 727, 688, 599, 577, 531, 424 cm^{-1}; **HRMS (ESI)** m/z: $[M+H]^+$ Calcd for $C_{15}H_{19}O_2^+$ 231.1380; Found 231.1375.

9,9-Diphenylnon-8-ene-2,7-dione (17)

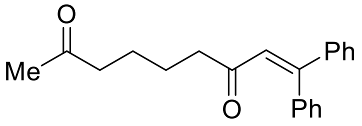

Using **1a** (0.2 mmol) and **16** (3.0 equiv), $FeSO_4 \cdot 7H_2O$ was used instead of $Fe(acac)_3$ in the absence of 1,10-Phen, in DMF at 80 °C for 4 h. Light yellow oil, 66% isolated yield (40.3 mg, 0.13 mmol). **^{1}H NMR (400 MHz, Chloroform-*d*)**: δ 7.40 (dd, *J* = 5.0, 1.9 Hz, 3H), 7.39–7.25 (m, 5H), 7.24–7.14 (m, 2H), 6.57 (s, 1H), 2.33 (t, *J* = 7.0 Hz, 2H), 2.24 (t, *J* = 7.0 Hz, 2H), 2.09 (s, 3H), 1.55–1.35 (m, 4H); **^{13}C NMR (101 MHz, Chloroform-*d*)**: δ 208.9, 202.0, 153.6, 141.0, 139.2, 129.6, 129.5, 128.7, 128.5, 128.5, 128.4, 126.7, 43.6, 42.9, 30.0, 23.8, 23.3; **IR (neat)**: 3058, 3027, 2920, 1713, 1689, 1633, 1589, 1570, 1491, 1445, 1407, 1355, 1279, 1245, 1160, 1137, 1106, 1076, 1059,

1031, 766, 698 cm^{-1}; **HRMS (ESI)** m/z: $[M+H]^+$ Calcd for $C_{21}H_{23}O_2^+$ 307.1693; Found 307.1686.

Diethyl 2-(2,7-dioxo-1-phenyloctyl)malonate (19)

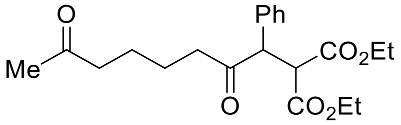

Using **1a** (0.20 mmol) and **18** (2.0 equiv), $FeSO_4 \cdot 7H_2O$ was used instead of $Fe(acac)_3$ in the absence of 1,10-Phen, in DMF at 80 °C for 4 h. Colorless oil, 43% isolated yield (32.0 mg, 0.085 mmol). **^{1}H NMR (400 MHz, Chloroform-*d*)**: δ 7.36–7.25 (m, 3H), 7.23–7.16 (m, 2H), 4.41 (d, *J* = 11.5 Hz, 1H), 4.28–4.15 (m, 3H), 3.89 (q, *J* = 7.1 Hz, 2H), 2.62–2.49 (m, 1H), 2.49–2.35 (m, 1H), 2.31 (t, *J* = 7.1 Hz, 2H), 2.07 (s, 3H), 1.61–1.35 (m, 4H), 1.27 (t, *J* = 7.1 Hz, 3H), 0.92 (t, *J* = 7.1 Hz, 3H); **^{13}C NMR (101 MHz, Chloroform-*d*)**: δ 208.8, 207.4, 168.4, 167.9, 134.1, 129.1, 129.1, 128.4, 62.0, 61.4, 57.5, 54.9, 43.4, 41.3, 29.9, 23.1, 23.0, 14.1, 13.8; **IR (neat)**: 2982, 2938, 1732, 1713, 1455, 1368, 1275, 1230, 1176, 1148, 1113, 1096, 1033, 701 cm^{-1}; **HRMS (ESI)** m/z: $[M+Na]^+$ Calcd for $C_{21}H_{28}NaO_6^+$ 399.1778; Found 399.1769.

(2) The reaction of acyl chloride 22 derived from 1a with nucleophiles

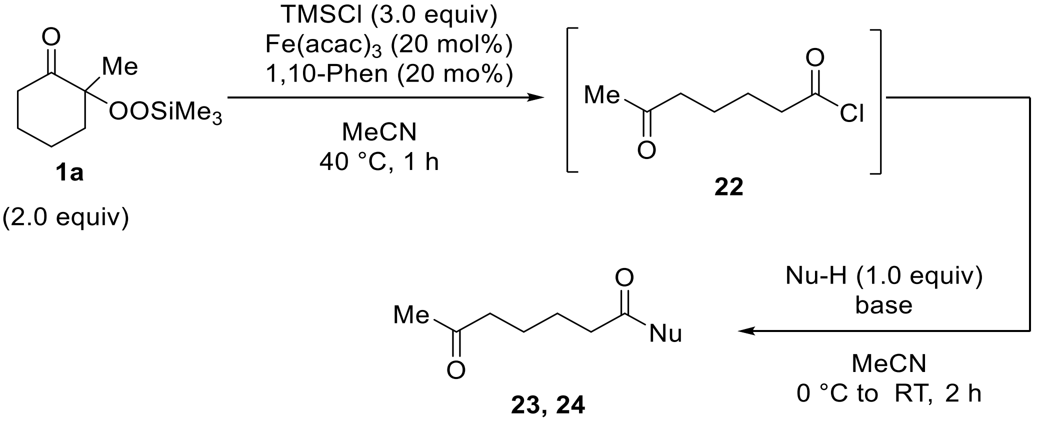

To a solution of $Fe(acac)_3$ (14.1 mg, 0.040 mmol, 20 mol%) and 1,10-Phen (7.1 mg, 0.040 mmol, 20 mol%) in dry MeCN (2.0 mL) was added TMSCl (64.8 mg, 0.60 mmol, 3.0 equiv) and α-ketoalkylsilyl peroxide **1a** (86.5 mg, 0.40 mmol) sequentially at room temperature under argon atmosphere. After stirring the mixture at 40 °C for 1 h, the

reaction mixture was cooled to 0 °C and nucleophile (0.20 mmol) and base (0.30 mmol) were added. This reaction mixture was stirred at room temperature for 2 h. The resulting mixture was quenched with water (2.0 mL) and extracted with ethyl acetate (15 mL × 3). The combined organic layer was washed with water (10 mL × 3) and then brine (10 mL). This was dried over Na_2SO_4, filtrated and concentrated under reduced pressure. The residue was purified by column chromatography on silica gel (eluted with petroleum ether/ethyl acetate = 10/1 to 2/1, gradient) to give product.

***N*-Benzyl-6-oxoheptanamide (23)**[22]

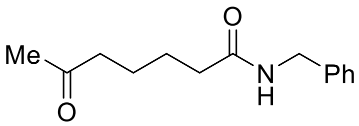

The 0.2 mmol-scale reaction: White solid, 93% isolated yield (43.4 mg, 0.19 mmol). The 1.0 mmol-scale reaction: 84% isolated yield (196 mg, 0.84 mmol).**^{1}H NMR (400 MHz, Chloroform-*d*)**: δ 7.36–7.31 (m, 2H), 7.30–7.25 (m, 3H), 5.86 (s, 1H), 4.44 (d, *J* = 5.7 Hz, 2H), 2.46 (t, *J* = 6.7 Hz, 2H), 2.23 (t, *J* = 7.0 Hz, 2H), 2.13 (s, 3H), 1.71–1.51 (m, 4H); **^{13}C NMR (101 MHz, Chloroform-*d*)**: δ 209.0, 172.6, 138.5, 128.8, 128.0, 127.6, 43.8, 43.4, 36.5, 30.1, 25.2, 23.3.

Benzyl 6-oxoheptanoate (24)[23]

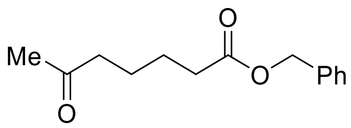

Colorless oil, 99% isolated yield (46.3 mg, 0.20 mmol). **^{1}H NMR (400 MHz, Chloroform-*d*)**: δ 7.41–7.29 (m, 5H), 5.11 (s, 2H), 2.43 (t, *J* = 6.8 Hz, 2H), 2.37 (t, *J* = 7.1 Hz, 2H), 2.12 (s, 3H), 1.67–1.55 (m, 4H); **^{13}C NMR (101 MHz, Chloroform-*d*)**: δ 208.6, 173.3, 136.1, 128.7, 128.3, 66.3, 43.4, 34.2, 30.0, 24.5, 23.3.

(3) Fridel-Crafts acylation of acyl chloride 22 derived from 1a

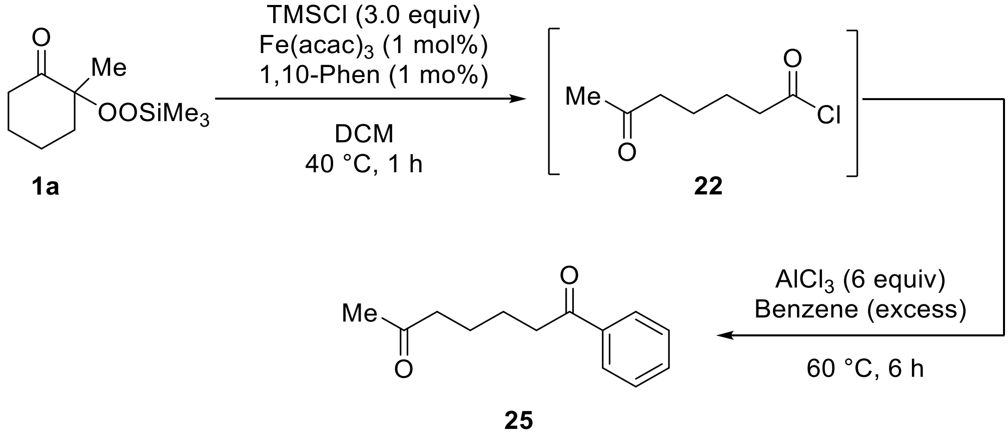

To a solution of $Fe(acac)_3$ (0.7 mg, 0.002 mmol, 1 mol%) and 1,10-Phen (0.4 mg, 0.002 mmol, 1 mol%) in dry DCM (1.0 mL) was added TMSCl (64.8 mg, 0.60 mmol, 3 equiv) and α-ketoalkyllsilyl peroxide **1a** (43.2 mg, 0.20 mmol) sequentially at room temperature under argon atmosphere. After stirring the mixture at 40 °C for 1 h, volatiles were removed under reduced pressure (3.0 Torr) at room temperature. Dry benzene (3.0 mL) was added to the residue and the mixture was passed through the membrane filer (PTFE syringe filter, pore size: 0.45 μm). Then, $AlCl_3$ (160 mg, 1.2 mmol) was added to the filtrate and the mixture was stirred at 60 °C for 6 h under argon atmosphere. The resulting mixture was diluted with DCM (50 mL) and washed with 1 N HCl aq. (20 mL× 3) and brine (20 mL). The organic layer was dried over Na_2SO_4, filtrated and concentrated under reduced pressure. The residue was purified by column chromatography on silica gel (eluted with DCM) to give product **25**.

1-Phenylheptane-1,6-dione (25)[24]

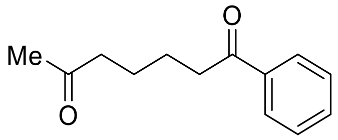

Light yellow oil, 57% isolated yield (23.2 mg, 0.11 mmol). **^{1}H NMR (400 MHz, Methanol-*d*$_4$)** δ 8.04–7.96 (m, 2H), 7.65–7.56 (m, 1H), 7.55–7.46 (m, 2H), 3.05 (t, *J* = 7.0 Hz, 2H), 2.55 (t, *J* = 6.9 Hz, 2H), 2.15 (s, 3H), 1.77–1.59 (m, 4H); **^{13}C NMR (101**

MHz, Methanol-d_4): δ 211.8, 202.4, 138.3 134.2, 129.7, 129.1, 44.1, 39.2, 29.8, 24.9, 24.4.

(4) Radical trapping experiment of 1a with TEMPO

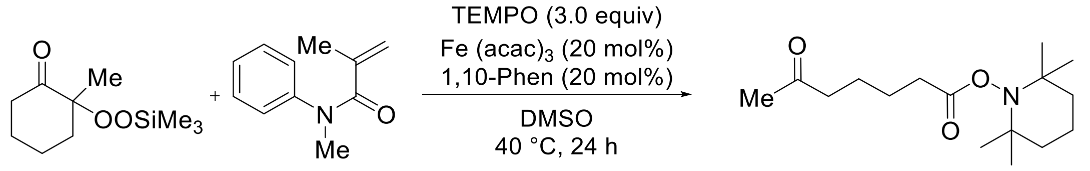

1a (2.0 equiv) **7** **26**

To the solution of **7** (35.0 mg, 0.20 mmol), Fe(acac)$_3$ (14.1 mg, 0.04 mmol, 20 mol%), 1,10-Phen (7.2 mg, 0.04 mmol, 20 mol%) and 2,2,6,6-tetramethylpiperidine 1-oxyl (TEMPO, 93.8 mg, 0.60 mmol, 3 equiv) in dry DMSO (1.0 mL) was added α-ketoalkylsilyl peroxide **1a** (86.5 mg, 0.40 mmol, 2 equiv) dropwise at room temperature under argon atmosphere. The reaction mixture was stirred at 40 °C for 24 h. After being cooled to room temperature, the reaction mixture was quenched with water (2.0 mL) and extracted with ethyl acetate (15 mL × 3). The organic layer was washed with water (10 mL × 3) and then brine (10 mL). This was dried over Na_2SO_4, filtrated and concentrated under reduced pressure. The residue was analyzed by ^{1}H NMR to determine the yields of products using nitromethane as an internal standard. The yield of TEMPO adducts **26** was estimated to be 40% by ^{1}H NMR spectroscopy without the detection of **10a**. This product **26** was partially isolated as by column chromatography on silica gel (eluted with ethyl acetate/petroleum ether = 1/30 to 1/10, gradient).

2,2,6,6-Tetramethylpiperidin-1-yl 6-oxoheptanoate (26)

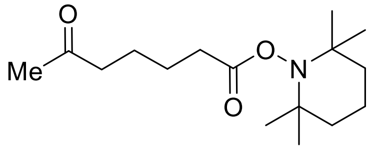

^{1}H NMR (400 MHz, Methanol-d_4): δ 2.56 (t, *J* = 6.8 Hz, 2H), 2.43 (t, *J* = 6.8 Hz, 2H), 2.16 (s, 3H), 1.77–1.62 (m, 7H), 1.61–1.54 (m, 2H), 1.48–1.39 (m, 1H), 1.21 (s, 6H), 1.04 (s, 6H); **^{13}C NMR (101 MHz, Methanol-d_4)**: δ 211.4, 175.6, 61.3, 43.7, 39.9, 33.2,

32.1, 29.8, 25.6, 24.4, 21.0, 17.9; **IR (neat)**: 3480, 2973, 2936, 2872, 2563, 1764, 1716, 1453, 1422, 1378, 1364, 1265, 1247, 1234, 1209, 1165, 1123, 1083, 1046, 954, 934 cm^{-1}; **HRMS (ESI)** m/z: $[M+H]^+$ Calcd for $C_{16}H_{30}NO_3^+$ 284.2220; Found 284.2214.

(5) Metal-catalyzed reactions of β-acetoxy, and β-amidoalkylsilyl peroxides

Iron-catalyzed azidation of β-acetoxyalkylsilyl peroxide

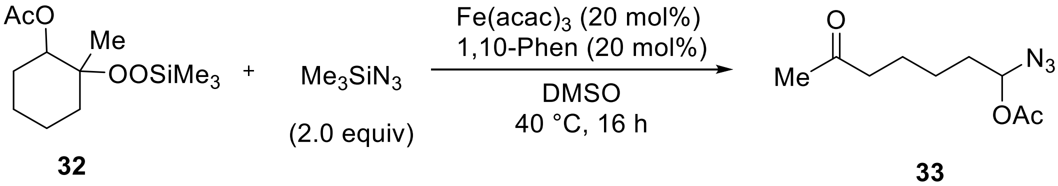

To a solution of $Fe(acac)_3$ (14.1 mg, 0.04 mmol, 20 mol%) and 1,10-Phen (7.2 mg, 0.04 mmol, 20 mol%) in dry DMSO (1.0 mL) was added β-acetoxyalkylsilyl peroxide **32** (60.4 mg, 0.2 mmol) and trimethylsilyl azide (46.1 mg, 0.4 mmol, 2.0 equiv) sequentially at room temperature under argon atmosphere. The reaction mixture was stirred at 40 °C for 16 h. The reaction mixture was quenched with water (2.0 mL) and extracted with ethyl acetate (15 mL × 3). The combined organic layer was washed with water (10 mL × 3), K_2CO_3 (10 mL) aq. and brine (10 mL), dried over Na_2SO_4, filtrated and concentrated under reduce pressure. The residue was purified by column chromatography on silica gel (eluted with petroleum ether/ethyl acetate=10/1) to afford product **33**.

1-Azido-6-oxoheptyl acetate (33)

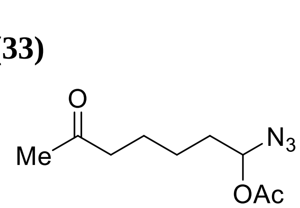

Colorless oil, 86% isolated yield (36.6 mg, 0.17 mmol). **^{1}H NMR (400 MHz, Chloroform-*d*)**: δ 5.84 (t, *J* = 6.2 Hz, 1H), 2.44 (t, *J* = 7.2 Hz, 2H), 2.13 (s, 3H), 2.12 (s, 3H), 1.74–1.66 (m, 2H), 1.63–1.55 (m, 2H), 1.43–1.33 (m, 2H); **^{13}C NMR (101 MHz, Chloroform-*d*)**: δ 208.5, 170.6, 85.1, 43.4, 33.6, 30.1, 23.9, 23.2, 21.0; **IR (neat)**: 2954, 2926, 2869, 2112, 1747, 1714, 1462, 1435, 1372, 1256, 1210, 1162, 1087, 1048,

1017, 936, 800, 725, 636, 600, 563, 492, 405 cm^{-1}; **HRMS:** m/z: $[M+Na]^+$ Calcd for $C_9H_{15}N_3NaO_3^+$ 236.1006; Found 236.1007.

Copper-catalyzed cyanation of β-amidoalkylsilyl peroxide

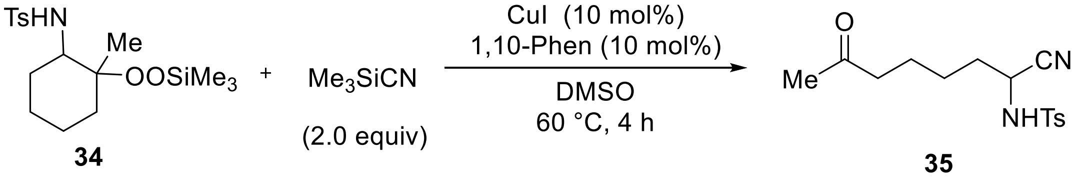

To a solution of copper iodide (3.8 mg, 0.02 mmol, 10 mol%), 1,10-Phen (3.6 mg, 0.02 mmol, 10 mol%) and β-amidoalkylsilyl peroxide **34** (82.7 mg, 0.2 mmol) in dry DMF (2.0 mL) was added trimethylsilyl cyanide (39.7 mg, 0.4 mmol, 2.0 equiv) at room temperature under argon atmosphere. The reaction mixture was stirred at 60 °C for 4 h. The reaction mixture was quenched with water (2.0 mL) and extracted with ethyl acetate (15 mL × 3). The combined organic layer was washed with water (10 mL × 3) and brine (10 mL), dried over Na_2SO_4, filtrated and concentrated under reduce pressure. The residue was purified by column chromatography on silica gel (eluted with petroleum ether/ethyl acetate=5/1 to 2/1) to afford product **35**.

***N*-(1-Cyano-6-oxoheptyl)-4-methylbenzenesulfonamide (35)**

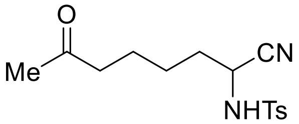

Colorless oil, 88% isolated yield (54.1 mg, 0.18 mmol). **^{1}H NMR (400 MHz, Chloroform-*d*)**: δ 7.77 (d, *J* = 8.4 Hz, 2H), 7.34 (d, *J* = 8.0 Hz, 2H), 5.75 (d, *J* = 9.5 Hz, 1H), 4.20 (dt, *J* = 9.5, 7.1 Hz, 1H), 2.46–2.40 (m, 5H), 2.13 (s, 3H), 1.84–1.76 (m, 2H), 1.61–1.51 (m, 2H), 1.50–1.38 (m, 2H); **^{13}C NMR (101 MHz, Chloroform-*d*)**: δ 208.9, 144.6, 136.2, 130.1, 127.3, 117.6, 44.3, 43.1, 33.8, 30.1, 24.6, 22.6, 21.8; **IR (neat)**: 3251, 2927, 2869, 1704, 1598, 1453, 1337, 1307, 1291, 1266, 1185, 1158, 1091, 1038, 1019, 908, 815, 734, 705, 665, 577, 544 cm^{-1}; **HRMS:** m/z: $[M+Na]^+$ Calcd for $C_{15}H_{20}N_2NaO_3S^+$ 331.1087; Found 331.1086.

(6) Visible light-promoted alkylation of phenyl vinyl sulfone

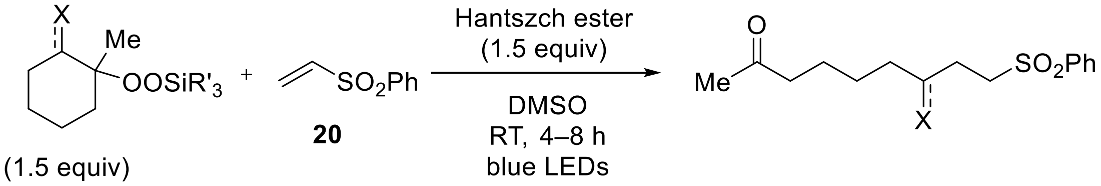

To a solution of phenyl vinyl sulfone **20** (16.8 mg, 0.10 mmol) and diethyl 1,4-dihydro-2,6-dimethyl-3,5-pyridinedicarboxylate (Hantzsch ester, 37.4 mg, 0.15 mmol, 1.5 equiv) in dry DMSO (0.5 mL) was added alkylsilyl peroxide **1a**, **32** or **34** (0.15 mmol, 1.5 equiv) at room temperature under argon atmosphere. The mixture was stirred at room temperature for 4–8 h under irradiation with 405 nm blue LED (Benstartech, BX-UV-COB-405, 6 W, approximately 1 cm away from the reaction tube). The reaction mixture was quenched with water (2.0 mL) and extracted with ethyl acetate (10 mL × 3). The organic layer was washed with water (10 mL × 3) and then brine (10 mL). This was dried over Na_2SO_4, filtrated and concentrated under reduced pressure. The residue was then purified by column chromatography on silica gel (eluted with petroleum ether/ethyl acetate) to afford the corresponding product **21**, **36** or **37**, respectively.

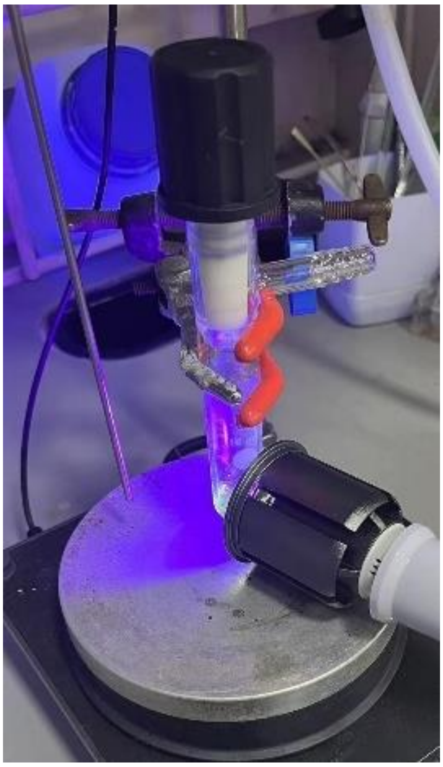

Figure S1. Reaction setup.

9-(Phenylsulfonyl)nonane-2,7-dione (21)

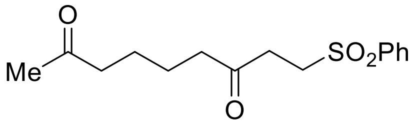

White solid, 93% isolated yield (27.6 mg, 0.093 mmol). **m.p.** 79.1-80.0 °C. **^{1}H NMR (400 MHz, Chloroform-*d*)**: δ 7.95–7.87 (m, 2H), 7.72–7.63 (m, 1H), 7.63–7.53 (m, 2H), 3.47–3.30 (m, 2H), 2.93–2.84 (m, 2H), 2.51–2.37 (m, 4H), 2.12 (s, 3H), 1.59–1.47 (m, 4H); **^{13}C NMR (101 MHz, Chloroform-d)**: δ 208.6, 205.8, 139.1, 134.1, 129.6, 128.1, 50.7, 43.4, 42.7, 35.1, 30.1, 23.1, 23.1; **IR (neat)**: 2944, 2919, 2888, 1704, 1683, 1607, 1576, 1497, 1467, 1450, 1412, 1374, 1338, 1305, 1279, 1248, 1233, 1161, 1107, 1078, 1064, 1054, 1018, 998, 989, 966, 764, 744, 695, 565, 480 cm^{-1}; **HRMS (ESI)** m/z: $[M+Na]^+$ Calcd for $C_{15}H_{20}NaO_4S^+$ 319.0975; Found 319.0968.

8-Oxo-1-(phenylsulfonyl)nonan-3-yl acetate (36)

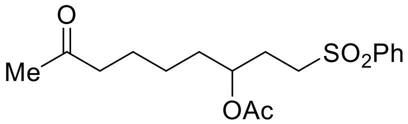

Light yellow oil, 87% isolated yield (29.5 mg, 0.087 mmol). **^{1}H NMR (400 MHz, Chloroform-*d*)**: δ 7.93–7.88 (m, 2H), 7.70–7.64 (m, 1H), 7.62–7.54 (m, 2H), 4.93–4.78 (m, 1H), 3.15–3.02 (m, 3H), 2.46–2.32 (m, 2H), 2.11 (s, 3H), 2.06–1.89 (m, 5H), 1.61–1.44 (m, 3H), 1.28–1.22 (m, 2H); **^{13}C NMR (101 MHz, Chloroform-*d*)**: δ 208.7, 170.7, 139.0, 134.0, 129.5, 128.2, 71.9, 52.8, 43.4, 34.0, 30.1, 27.2, 24.8, 23.4, 21.2; **IR (neat)**: 3059, 2927, 2855, 1733, 1714, 1447, 1372, 1307, 1266, 1239, 1150, 1086, 1025, 956, 797, 732, 702, 689, 594, 566, 539 cm^{-1}; **HRMS (ESI)** m/z: $[M+Na]^+$ Calcd for $C_{17}H_{24}NaO_5S^+$ 363.1237; Found 363.1231.

4-Methyl-*N*-(8-oxo-1-(phenylsulfonyl)nonan-3-yl)benzenesulfonamide (37)

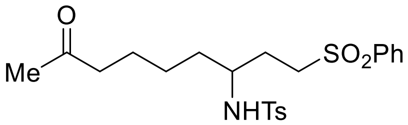

Light yellow oil, 64% isolated yield (28.8 mg, 0.064 mmol). **^{1}H NMR (400 MHz, Chloroform-*d*)**: δ 7.90–7.83 (m, 2H), 7.73–7.62 (m, 3H), 7.61–7.52 (m, 2H), 7.26 (d, *J* = 8.1 Hz, 2H), 4.72 (d, *J* = 8.8 Hz, 1H), 3.34–3.19 (m, 1H), 3.20–2.98 (m, 2H), 2.40 (s, 3H), 2.24 (t, *J* = 7.3 Hz, 2H), 2.07 (s, 3H), 2.03–1.89 (m, 1H), 1.81–1.69 (m, 1H), 1.44–1.15 (m, 4H), 1.12–0.91 (m, 2H); **^{13}C NMR (101 MHz, Chloroform-*d*):** δ 208.7, 143.7, 139.2, 138.0, 133.9, 129.9, 129.5, 128.1, 127.1, 53.0, 53.0, 43.2, 35.5, 30.0, 28.0, 24.9, 23.2, 21.7; **IR (neat)**: 3276, 2933, 2255, 1709, 1598, 1447, 1305, 1151, 1086, 907, 815, 726, 688, 663, 648, 598, 578, 549 cm^{-1}; **HRMS (ESI)** m/z: $[M+Na]^+$ Calcd for $C_{22}H_{29}NNaO_5S_2^+$ 474.1379; Found 474.1373.

5. References

(1) W.-B. Du, N.-N. Wang, C. Pan, S.-F. Ni, L.-R. Wen, M. Li and L.-B. Zhang, *Green Chem.*, 2021**, 23**, 2420–2426.

(2) A. F. Garrido-Castro, A. Gini, M. C. Maestro and J. Alemán, *Chem. Commun.*, 2020, **56**, 3769–3772.

(3) M. Tobisu, K. Koh, T. Furukawa and N. Chatani, *Angew. Chem., Int. Ed.*, 2012**, 51**, 11363–11366.

(4) X.-T. Wang, W.-B. Han, H.-J. Chen, Q. Zha and Y. Wu, *J. Org. Chem.* 2020, **85**, 10007–10021.

(5) R. C. P. Cubbon and C. Hewlett, *J. Chem. Soc. C*, 1968, 2978–2982.

(6) J. D. Druliner, F. W. Hobbs and W. C. Seide, *J. Org. Chem.*, 1988, **53**, 700–702.

(7) A. Riahi, J. Muzart, M. Abe and N. Hoffmann, *New J. Chem.*, 2013, **37**, 2245–2249.

(8) A. S. K. Tsang, A. Kapat and F. Schoenebeck, *J. Am. Chem. Soc.*, 2016, **138**, 518–526.

(9) T. Tokuyasu, S. Kunikawa, M. Abe, A. Masuyama, M. Nojima, H.-S. Kim, K. Begum and Y. Wataya, *J. Org. Chem.*, 2003, **68**, 7361–7367.

(10) T. Suga, T. Shishibori and T. Matsuura, *J. Org. Chem.*, 1967, **32**, 965–969.

(11) S. Tranchimand, B. Faure, J.-V. Naubron, V. Alphand, A. Archelas and G. Iacazio, *Eur. J. Org. Chem.*, 2012**, 2012**, 4365–4372.

(12) T. Suga, T. Hirata, H. Hamada and S. Murakami, *Phytochemistry*, 1988, **27**, 1041–1044.

(13) S. Sakurai, T. Kato, R. Sakamoto and K. Maruoka, *Tetrahedron* 2019, **75**, 172–179.

(14) Y. Li, H.-D. Hao and Y. Wu, *Org. Lett.* 2009, **11**, 2691–2694.

(15) P. A. Allegretti and E. M. Ferreira, *J. Am. Chem. Soc.* 2013, **135**, 17266–17269.

(16) SK A. Saleh, A. Hazra and S. Hajra, *Adv. Synth. Catal.*, 2022, **364**, 391–404.

(17) J. U. Jeong, B. Tao, I. Sagasser, H. Henniges and K. B. Sharpless, *J. Am. Chem. Soc.* 1998, **120**, 6844–6845.

(18) X.-H. Ouyang, R.-J. Song and J.-H. Li, *Eur. J. Org. Chem.*, 2014, **2014**, 3395–3401.

(19) M.-Z. Zhang, P.-Y. Ji, Y.-F. Liu and C.-C. Guo, *J. Org. Chem.*, 2015, **80**, 10777–10786.

(20) X. Fan, T. Lei, B. Chen, C.-H. Tung and L.-Z. Wu, *Org. Lett.*, 2019, **21**, 4153–4158.

(21) L. Yang, W. Lu, W. Zhou and F. Zhang, *Green Chem.*, 2016, **18**, 2941–2945.

(22) M. A. Hussein, V. T. Huynh, R. Hommelsheim, R. M. Koenigs and T. V. Nguyen, *Chem. Commun.*, 2018, **54**, 12970–12973.

(23) R. R. Huddleston and M. J. Krische, *Org. Lett.*, 2003, **5**, 1143–1146.

(24) E. P. Balskus, J. Méndez-Andino, R. M. Arbit, and L. A. Paquette. *J. Org. Chem.* 2001, **66**, 6695–6704.

6. ^{1}H NMR and ^{13}C NMR Spectra

2-Methyl-2-((trimethylsilyl)peroxy)cyclohexan-1-one (1a)

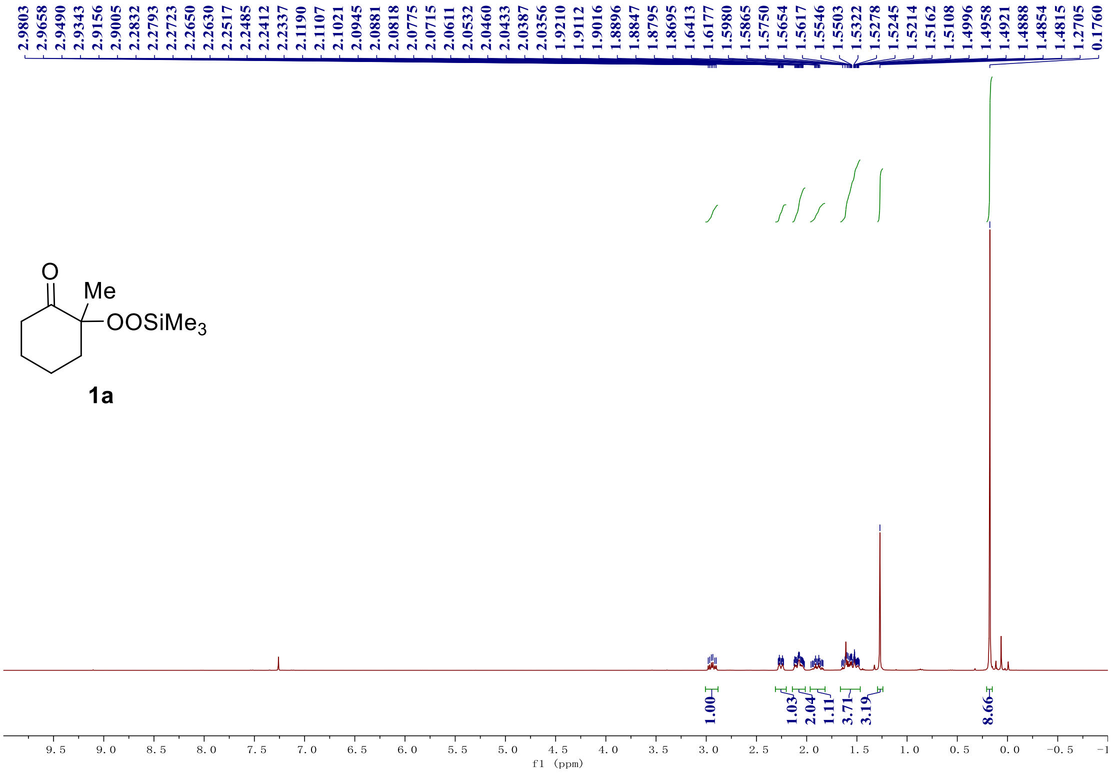

^{1}H NMR (400 MHz, $CDCl_3$) spectrum of compound **1a**

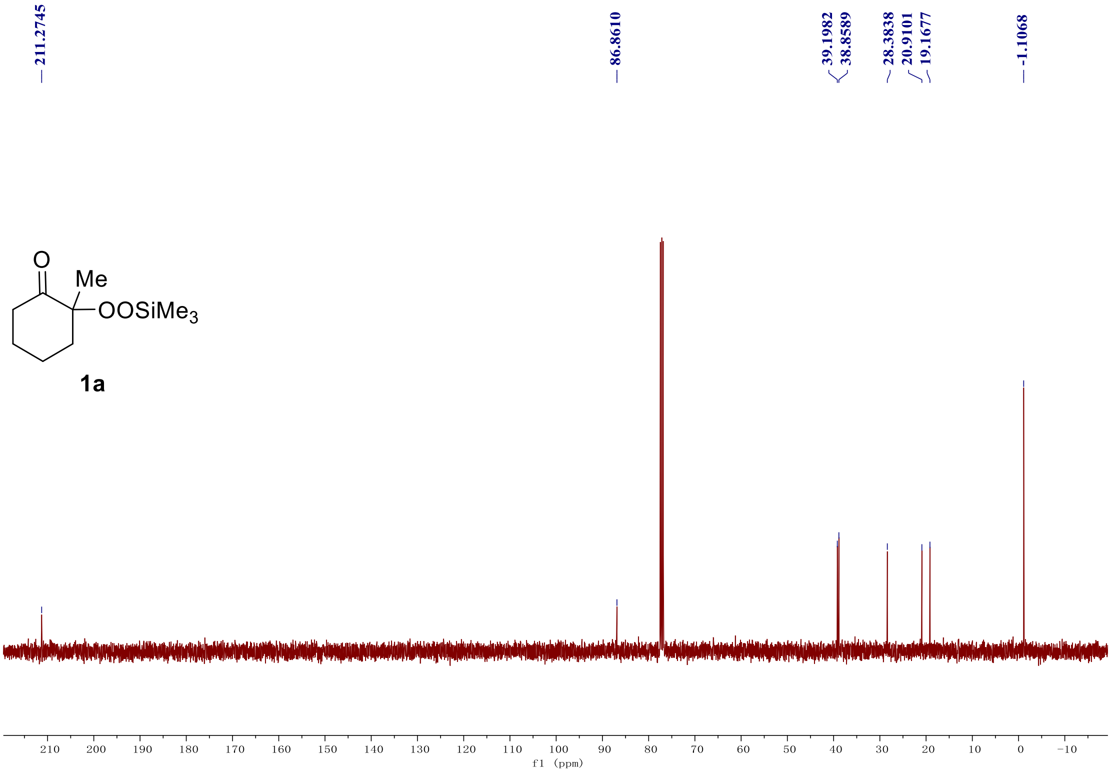

^{13}C NMR (101 MHz, $CDCl_3$) spectrum of compound **1a**

2-Methyl-2-((trimethylsilyl)peroxy)cyclopentan-1-one (1b)

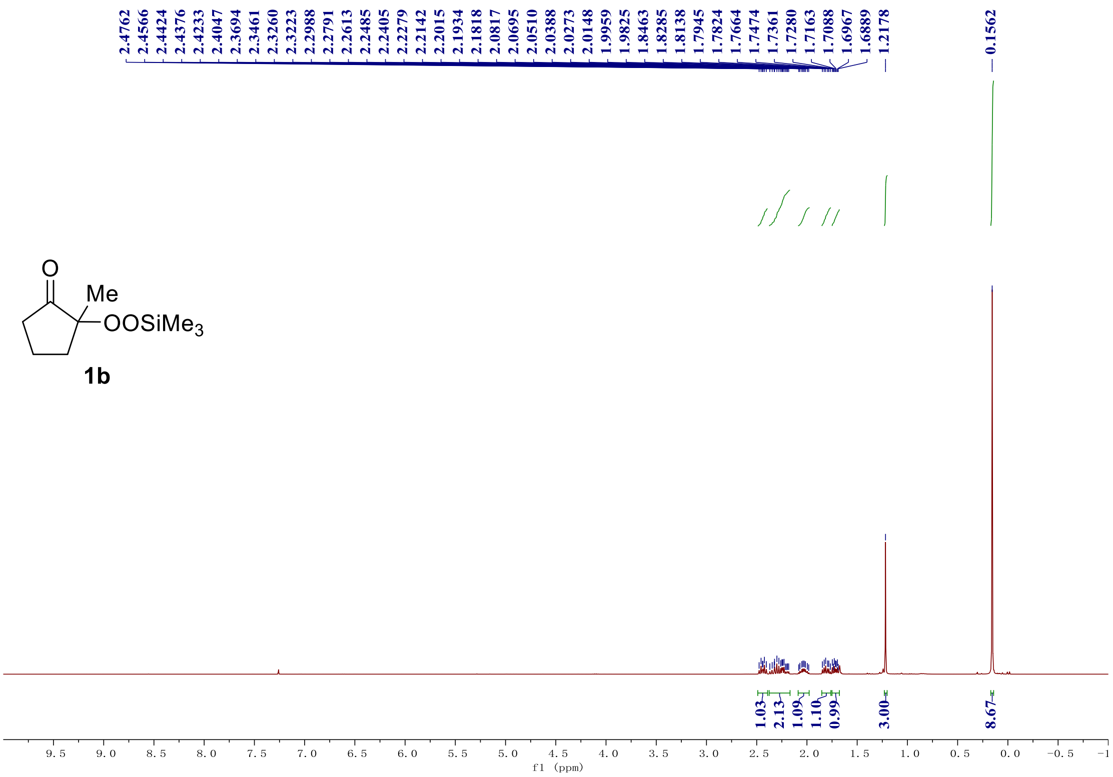

^{1}H NMR (400 MHz, $CDCl_3$) spectrum of compound **1b**

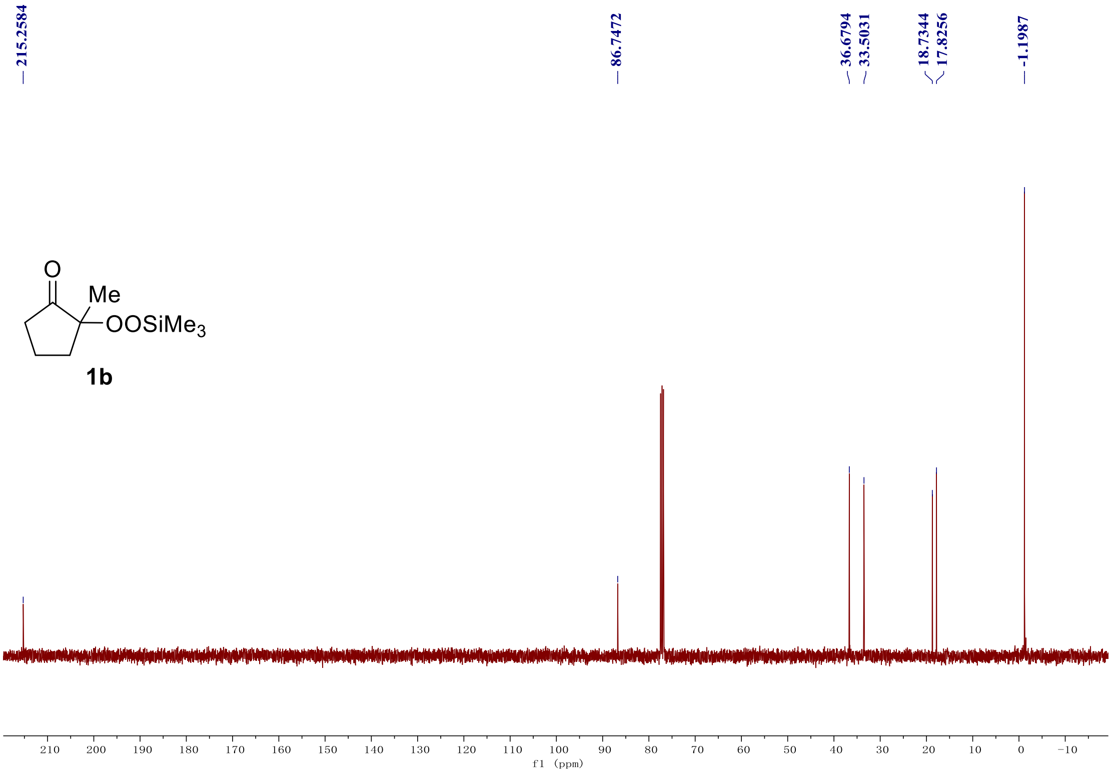

^{13}C NMR (101 MHz, $CDCl_3$) spectrum of compound **1b**

2-Methyl-2-((trimethylsilyl)peroxy)cycloheptan-1-one (1c)

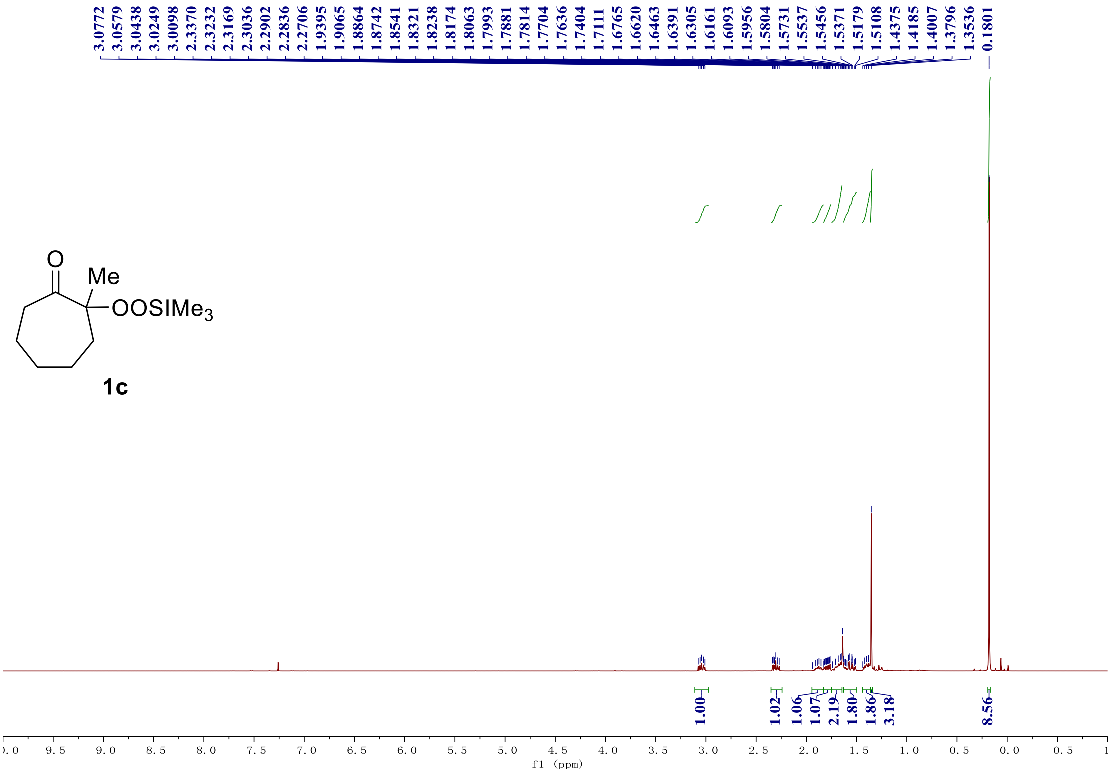

^{1}H NMR (400 MHz, $CDCl_3$) spectrum of compound **1c**

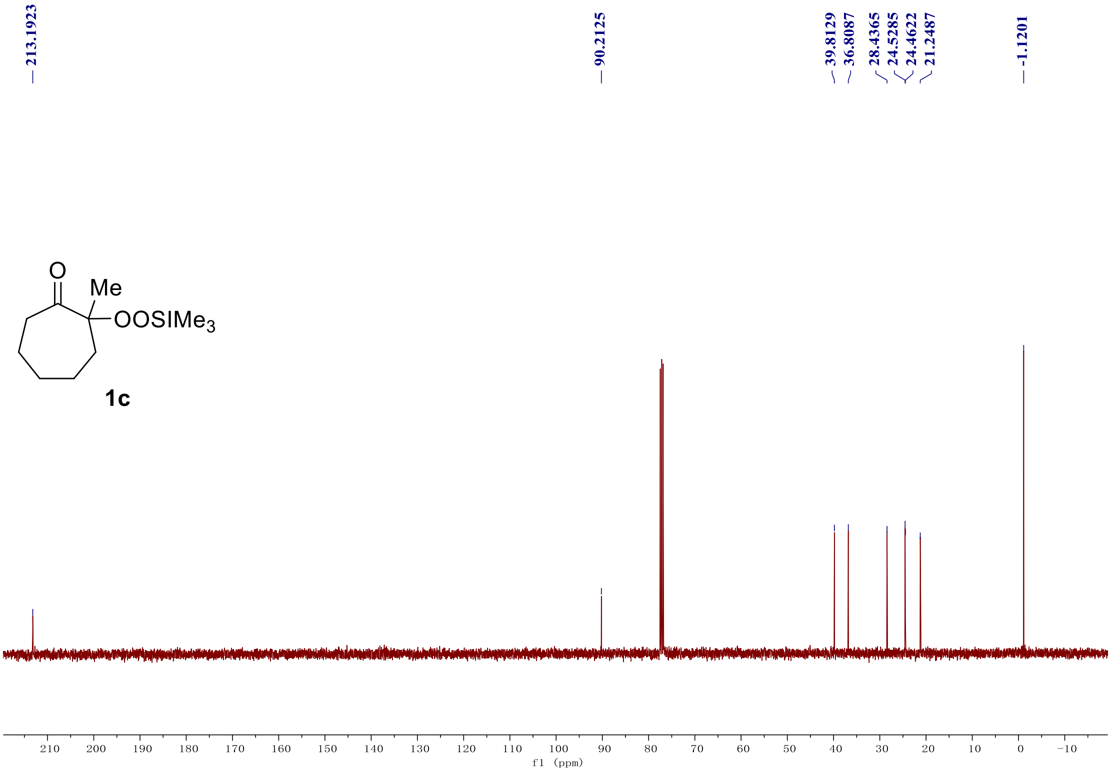

^{13}C NMR (101 MHz, $CDCl_3$) spectrum of compound **1c**

2-Methyl-2-((trimethylsilyl)peroxy)cyclooctan-1-one (1d)

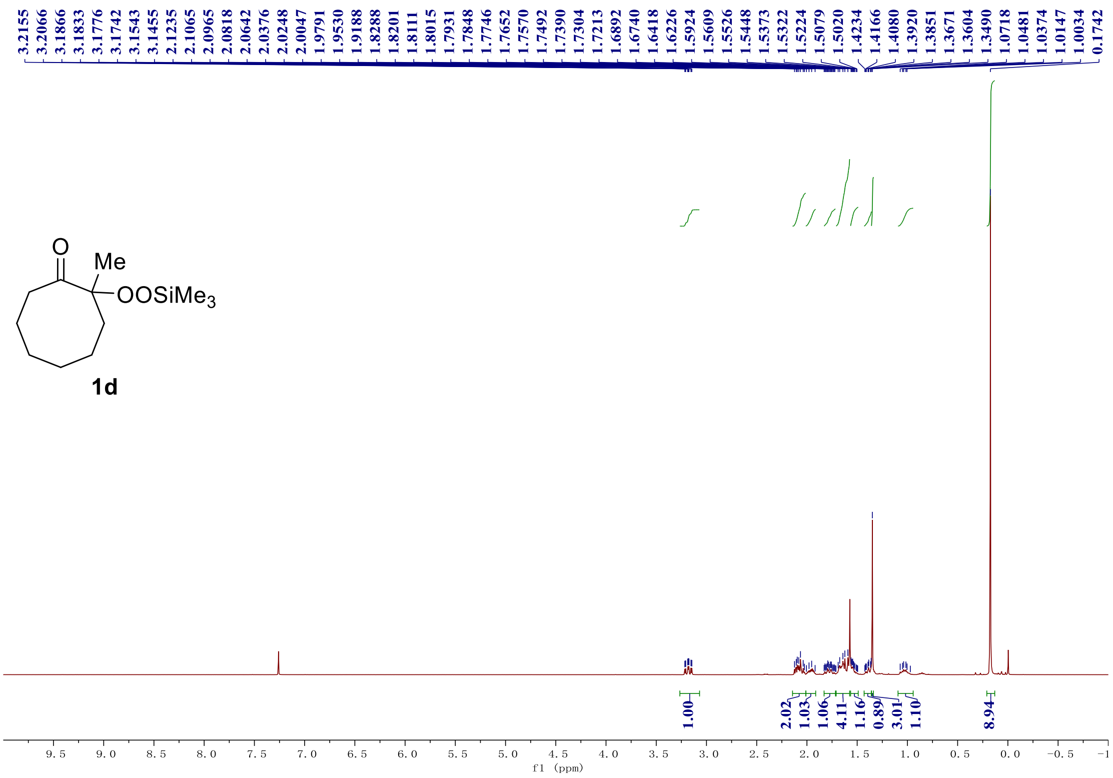

^1H NMR (400 MHz, $CDCl_3$) spectrum of compound **1d**

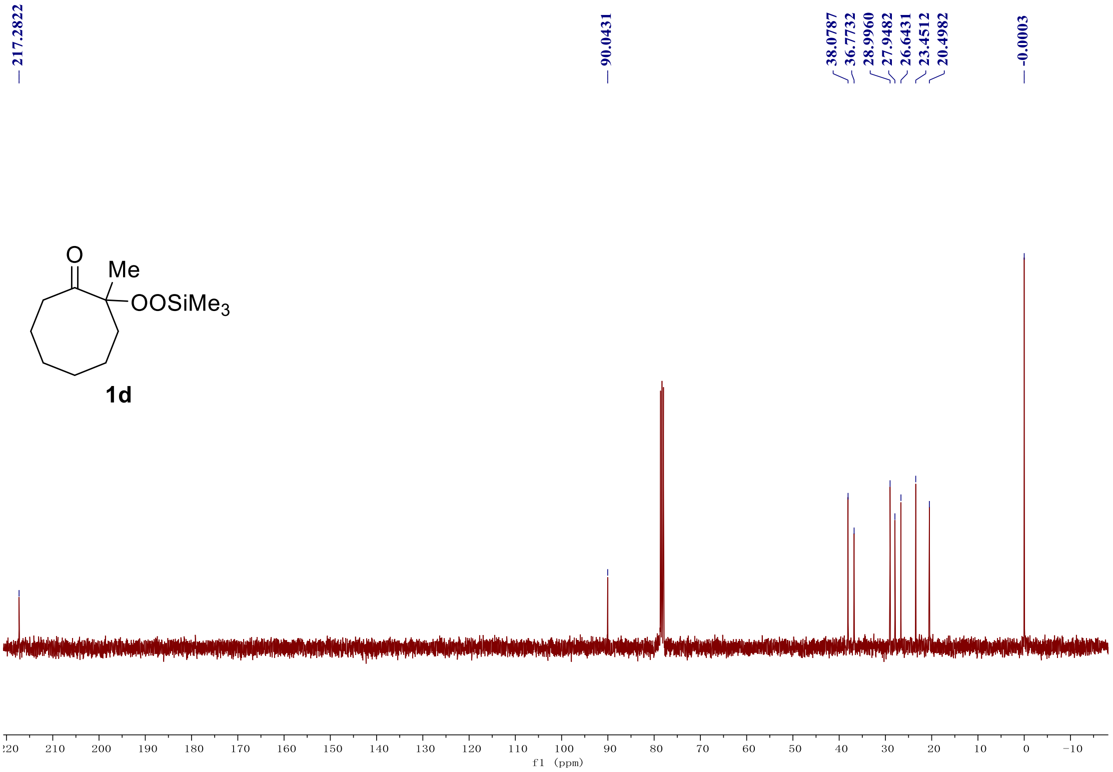

^{13}C NMR (101 MHz, $CDCl_3$) spectrum of compound **1d**

2-Methyl-2-((trimethylsilyl)peroxy)-3,4-dihydronaphthalen-1(2H)-one (1e)

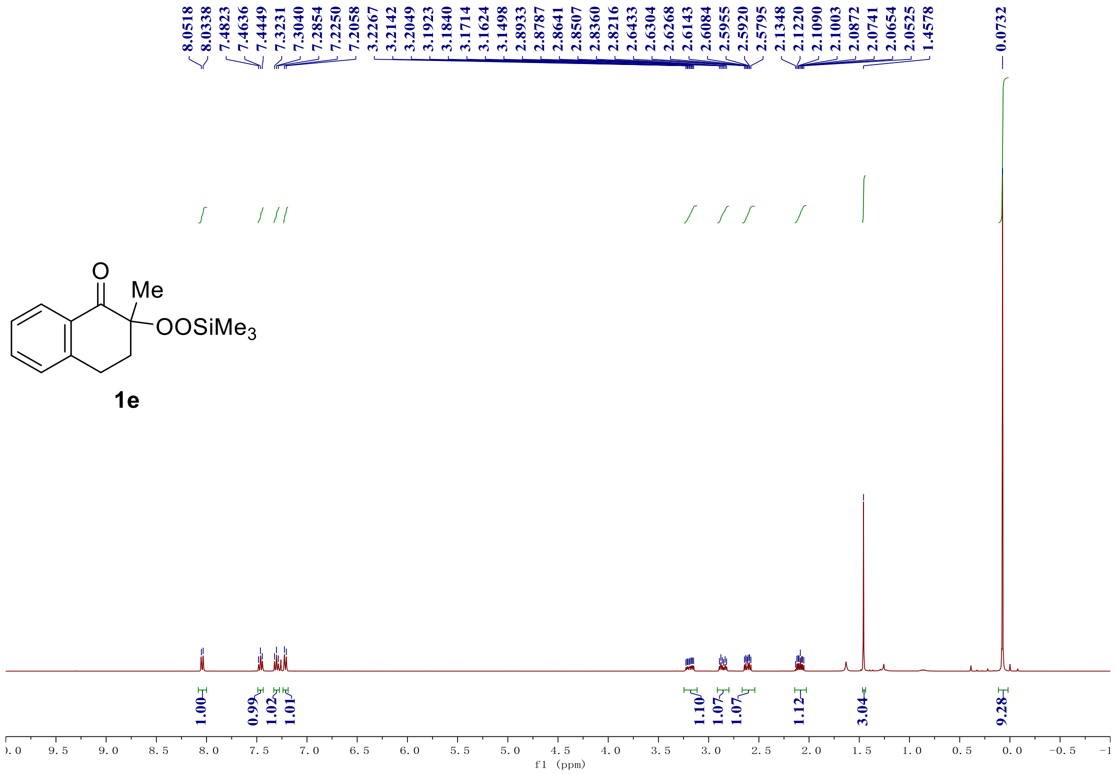

^{1}H NMR (400 MHz, $CDCl_3$) spectrum of compound **1e**

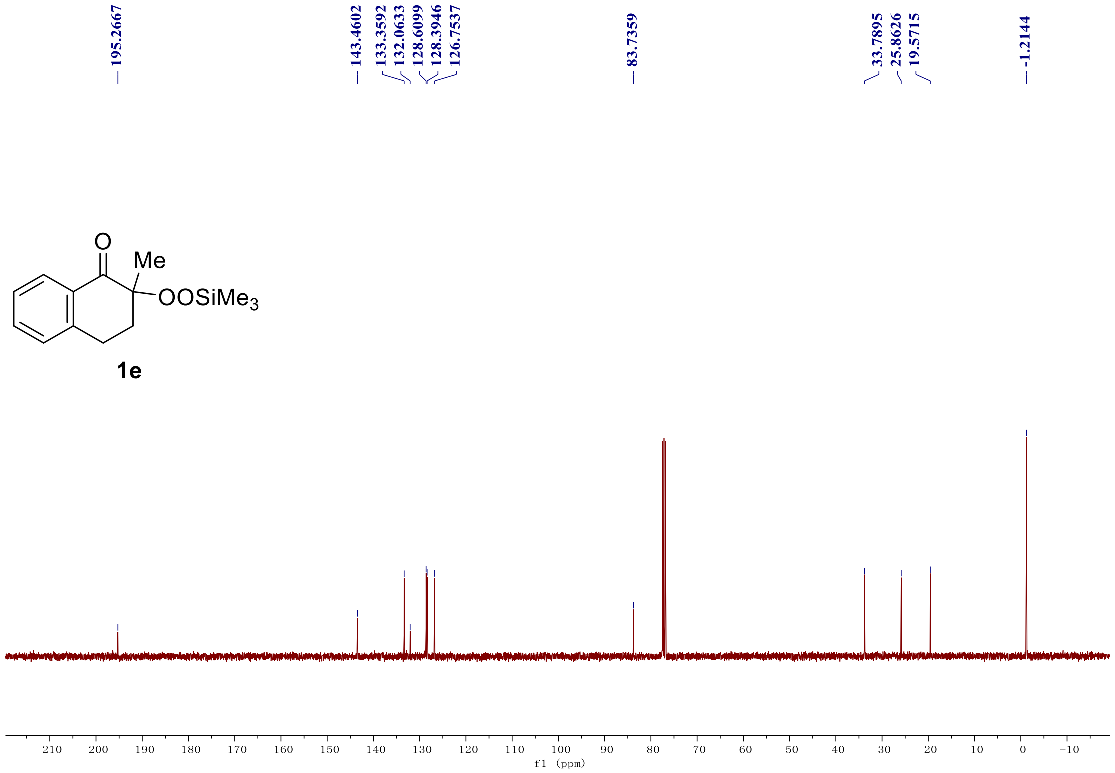

^{13}C NMR (101 MHz, $CDCl_3$) spectrum of compound **1e**

2-Methyl-2-((trimethylsilyl)peroxy)pentan-3-one (1f)

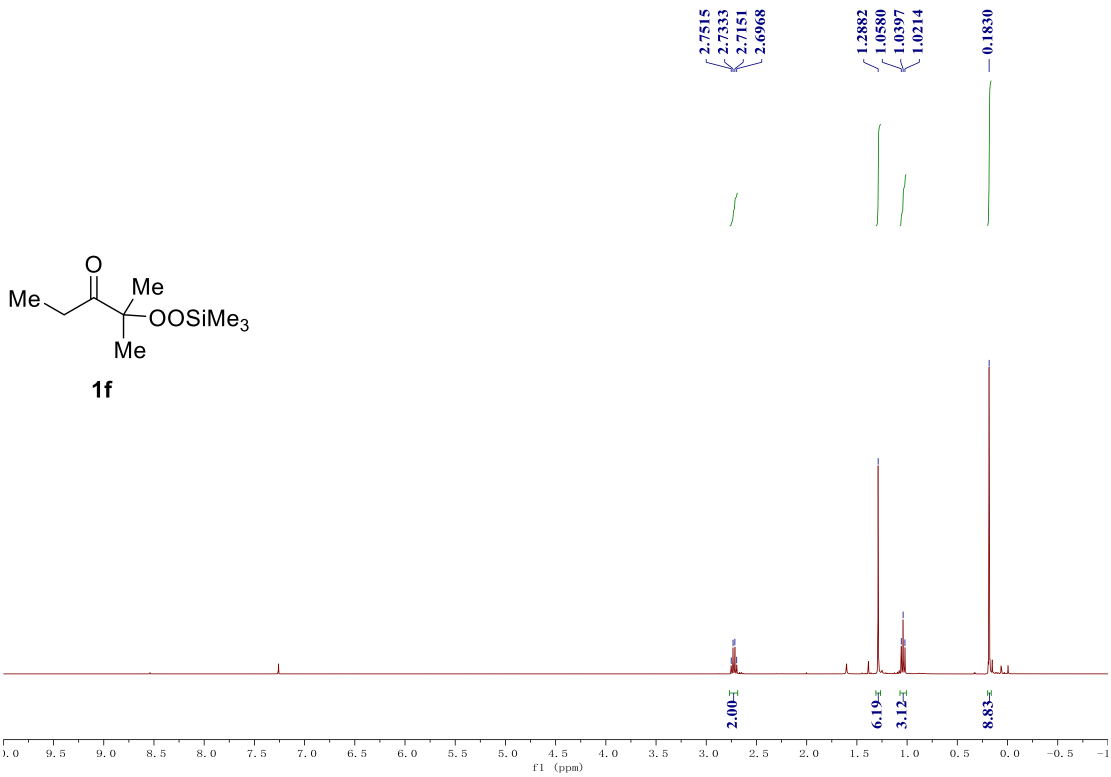

^{1}H NMR (400 MHz, $CDCl_3$) spectrum of compound **1f**

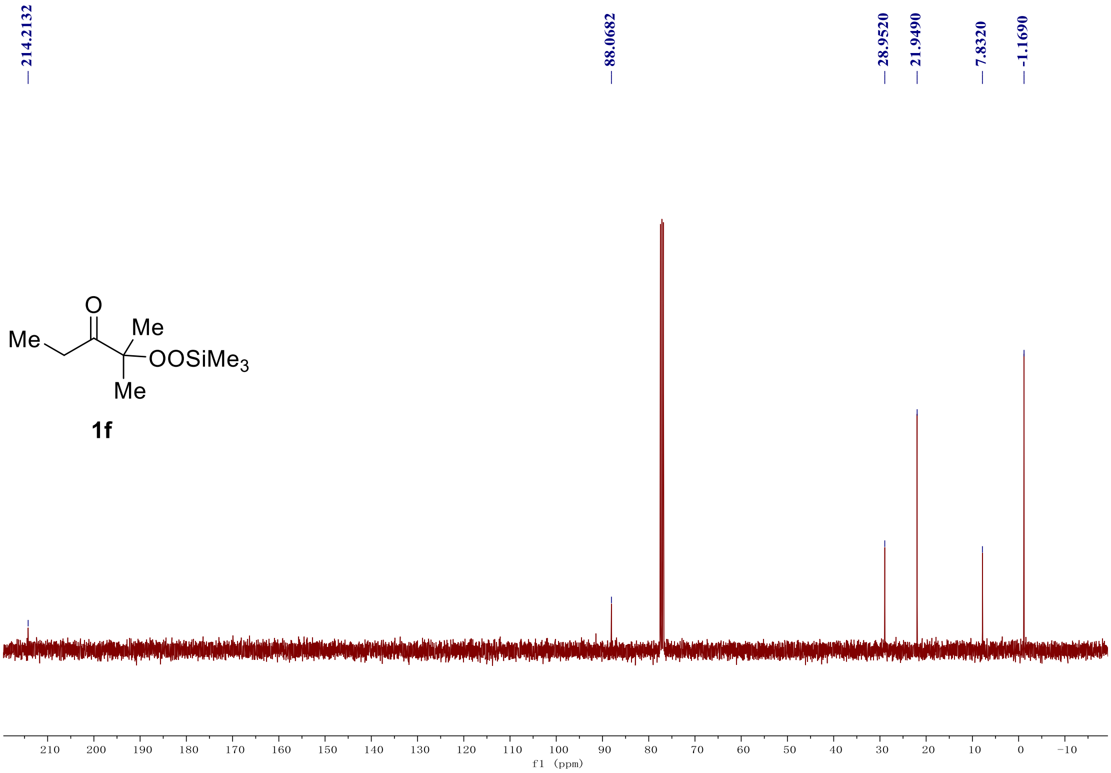

^{13}C NMR (101 MHz, $CDCl_3$) spectrum of compound **1f**

2,4-Dimethyl-2-((trimethylsilyl)peroxy)pentan-3-one (1g)

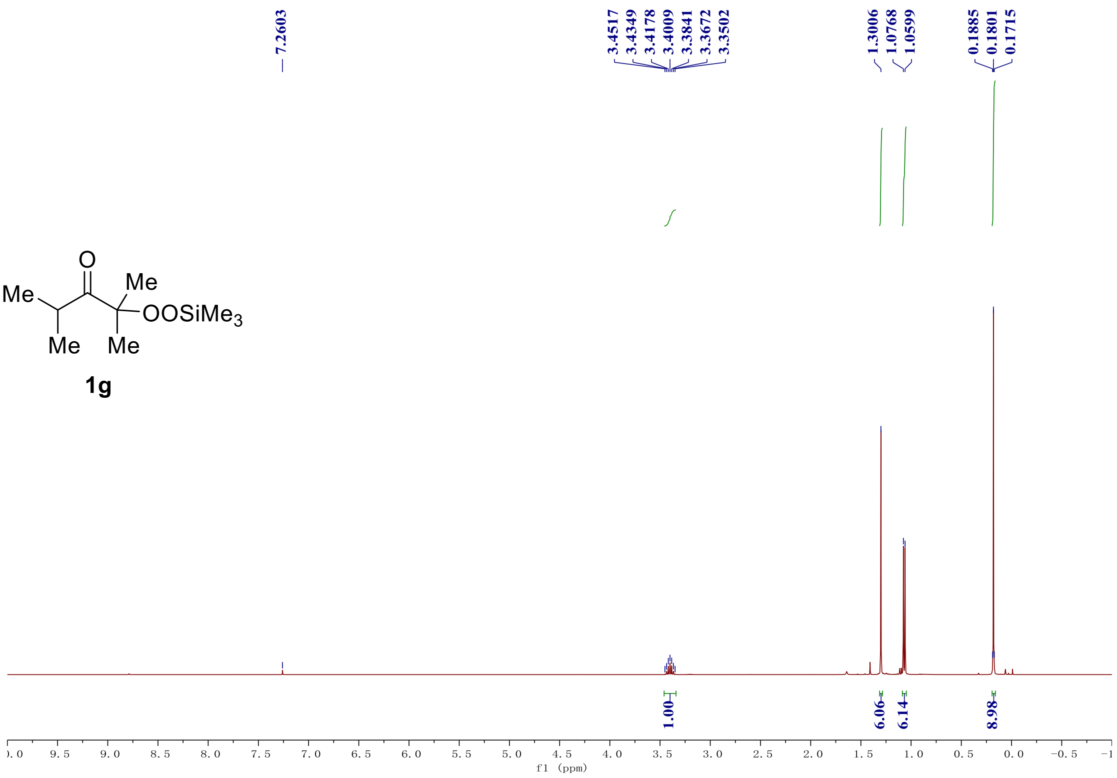

^{1}H NMR (400 MHz, $CDCl_3$) spectrum of compound **1g**

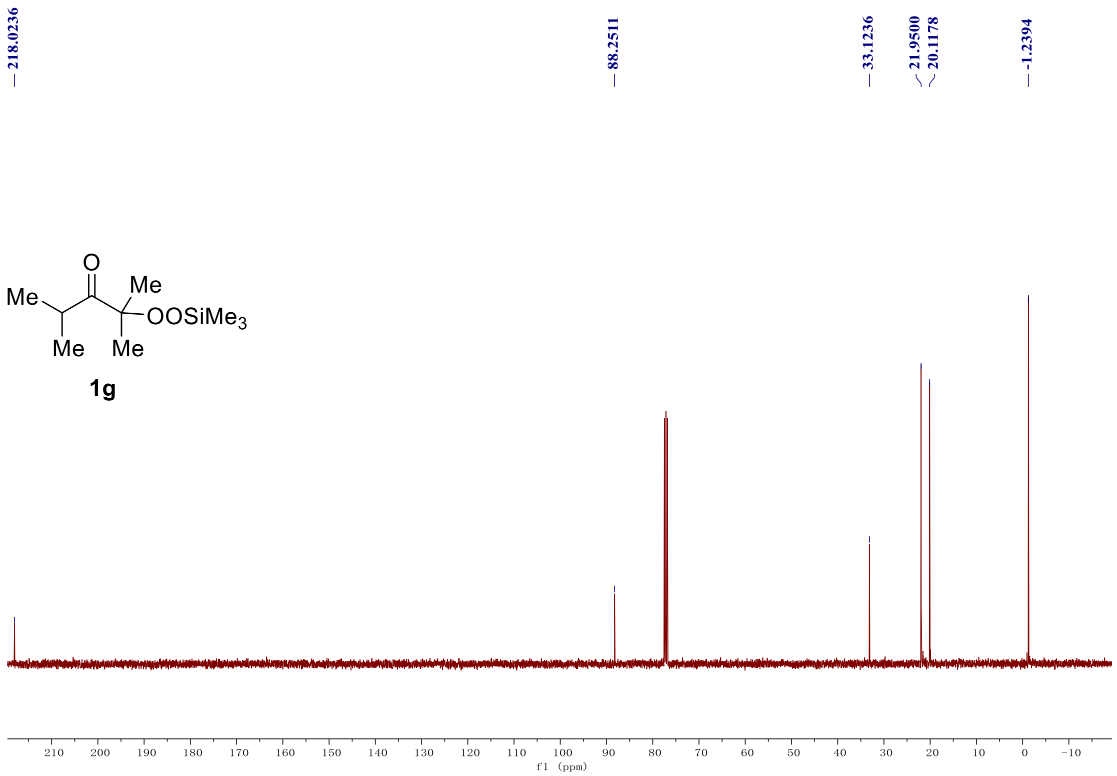

^{13}C NMR (101 MHz, $CDCl_3$) spectrum of compound **1g**

2,6-Dimethyl-2-((trimethylsilyl)peroxy)cyclohexan-1-one (1h)

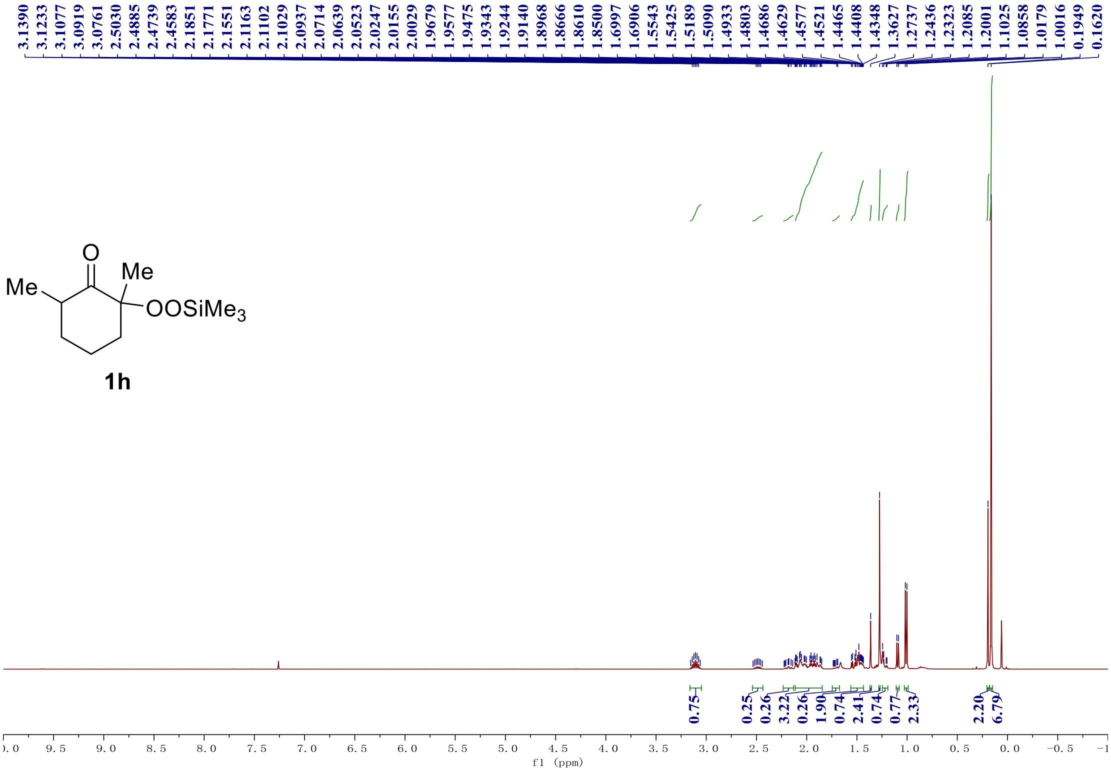

^{1}H NMR (400 MHz, CDCl$_3$) spectrum of compound **1h**

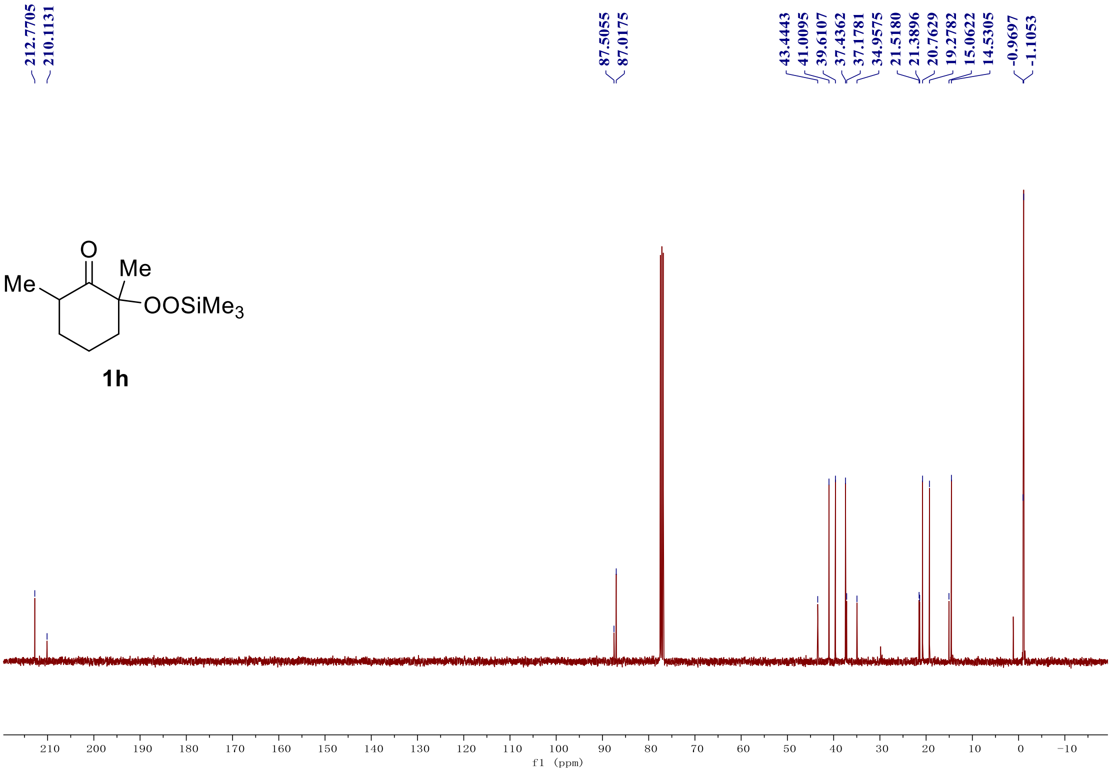

^{13}C NMR (101 MHz, CDCl$_3$) spectrum of compound **1h**

(2*S*, 5*R*)-2-Isopropyl-5-methyl-2-((trimethylsilyl)peroxy)cyclohexan-1-one (1i)

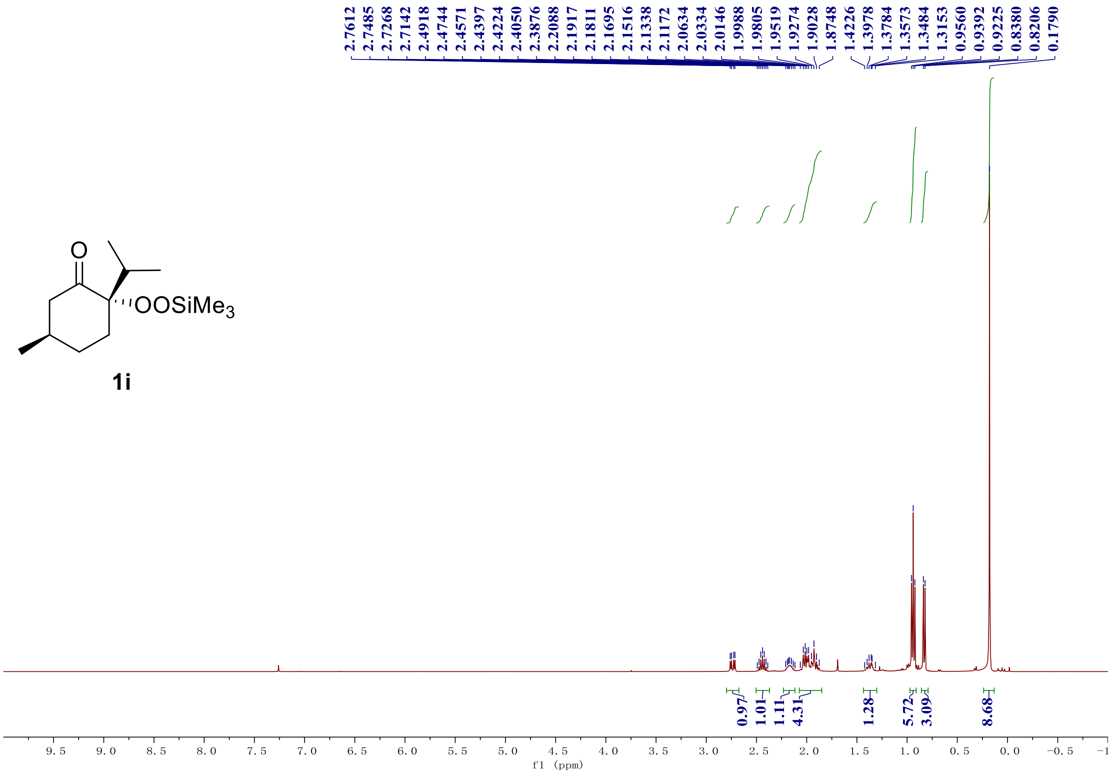

^{1}H NMR (400 MHz, CDCl$_3$) spectrum of compound **1i**

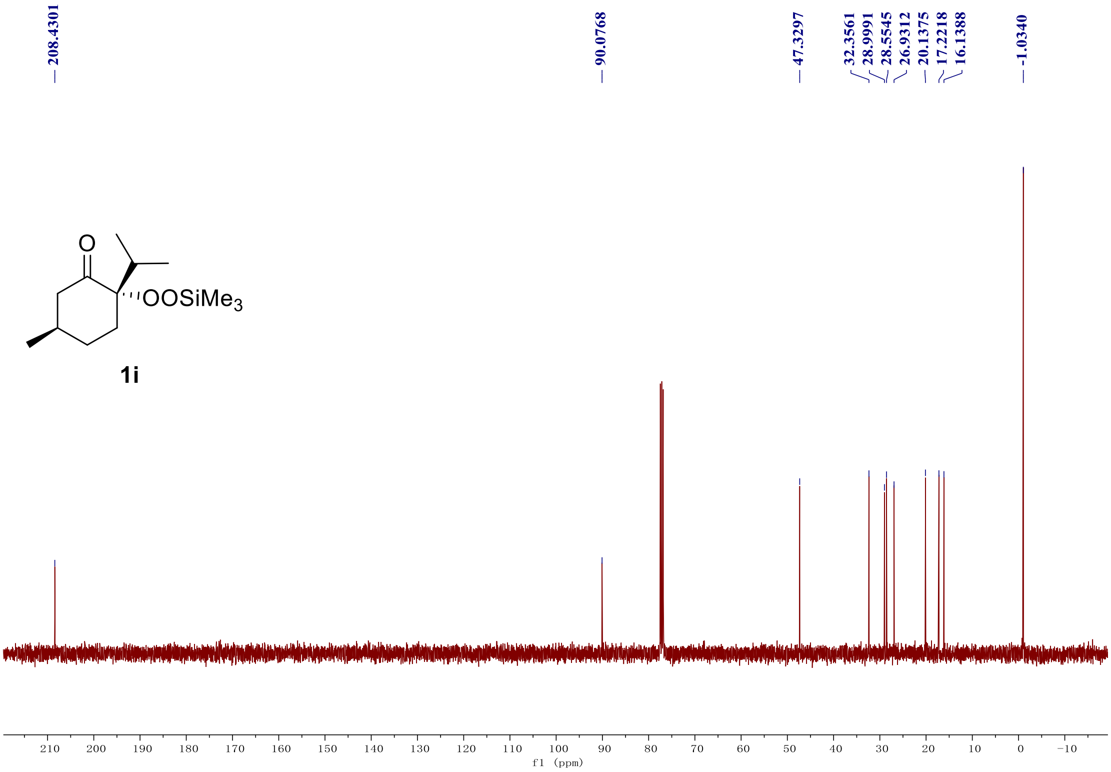

^{13}C NMR (101 MHz, CDCl$_3$) spectrum of compound **1i**

(2*R*, 5*R*)-2-Isopropyl-5-methyl-2-((trimethylsilyl)peroxy)cyclohexan-1-one (1j)

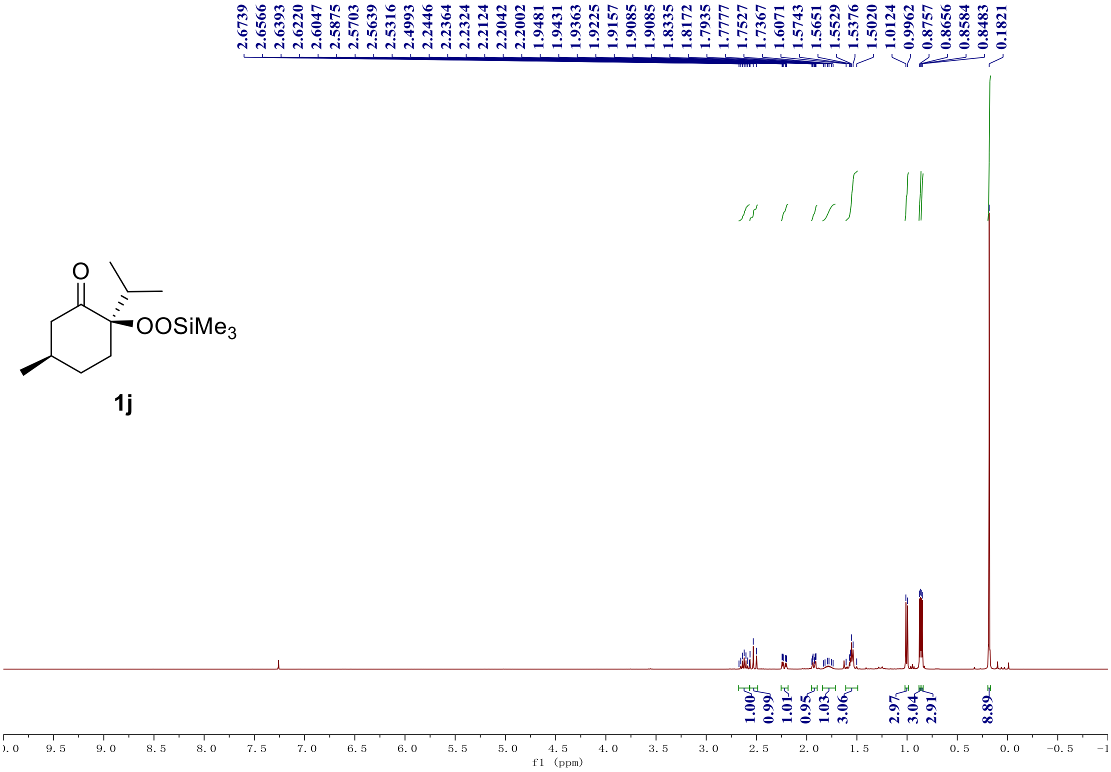

^{1}H NMR (400 MHz, CDCl$_3$) spectrum of compound **1j**

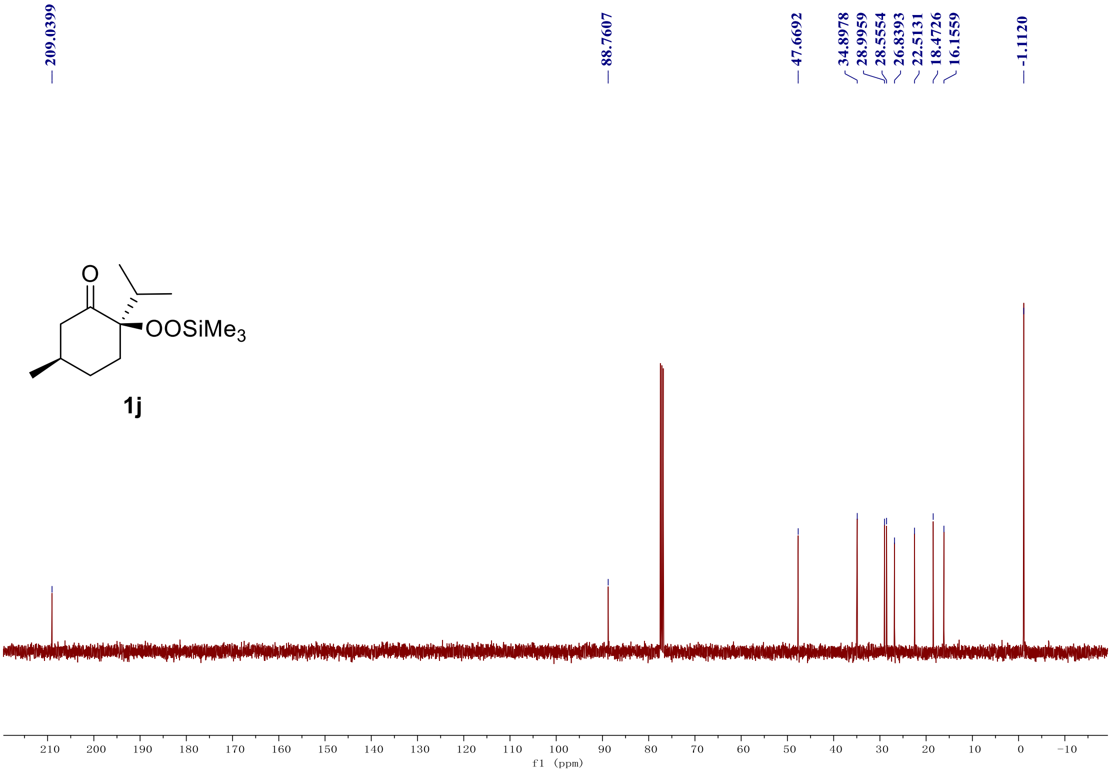

^{13}C NMR (101 MHz, CDCl$_3$) spectrum of compound **1j**

2-Ethyl-2-((trimethylsilyl)peroxy)cyclohexan-1-one (1k)

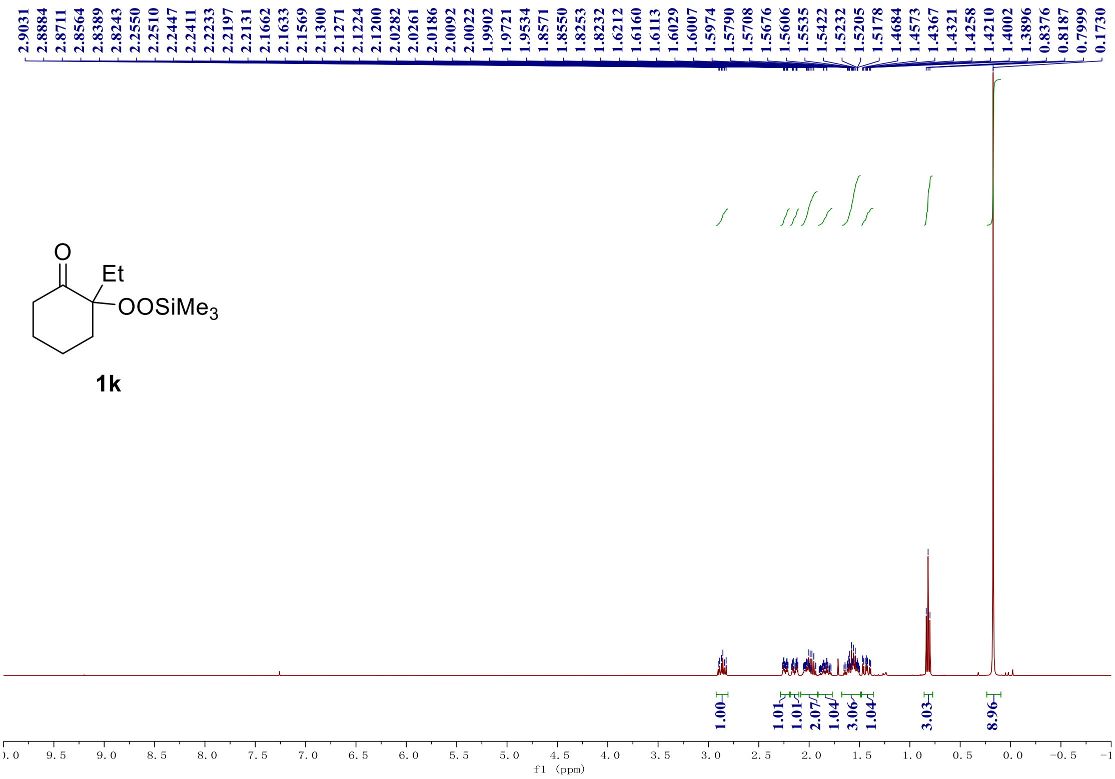

^{1}H NMR (400 MHz, CDCl$_3$) spectrum of **1k**

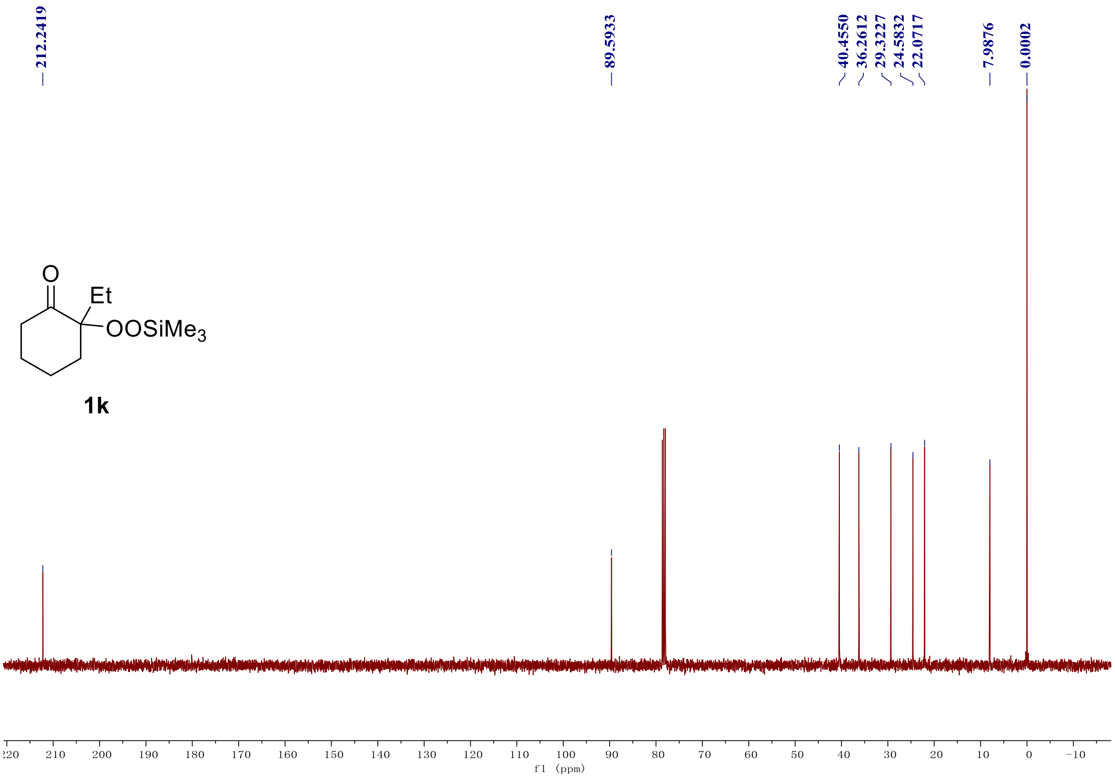

^{13}C NMR (101 MHz, CDCl$_3$) spectrum of **1k**

2-Phenyl-2-((triethylsilyl)peroxy)cyclohexan-1-one (1m)

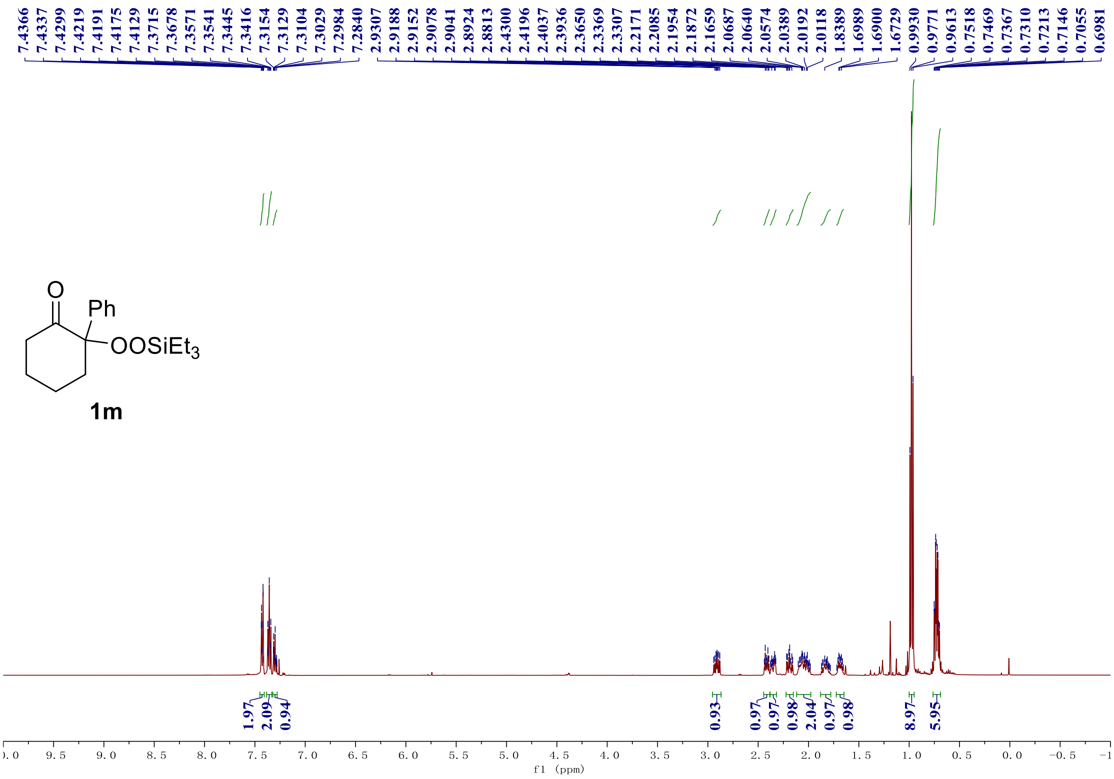

^{1}H NMR (500 MHz, CDCl$_3$) spectrum of **1m**

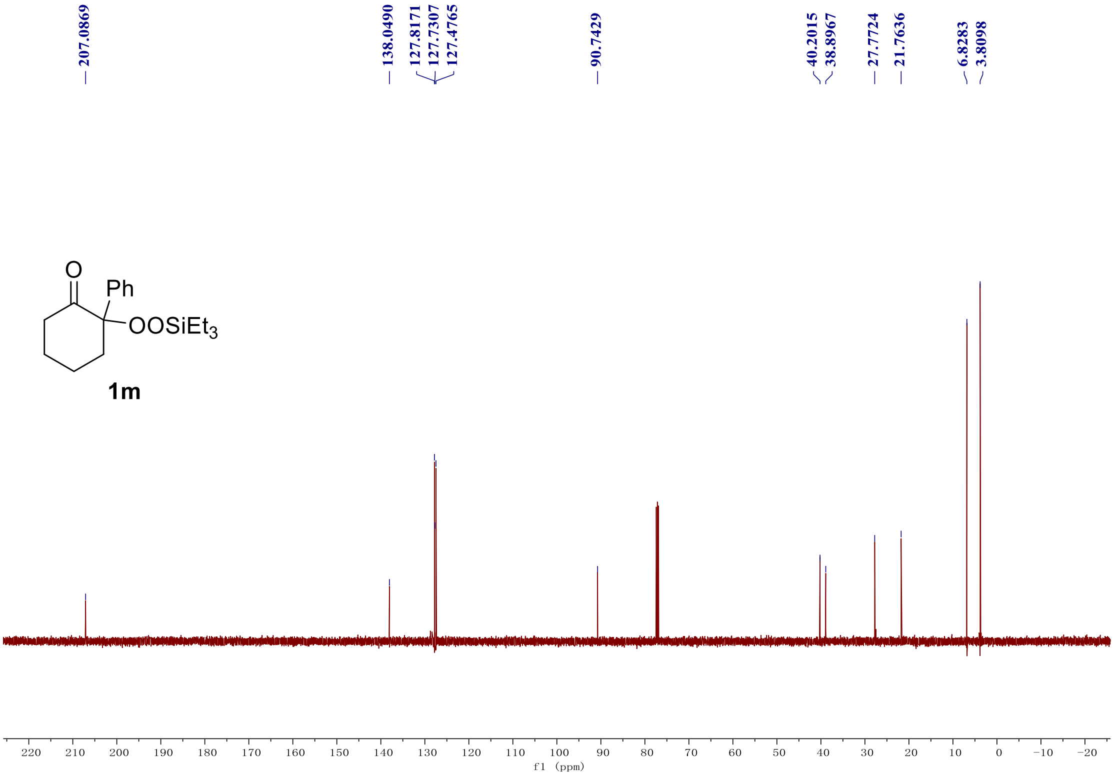

^{13}C NMR (126 MHz, CDCl$_3$) spectrum of **1m**

1-(1,3-Dimethyl-2-oxoindolin-3-yl)octane-2,7-dione (8a)

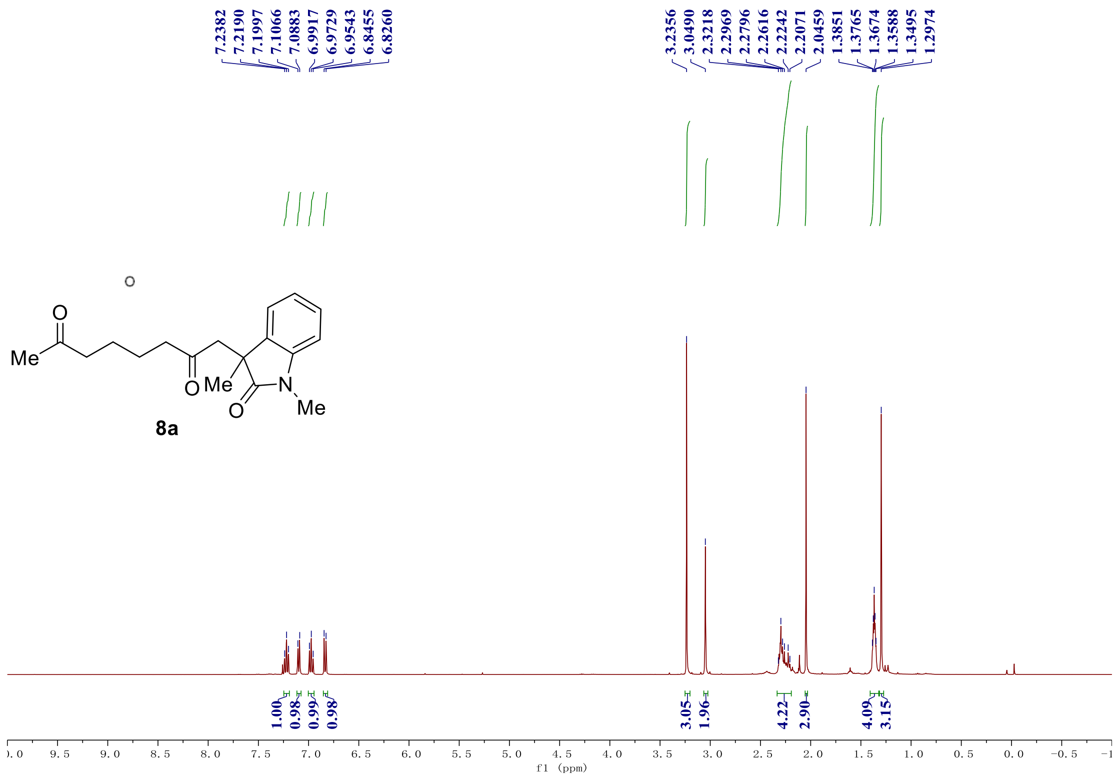

^{1}H NMR (400 MHz, $CDCl_3$) spectrum of compound **8a**

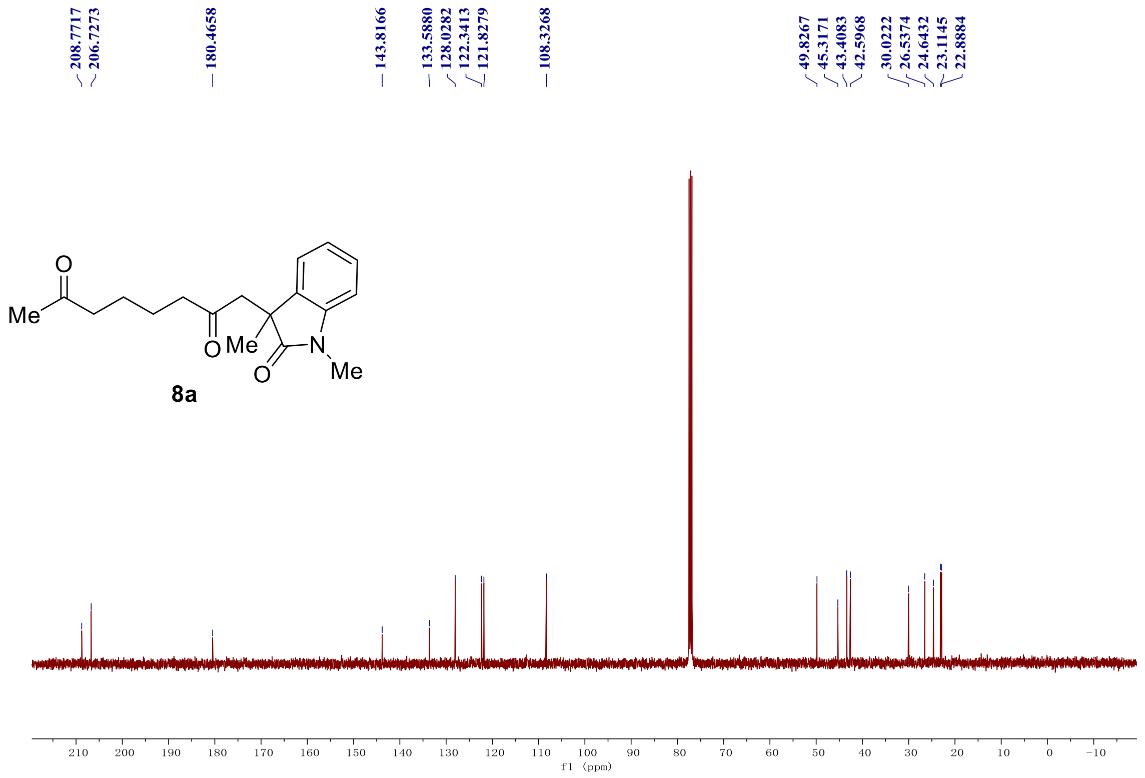

^{13}C NMR (101 MHz, $CDCl_3$) spectrum of compound **8a**

1-(1,3-Dimethyl-2-oxoindolin-3-yl)heptane-2,6-dione (8b)

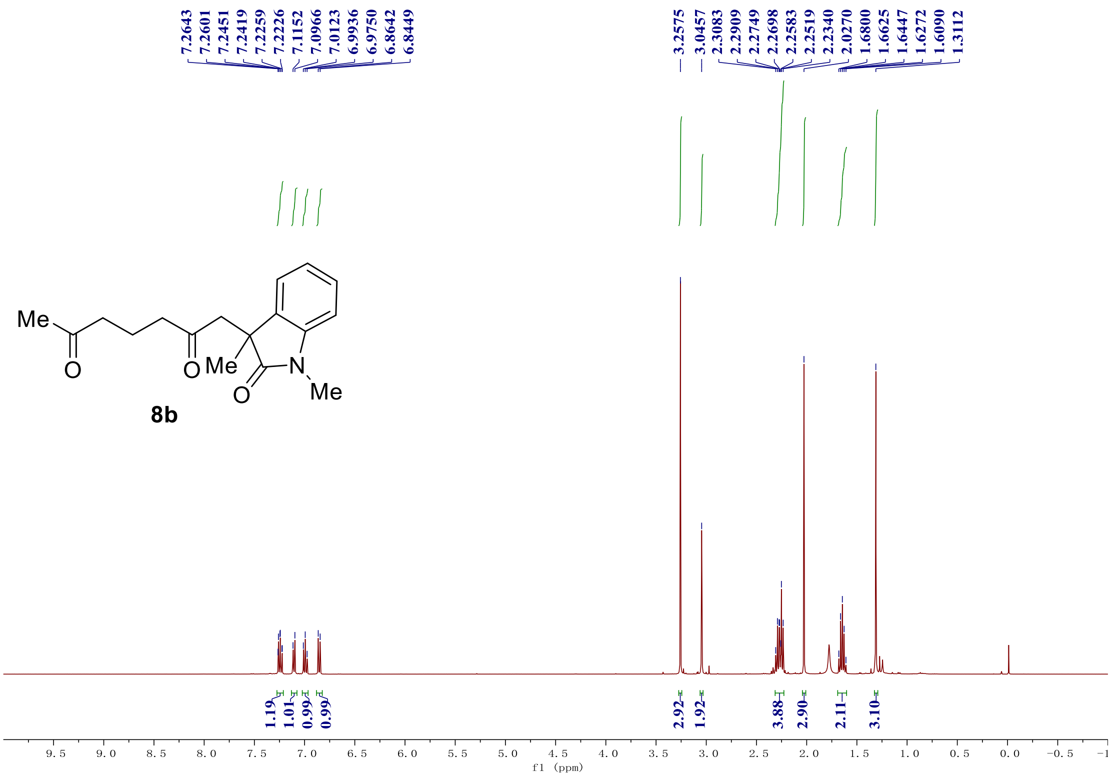

^{1}H NMR (400 MHz, $CDCl_3$) spectrum of compound **8b**

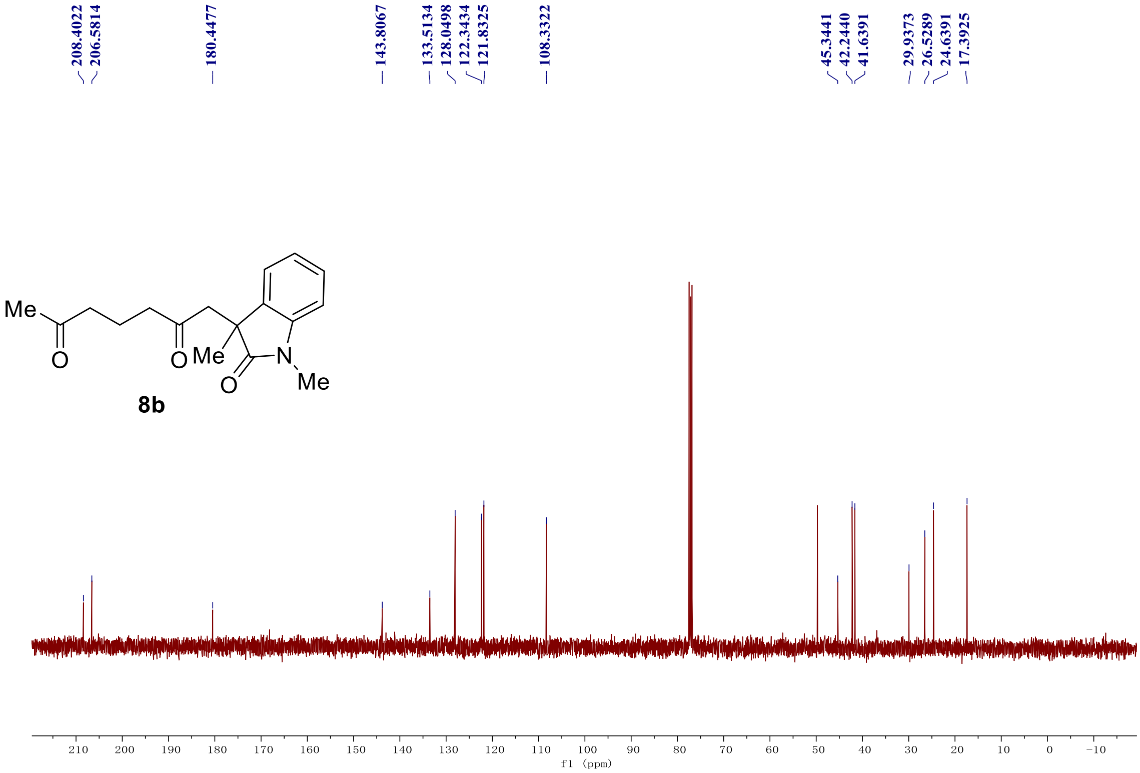

^{13}C NMR (101 MHz, $CDCl_3$) spectrum of compound **8b**

1-(1,3-Dimethyl-2-oxoindolin-3-yl)nonane-2,8-dione (8c)

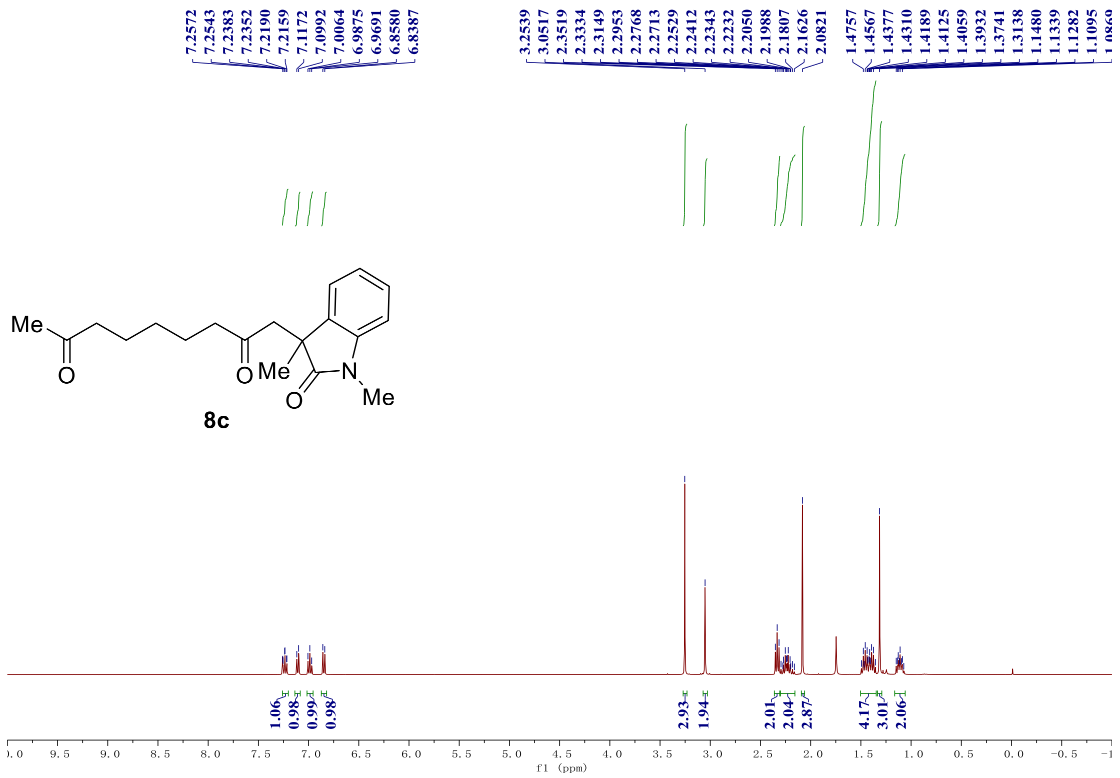

^{1}H NMR (400 MHz, $CDCl_3$) spectrum of compound **8c**

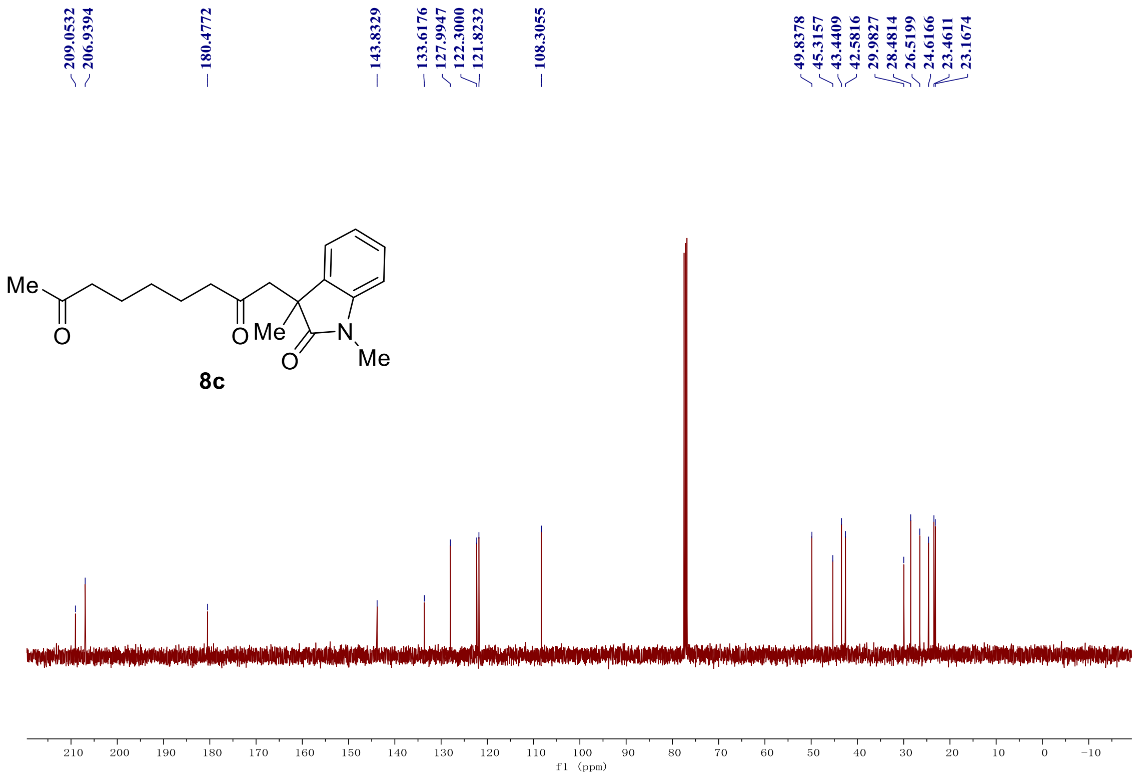

^{13}C NMR (101 MHz, $CDCl_3$) spectrum of compound **8c**

1-(1,3-Dimethyl-2-oxoindolin-3-yl)decane-2,9-dione (8d)

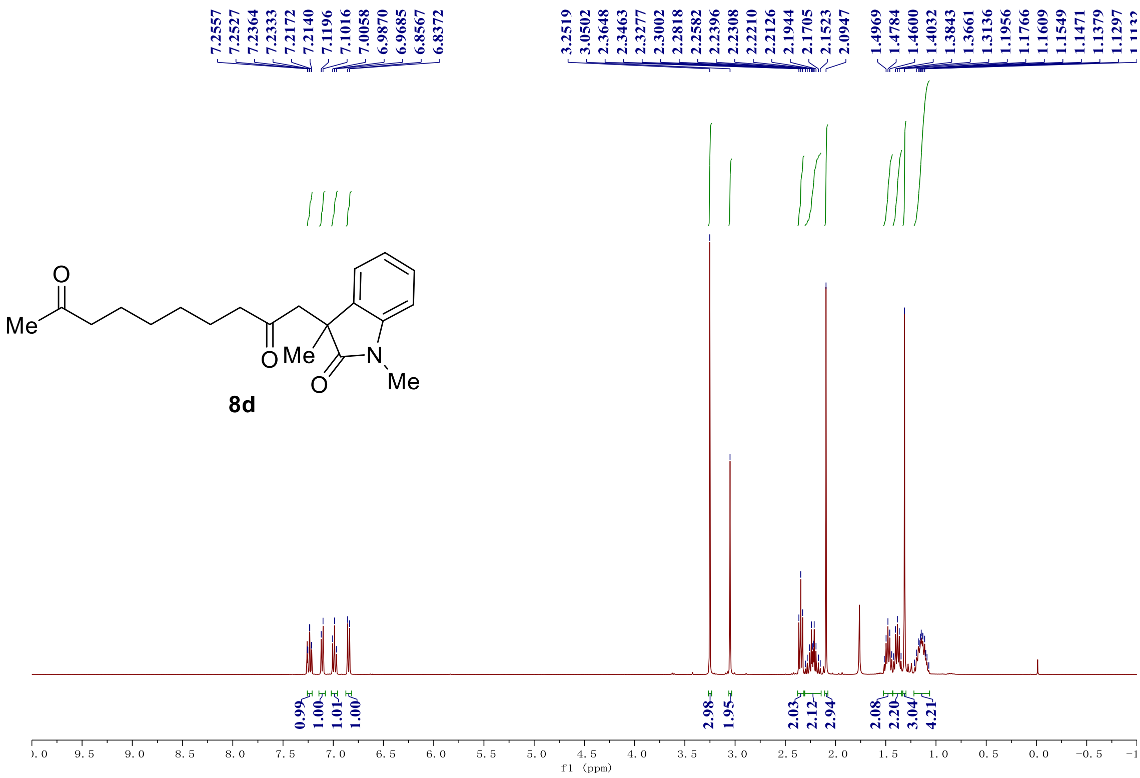

^{1}H NMR (400 MHz, $CDCl_3$) spectrum of compound **8d**

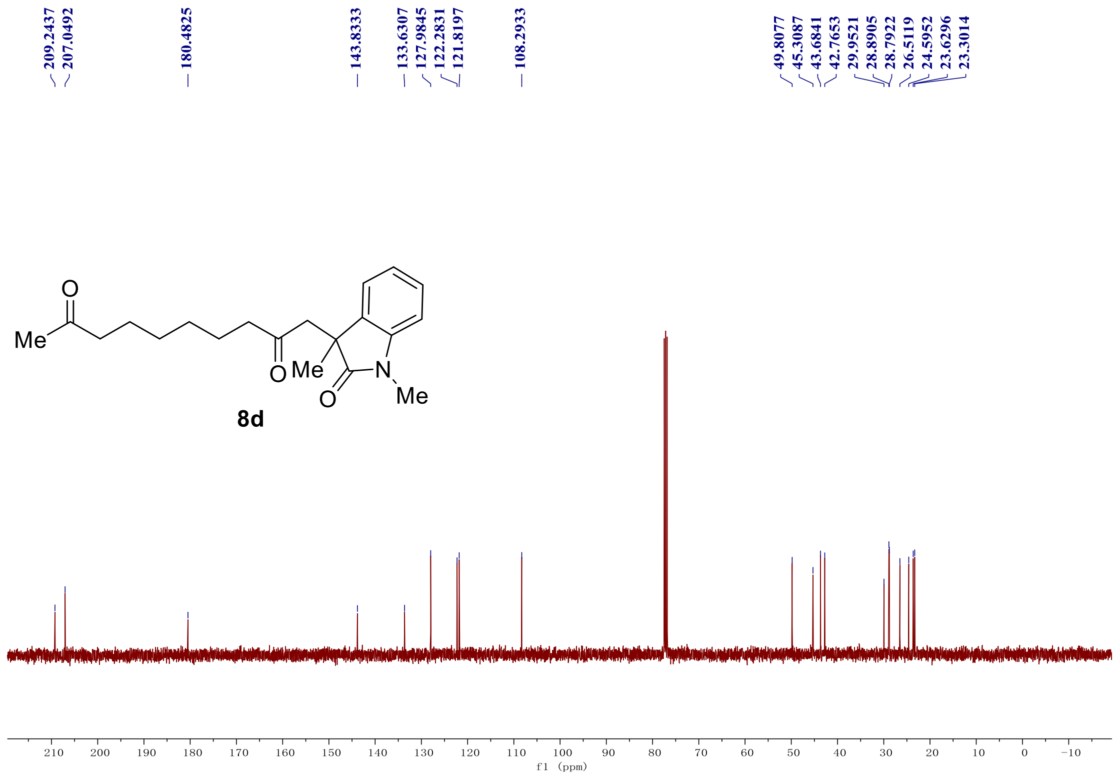

^{13}C NMR (101 MHz, $CDCl_3$) spectrum of compound **8d**

1,3-Dimethyl-3-(2-oxo-2-(2-(3-oxobutyl)phenyl)ethyl)indolin-2-one (8e)

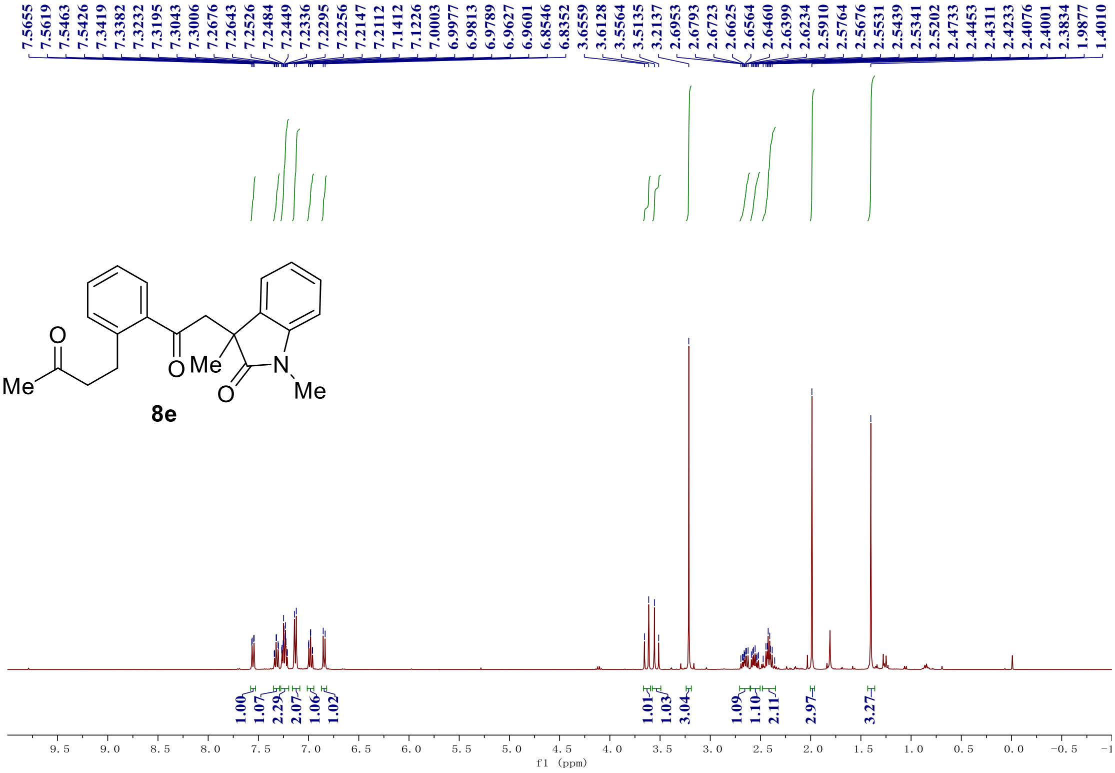

^{1}H NMR (400 MHz, $CDCl_3$) spectrum of compound **8e**

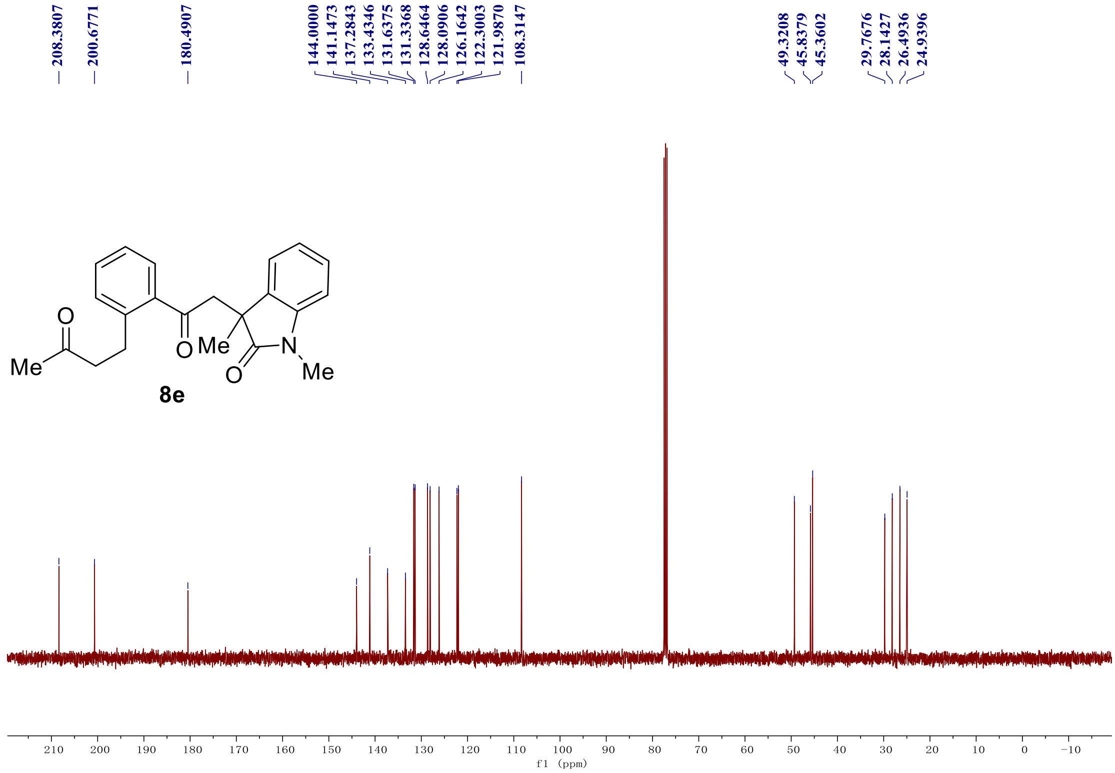

^{13}C NMR (101 MHz, $CDCl_3$) spectrum of compound **8e**

1,3-Dimethyl-3-(2-oxobutyl)indolin-2-one (8f)

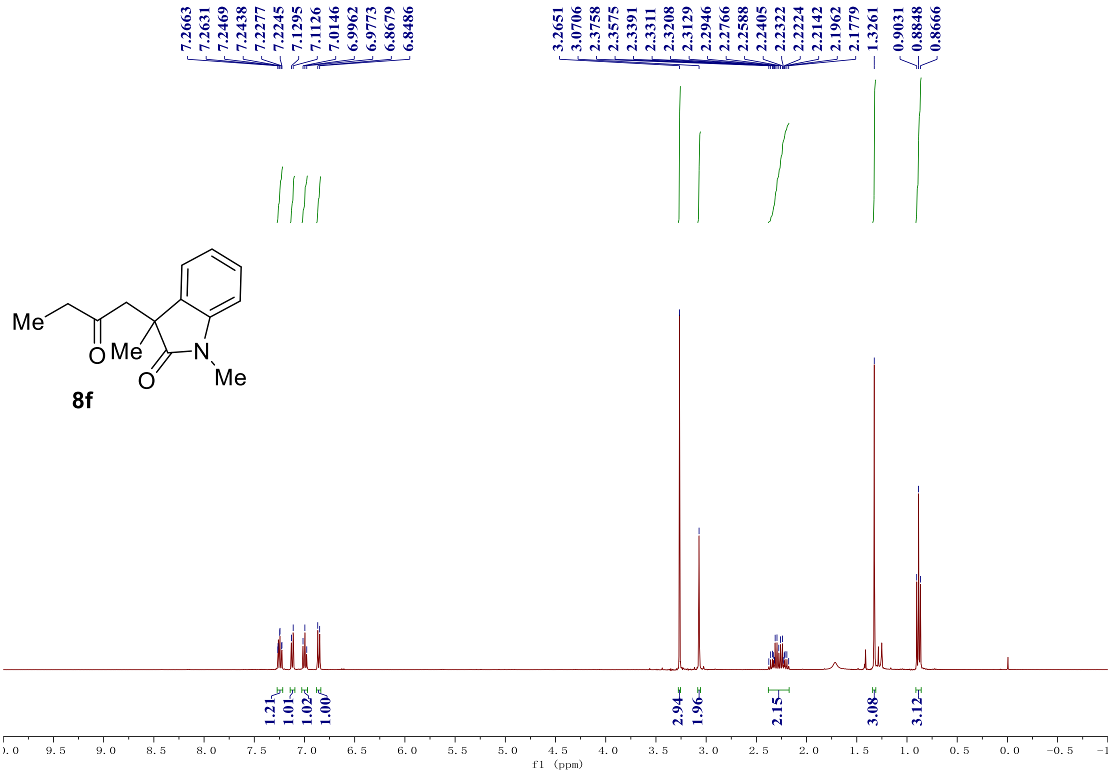

^{1}H NMR (400 MHz, CDCl$_3$) spectrum of compound **8f**

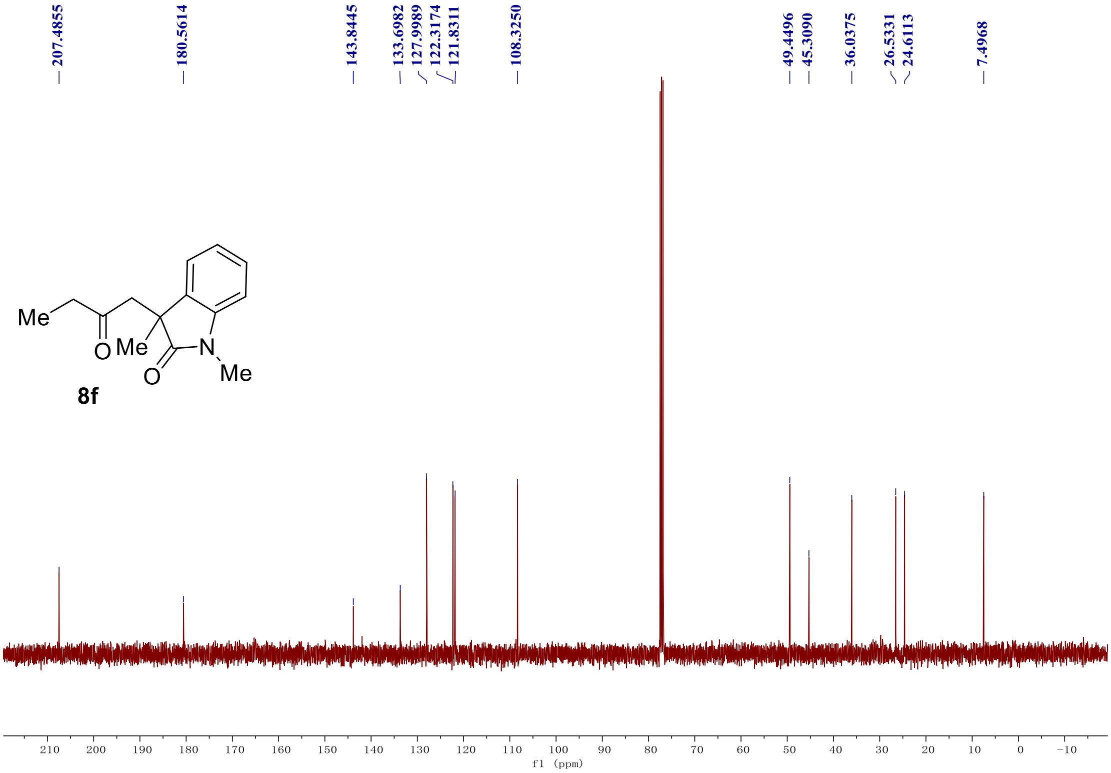

^{13}C NMR (101 MHz, CDCl$_3$) spectrum of compound **8f**

1,3-Dimethyl-3-(3-methyl-2-oxobutyl)indolin-2-one (8g)

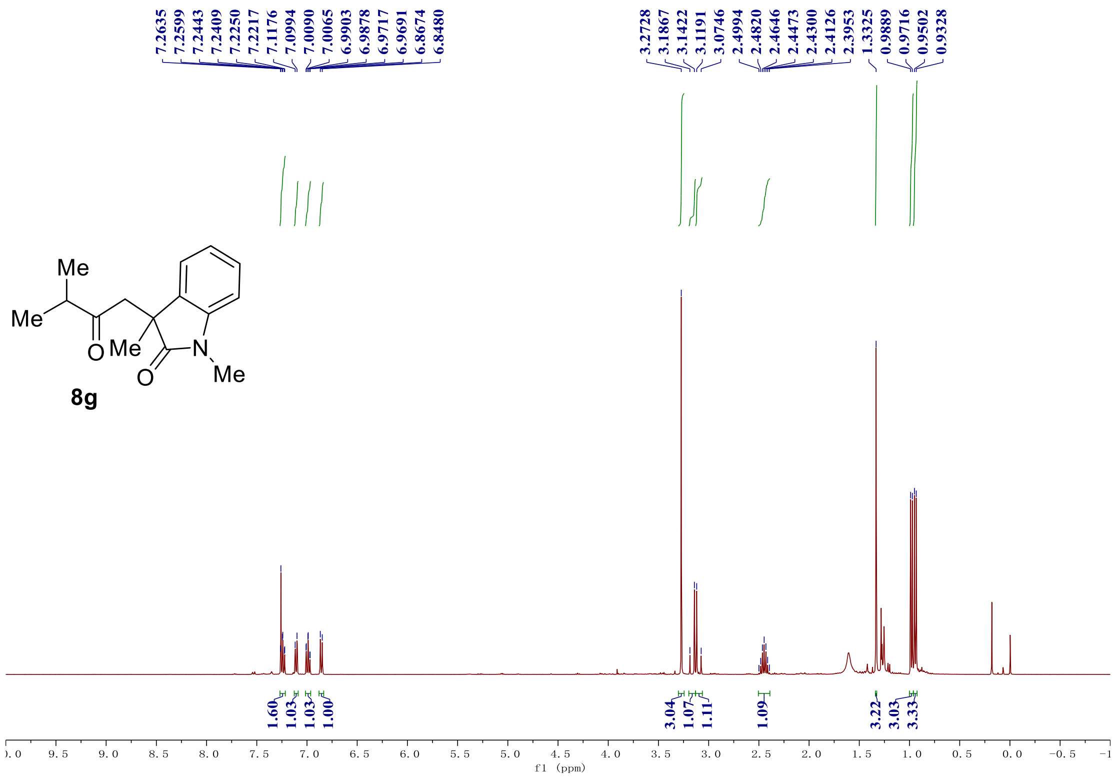

^{1}H NMR (400 MHz, $CDCl_3$) spectrum of compound **8g**

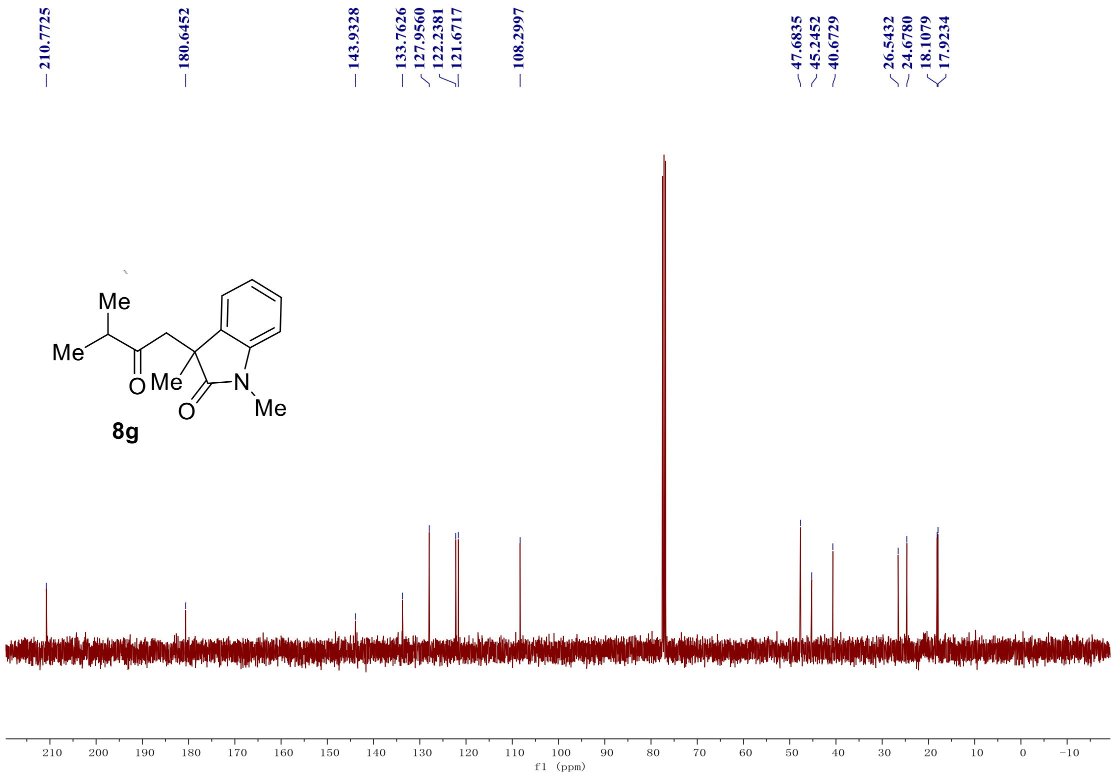

^{13}C NMR (101 MHz, $CDCl_3$) spectrum of compound **8g**

1-(1,3-Dimethyl-2-oxoindolin-3-yl)-3-methyloctane-2,7-dione (8h)

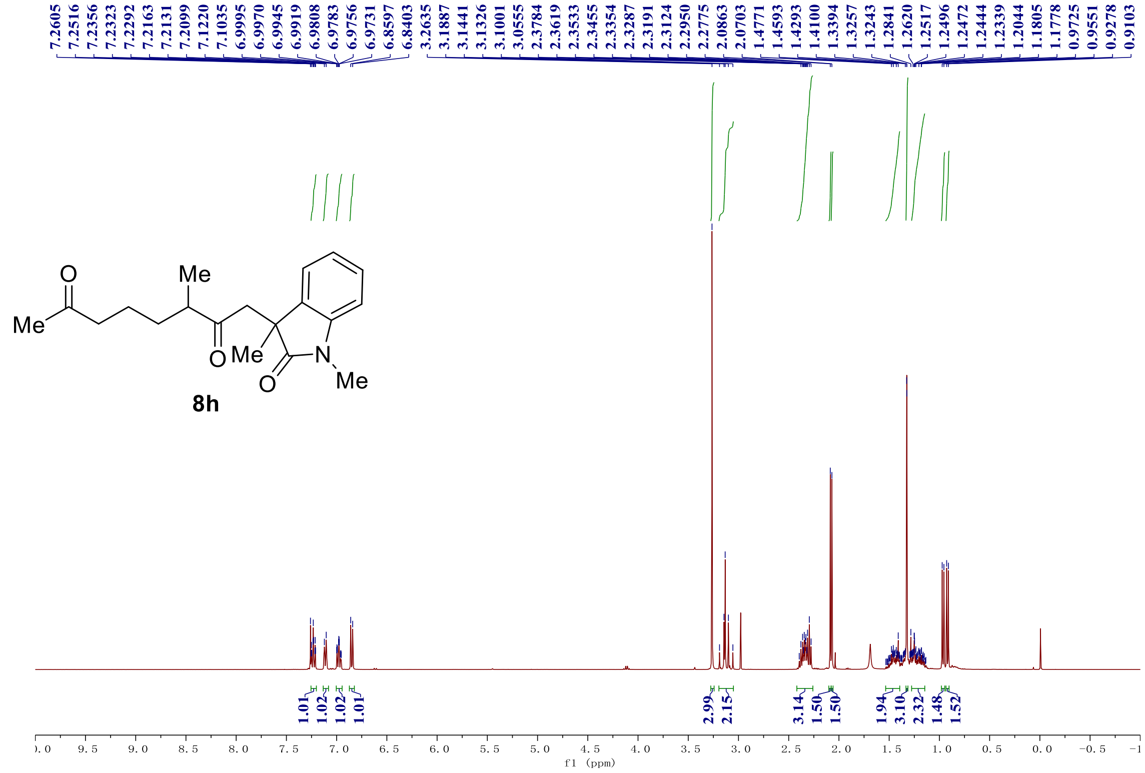

^{1}H NMR (400 MHz, $CDCl_3$) spectrum of compound **8h**

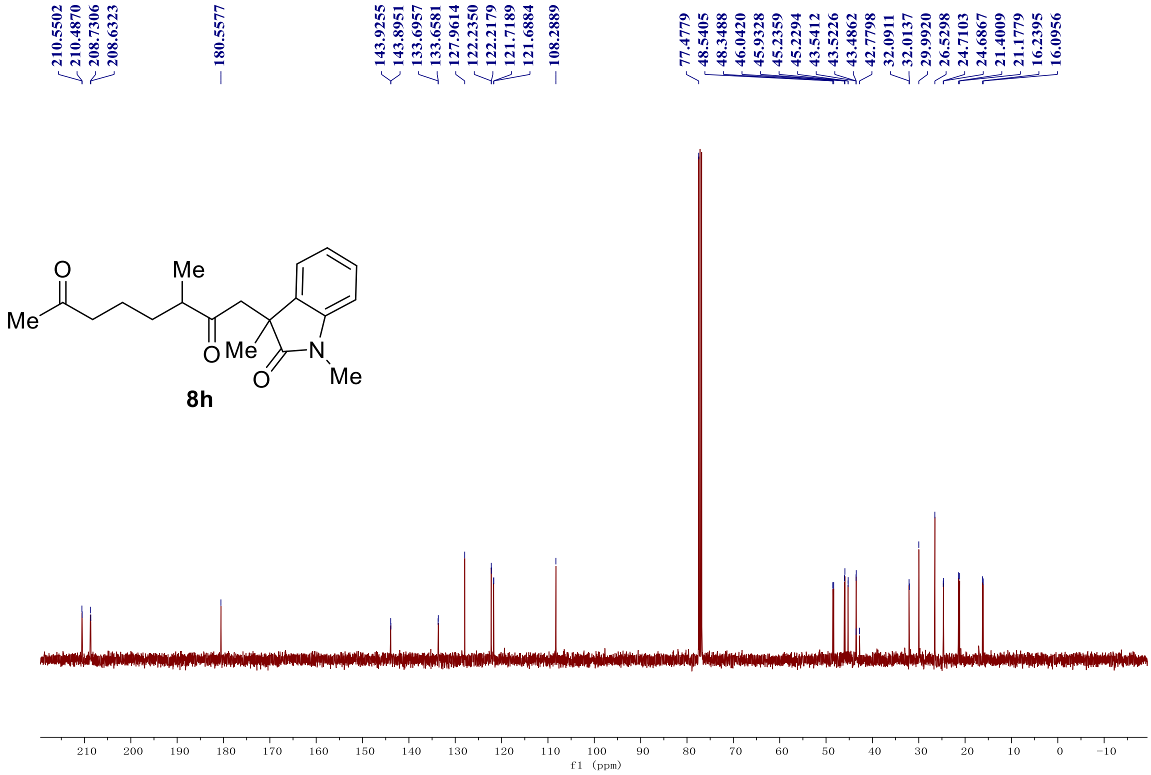

^{13}C NMR (101 MHz, $CDCl_3$) spectrum of compound **8h**

(4*R*)-1-(1,3-Dimethyl-2-oxoindolin-3-yl)-4,8-dimethylnonane-2,7-dione (8i)

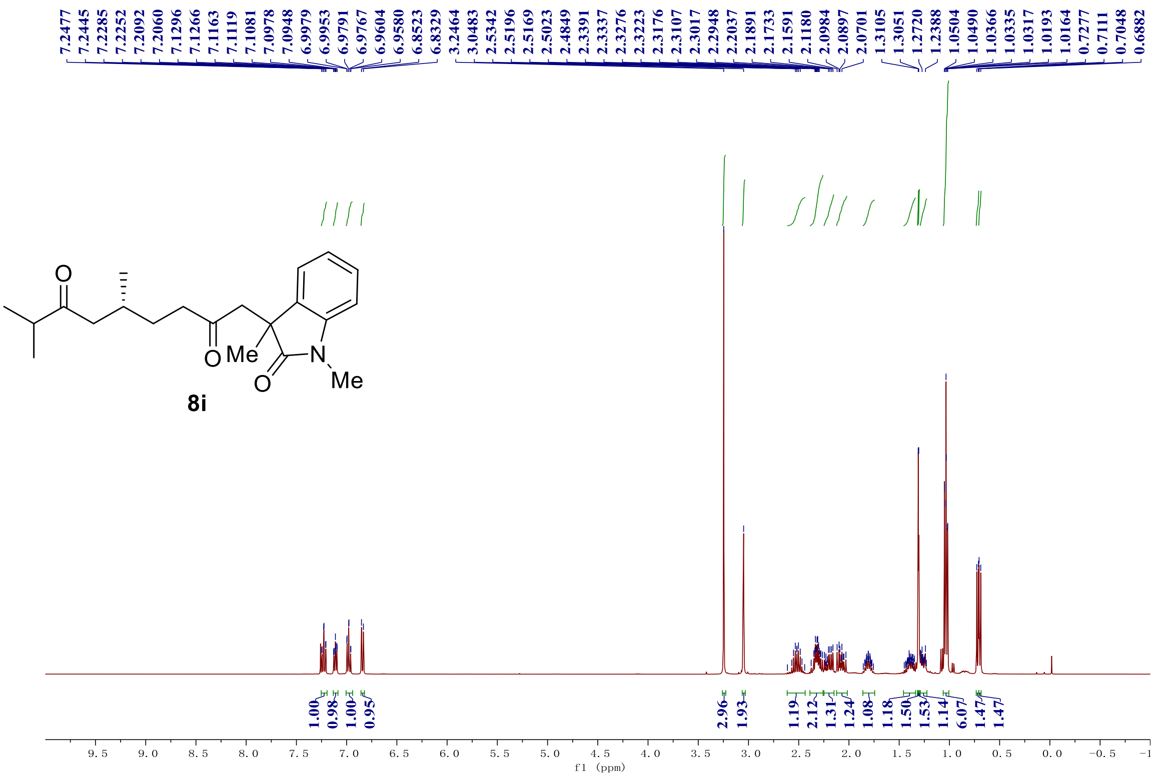

^{1}H NMR (400 MHz, CDCl$_3$) spectrum of compound **8i**

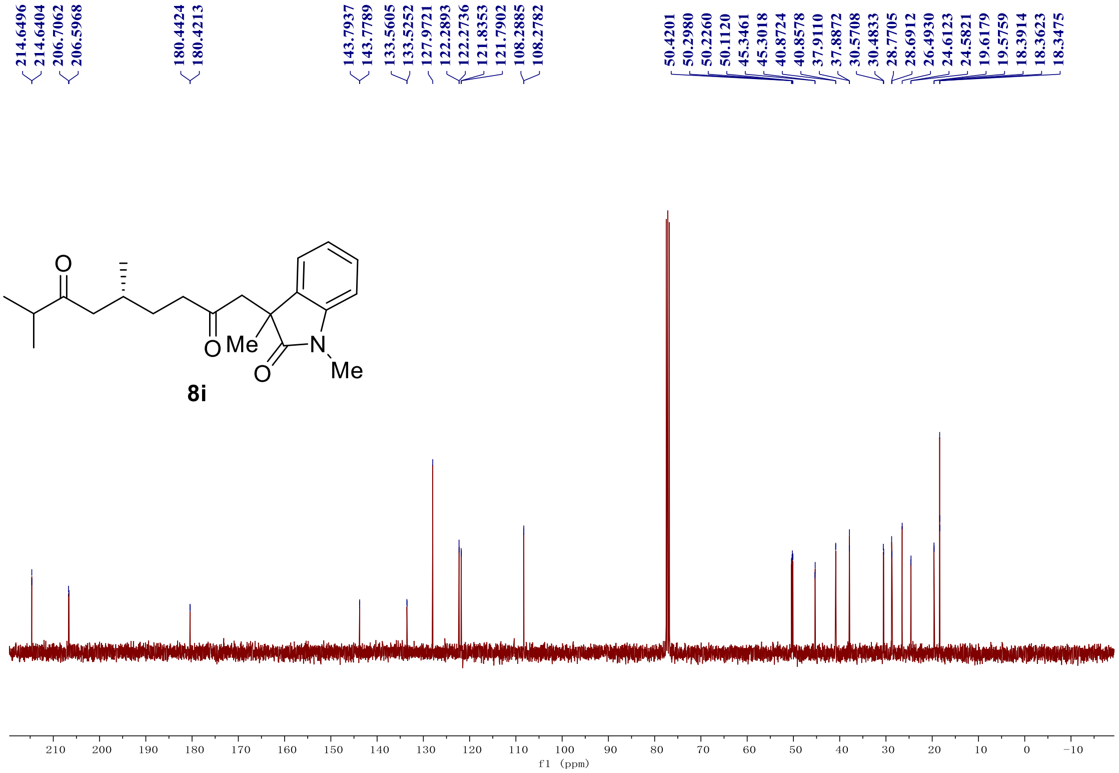

^{13}C NMR (101 MHz, CDCl$_3$) spectrum of compound **8i**

1-(1,3-Dimethyl-2-oxoindolin-3-yl)nonane-2,7-dione (8k)

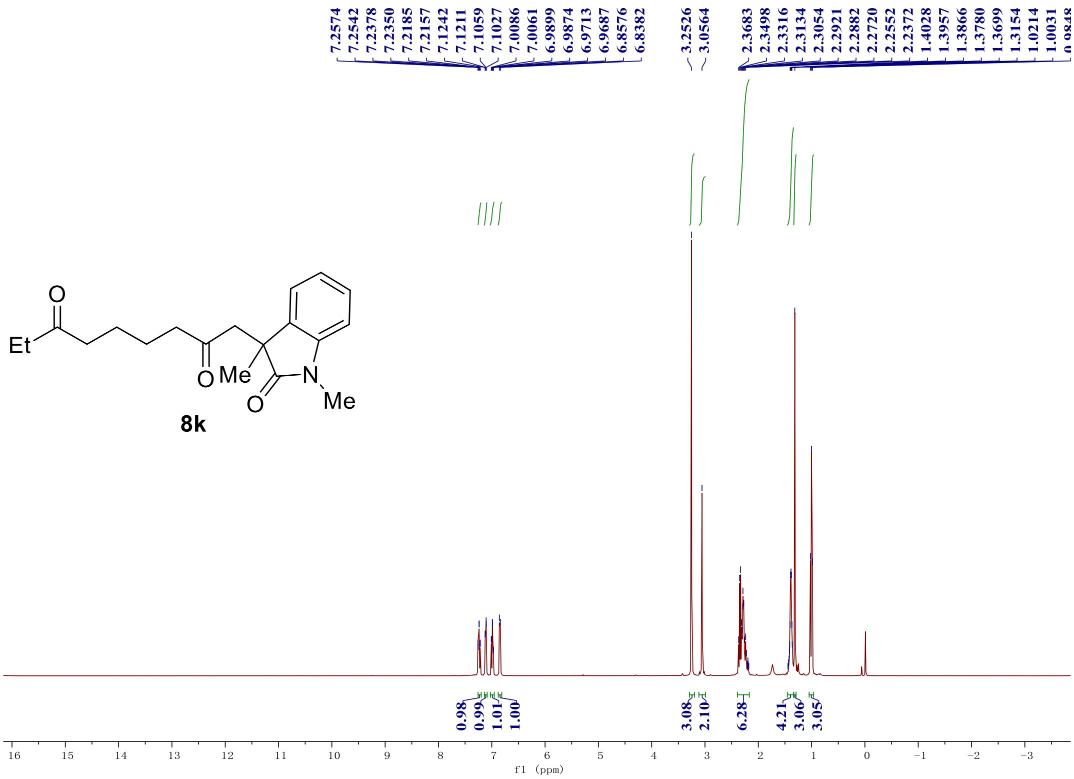

^{1}H NMR (400 MHz, $CDCl_3$) spectrum of **8k**

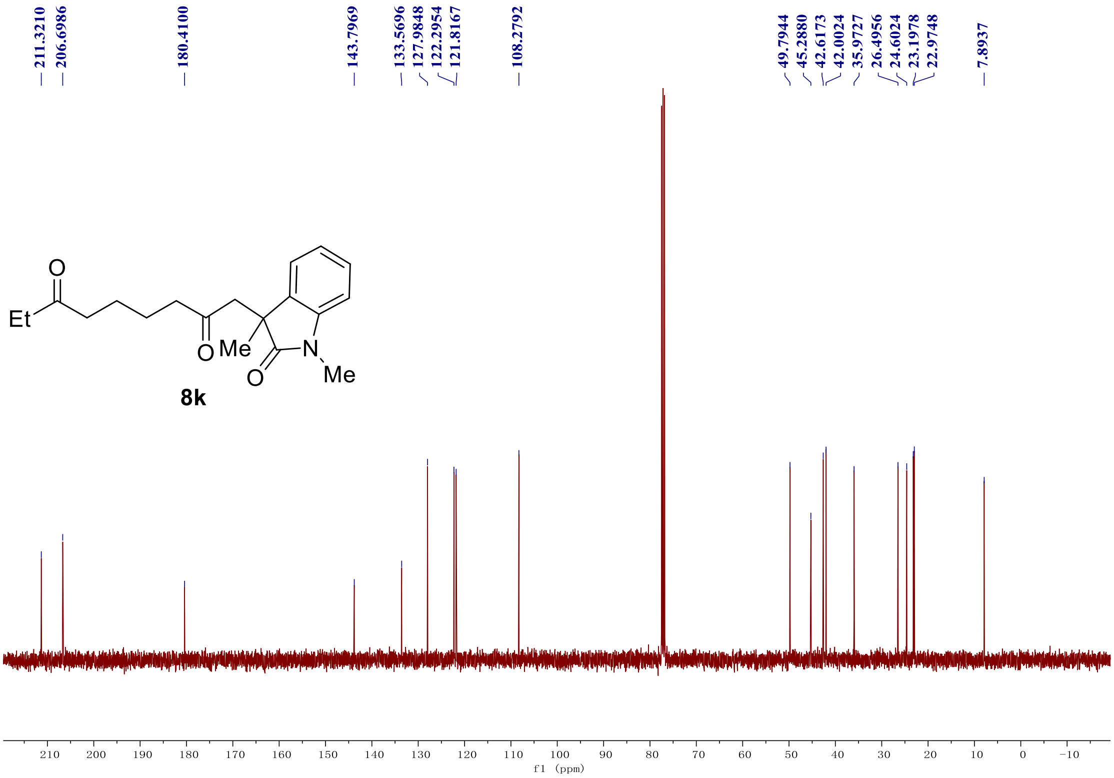

^{13}C NMR (101 MHz, $CDCl_3$) spectrum of **8k**

7-(1,3-Dimethyl-2-oxoindolin-3-yl)-1-phenylheptane-1,6-dione (8m)

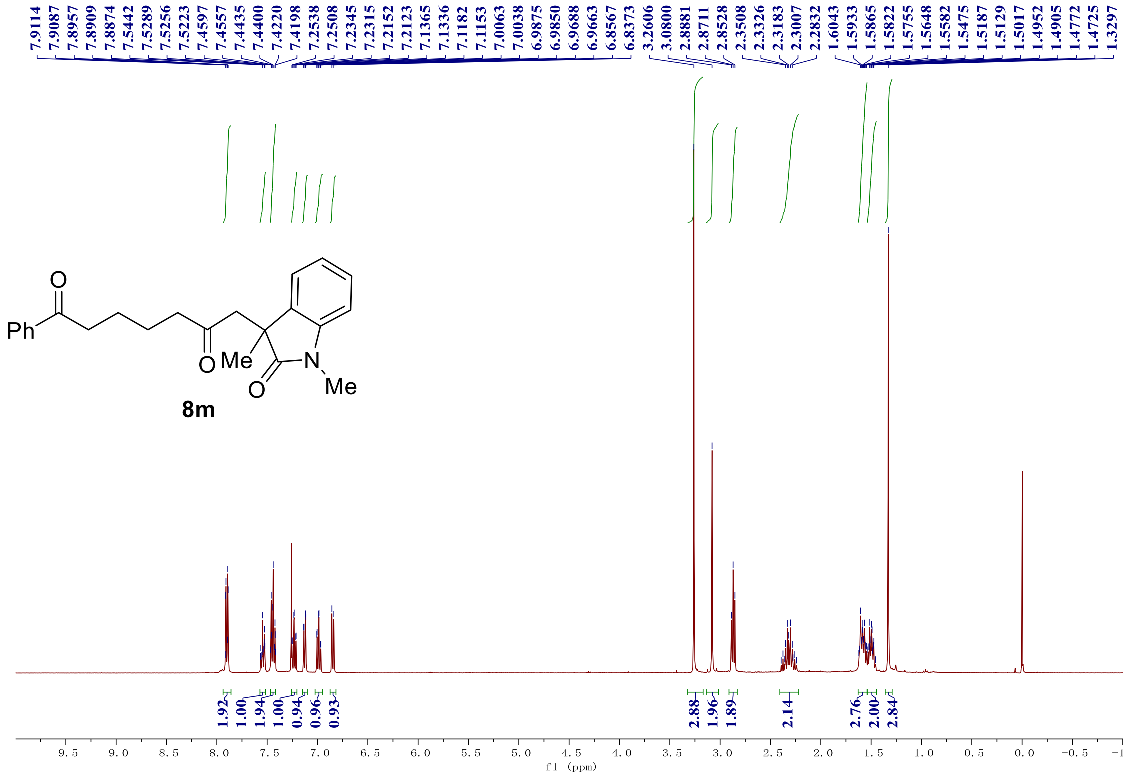

^{1}H NMR (400 MHz, CDCl$_3$) spectrum of **8m**

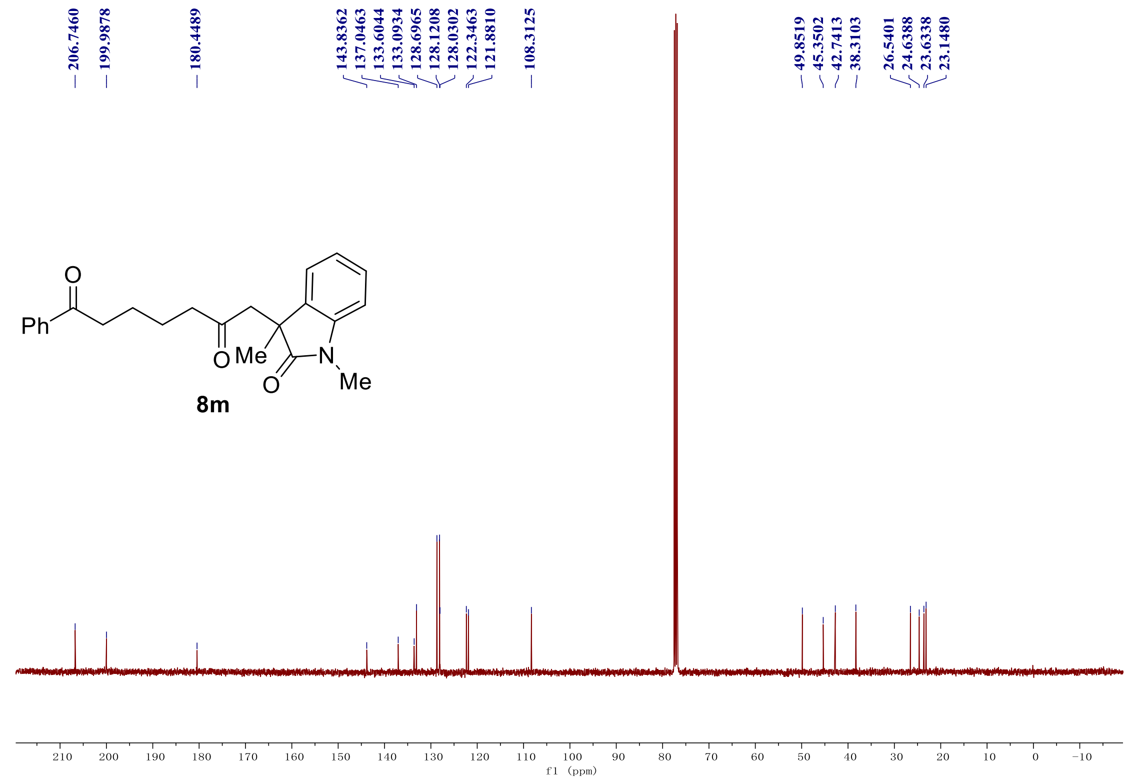

^{13}C NMR (101 MHz, CDCl$_3$) spectrum of **8m**

3-Isobutyl-1,3-dimethylindolin-2-one (9g)

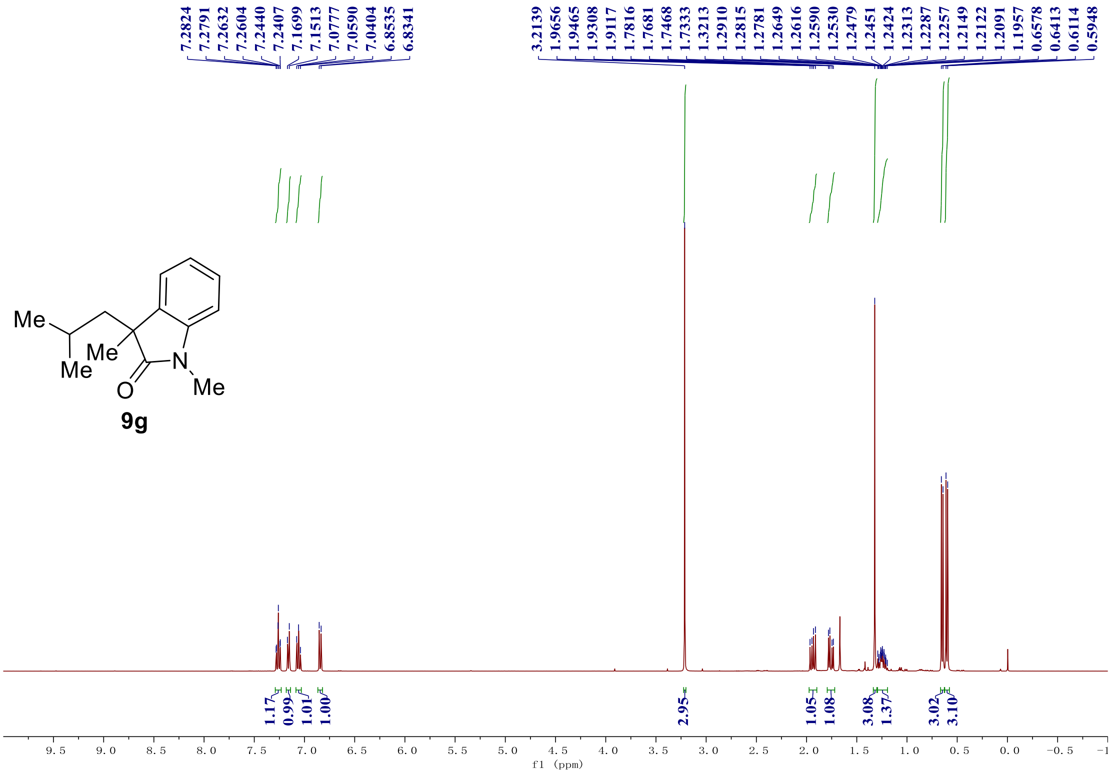

^{1}H NMR (400 MHz, $CDCl_3$) spectrum of compound **9g**

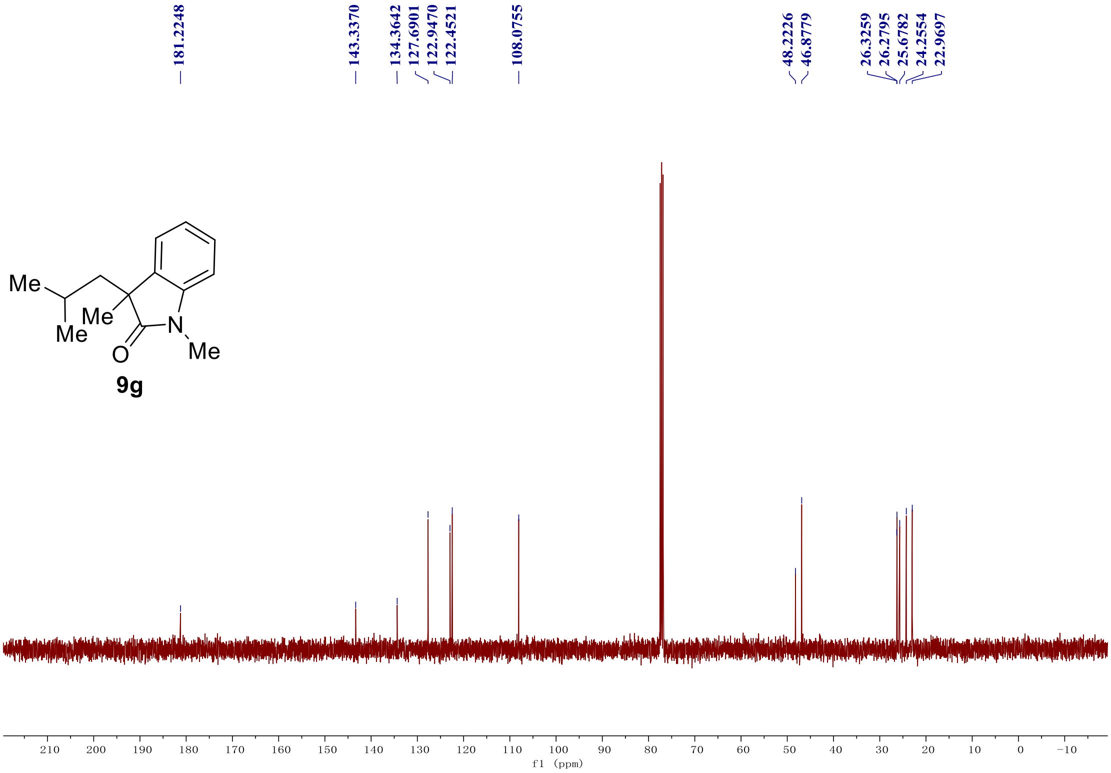

^{13}C NMR (101 MHz, $CDCl_3$) spectrum of compound **9g**

1,3-Dimethyl-3-(2-methyl-6-oxoheptyl)indolin-2-one (9h)

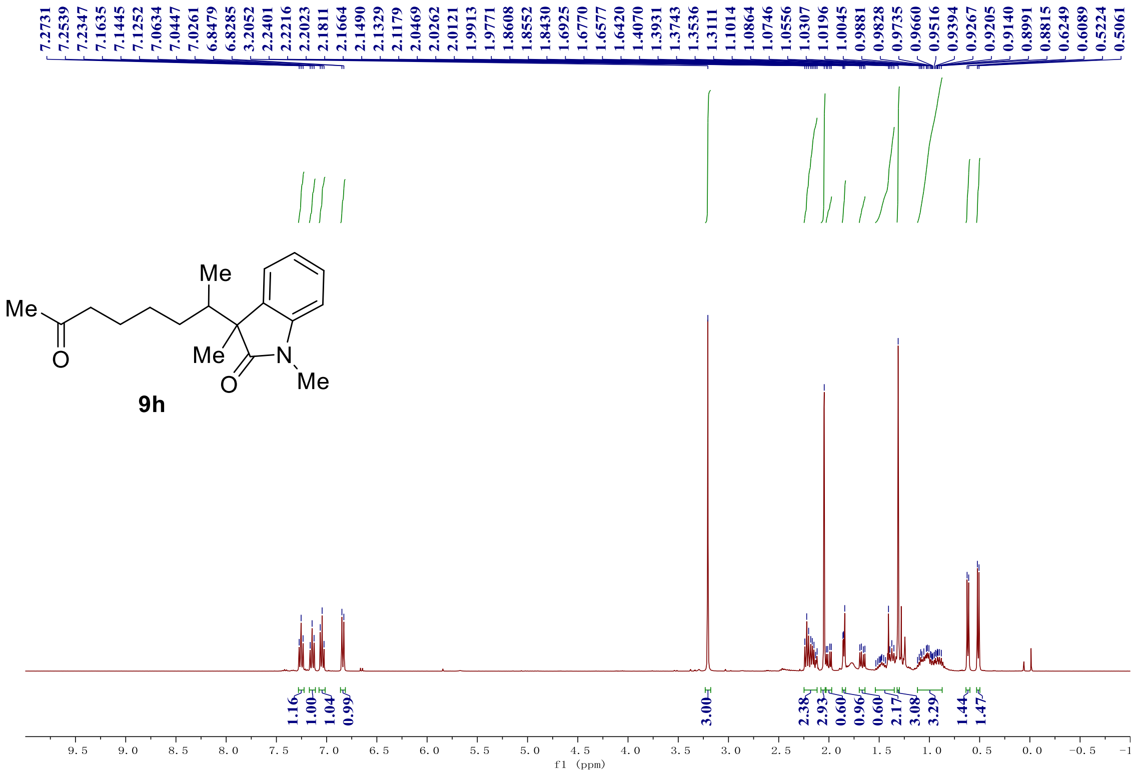

^{1}H NMR (400 MHz, $CDCl_3$) spectrum of compound **9h**

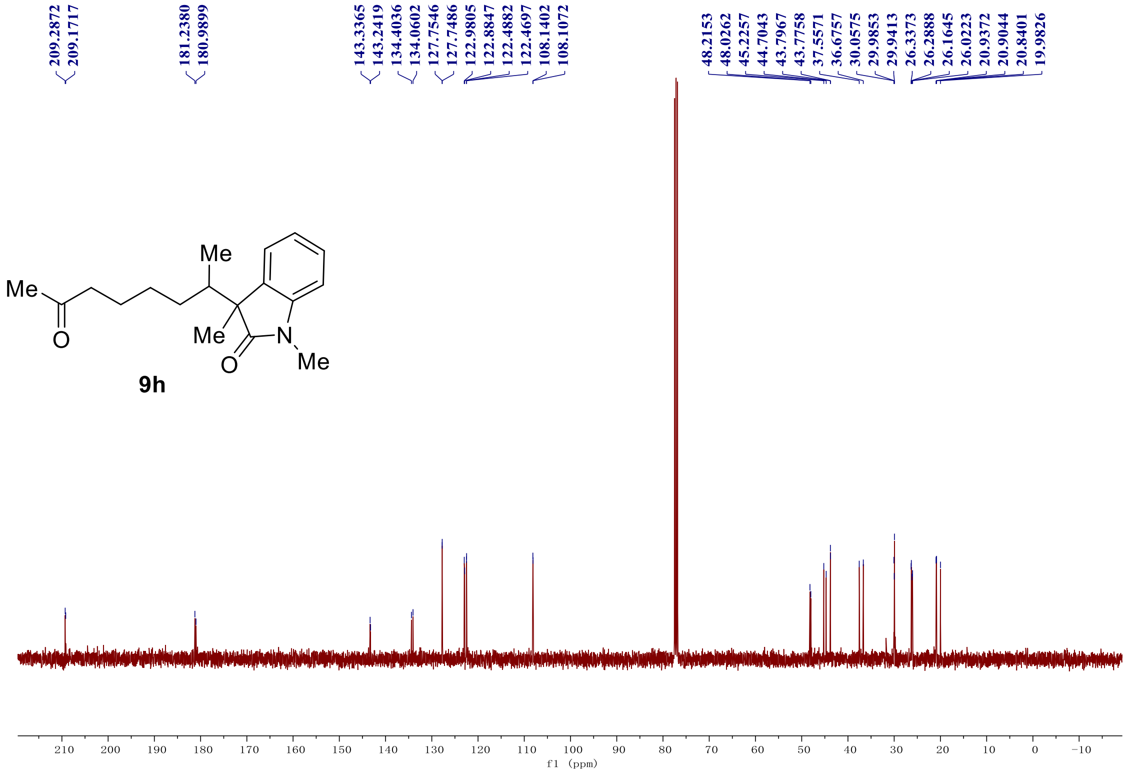

^{13}C NMR (101 MHz, $CDCl_3$) spectrum of compound **9h**

1-(4-Methyl-3-oxo-3,4-dihydroquinoxalin-2-yl)heptane-1,6-dione (11)

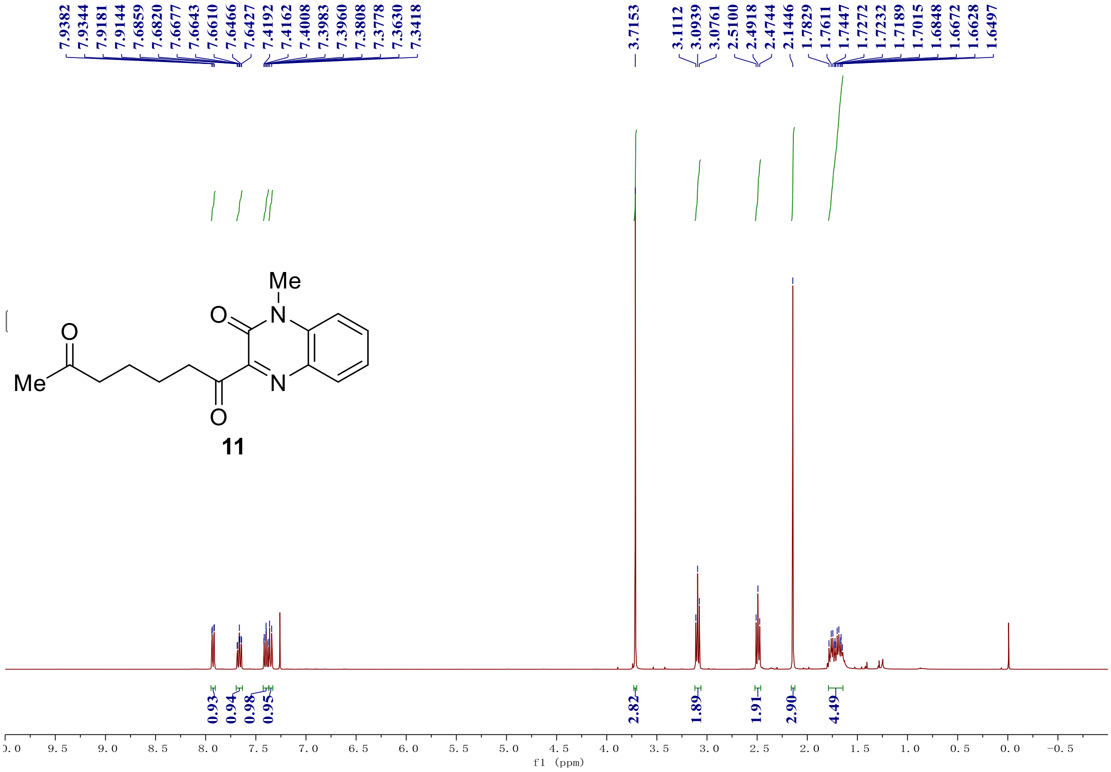

^{1}H NMR (400 MHz, CDCl$_3$) spectrum of compound **11**

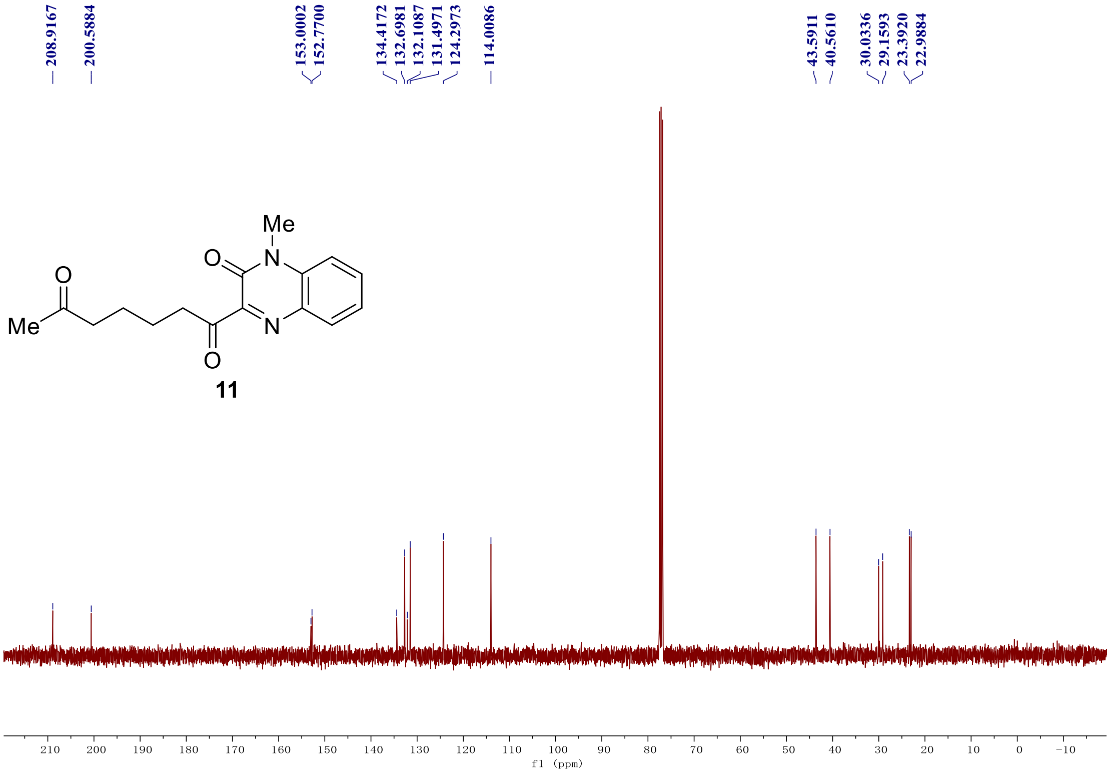

^{13}C NMR (101 MHz, CDCl$_3$) spectrum of compound **11**

1-(2-Methylphenanthridin-6-yl)heptane-1,6-dione (13)

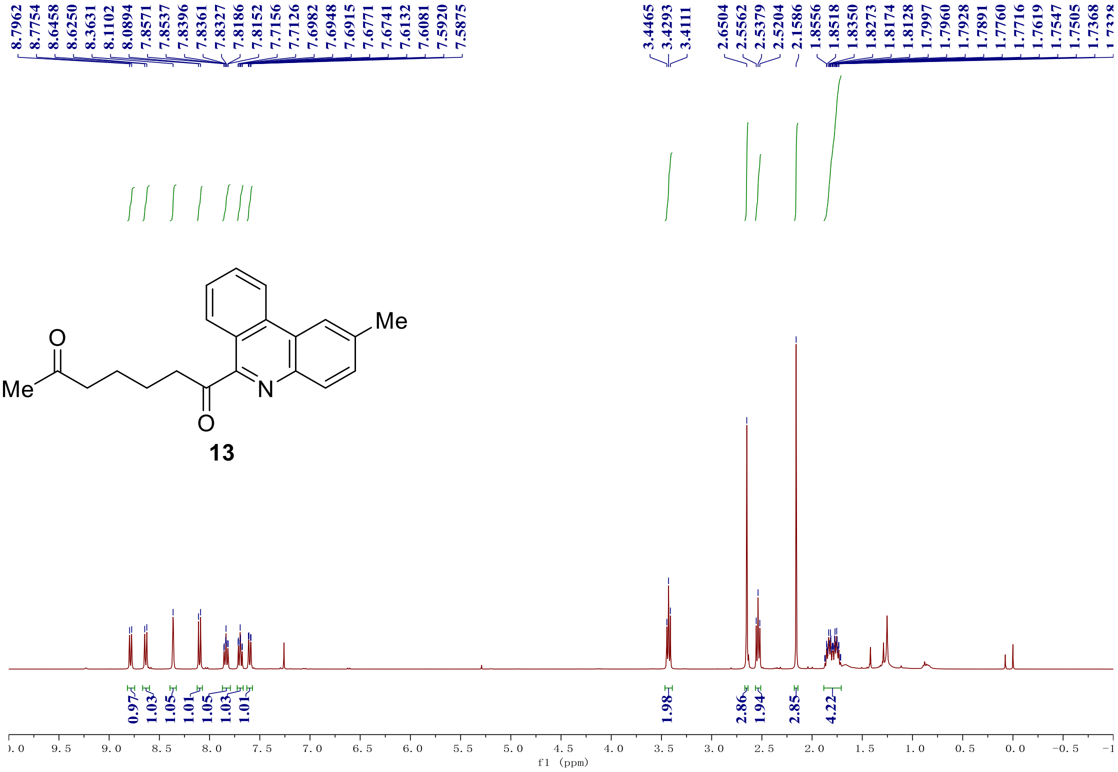

^{1}H NMR (400 MHz, $CDCl_3$) spectrum of compound **13**

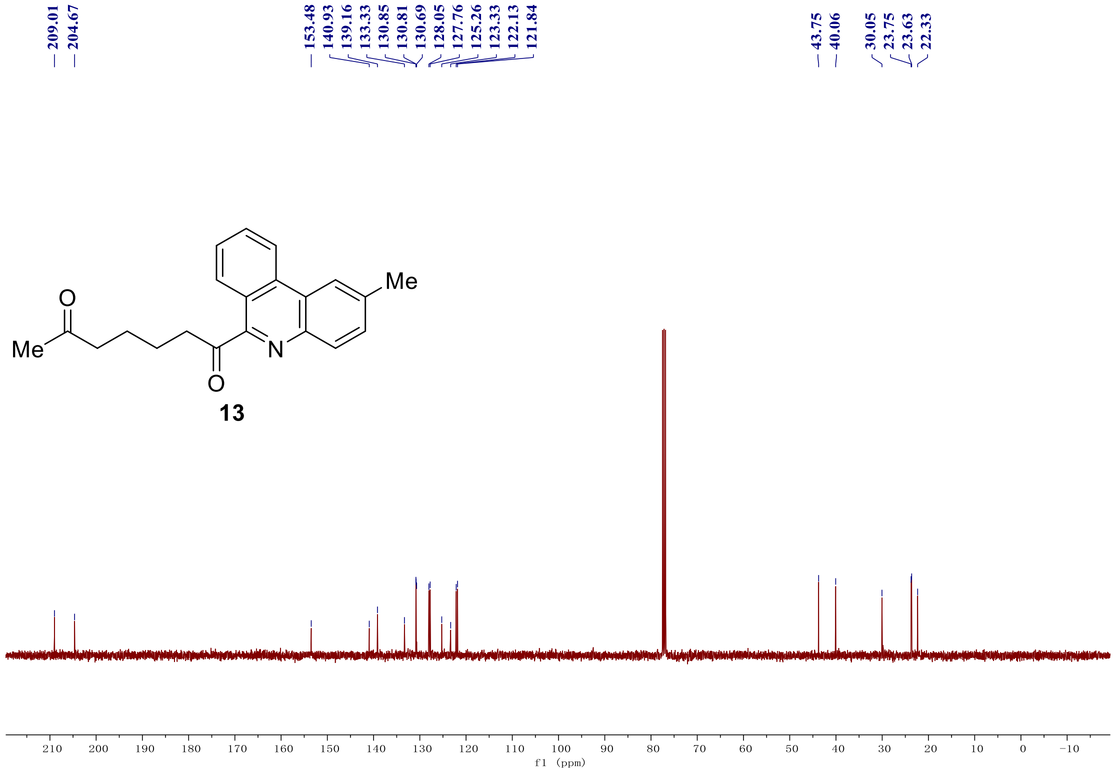

^{13}C NMR (101 MHz, $CDCl_3$) spectrum of compound **13**

9-Phenylnon-8-ene-2,7-dione (15)

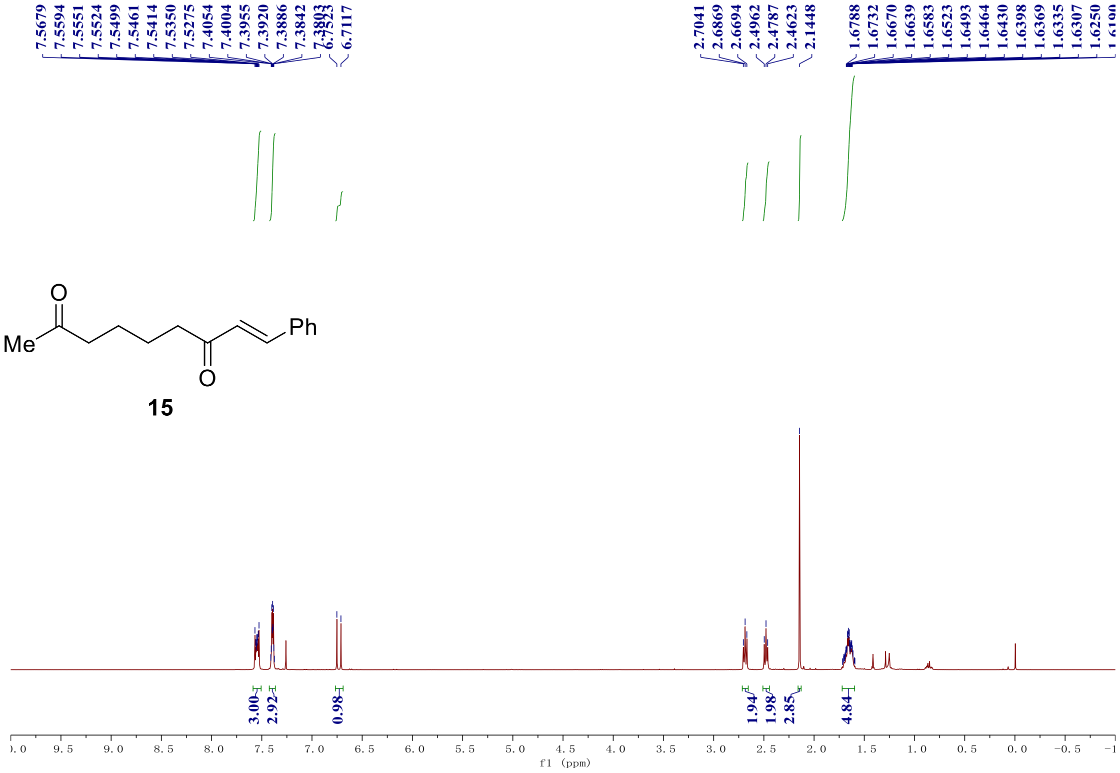

^{1}H NMR (400 MHz, $CDCl_3$) spectrum of compound **15**

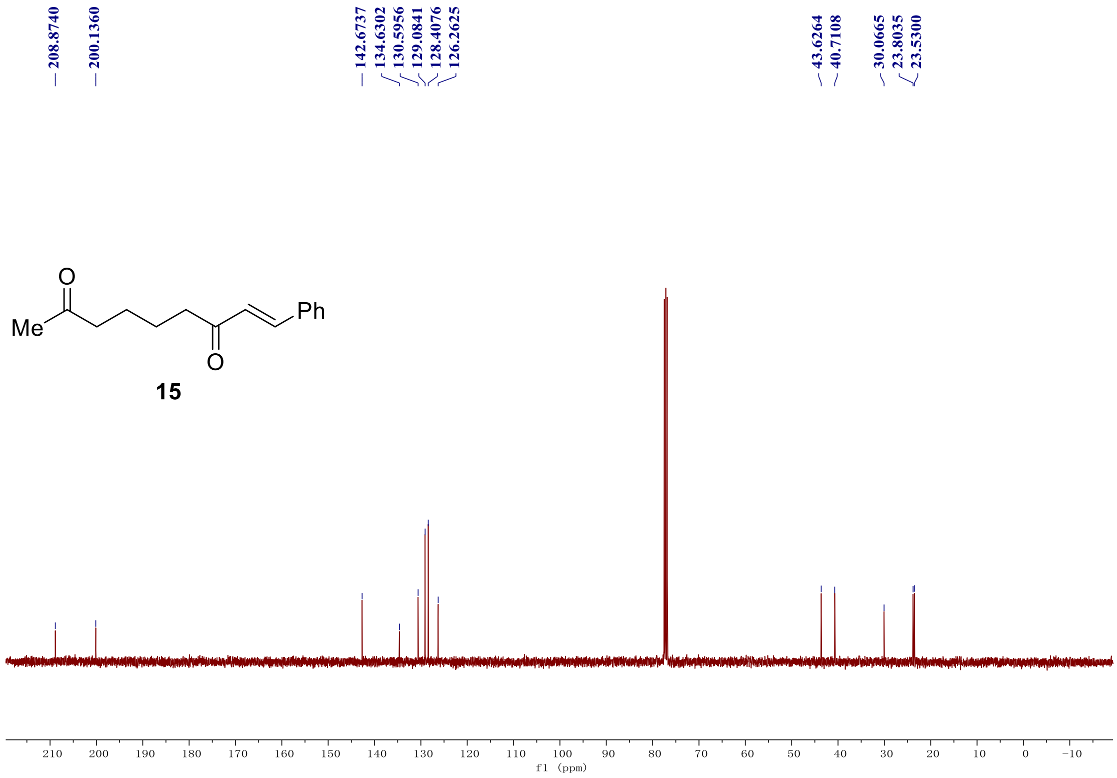

^{13}C NMR (101 MHz, $CDCl_3$) spectrum of compound **15**

9,9-Diphenylnon-8-ene-2,7-dione (17)

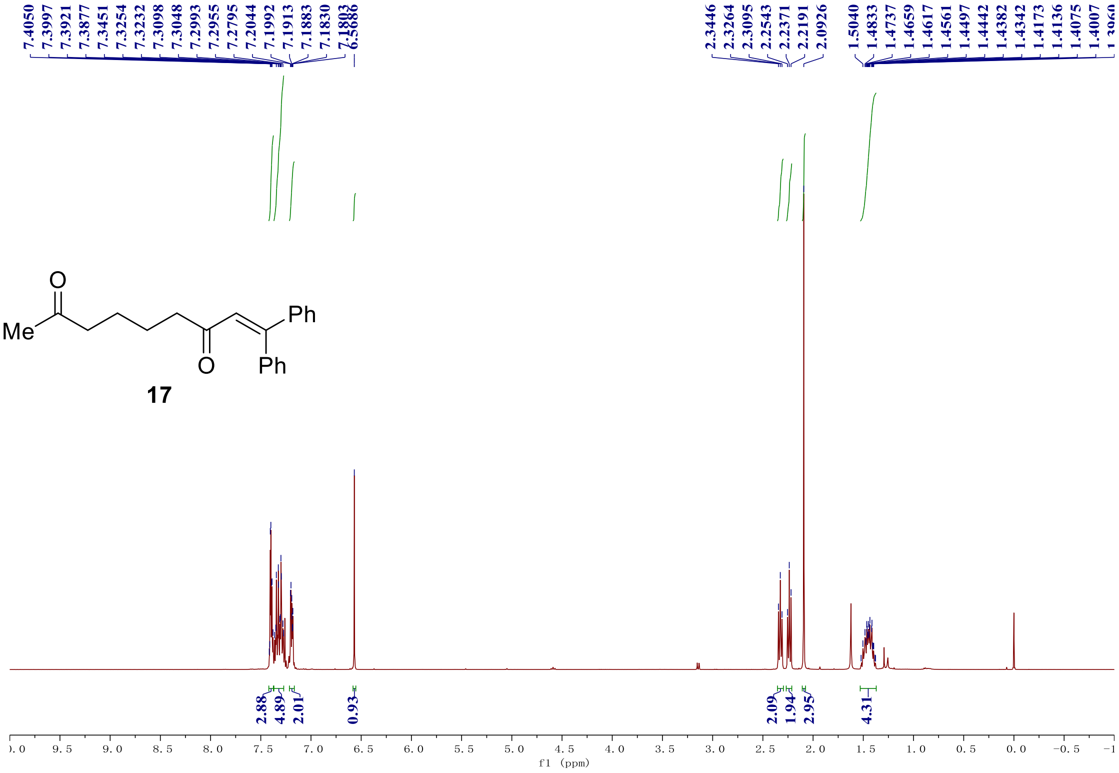

^{1}H NMR (400 MHz, CDCl$_3$) spectrum of compound **17**

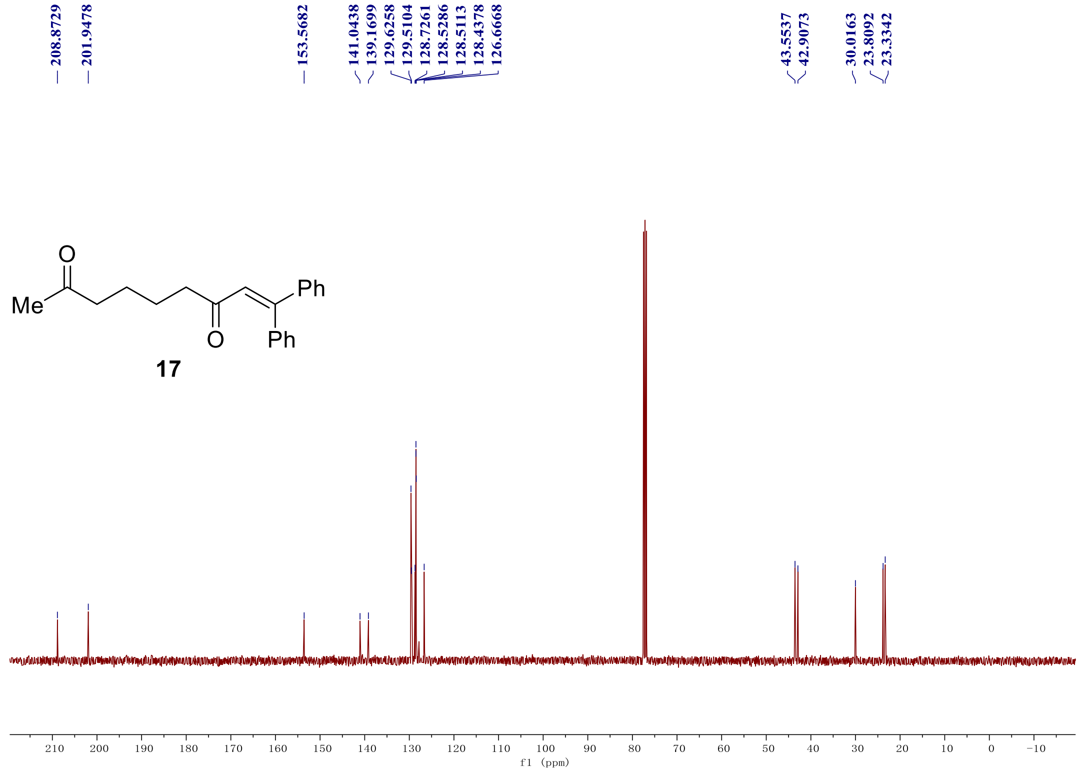

^{13}C NMR (101 MHz, CDCl$_3$) spectrum of compound **17**

Diethyl 2-(2,7-dioxo-1-phenyloctyl)malonate (19)

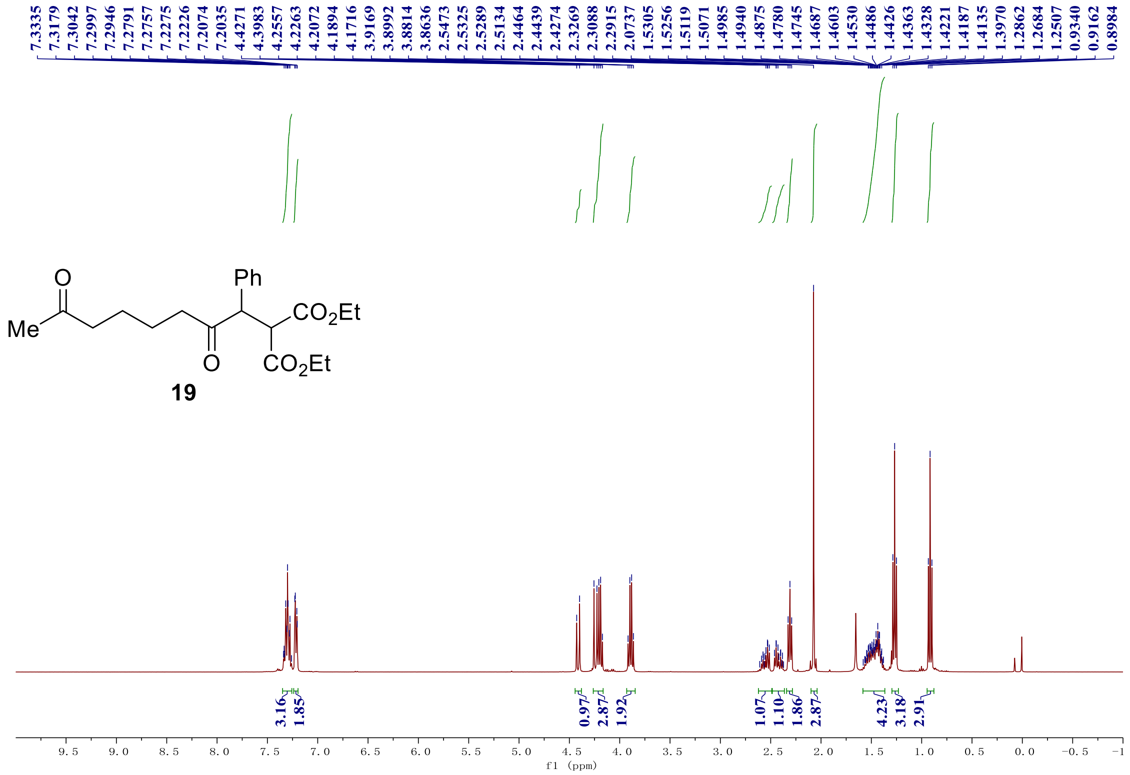

^{1}H NMR (400 MHz, $CDCl_3$) spectrum of compound **19**

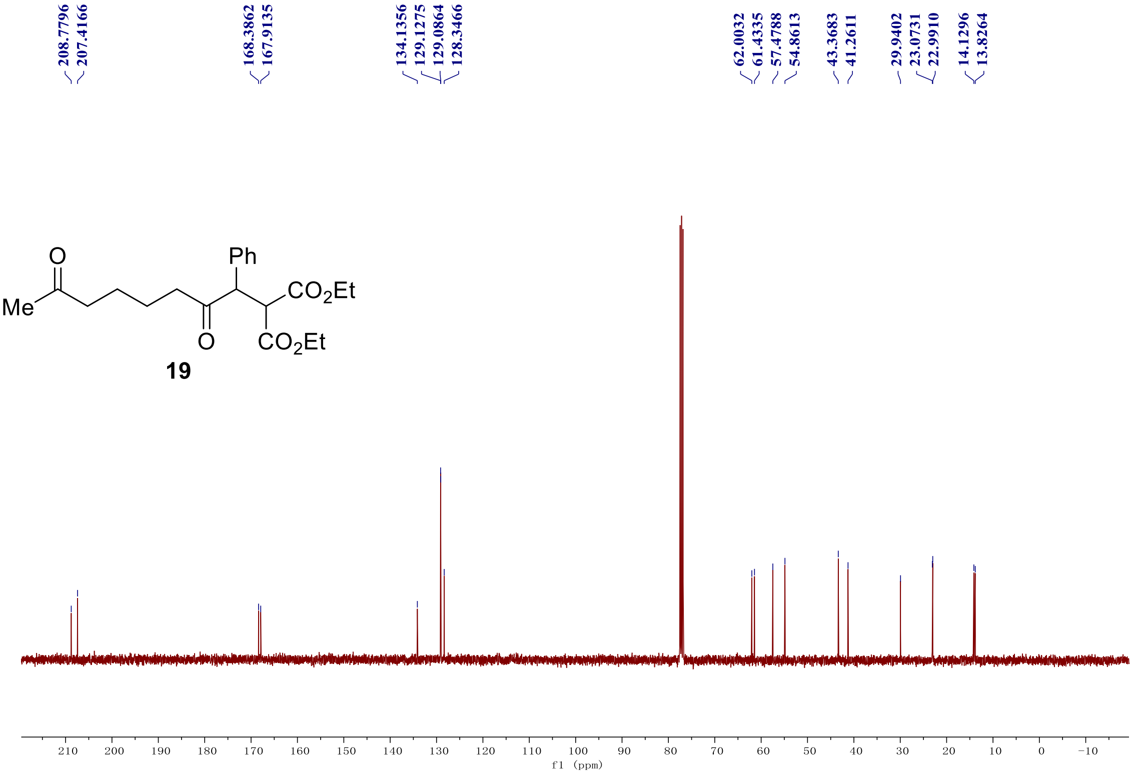

^{13}C NMR (101 MHz, $CDCl_3$) spectrum of compound **19**

9-(Phenylsulfonyl)nonane-2,7-dione (21)

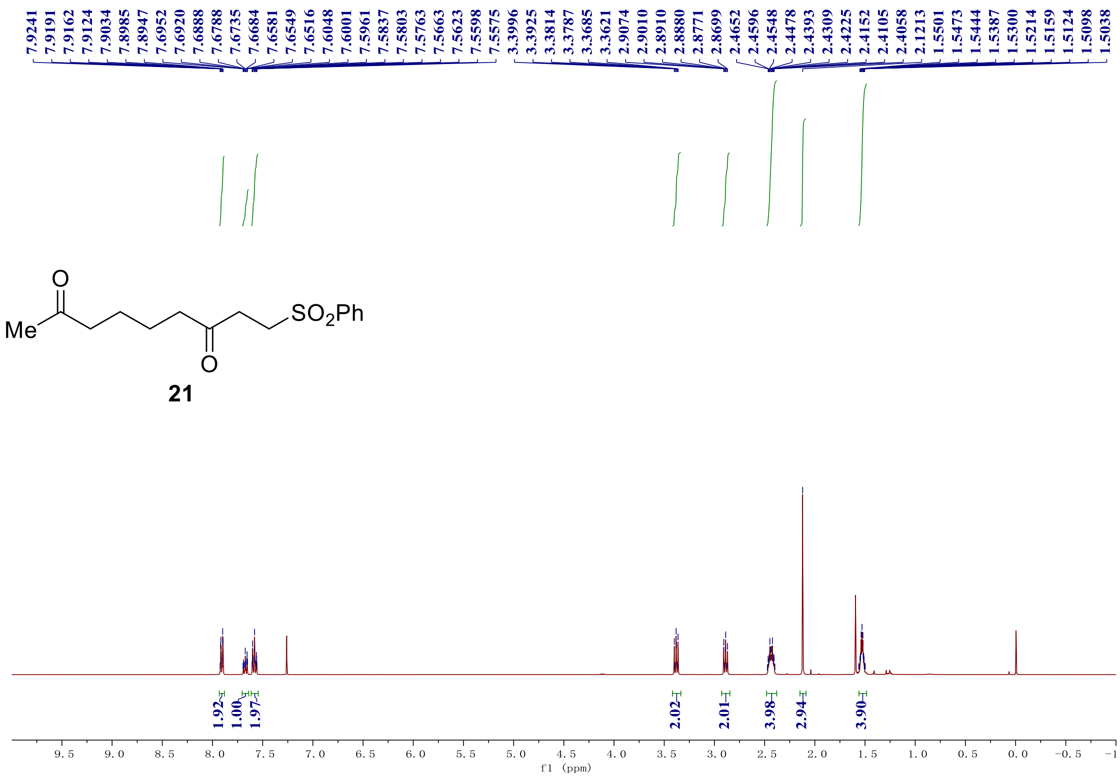

^{1}H NMR (400 MHz, $CDCl_3$) spectrum of compound **21**

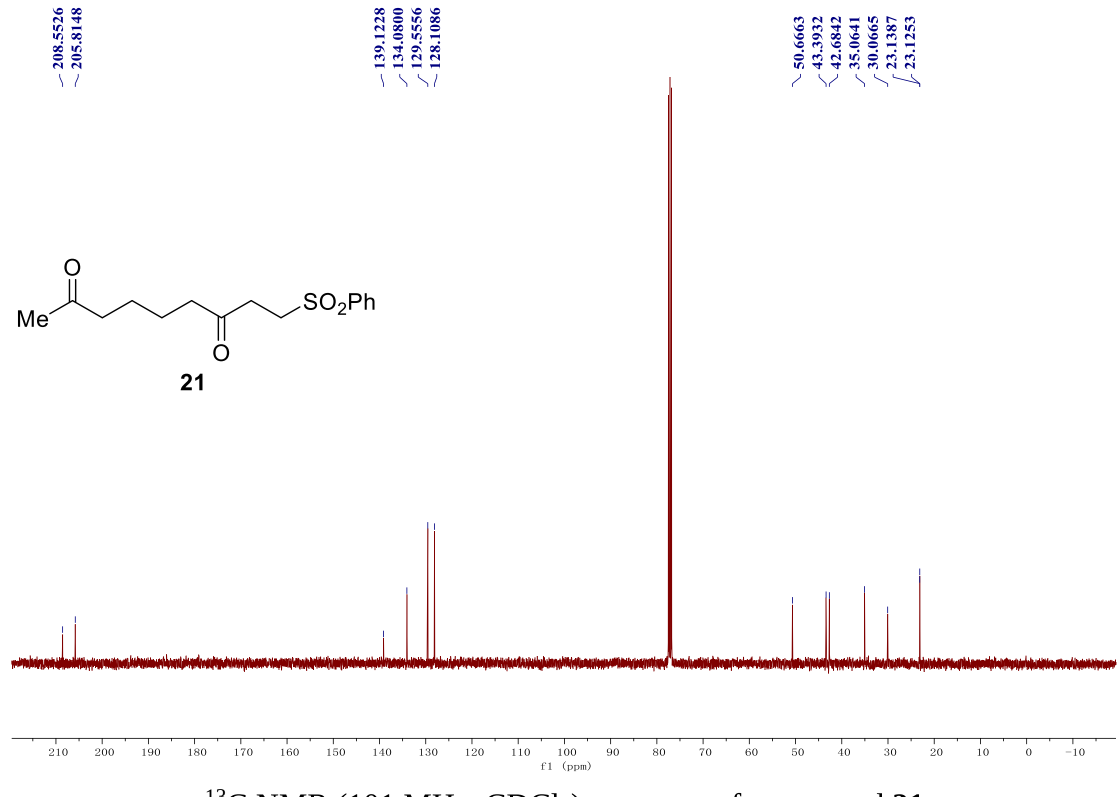

^{13}C NMR (101 MHz, $CDCl_3$) spectrum of compound **21**

N-Benzyl-6-oxoheptanamide (23)

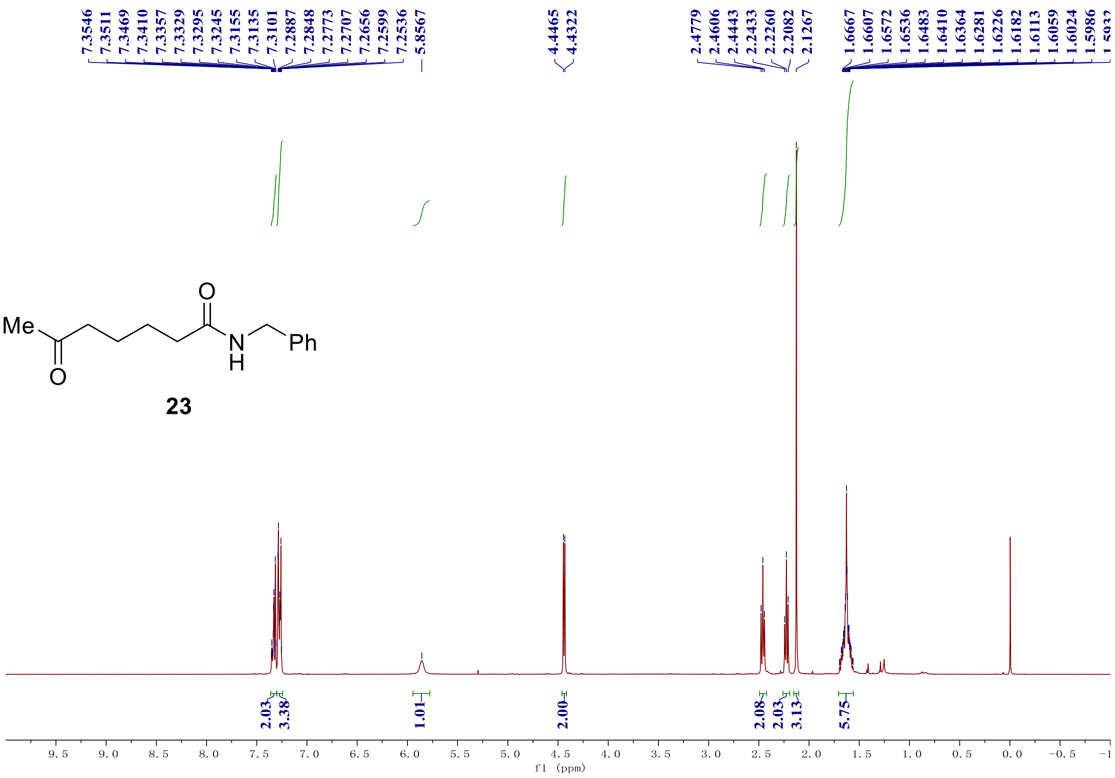

^{1}H NMR (400 MHz, $CDCl_3$) spectrum of compound **23**

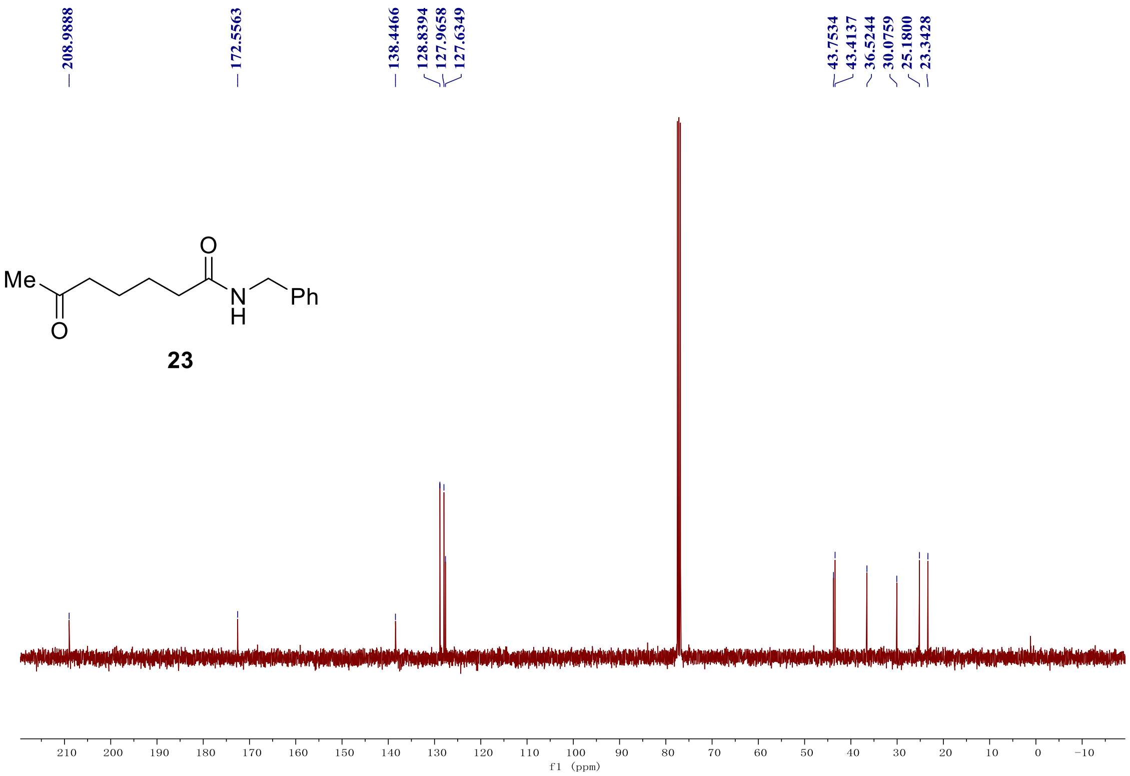

^{13}C NMR (101 MHz, $CDCl_3$) spectrum of compound **23**

Benzyl 6-oxoheptanoate (24)

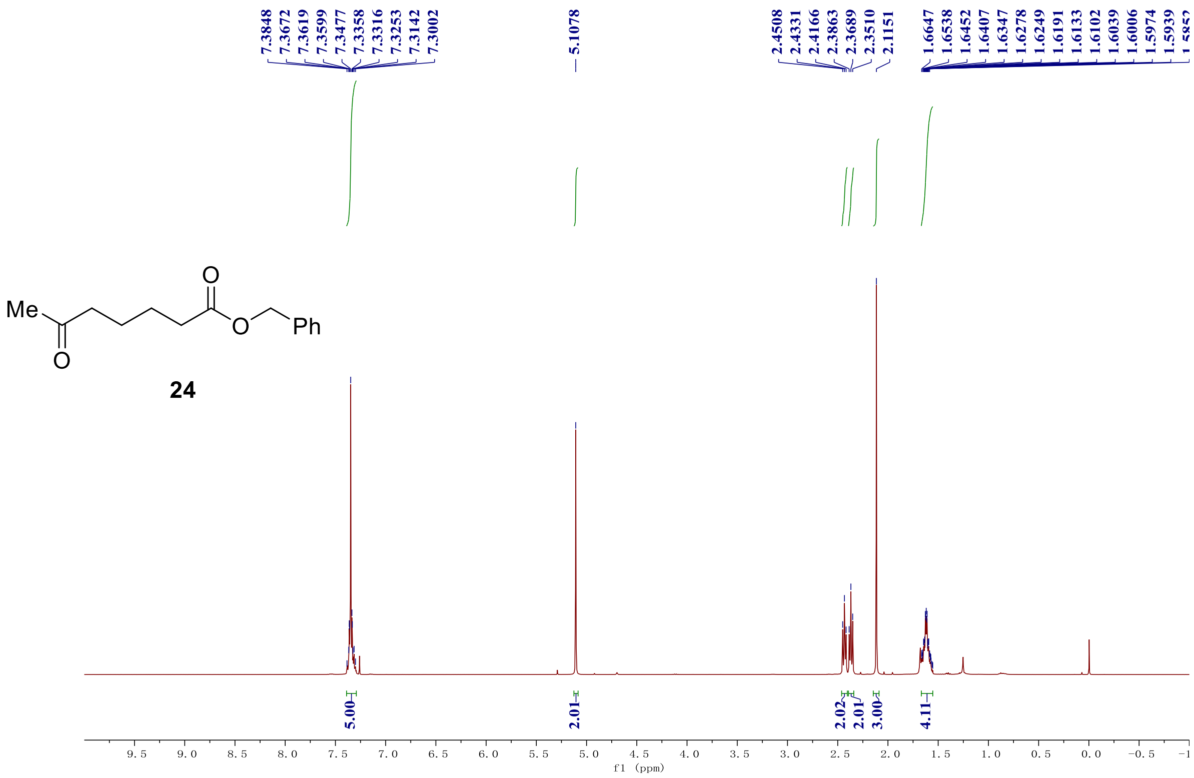

^{1}H NMR (400 MHz, $CDCl_3$) spectrum of compound **24**

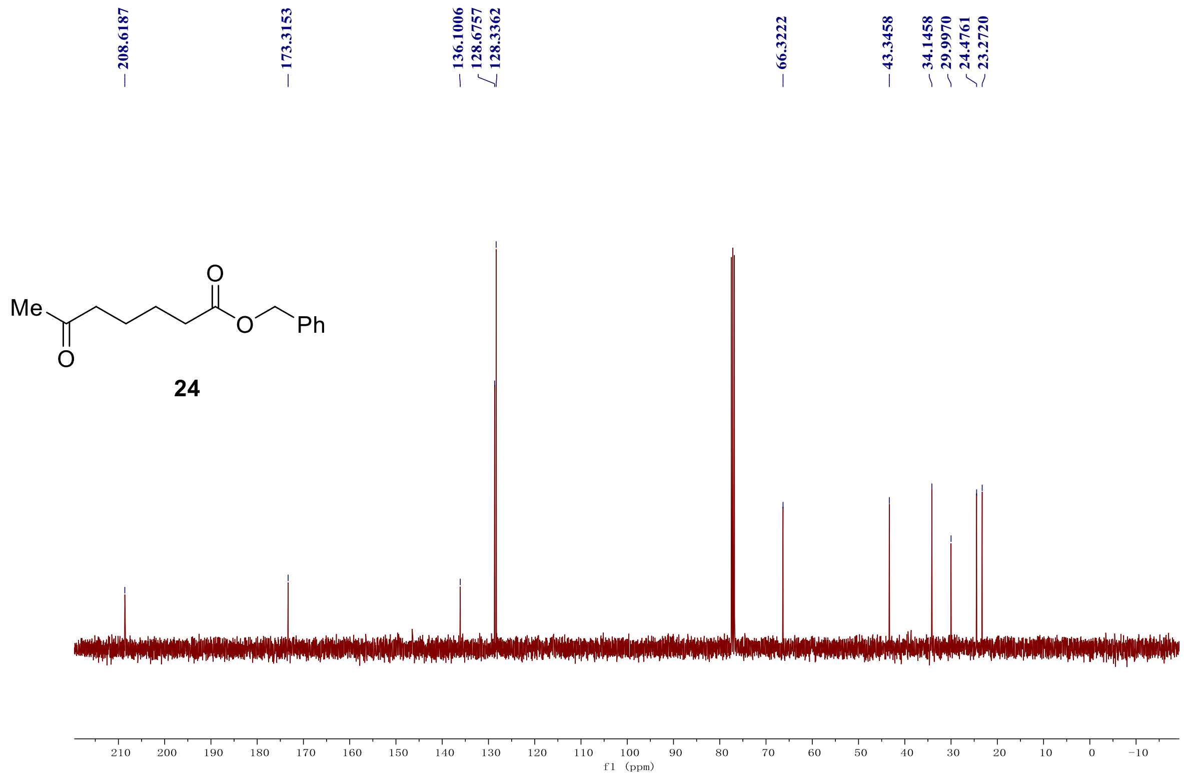

^{13}C NMR (101 MHz, $CDCl_3$) spectrum of compound **24**

1-Phenylheptane-1,6-dione (25)

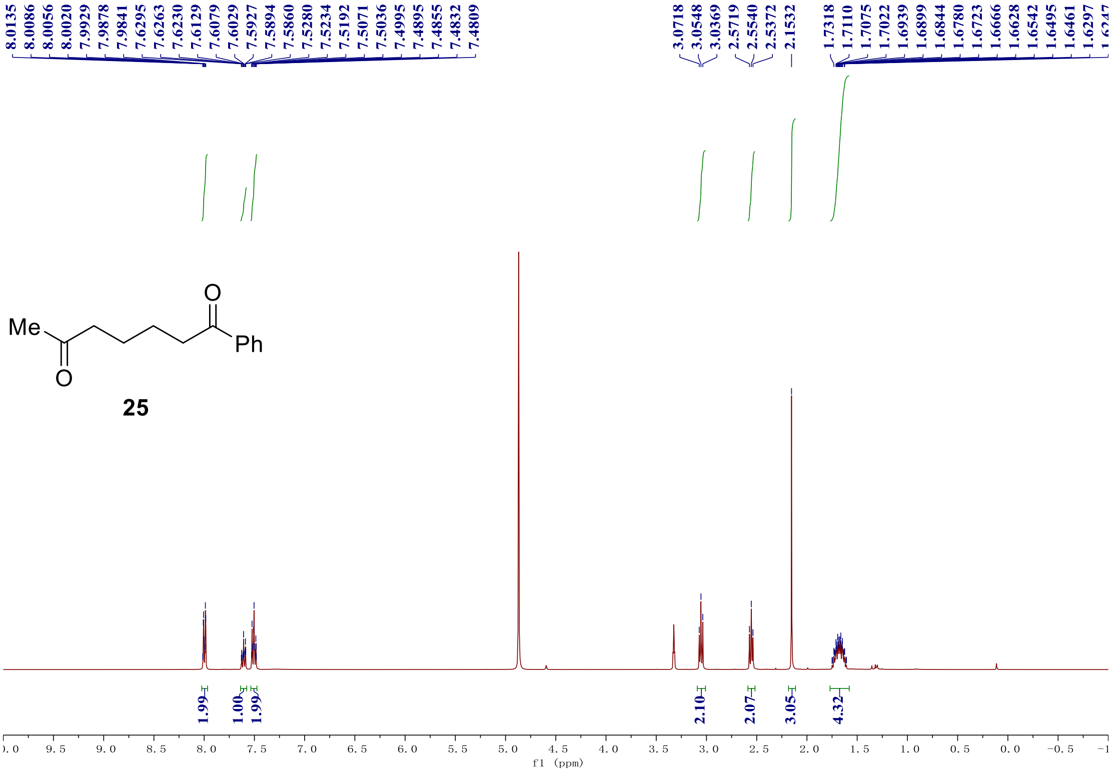

^{1}H NMR (400 MHz, CD_3OD) spectrum of compound **25**

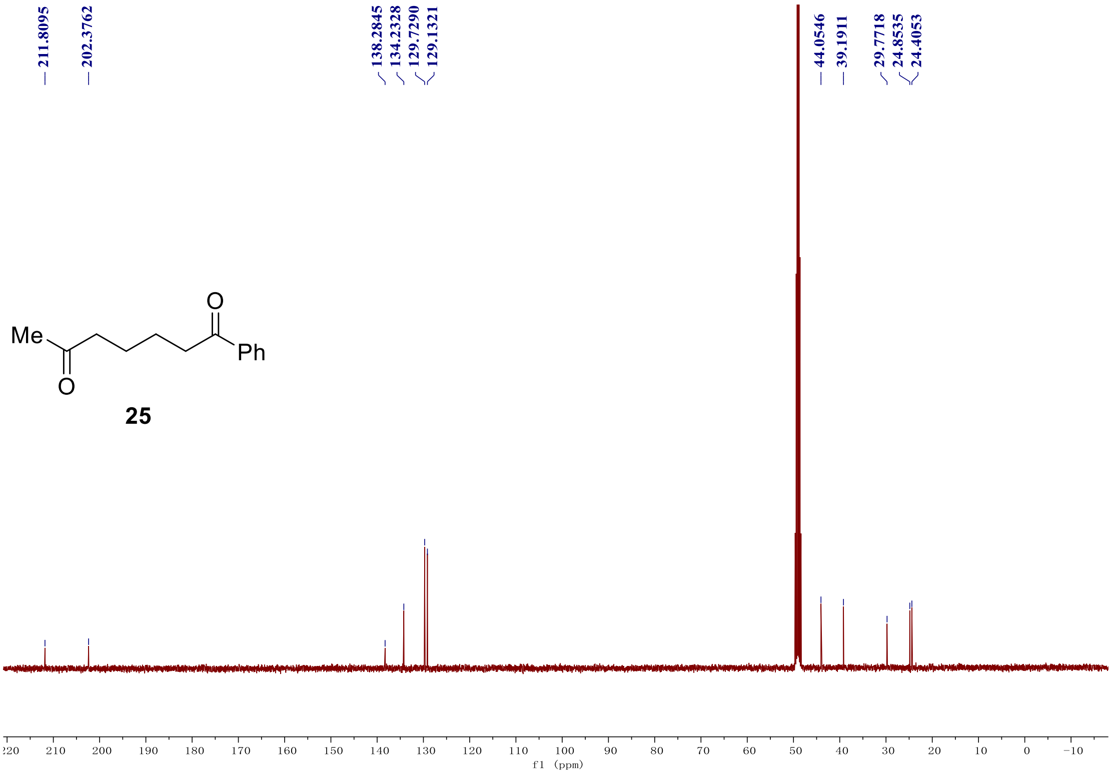

^{13}C NMR (101 MHz, CD_3OD) spectrum of compound **25**

2,2,6,6-Tetramethylpiperidin-1-yl 6-oxoheptanoate (26)

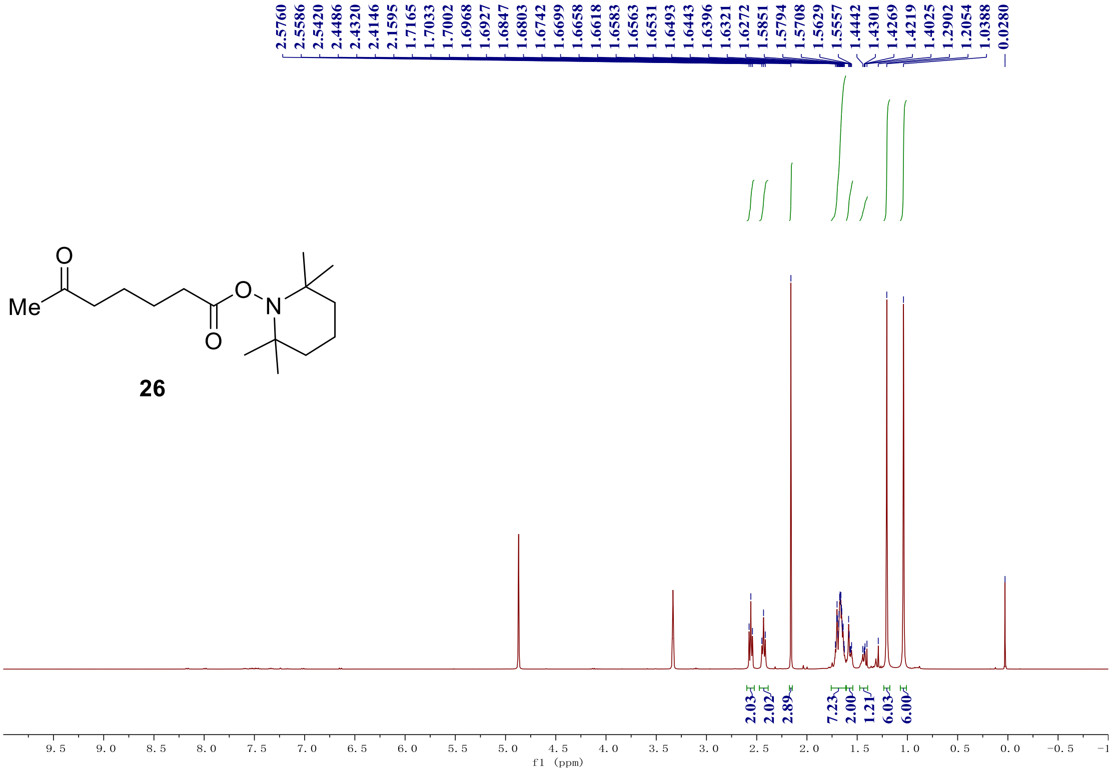

^{1}H NMR (400 MHz, CD_3OD) spectrum of compound **26**

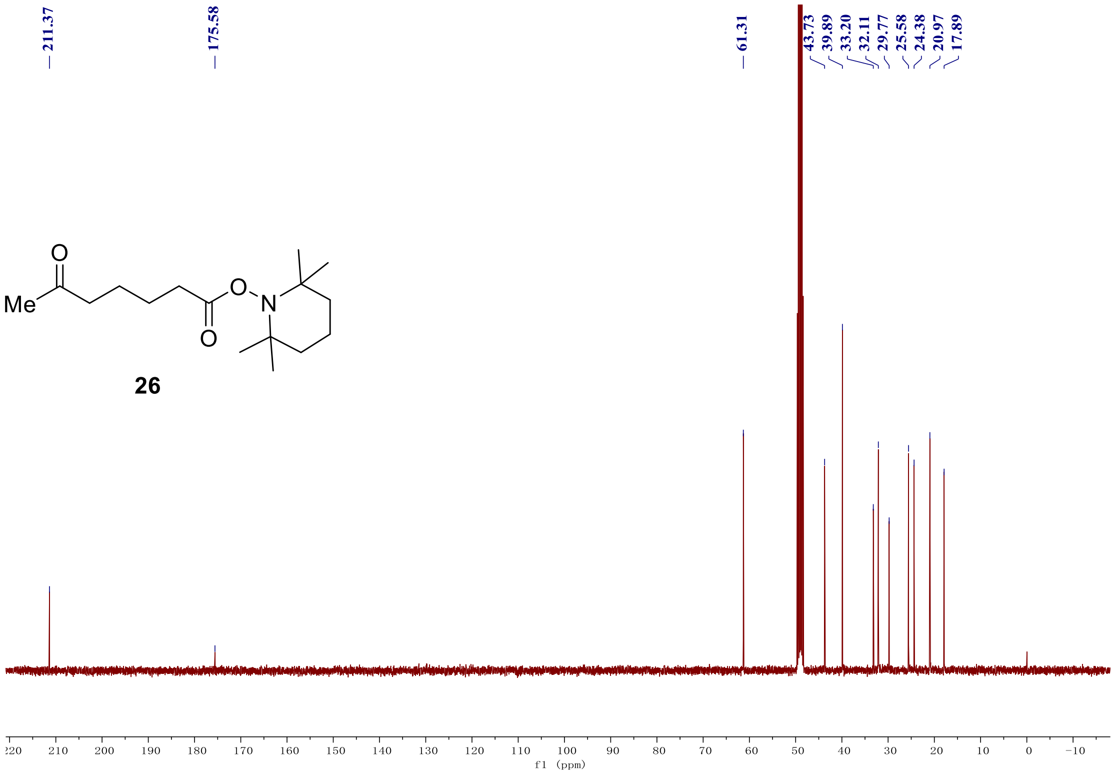

^{13}C NMR (101 MHz, CD_3OD) spectrum of compound **26**

2-Methyl-2-((triethylsilyl)peroxy)cyclohexyl acetate (32)

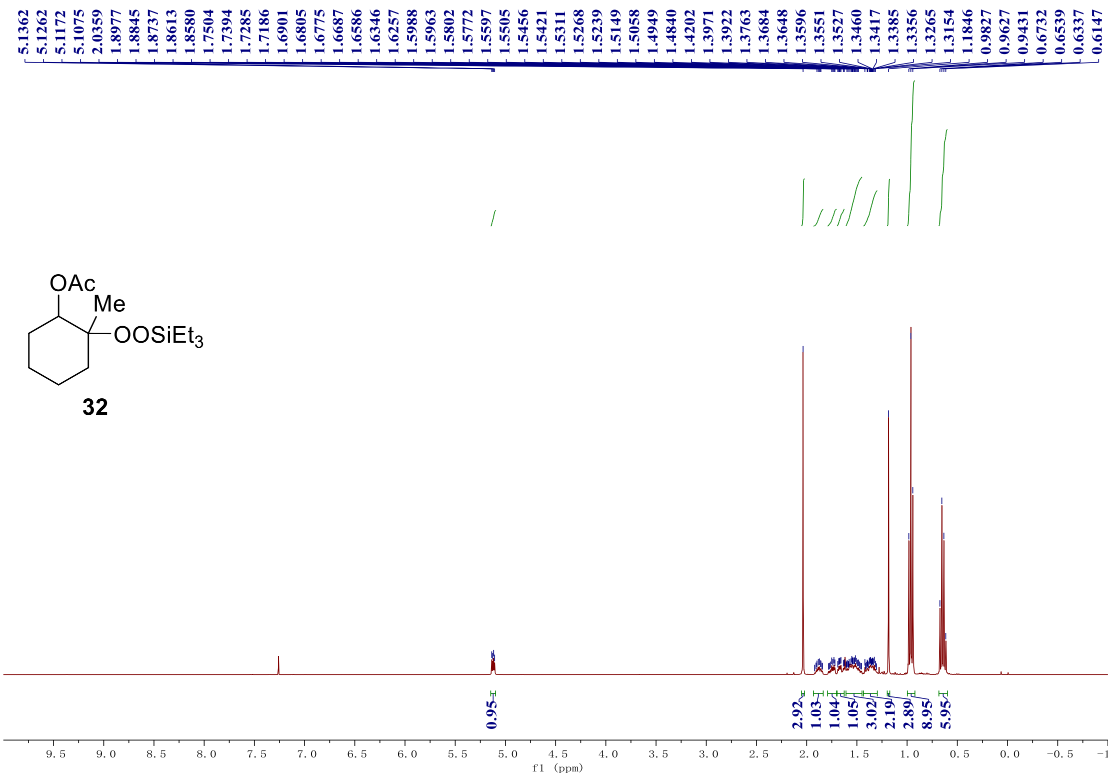

^{1}H NMR (400 MHz, $CDCl_3$) spectrum of compound **32**

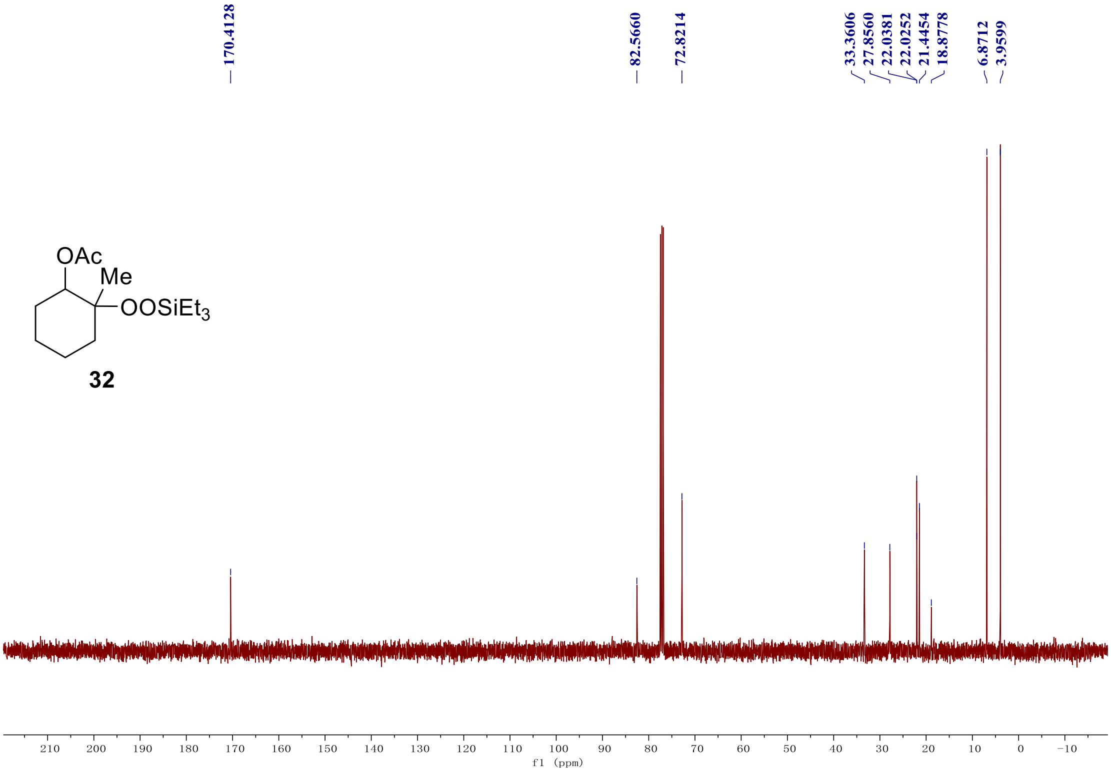

^{13}C NMR (101 MHz, $CDCl_3$) spectrum of compound **32**

1-Azido-6-oxoheptyl acetate (33)

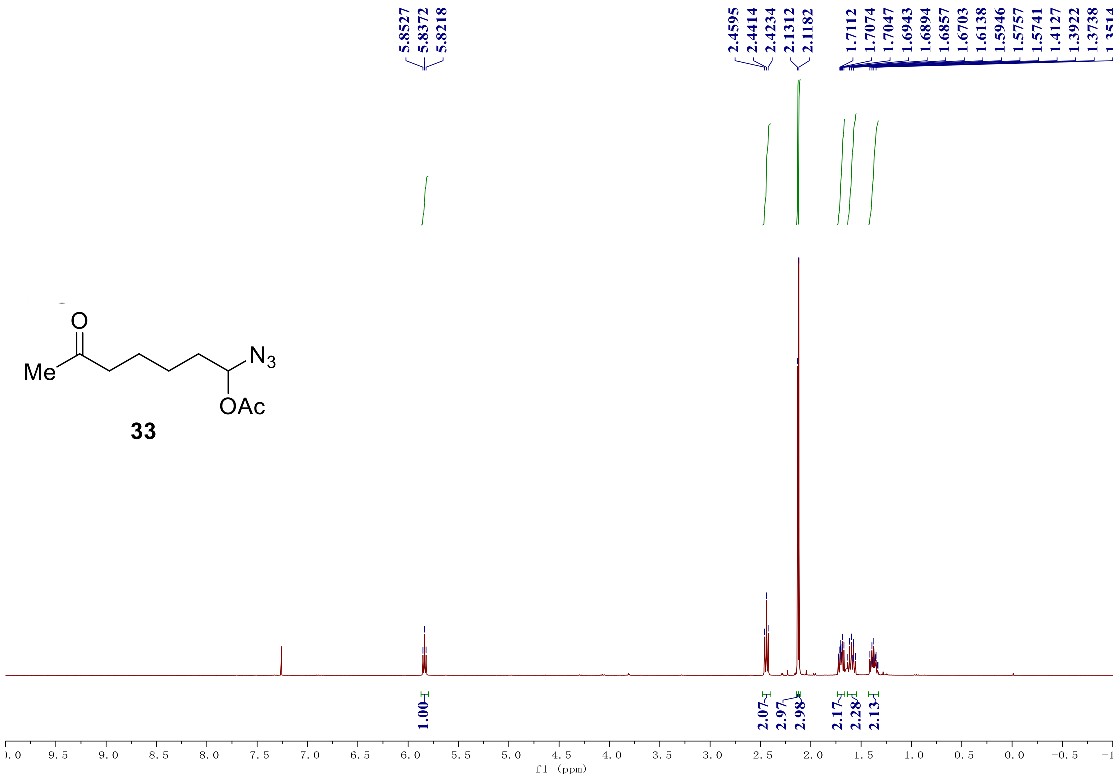

^{1}H NMR (400 MHz, $CDCl_3$) spectrum of **33**

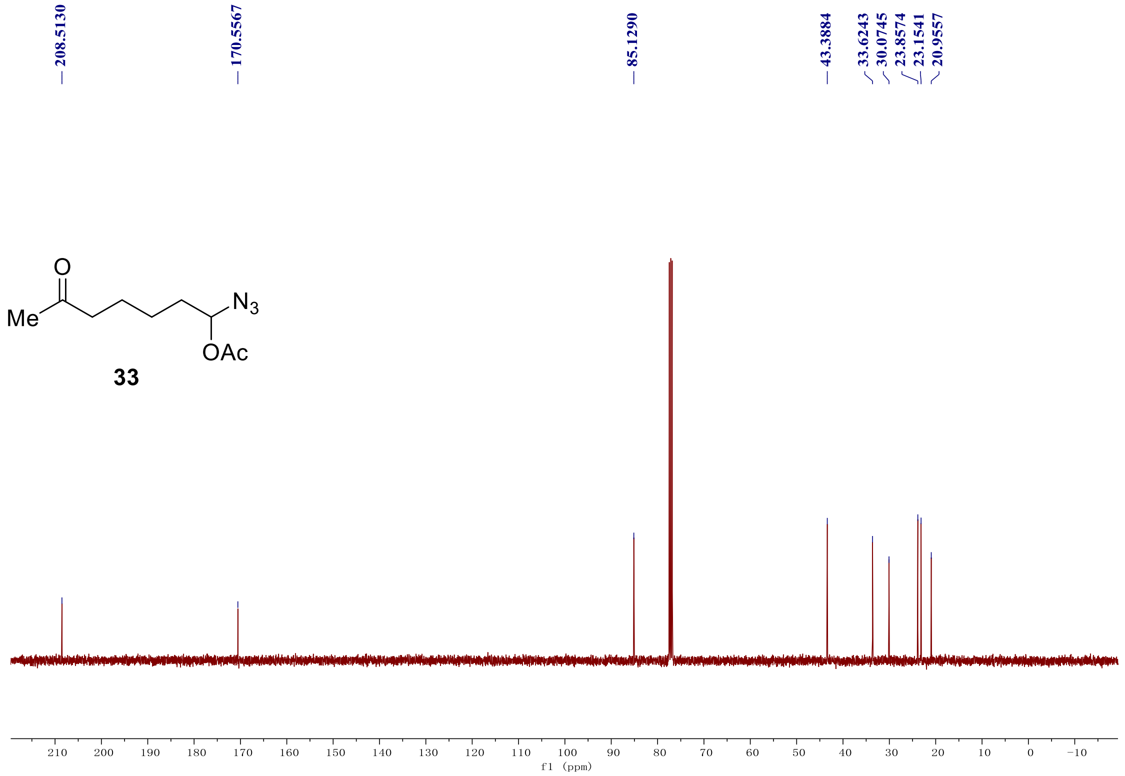

^{13}C NMR (101 MHz, $CDCl_3$) spectrum of **33**

4-Methyl-*N*-(2-methyl-2-((triethylsilyl)peroxy)cyclohexyl)benzenesulfonamide (34)

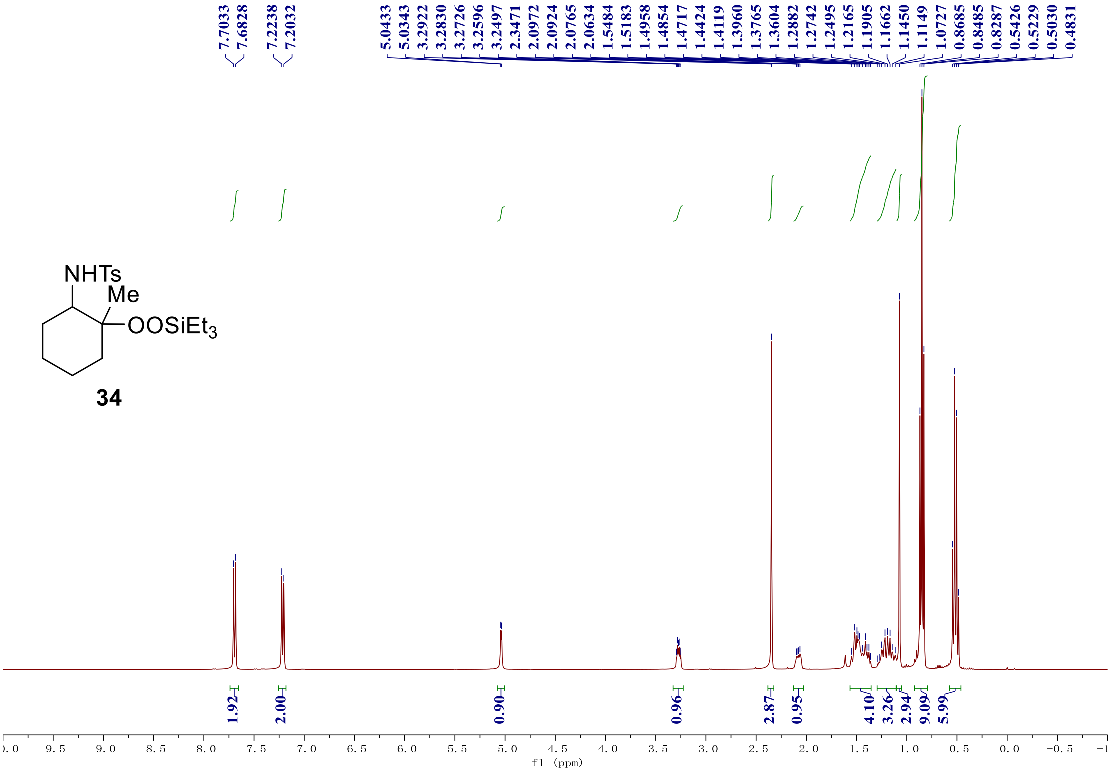

^{1}H NMR (400 MHz, $CDCl_3$) spectrum of compound **34**

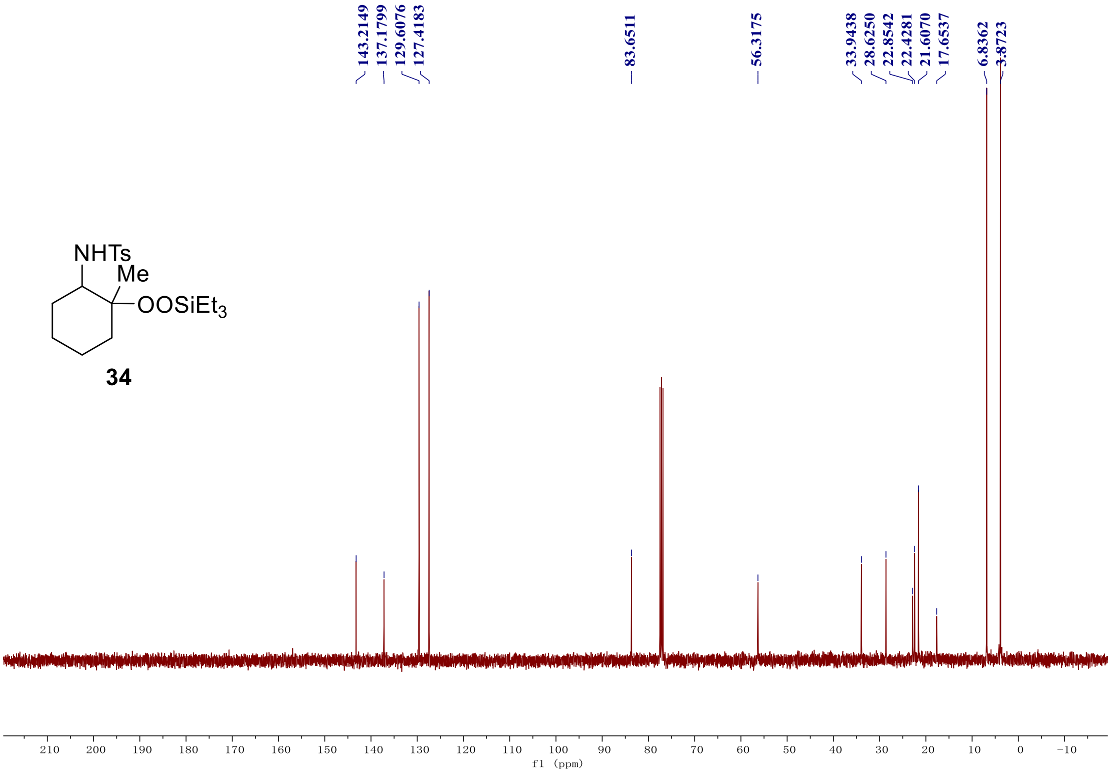

^{13}C NMR (101 MHz, $CDCl_3$) spectrum of compound **34**

N-(1-Cyano-6-oxoheptyl)-4-methylbenzenesulfonamide (35)

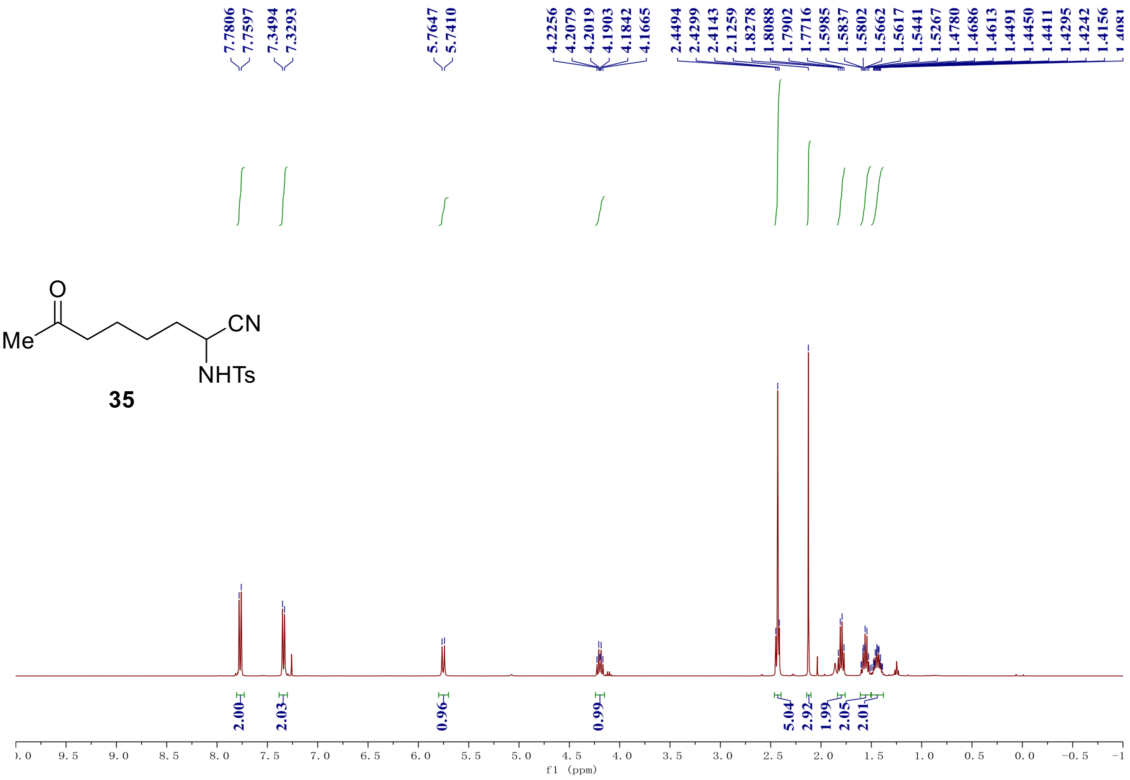

^{1}H NMR (400 MHz, $CDCl_3$) spectrum of **35**

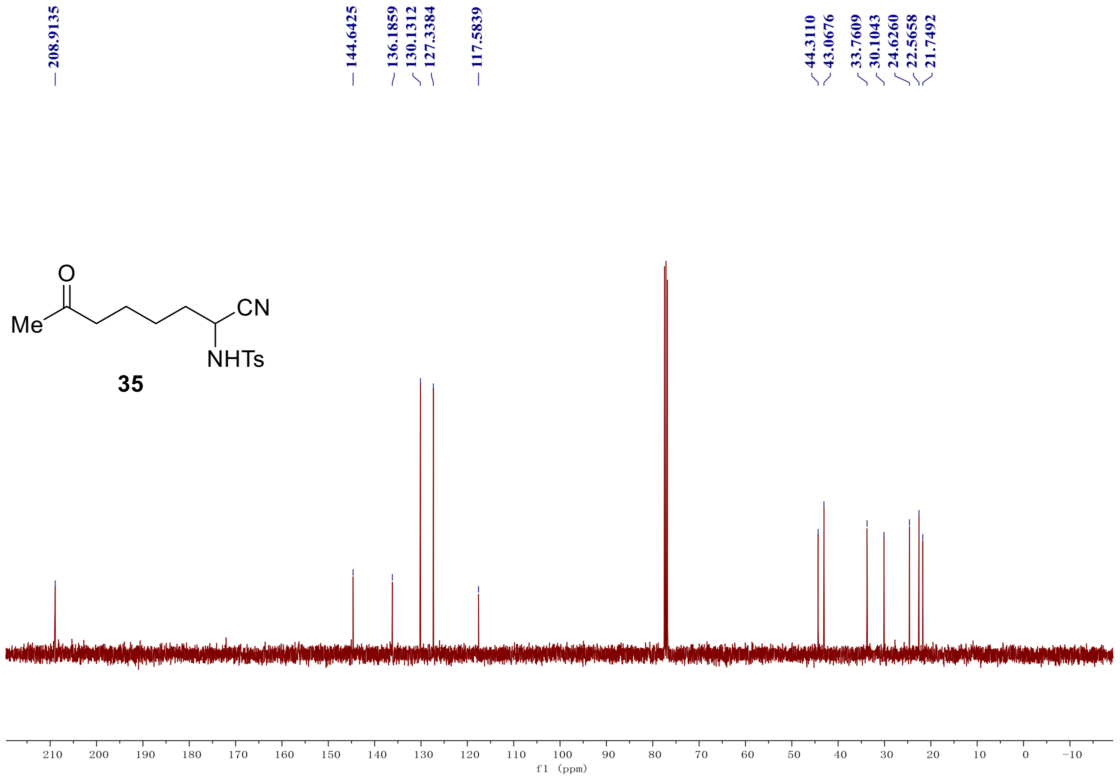

^{13}C NMR (101 MHz, $CDCl_3$) spectrum of **35**

8-Oxo-1-(phenylsulfonyl)nonan-3-yl acetate (36)

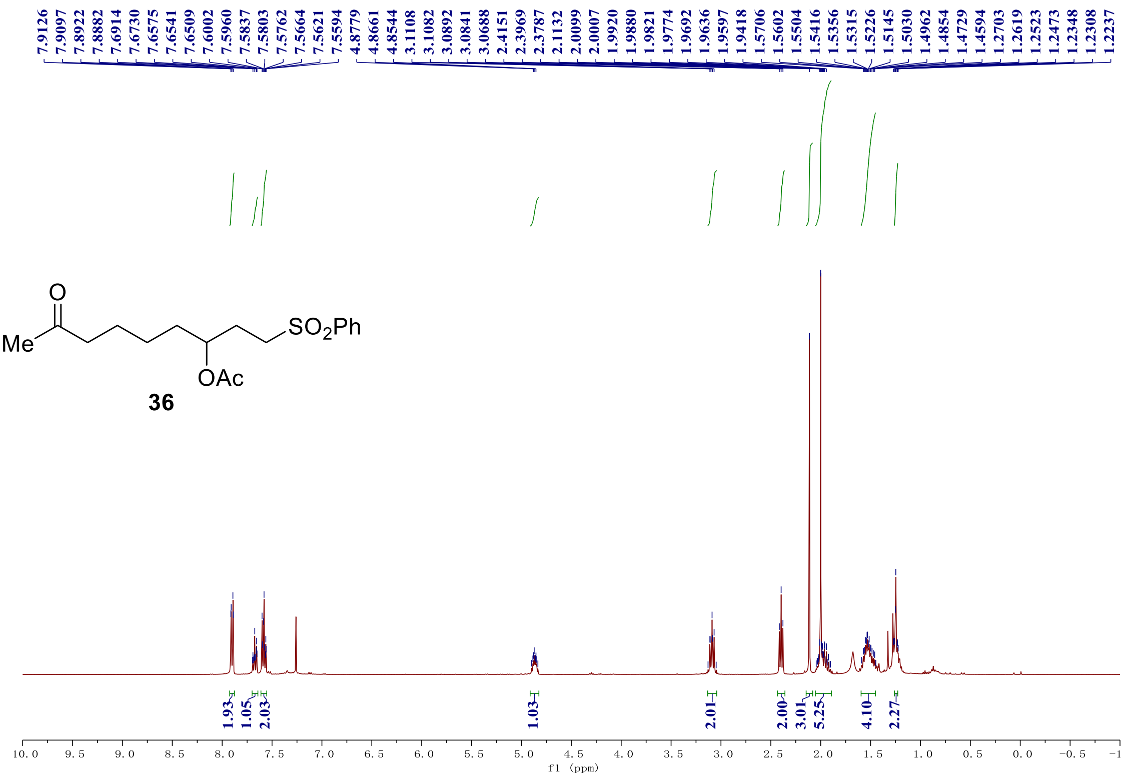

^{1}H NMR (400 MHz, $CDCl_3$) spectrum of compound **36**

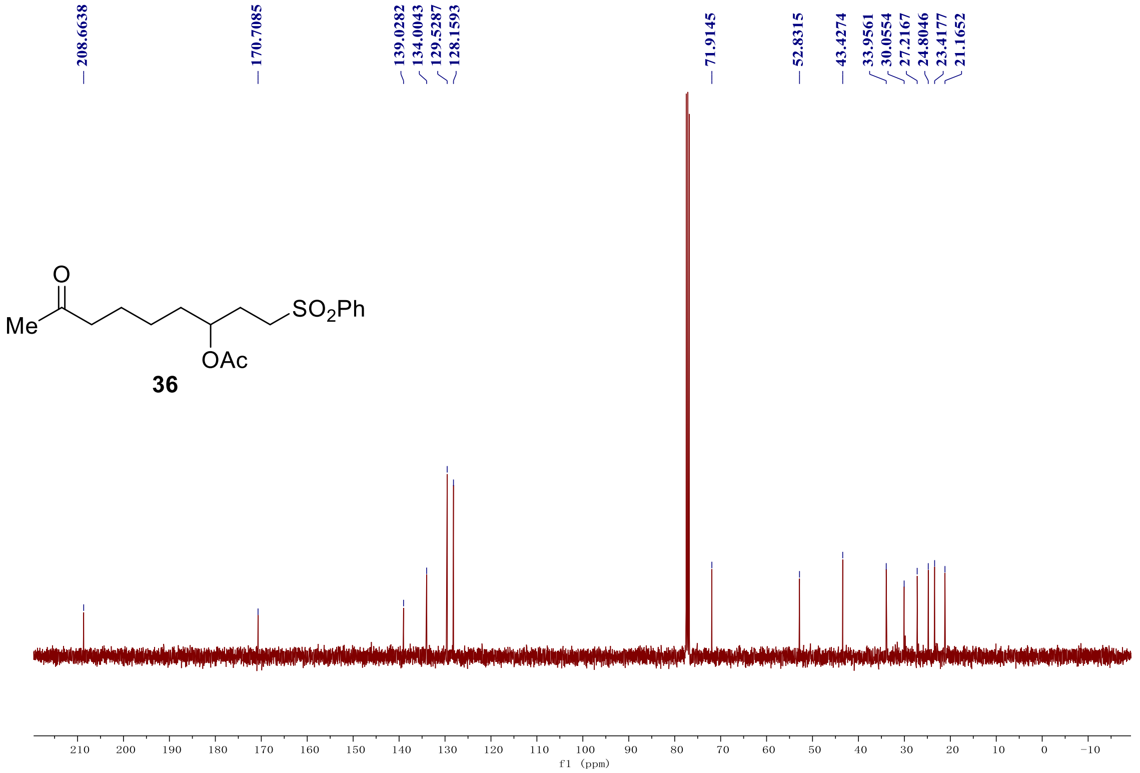

^{13}C NMR (101 MHz, $CDCl_3$) spectrum of compound **36**

4-Methyl-*N*-(8-oxo-1-(phenylsulfonyl)nonan-3-yl)benzenesulfonamide (37)

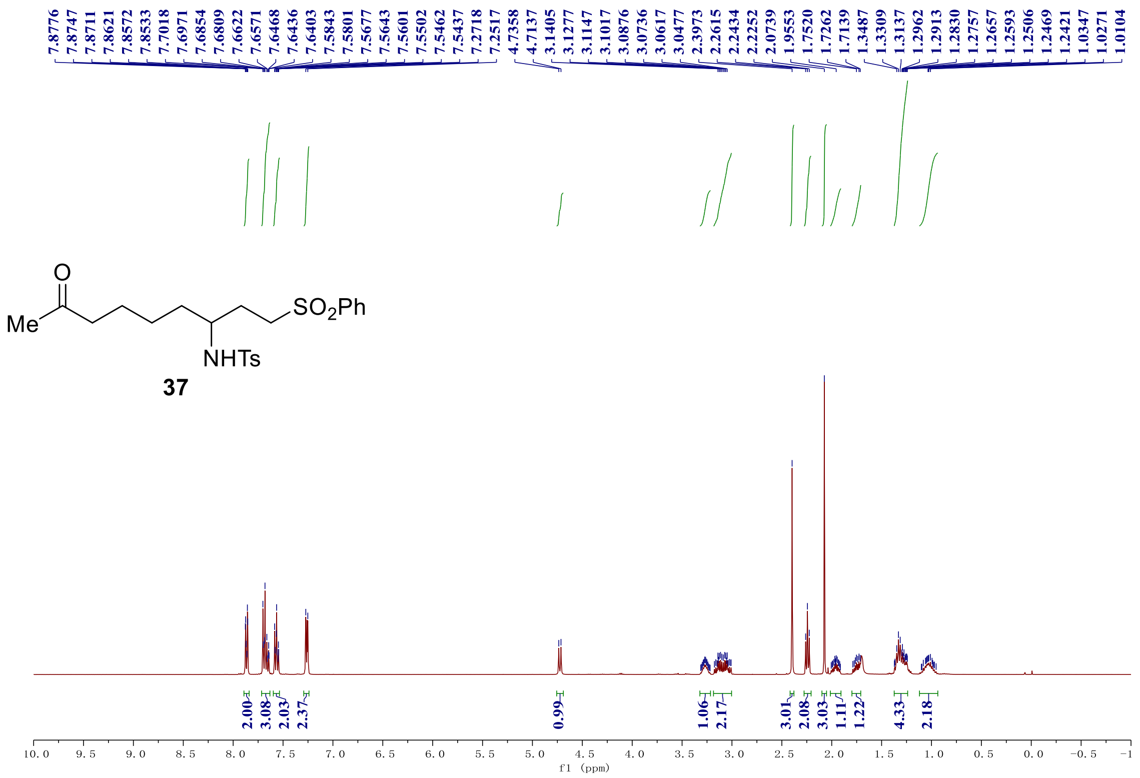

^1H NMR (400 MHz, $CDCl_3$) spectrum of compound **37**

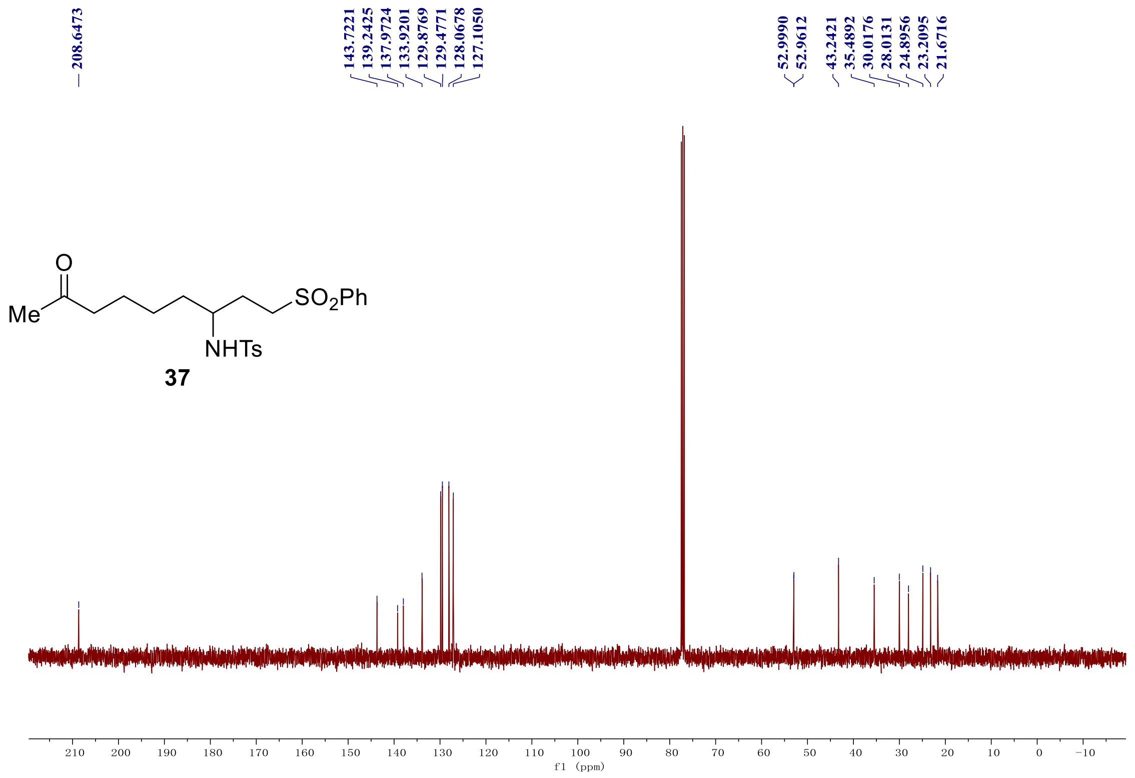

^{13}C NMR (101 MHz, $CDCl_3$) spectrum of compound **37**

2-Hydroperoxy-2-methylcyclohexan-1-one (38a)

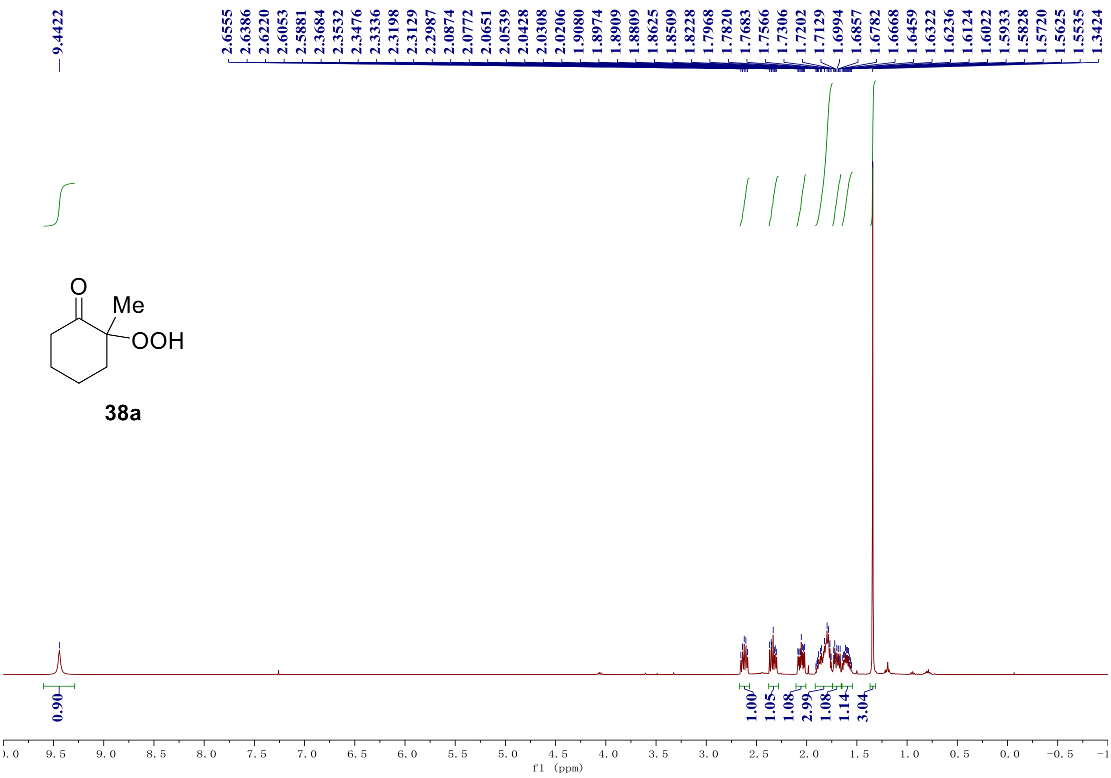

^{1}H NMR (400 MHz, $CDCl_3$) spectrum of compound **38a**

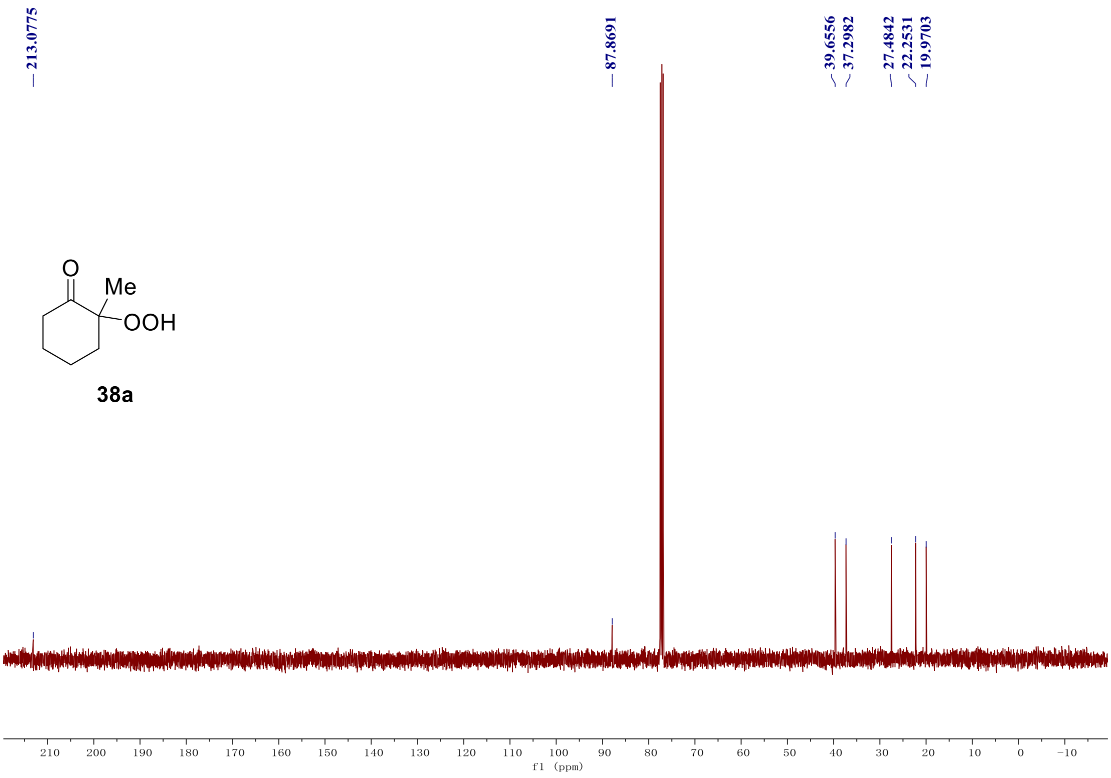

^{13}C NMR (101 MHz, $CDCl_3$) spectrum of compound **38a**

2-Hydroperoxy-2-methylcyclopentan-1-one (38b)

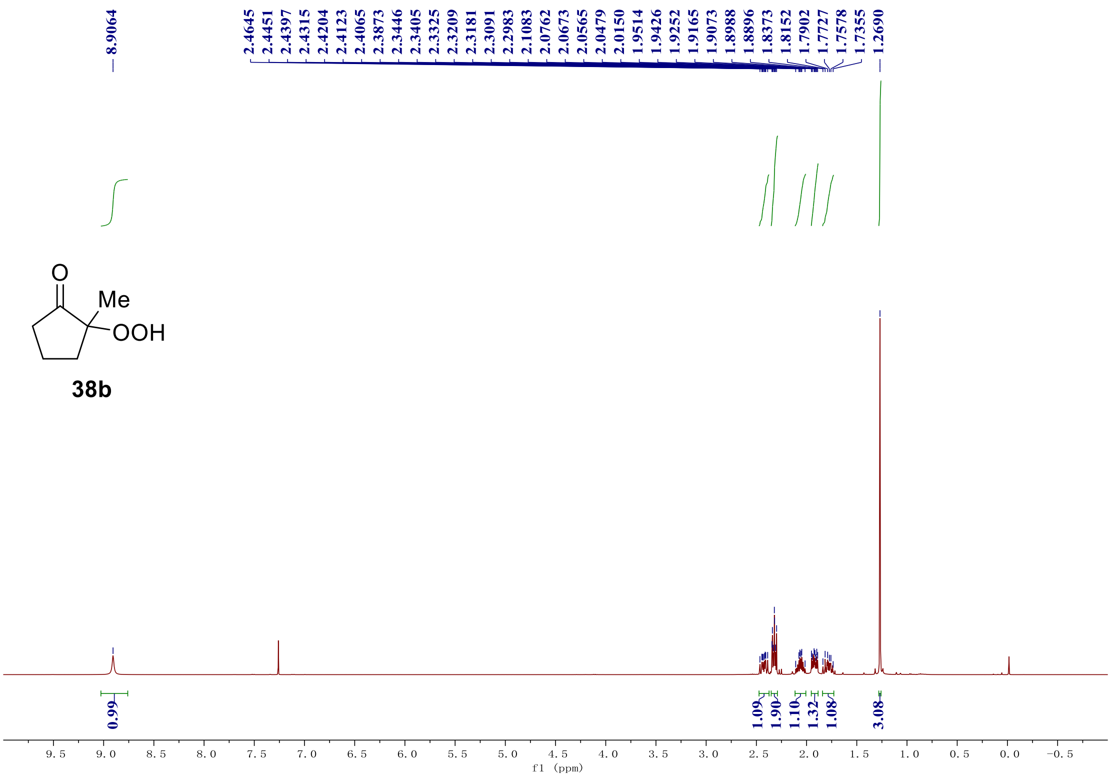

^{1}H NMR (400 MHz, $CDCl_3$) spectrum of compound **38b**

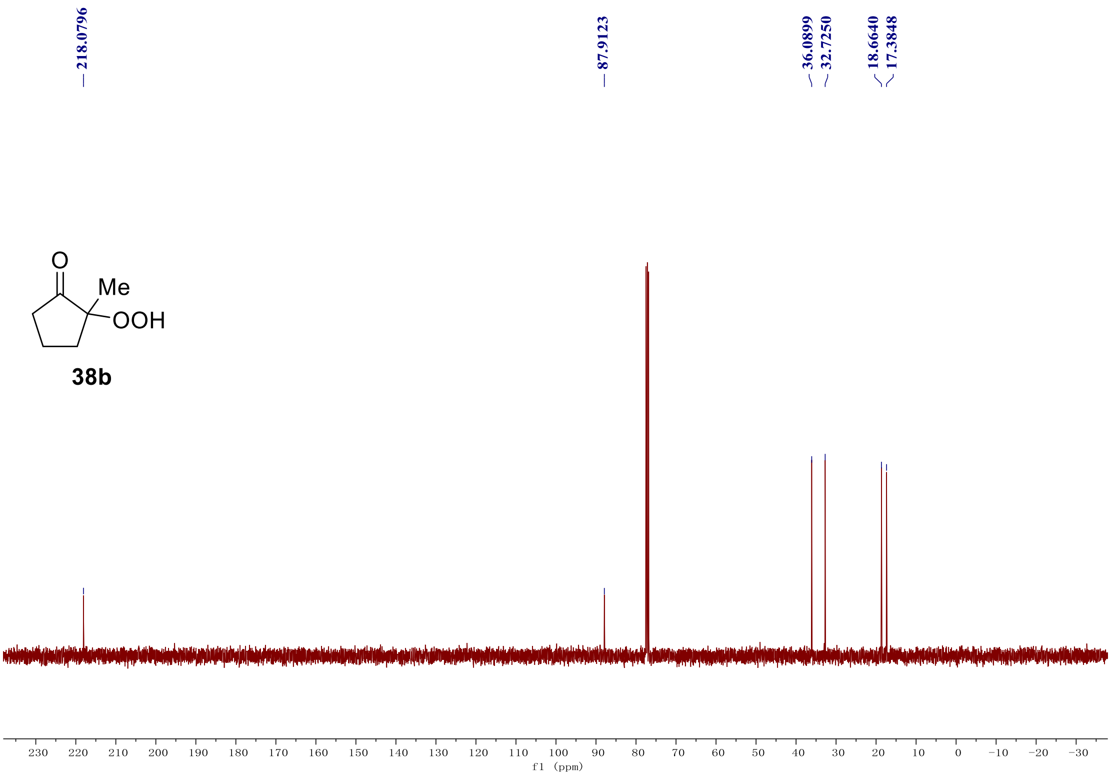

^{13}C NMR (101 MHz, $CDCl_3$) spectrum of compound **38b**

2-Hydroperoxy-2-methylcycloheptan-1-one (38c)

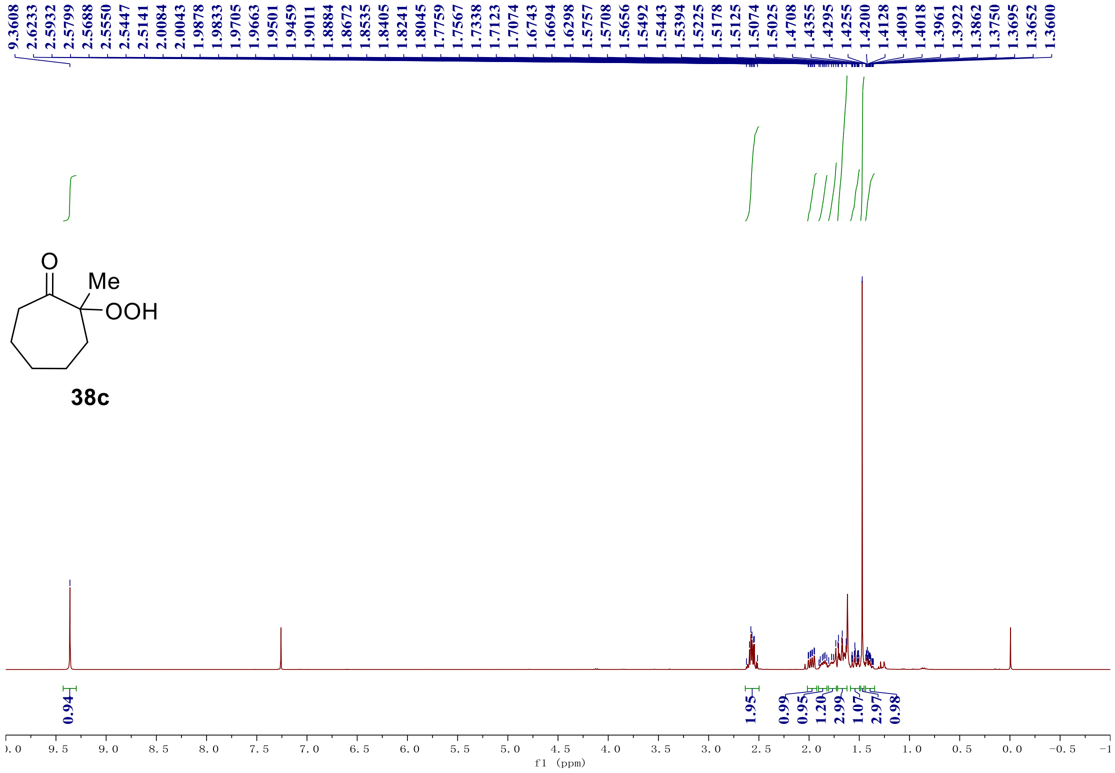

^{1}H NMR (400 MHz, $CDCl_3$) spectrum of compound **38c**

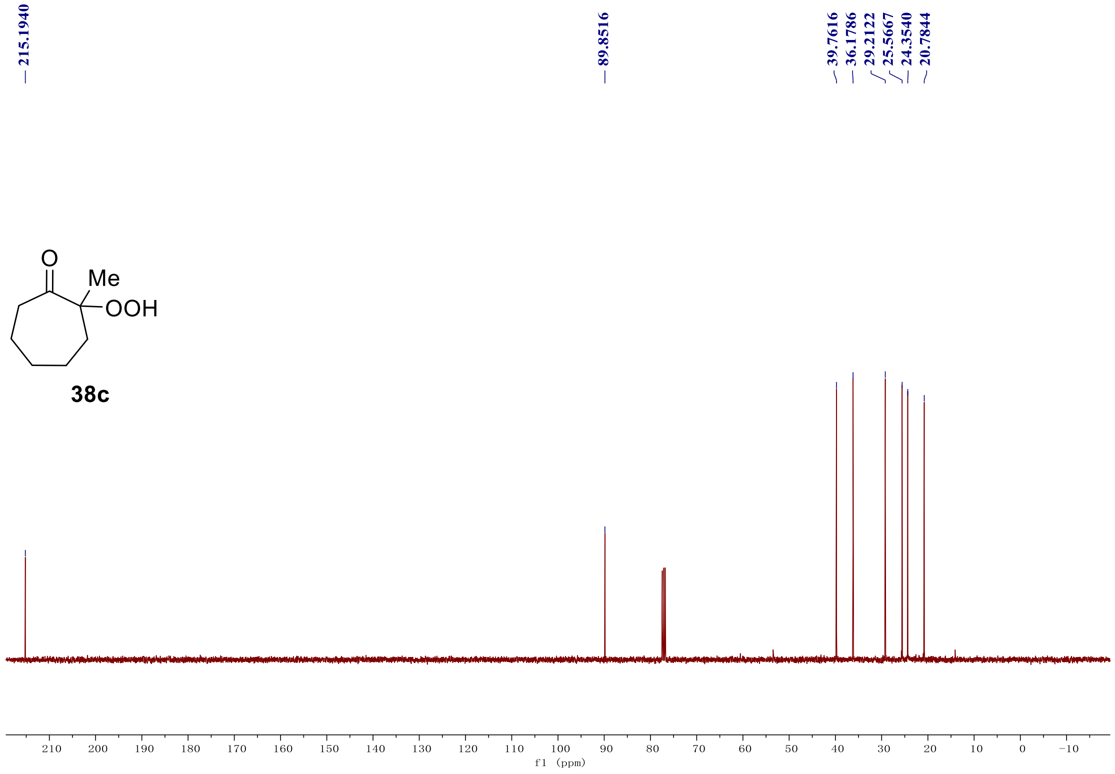

^{13}C NMR (101 MHz, $CDCl_3$) spectrum of compound **38c**

2-Hydroperoxy-2-methylcyclooctan-1-one (38d)

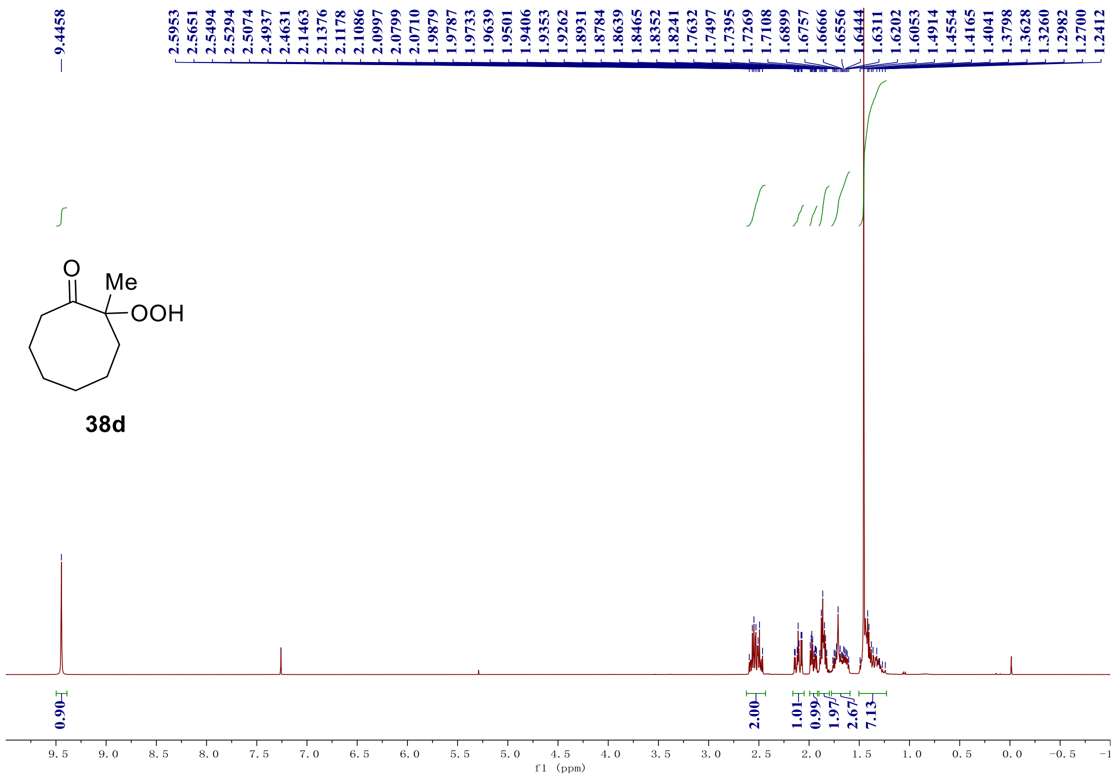

^{1}H NMR (400 MHz, $CDCl_3$) spectrum of compound **38d**

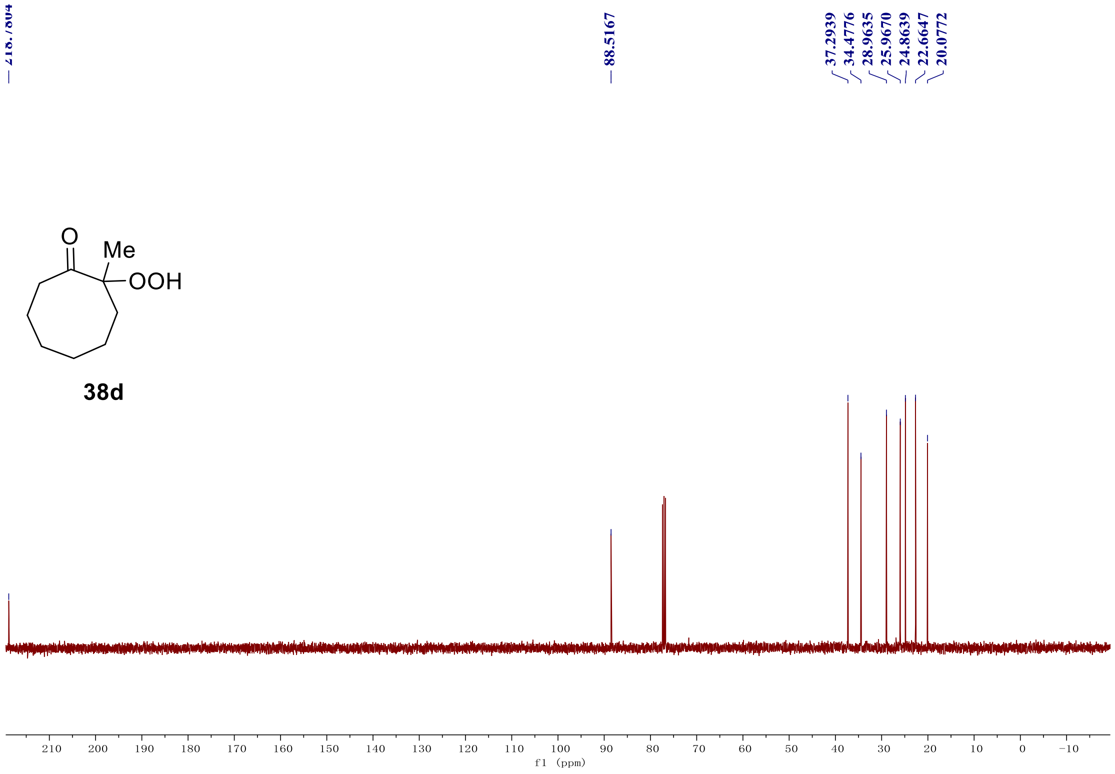

^{13}C NMR (101 MHz, $CDCl_3$) spectrum of compound **38d**

2-Hydroperoxy-2-methyl-3,4-dihydronaphthalen-1(2H)-one (38e)

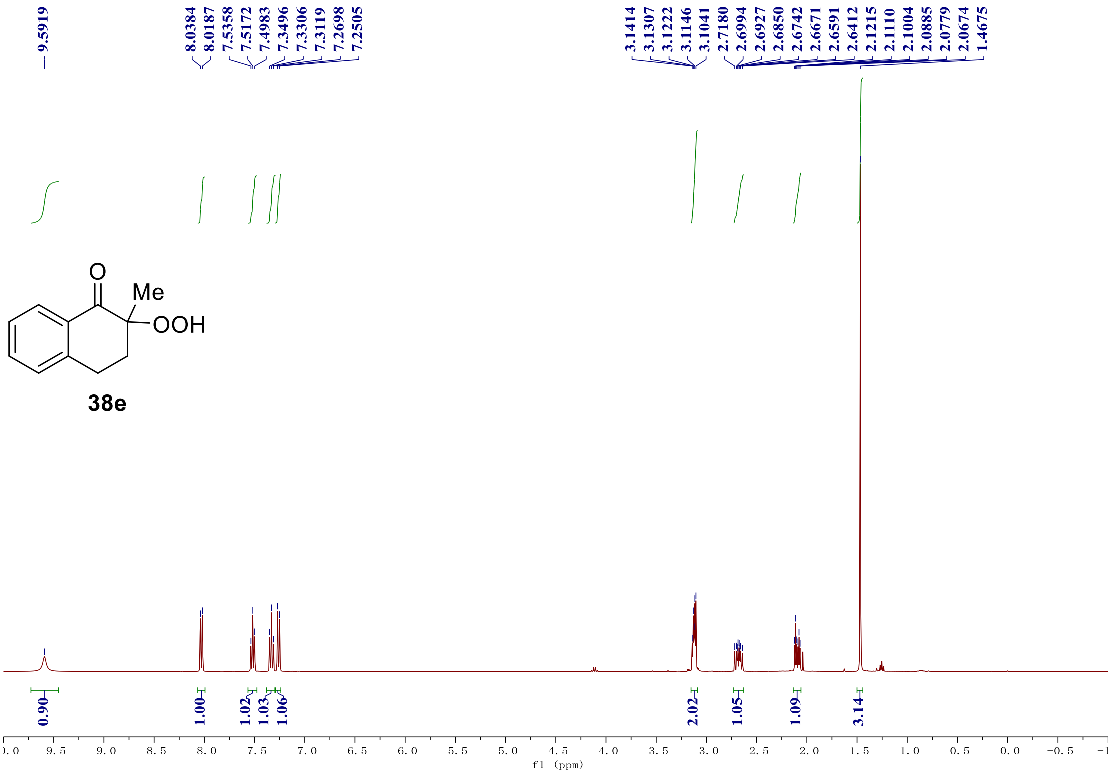

^{1}H NMR (400 MHz, $CDCl_3$) spectrum of compound **38e**

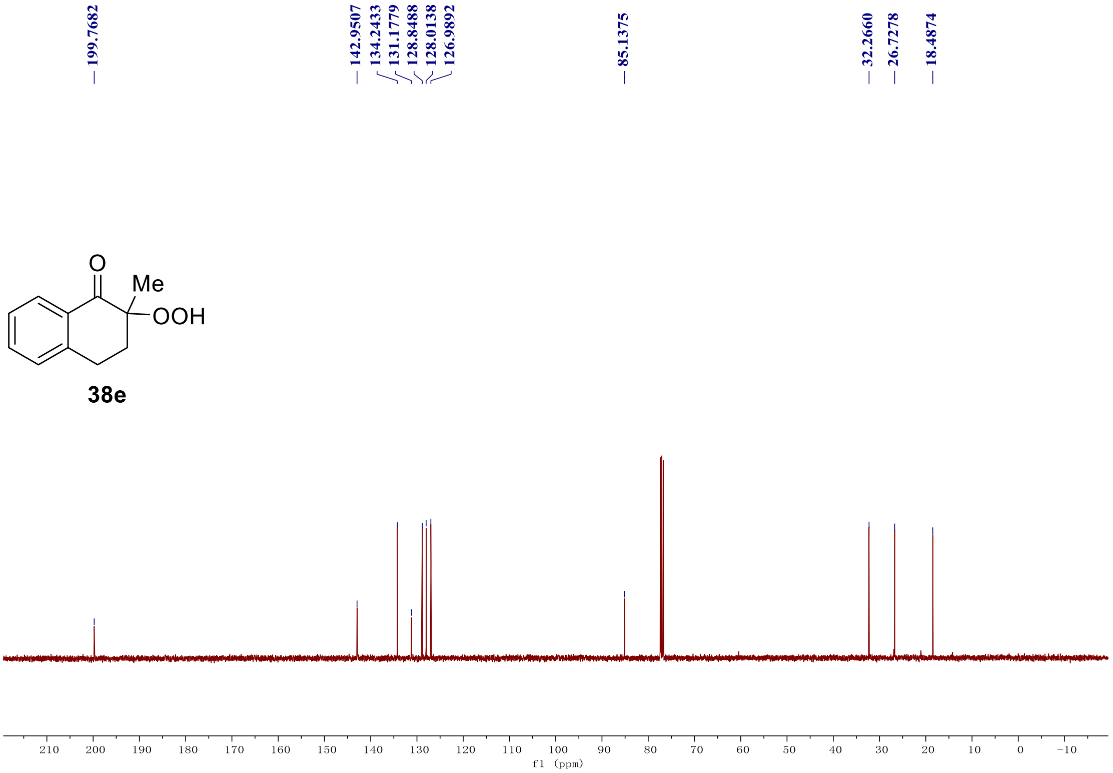

^{13}C NMR (101 MHz, $CDCl_3$) spectrum of compound **38e**

2-Hydroperoxy-2-methylpentan-3-one (38f)

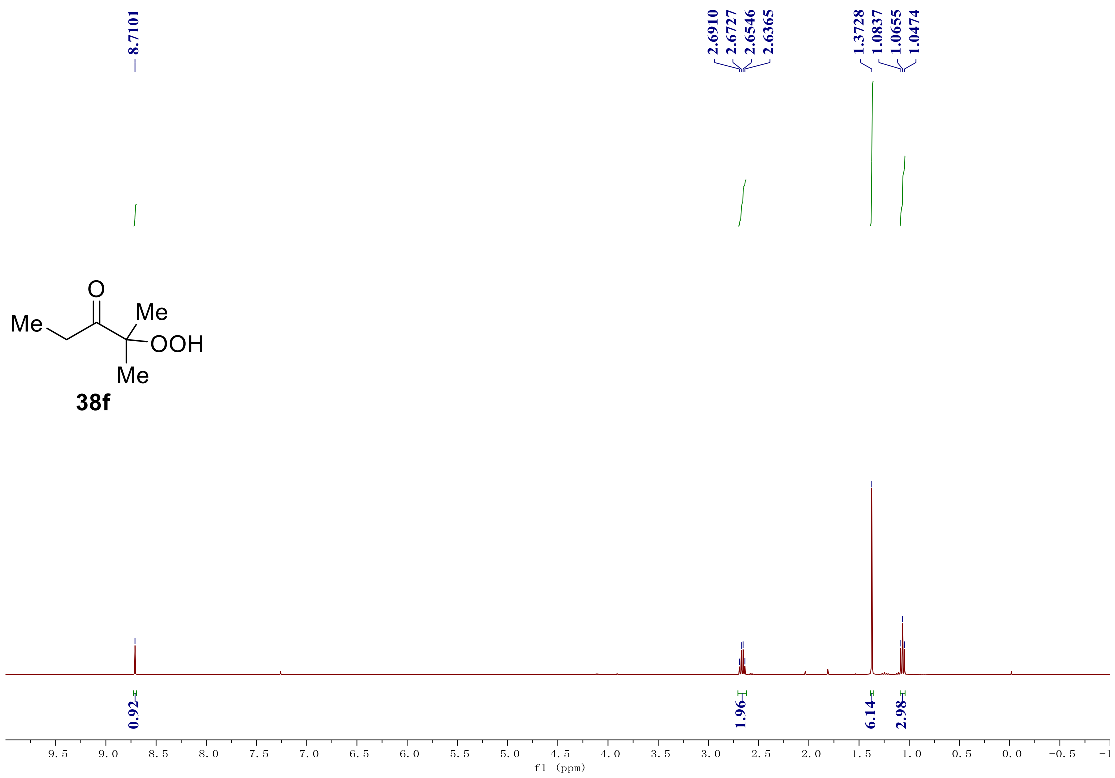

^{1}H NMR (400 MHz, $CDCl_3$) spectrum of compound **38f**

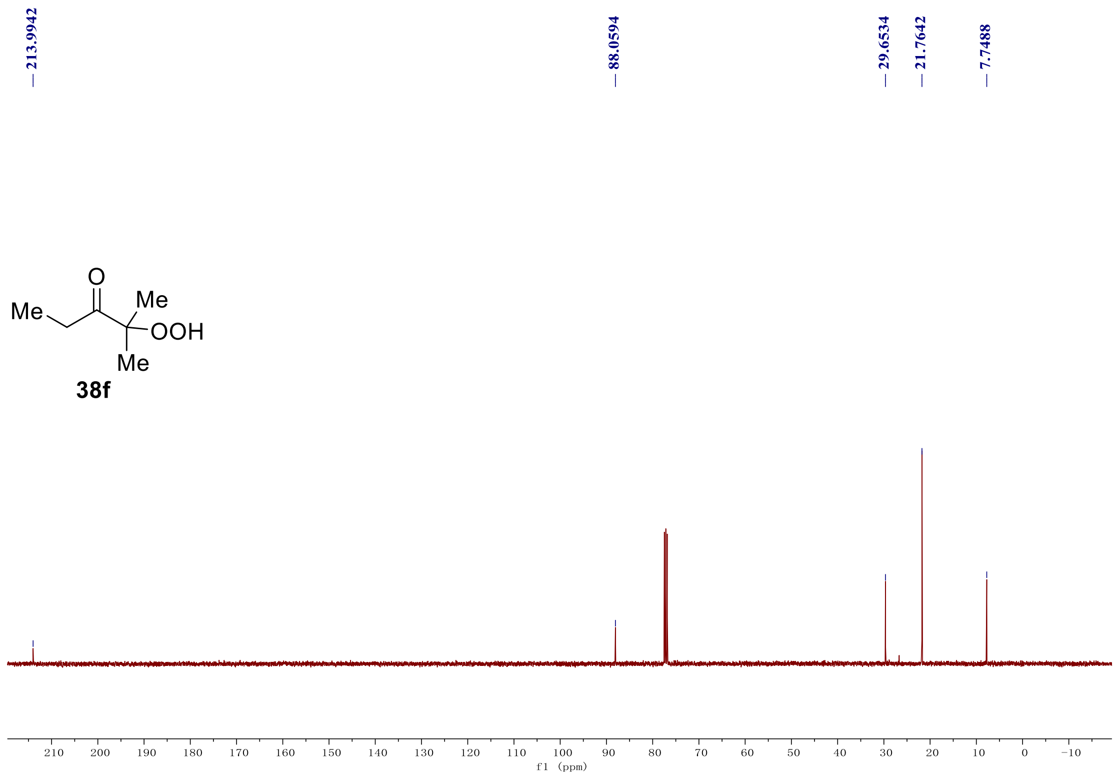

^{13}C NMR (101 MHz, $CDCl_3$) spectrum of compound **38f**

2-Hydroperoxy-2,4-dimethylpentan-3-one (38g)

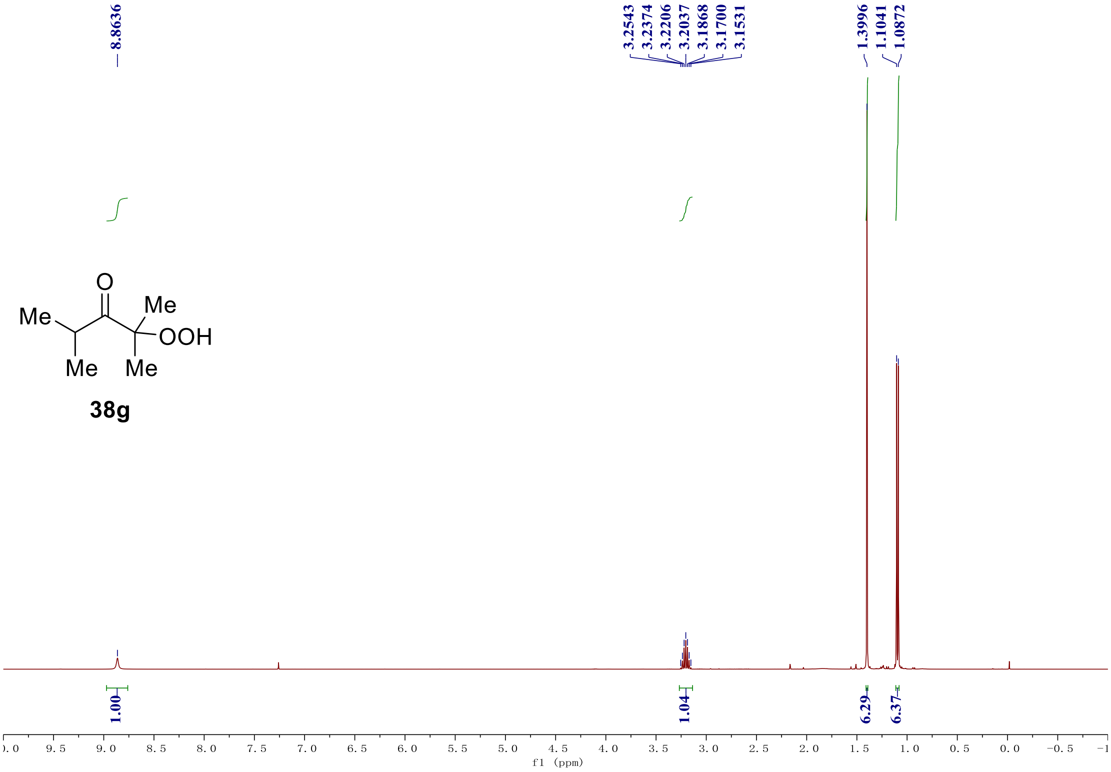

^{1}H NMR (400 MHz, $CDCl_3$) spectrum of compound **38g**

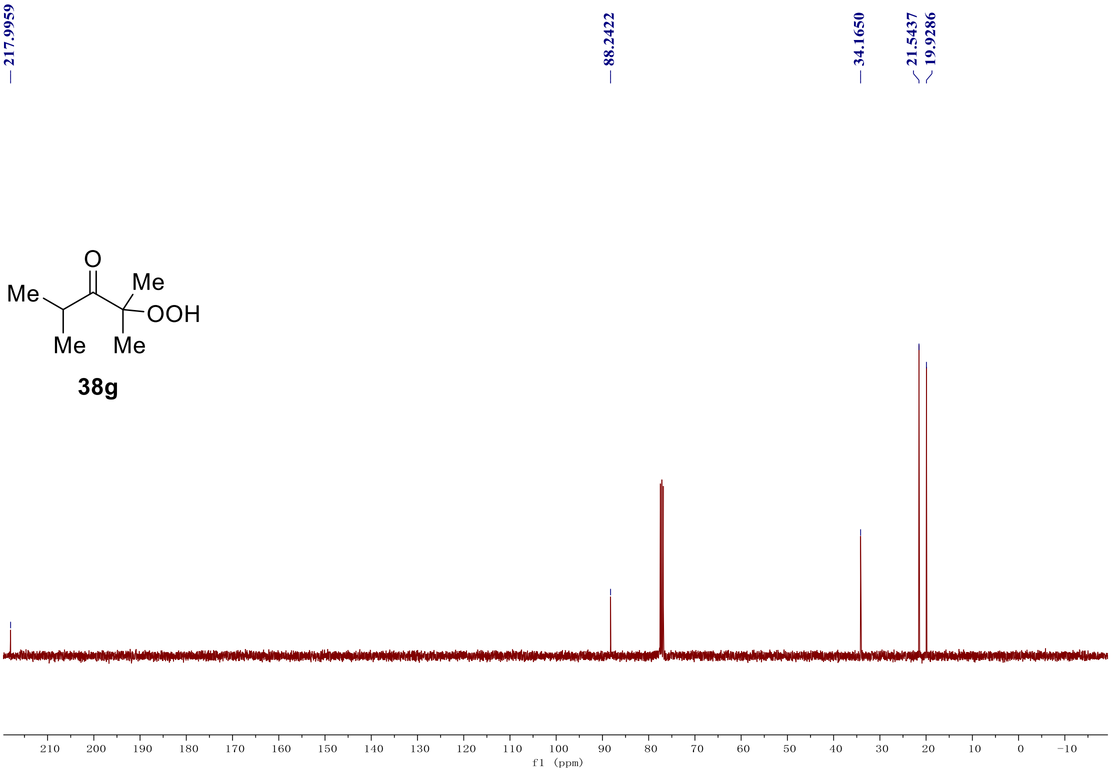

^{13}C NMR (101 MHz, $CDCl_3$) spectrum of compound **38g**

2-Hydroperoxy-2,6-dimethylcyclohexan-1-one (38h)

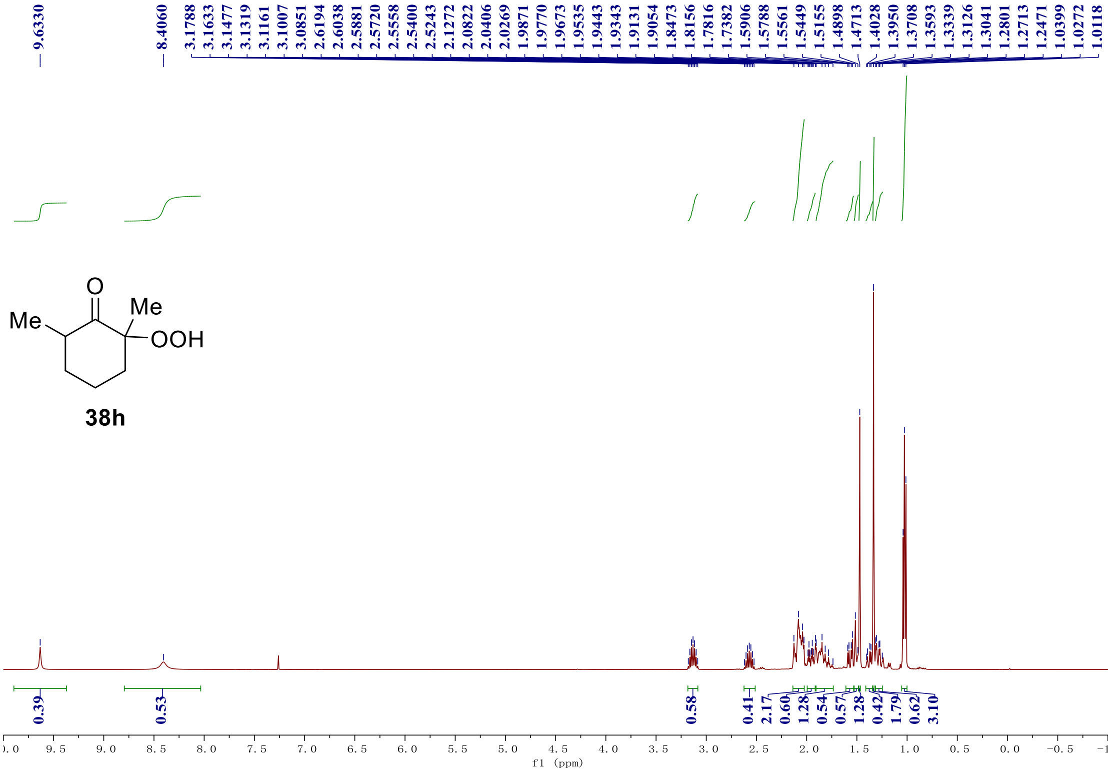

^{1}H NMR (400 MHz, $CDCl_3$) spectrum of compound **38h**

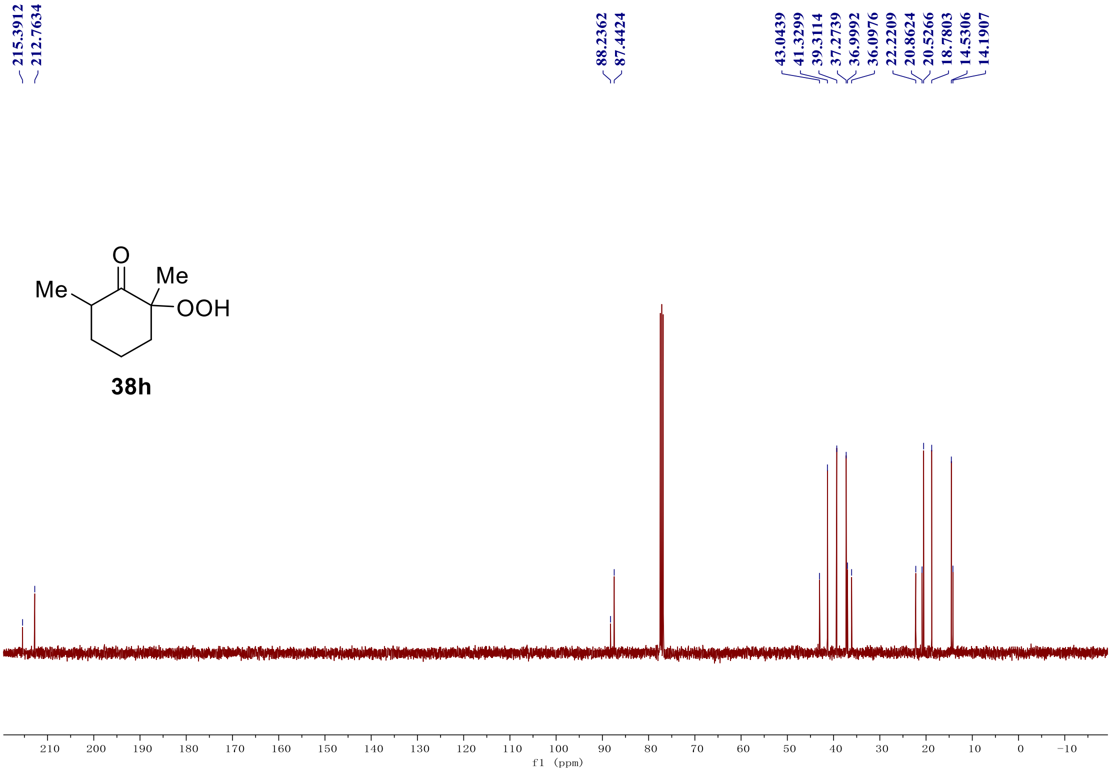

^{13}C NMR (101 MHz, $CDCl_3$) spectrum of compound **38h**

(2*S*, 5*R*)-2-Hydroperoxy-2-isopropyl-5-methylcyclohexan-1-one (38i)

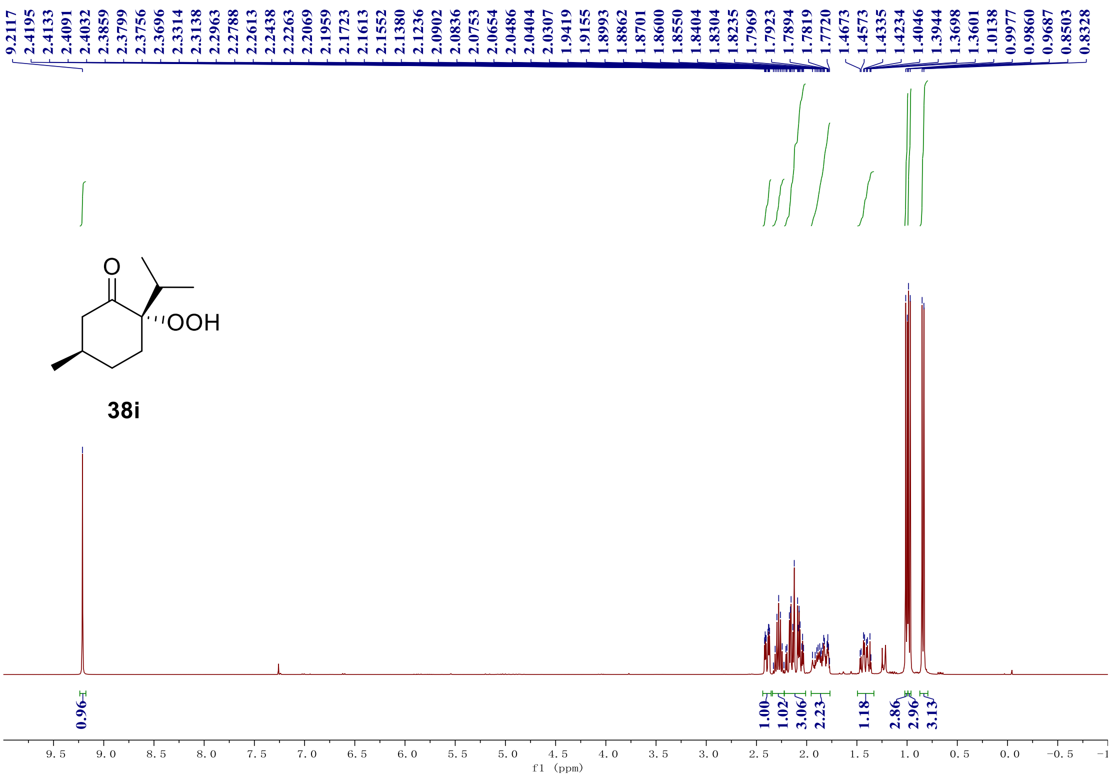

^{1}H NMR (400 MHz, $CDCl_3$) spectrum of compound **38i**

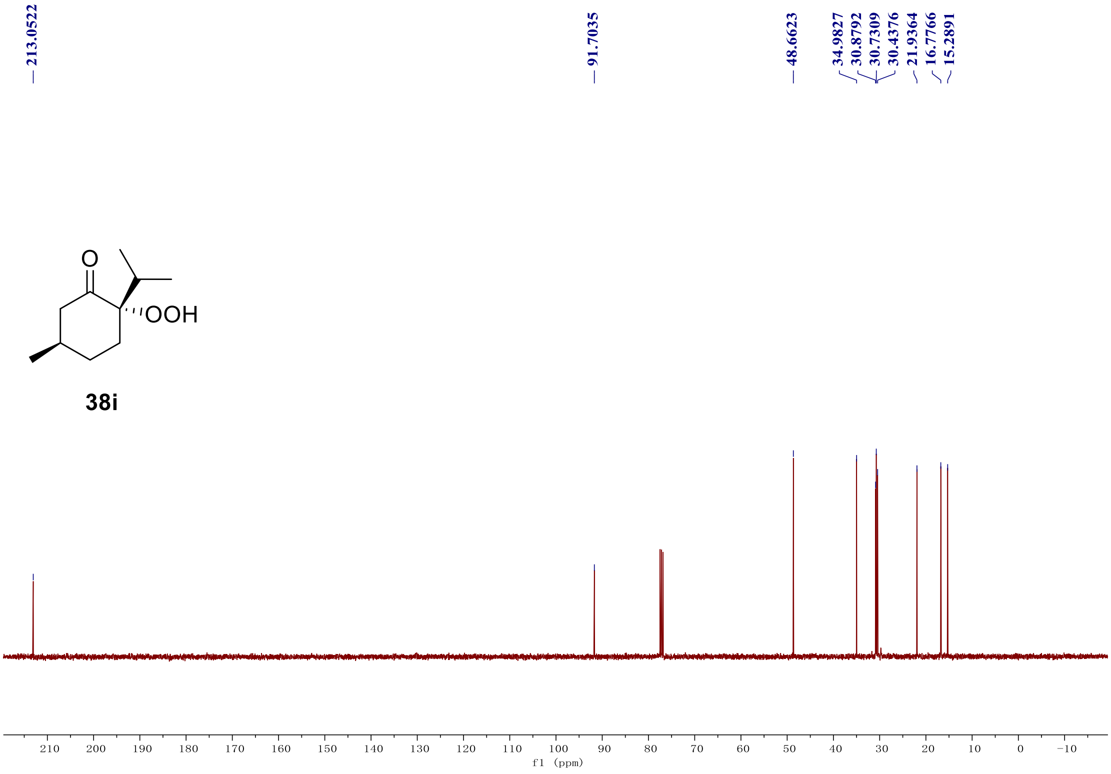

^{13}C NMR (101 MHz, $CDCl_3$) spectrum of compound **38i**

(2*R*, 5*R*)-2-Hydroperoxy-2-isopropyl-5-methylcyclohexan-1-one (38j)

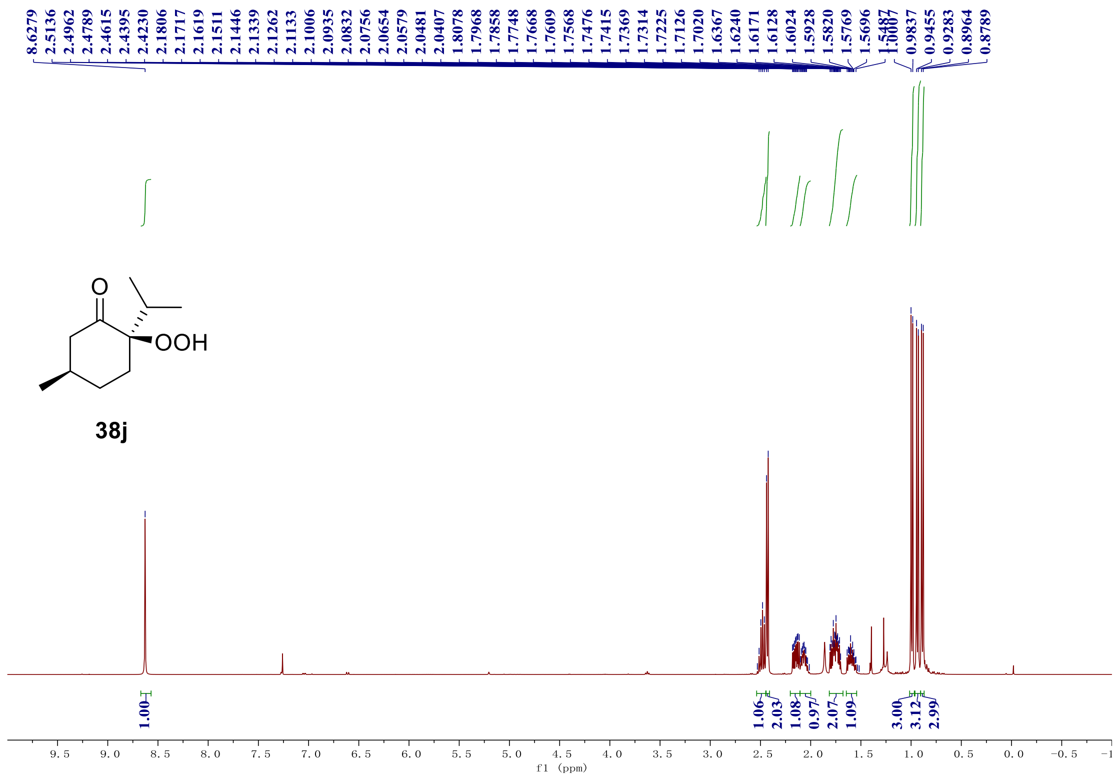

^{1}H NMR (400 MHz, $CDCl_3$) spectrum of compound **38j**

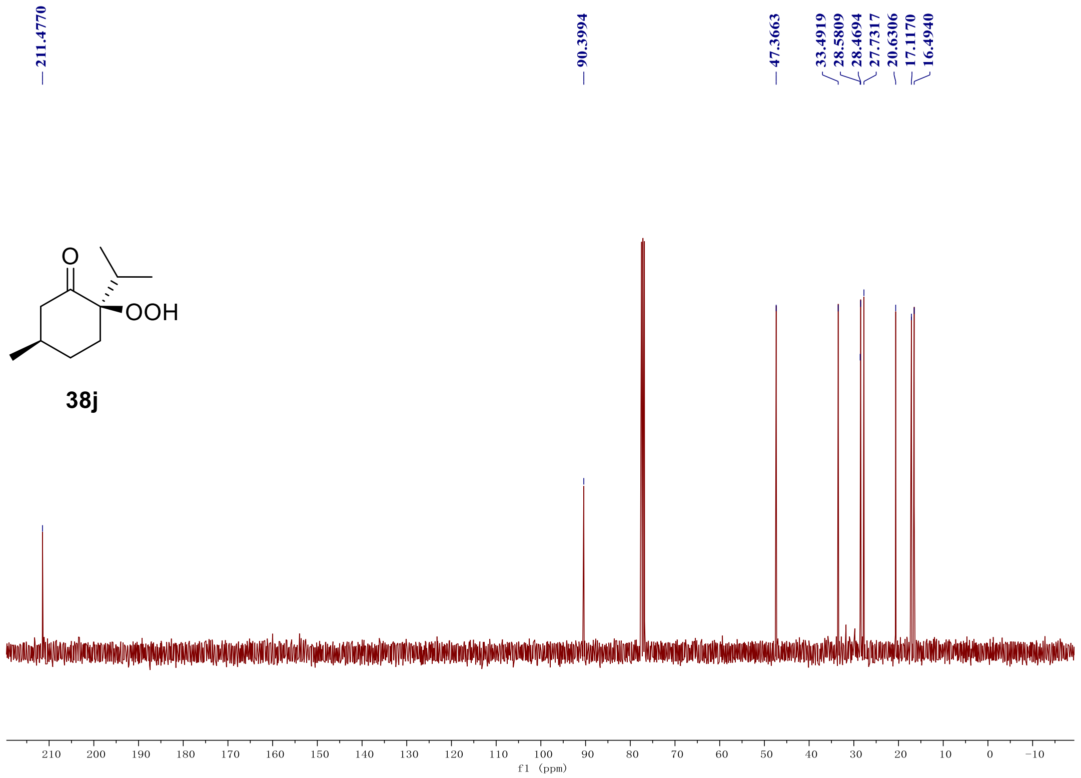

^{13}C NMR (101 MHz, $CDCl_3$) spectrum of compound **38j**

2-Ethyl-2-hydroperoxycyclohexan-1-one (38k)

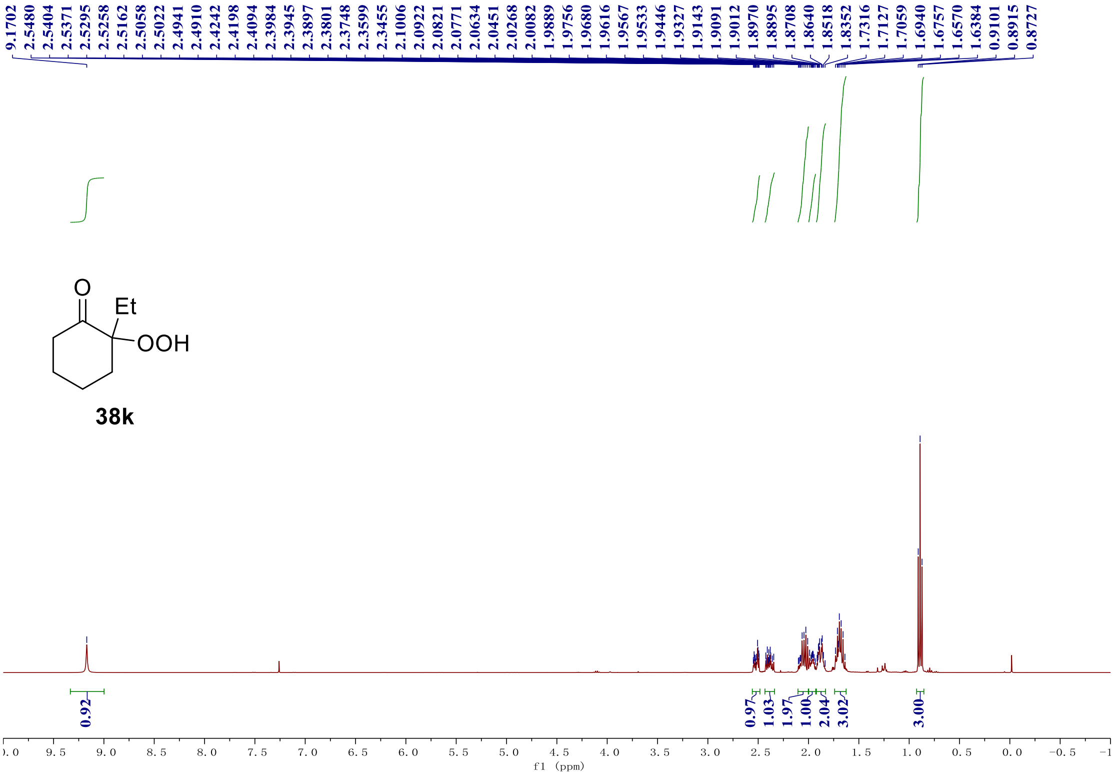

^{1}H NMR (400 MHz, $CDCl_3$) spectrum of **38k**

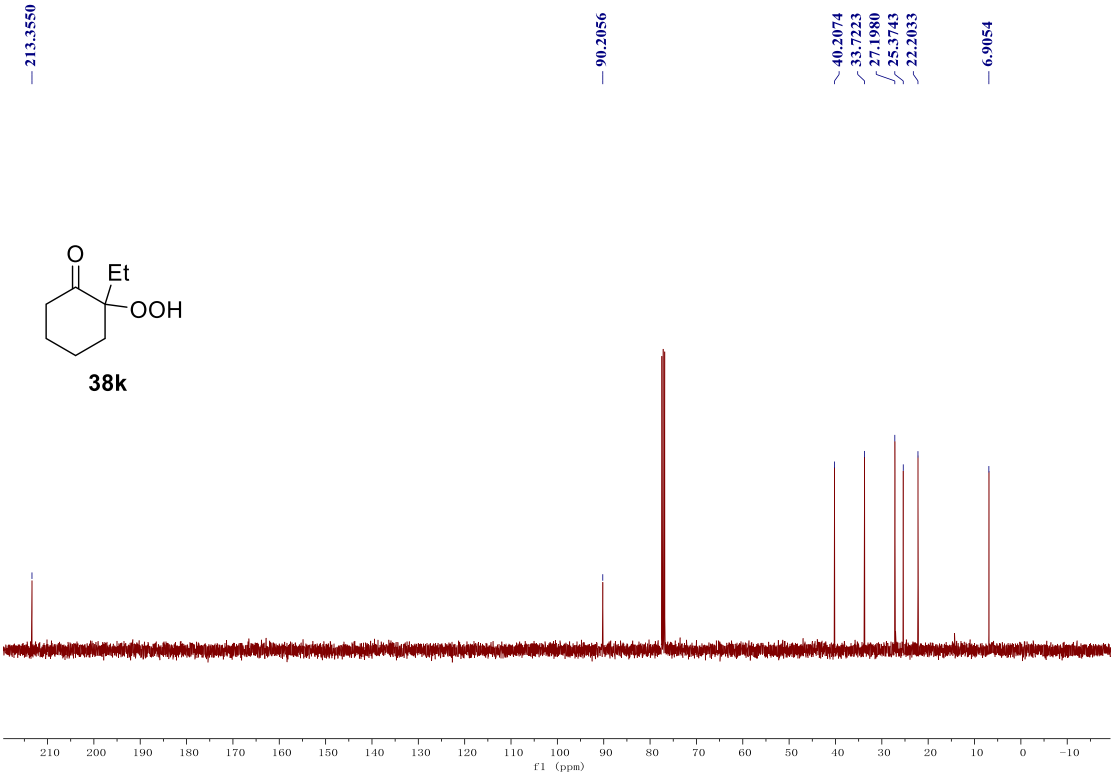

^{13}C NMR (101 MHz, $CDCl_3$) spectrum of **38k**

(2*S*, 5*R*)-2-Hydroxy-2-isopropyl-5-methylcyclohexan-1-one (39i)

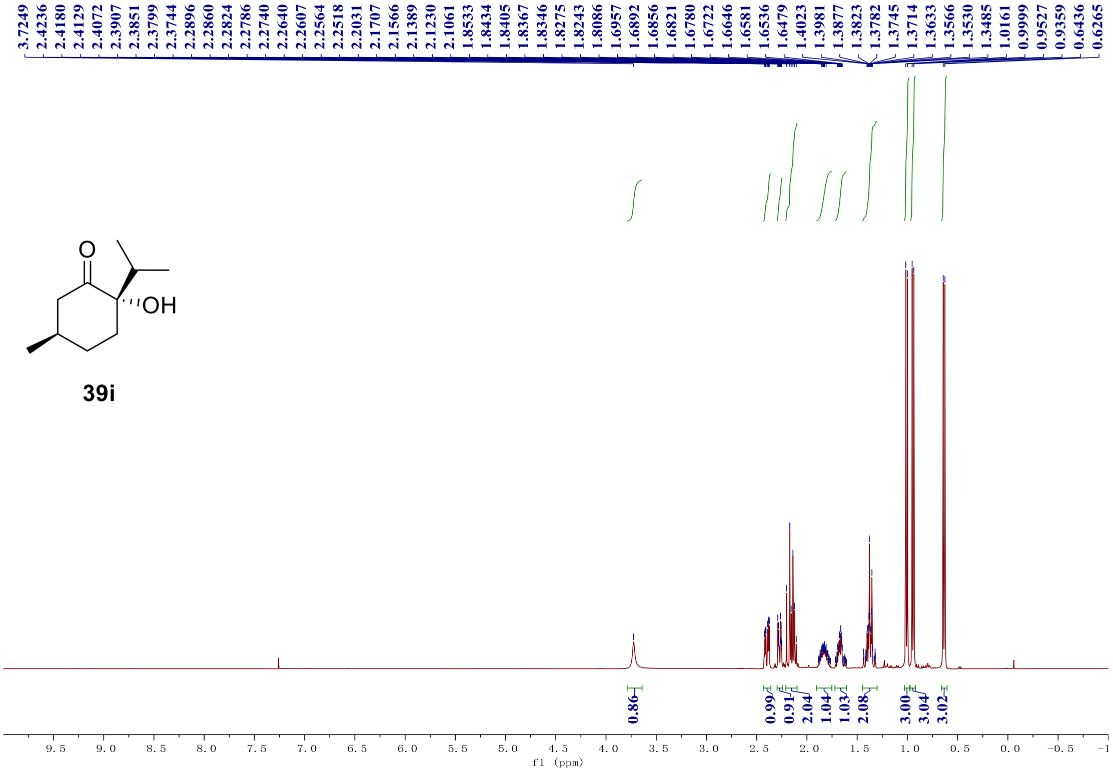

^{1}H NMR (400 MHz, $CDCl_3$) spectrum of compound **39i**

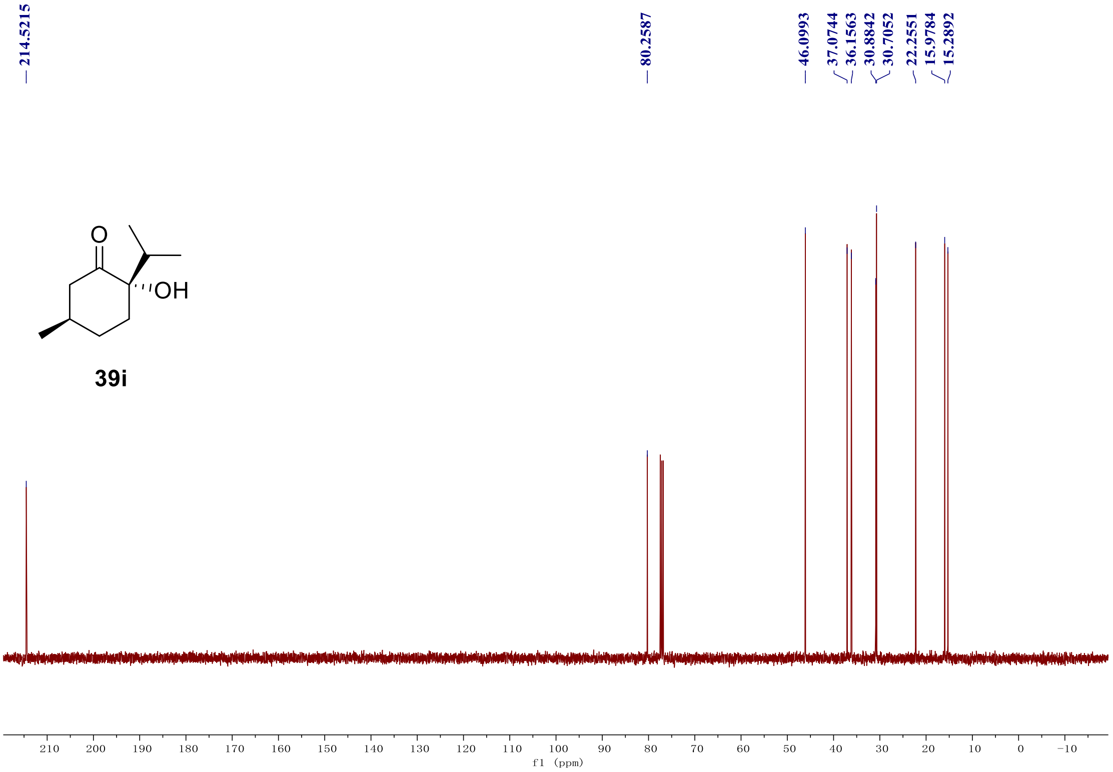

^{13}C NMR (101 MHz, $CDCl_3$) spectrum of compound **39i**

(2*R*, 5*R*)-2-Hydroxy-2-isopropyl-5-methylcyclohexan-1-one (39j)

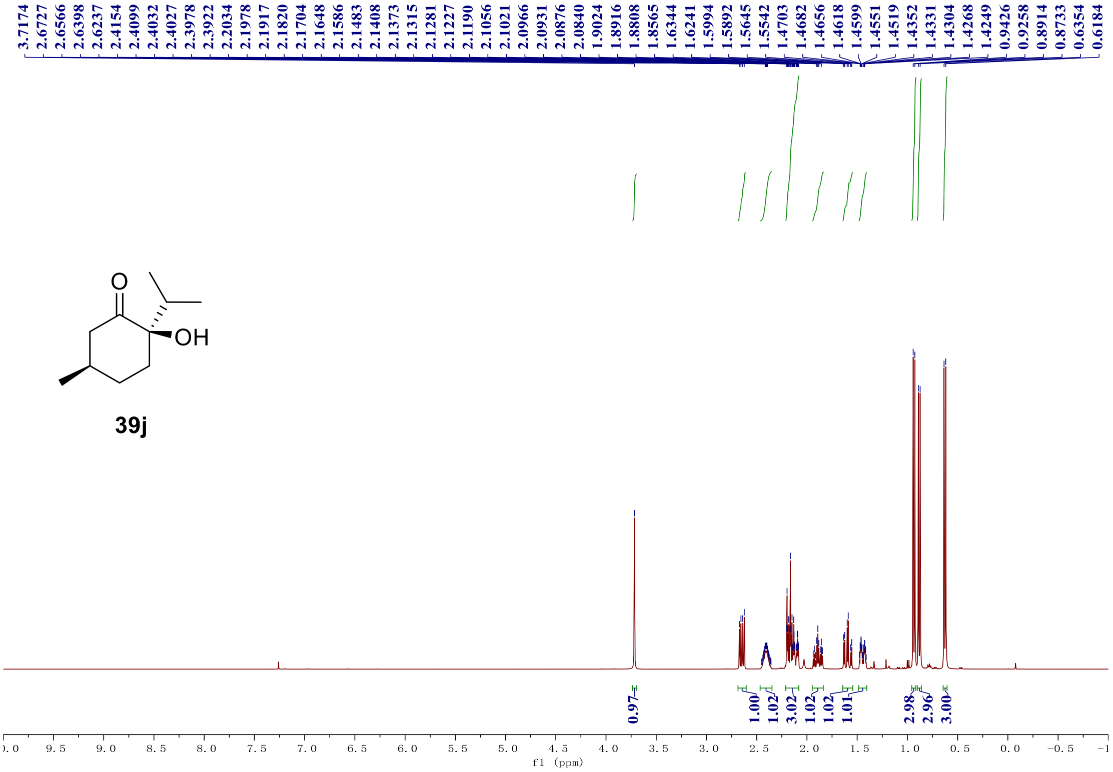

^{1}H NMR (400 MHz, $CDCl_3$) spectrum of compound **39j**

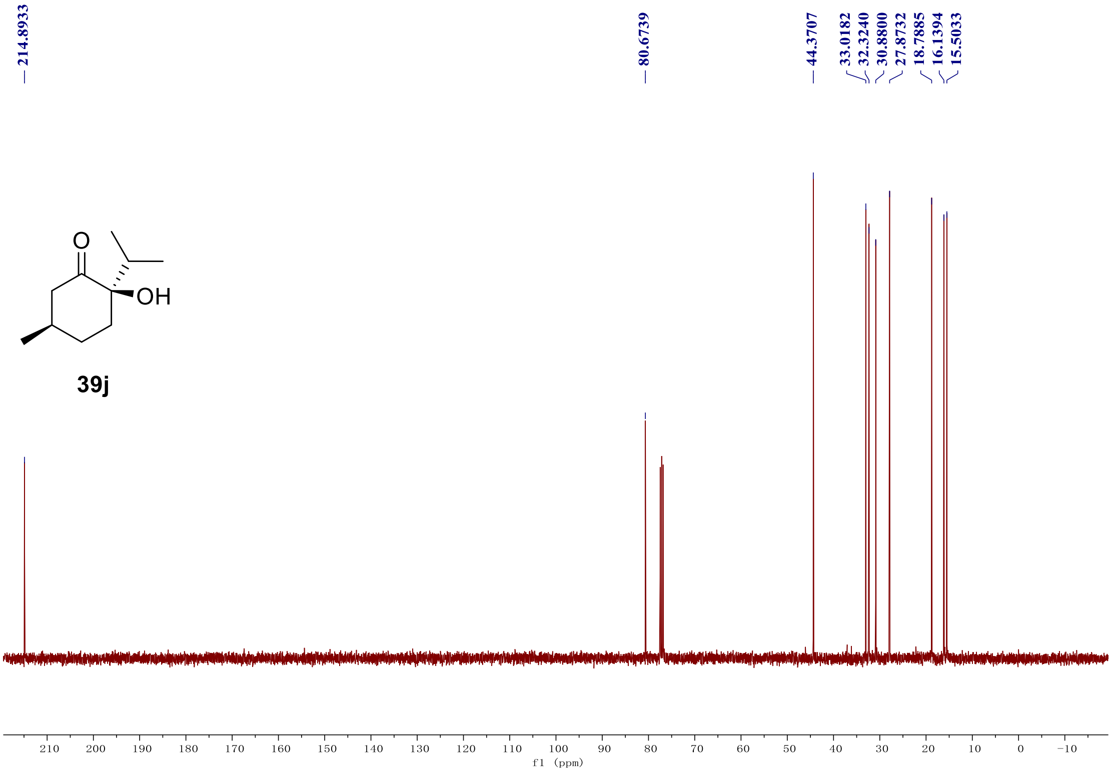

^{13}C NMR (101 MHz, $CDCl_3$) spectrum of compound **39j**

2-Hydroperoxy-2-methylcyclohexan-1-ol (40)

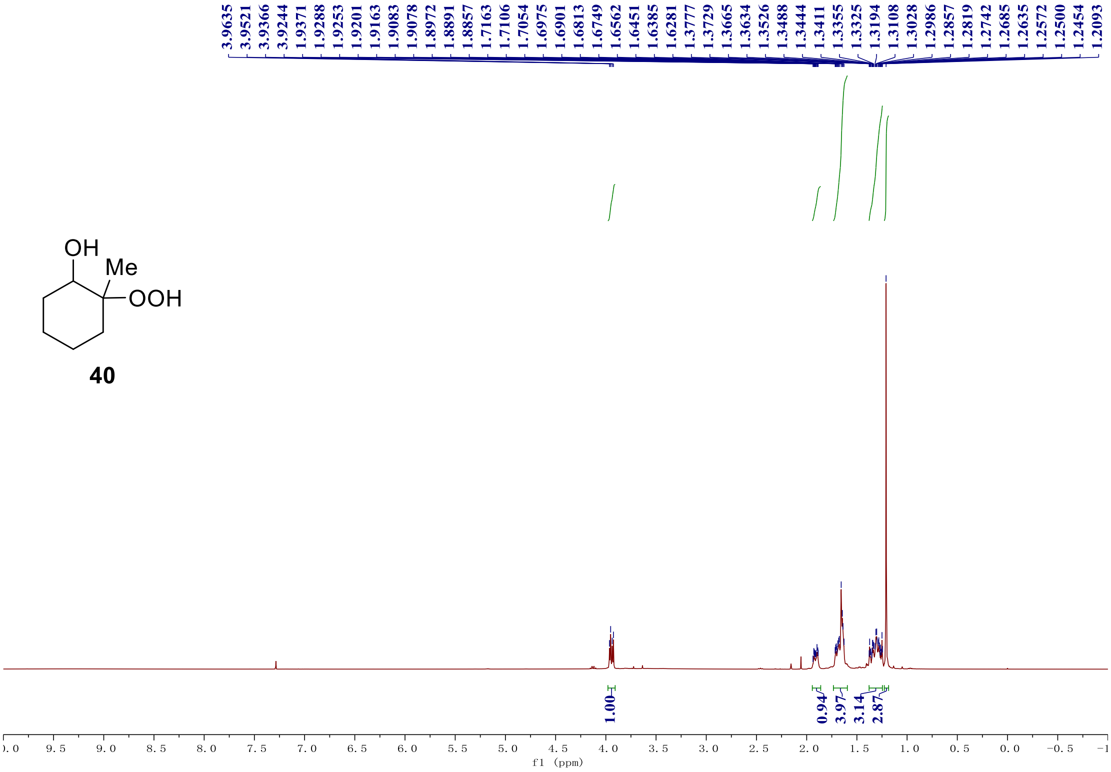

^{1}H NMR (400 MHz, $CDCl_3$) spectrum of compound **40**

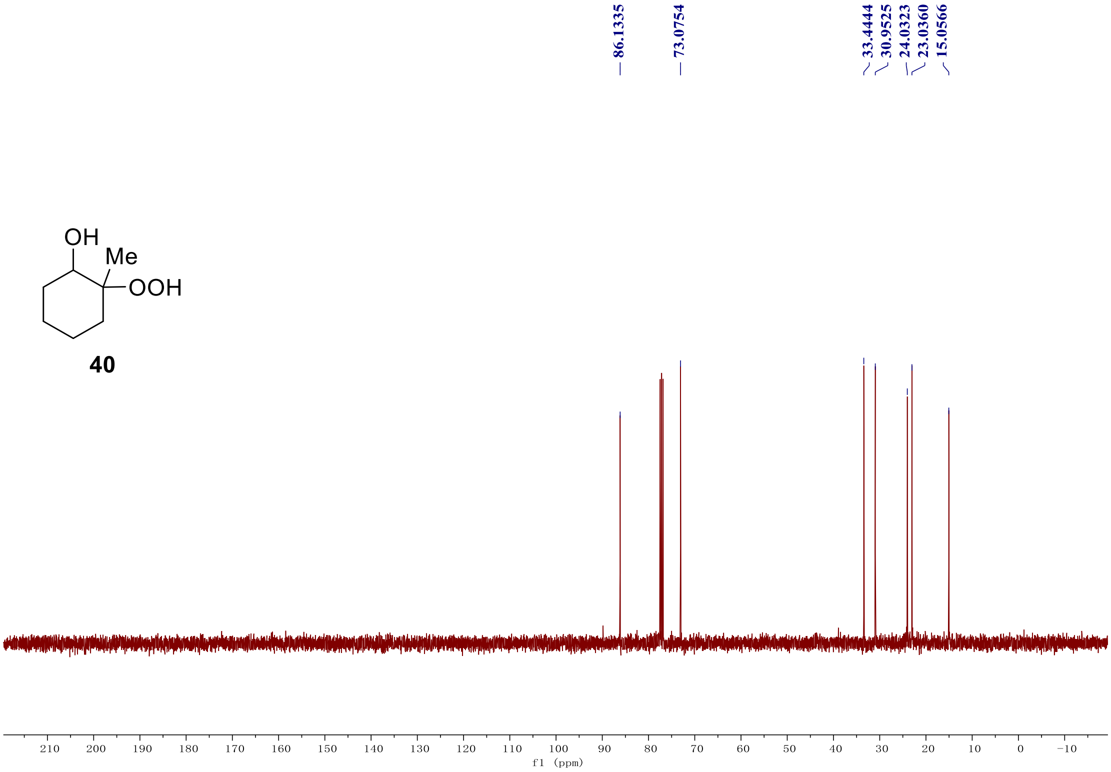

^{13}C NMR (101 MHz, $CDCl_3$) spectrum of compound **40**

2-Methyl-2-((triethylsilyl)peroxy)cyclohexan-1-ol (41)

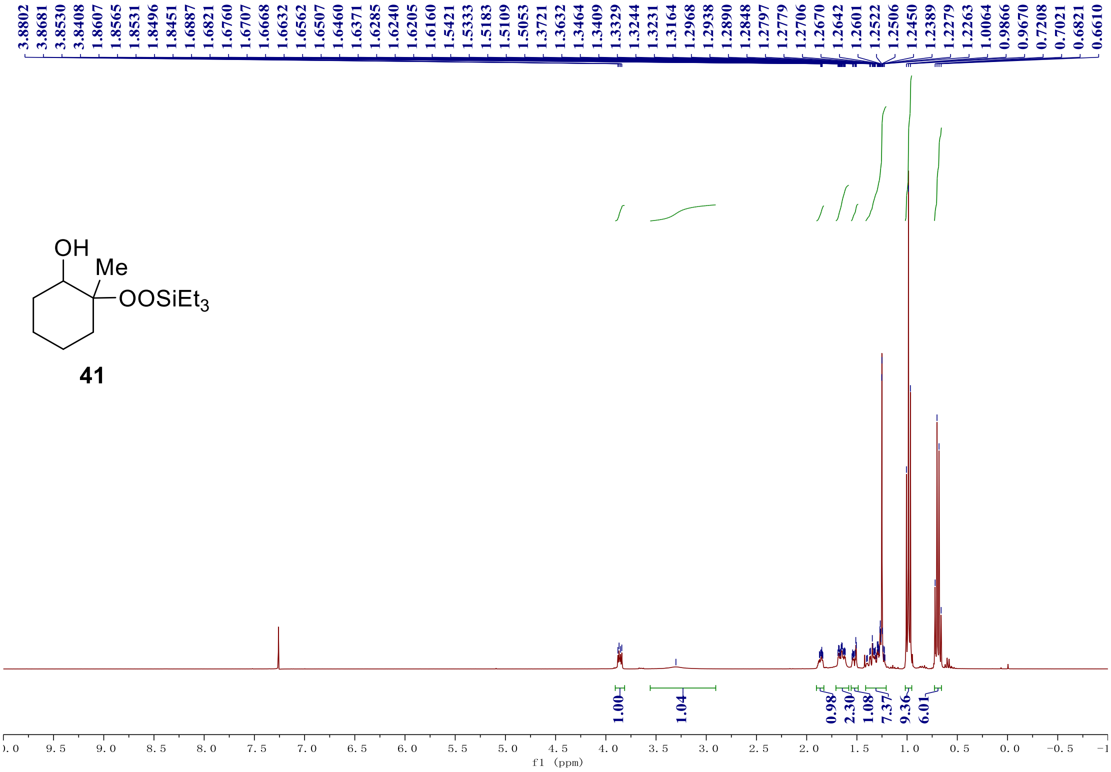

^{1}H NMR (400 MHz, $CDCl_3$) spectrum of compound **41**

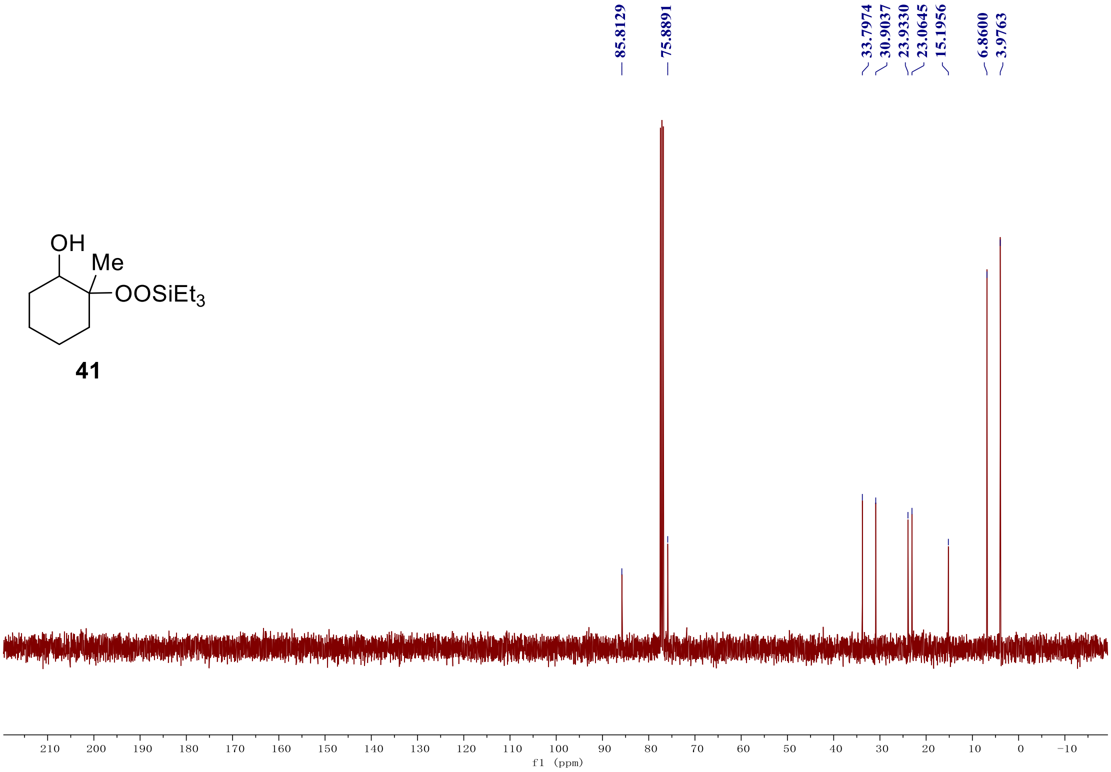

^{13}C NMR (101 MHz, $CDCl_3$) spectrum of compound **41**

N-(2-hydroperoxy-2-methylcyclohexyl)-4-methylbenzenesulfonamide (42)

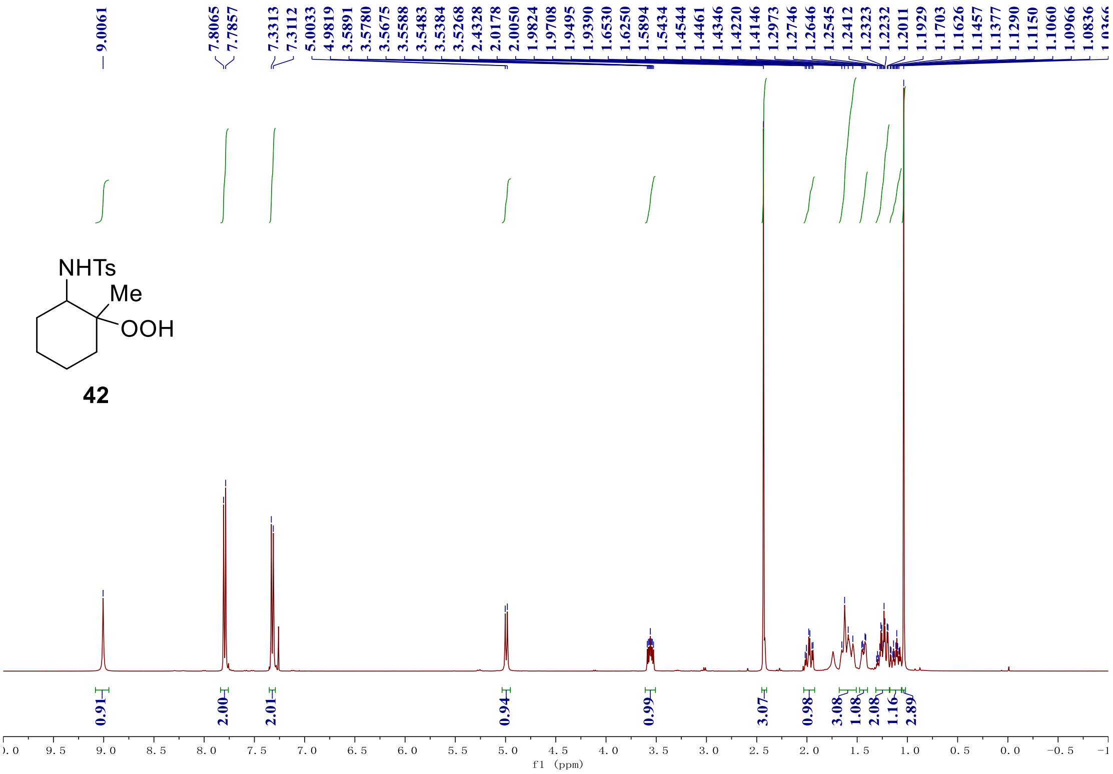

^{1}H NMR (400 MHz, $CDCl_3$) spectrum of compound **42**

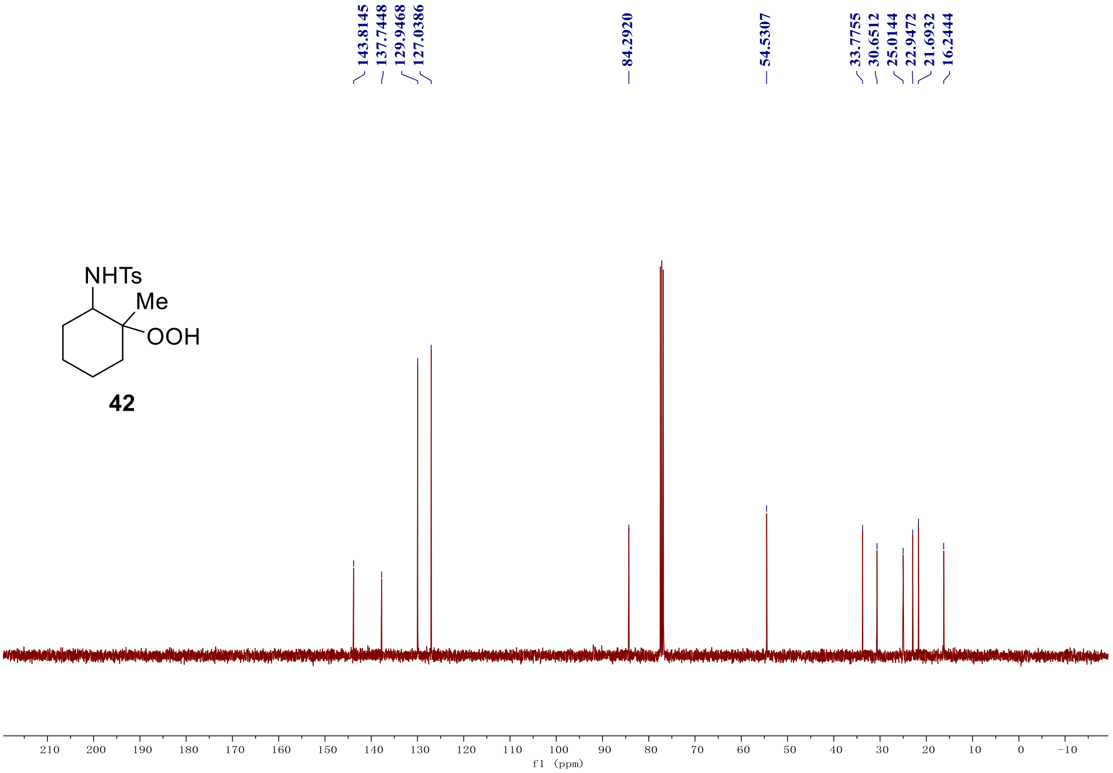

^{13}C NMR (101 MHz, $CDCl_3$) spectrum of compound **42**
